# Synthesis, In Vitro Antiproliferative Activity, and In Silico Evaluation of Novel Oxiranyl-Quinoxaline Derivatives

**DOI:** 10.3390/ph15070781

**Published:** 2022-06-23

**Authors:** Vincent Montero, Marc Montana, Omar Khoumeri, Florian Correard, Marie-Anne Estève, Patrice Vanelle

**Affiliations:** 1 Aix Marseille Univ, CNRS, ICR UMR 7273, Equipe Pharmaco-Chimie Radicalaire, Faculté de Pharmacie, CEDEX 05, 13385 Marseille, France; vincent.montero@etu.univ-amu.fr (V.M.); marc.montana@univ-amu.fr (M.M.); omar.khoumeri@univ-amu.fr (O.K.); 2 APHM, Hôpital Timone, Oncopharma, 13005 Marseille, France; 3 Aix Marseille Univ, CNRS, INP, Inst Neurophysiopathol, CEDEX 05, 13385 Marseille, France; florian.correard@univ-amu.fr (F.C.); marie-anne.esteve@univ-amu.fr (M.-A.E.); 4 APHM, Hôpital Timone, Service Pharmacie, 13005 Marseille, France; 5 APHM, Hôpital Conception, Service Central de la Qualité et de l’Information Pharmaceutiques, 13005 Marseille, France

**Keywords:** ligand-based drug design, antiproliferative activity, anticancer drug, neuroblastoma, quinoxaline, diastereoisomer, stereochemistry, R programming, docking, pharmacokinetics modeling

## Abstract

The quinoxaline core is a promising scaffold in medicinal chemistry. Multiple quinoxaline derivatives, such as the topoisomerase IIβ inhibitor XK-469 and the tissue transglutaminase 2 inhibitor GK-13, have been evaluated for their antiproliferative activity. Previous work reported that quinoxaline derivatives bearing an oxirane ring present antiproliferative properties against neuroblastoma cell lines SK-N-SH and IMR-32. Likewise, quinoxalines with an arylethynyl group displayed promising antineoplastic properties against glioblastoma and lung cancer cell lines, U87-MG and A549 respectively. Here, 40 new quinoxaline derivatives bearing an oxirane ring were synthesized using a tetrakis(dimethylamino)ethylene (TDAE) strategy and a Sonogashira cross-coupling reaction. Each reaction with TDAE furnished a pair of diastereoisomers *cis* and *trans*. These new compounds formed two series according to the substitution of position 2 on the quinoxaline core, with chlorine or phenylacetylene respectively. Each of these isomers was evaluated for antiproliferative activity against neuroblastoma cell lines SK-N-SH and IMR-32 by MTT assay. All cell viability assay results were analyzed using R programming, as well as a statistical comparison between groups of compounds. Our evaluation showed no difference in drug sensitivity between the two neuroblastoma cell lines. Moreover, *trans* derivatives were observed to display better activities than *cis* derivatives, leading us to conclude that stereochemistry plays an important role in the antiproliferative activity of these compounds. Further support for this hypothesis is provided by the lack of improvement in antineoplastic activity following the addition of the phenylacetylene moiety, probably due to steric hindrance. As a result, compounds with nitrofuran substituents from the TDAE series demonstrated the highest antiproliferative activity with IC_50_ = 2.49 ± 1.33 μM and IC_50_ = 3.96 ± 2.03 μM for compound **11a** and IC_50_ = 5.3 ± 2.12 μM and IC_50_ = 7.12 ± 1.59 μM for compound **11b** against SK-N-SH and IMR-32, respectively. Furthermore, an in silico study was carried out to evaluate the mechanism of action of our lead compounds and predict their pharmacokinetic properties.

## 1. Introduction

Neuroblastoma is a neuroendocrine tumor of the sympathetic nervous system that develops from immature nerve tissue cells called neuroblasts. With 90% of cases diagnosed under 5 years old, it is the most common extra-cranial solid tumor, and the 4th cause of cancer in children. Treatment options for this pediatric cancer rely on risk classification, depending on age at diagnosis and staging, among other factors, with surgery remaining the only effective treatment. The most threatening risk group, called “High-risk Neuroblastoma“, is a therapeutic challenge because of its frequent metastases at the time of diagnosis [1]. This group is associated in about 40% of its cases with MYCN gene amplification which is a strong predictor of poor prognosis [2]. Thus, MYCN oncogene amplification is the most important genomic feature in neuroblastoma for classification and staging. Hence, this disease requires aggressive multimodal therapy such as chemotherapy and radiation [3], often leading to multiple long-term complications [4]. Recently, anti-glycolipid disialoganglioside monoclonal antibodies (anti-GD2) like dinutuximab [5] and naxitamab [6], have been approved by the FDA as new therapeutical options for this pediatric cancer. Unfortunately, these new immunotherapies are usually restricted to children as last resort after other treatments. These other treatments are based on cytotoxic drugs such as alkylating agents like cisplatin and cyclophosphamide, topoisomerase II inhibitor etoposide, alkaloid vincristine, and anthracycline doxorubicin [7]. However, response rates to all these options being low, the need for new treatments is substantial. This led to the development of the quinoxaline derivative XK-469 [8], an antitumor agent mediating its effects by topoisomerase IIβ inhibition [9]. During clinical trials [10], this compound administered in monotherapy enabled a 14-month disease stabilization in a 14-year-old with relapsed neuroblastoma [11].

Quinoxaline derivatives show a wide range of therapeutic properties such as anti-infectious [12,13], anticancer [14,15], and many others [16] ensuring them a bright future in medicinal chemistry. Some of these derivatives are currently approved for human treatment: for instance, varenicline for tobacco cessation, brimonidine to treat open-angle glaucoma, and erdafitinib which is an FGFR inhibitor used to treat bladder cancer [17]. Others have undergone clinical trials, such as chloroquinoxaline sulphonamide (CQS) [18], a topoisomerase II poison like XK-469 [19], which has been evaluated against stage IV colorectal cancer and small-cell lung cancer.

**Figure 1:**
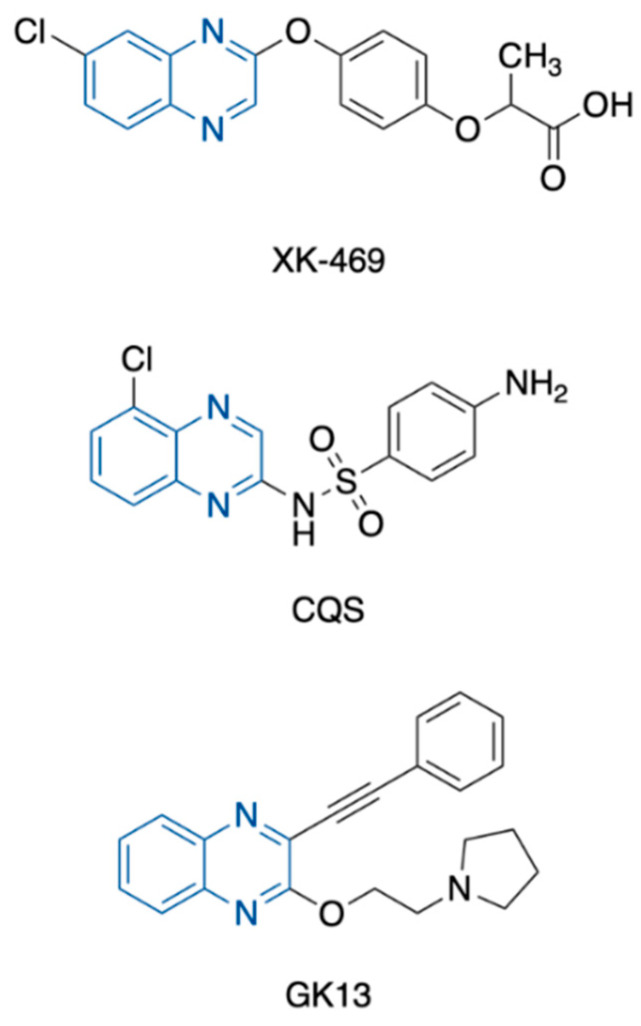
Structures of XK-469, CQS, and GK-13.

Another derivative of interest is the arylethynylquinoxaline GK-13, which demonstrated antiproliferative activity by inhibiting tissue transglutaminase 2 (TG2) [20], a ubiquitous calcium-dependent enzyme involved in apoptosis evasion and tumor cell drug resistance [21] (Figure 1).

Previous work reported that quinoxaline derivatives bearing an oxirane ring showed interesting antiproliferative activity, with IC_50_ = 3.9 ± 0.2 μM and IC_50_ = 5.0 ± 0.9 μM for the most active compound, similar to the reference XK-469 (IC_50_ = 4.6 ± 1.0 μM and IC_50_ = 13.0 ± 2.9 μM) against neuroblastoma cell lines SK-N-SH and IMR-32, respectively [22]. Epoxides are an appropriate choice in the design of anticancer agents as they resemble aziridine ring, a well-known class of cytotoxic agents [23]. Another work reported that quinoxaline derivatives bearing an arylethynyl moiety display IC_50_ of 3 μM against glioblastoma and lung cancer cell lines, U87-MG and A549 respectively [24]. Since our research activity focuses on the preparation of new potentially bioactive compounds [25,26,27], this work aimed to synthesize and evaluate from both in vitro and in silico perspectives, novel quinoxaline derivatives. These derivatives bear an oxirane ring substituted by a variety of aromatic and non-aromatic groups to evaluate the influence of this substitution. To determine whether the presence of an arylethynyl moiety can improve the antiproliferative activity of oxiranyl-quinoxaline derivatives against neuroblastoma cell lines SK-N-SH and IMR-32, two series were evaluated in this work with different substitutions on the position 2 of the quinoxaline core.

## 2. Results and Discussion

### 2.1. Chemistry

**Figure 2:**
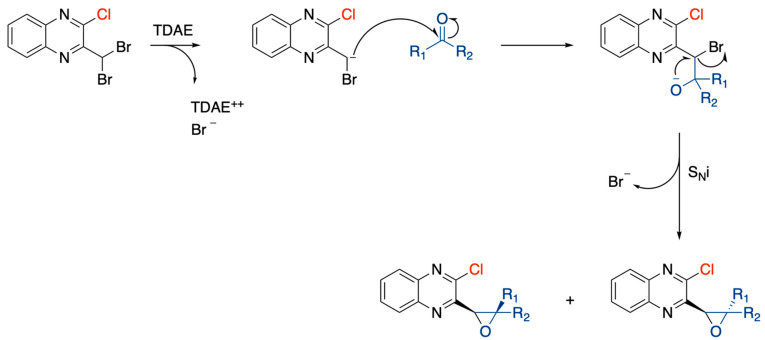
Mechanism of epoxide formation via TDAE reaction.

A first series (TDAE series) of epoxide diastereoisomers, with chlorine in position 2 of the quinoxaline core, was synthesized from 2-chloro-3-(dibromomethyl)quinoxaline and various carbonyl compounds using the organic electron donor TDAE to form the oxirane ring. The mechanism of this reaction is divided into two steps: firstly, TDAE in presence of dibromomethyl quinoxaline forms an anion that attacks the carbonyl to form an intermediate species; secondly, an intramolecular nucleophilic substitution (SNi) forms the epoxide ring (Figure 2).

**Figure 3:**
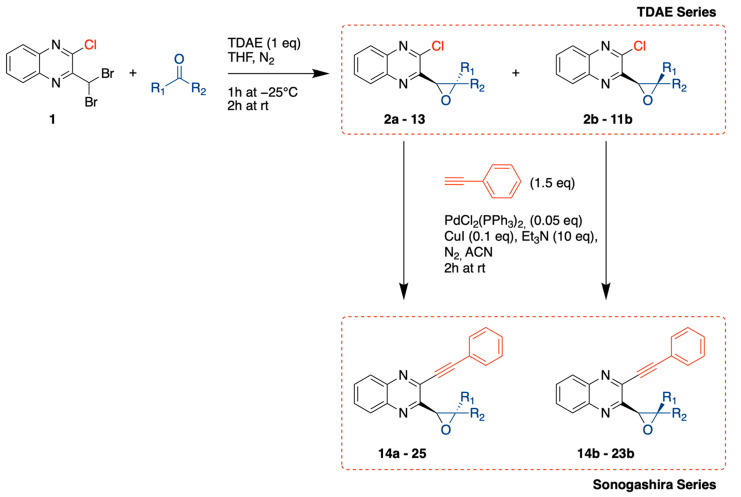
General synthesis procedure of novel antiproliferative quinoxaline derivatives.

Carbonyl compounds were chosen to cover a broad spectrum of chemical properties such as aromatic and non-aromatic groups, halogenated substituents in *ortho*, *meta*, *para* positions, electron-donating, and electron-withdrawing groups. This allowed us to obtain a mixture of *cis* and *trans* diastereoisomers that led to 20 new compounds (**2a–13**) after purifying each isomer by flash chromatography (Figure 3). Proportions of each diastereoisomer formed, as determined by 1H-NMR, were distributed nearly 50/50 between *cis* and *trans* isomers. For compounds **3**, **6,** and **12**, the *cis* isomer could not be retrieved after purification of the reaction mixture.

A second series (Sonogashira series) was obtained using a Sonogashira cross-coupling reaction with phenylacetylene on each purified isomer from the previous series. This enabled us to change chlorine in position 2 to the arylethynyl moiety, thereby obtaining 20 supplementary new compounds (**14a–25**) (Figure 3).

In the end, 40 novel quinoxaline derivatives were synthesized, enabling us to evaluate multiple parameters: firstly, the influence of the position 2 substitution of the quinoxaline core to determine whether the addition of an arylethynyl group to the oxirane ring improves antiproliferative activity; secondly, the influence of stereochemistry on this biological activity since we characterized a pair of diastereoisomers *cis* and *trans*; lastly, the influence of the variation of R-groups substituting the epoxide on the antiproliferative activity.

### 2.2. In Vitro Antiproliferative Activity Evaluation

The antiproliferative activity of all derivatives was evaluated by cell survival experiments against two neuroblastoma cell lines, SK-N-SH and IMR-32 respectively, using conventional tetrazolium reduction assay as per previous work [22,24]. Both cell lines are of human origin, but they differ by their primary origin site, their resistance profile, and oncogene amplification [28]. SK-N-SH are female cells expressing the efflux pump glycoprotein-p (P-gp) responsible for multi-drug resistance but do not display the MYCN oncogene amplification associated with poor prognosis. On the contrary, IMR-32 are male cells that do not express the P-gp but display a native amplification of the MYCN oncogene [29] involved in about 20% of neuroblastoma cases and are associated with advanced disease and unfavorable biology [2]. Thus, the comparison of the results between both cell lines will allow us to anticipate the drug resistance profile of our compounds.

The obtained data were analyzed with R programming [30], to determine IC_50_ (concentration that inhibits 50% of cell proliferation) of all compounds. Compounds were classified into three groups according to their IC_50_ on both cell lines: good activity (<30 μM), low activity (between 30 and 100 μM), and no activity (>100 μM).

The highest tested concentration being 100 μM, IC_50_ was not reached for some compounds (**4a**, **16a**, **16b**, **17a**, **17b**, **18a**, **19b**, **21a**, **21b**, **22b**). They are presented in Table 1 as IC_50_ > 100 μM and were considered not to have antiproliferative activity. Therefore, these molecules were excluded from further statistical testing.

Among the 40 new compounds synthesized, 6 compounds from the TDAE series (**2a**, **5a**, **6a**, **7a**, **11a**, **11b**) showed good antiproliferative activity (<30 μM) on both cell lines, with IC_50_ ranging from 2.49 μM to 26.9 μM against SK-N-SH, and from 3.96 μM to 22.69 μM against IMR-32. Compounds 11a and 11b even displayed better cytotoxic activity than the reference XK-469 (4.6 ± 1.0 μM and 13.0 ± 2.9 μM against SK-N-SH and IMR-32, respectively [22]). Likewise, 8 compounds of the Sonogashira series (**14a**, **14b**, **15a**, **20a**, **20b**, **23a**, **23b**, **25**) showed a similar range of activities from 9.5 μM to 25.35 μM and 7.26 μM to 25.65 μM against SK-N-SH and IMR32 neuroblastoma cells, respectively.

The *trans* isomers bearing an epoxide substituted with trifluoromethyl benzene did not display any antiproliferative activity against both cell lines in the TDAE (**4a**) or Sonogashira (**16a**) series. Out of the 19 other derivatives from the TDAE series, 12 resulted in total or partial loss of activity in the Sonogashira series by combining the arylethynyl moiety (**16b**, **17a**, **17b**, **18a**, **19a**, **19b**, **21a**, **21b**, **22b**, **23a**, **23b**, **24a**). This combination resulted in the loss of activity for all halogen substituents (**17a**, **17b**, **18a**, **19a**, **19b**), except for fluorine in the *meta* position (**20a**, **20b**), on which it had the opposite effect by improving its antiproliferative activity. These variations are likely due to the steric hindrance of the phenylacetylene group. In contrast, it improved the antiproliferative activity of the remaining 6 derivatives (**14a**, **14b**, **15a**, **20a**, **20b**, **25**).

**Figure 4:**
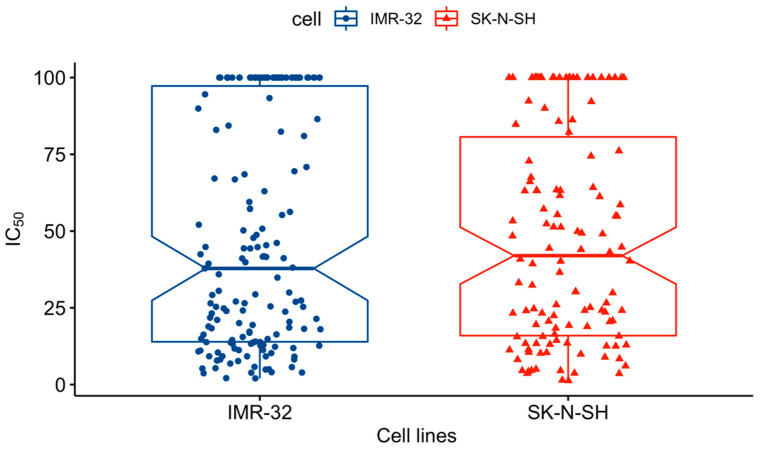
IC_50_ distribution between cell lines.

Since each molecule was tested against two different cell lines, we explored whether the antiproliferative activity of our derivatives was significantly different between them. Data visualization of all MTT assay results that were carried out (Figure 4) did not reveal obvious differences in IC_50_ distribution between cell lines. Statistical analysis led us to the conclusion that antiproliferative activities did not significantly differ between SK-N-SH and IMR-32, with *p*-value = 0.09. This result suggests that our compounds are probably not substrates of the efflux pump P-gp responsible for drug resistance. From this conclusion, we did not further consider IC_50_ against SK-N-SH and IMR-32 apart for statistical testing.

**Figure 5:**
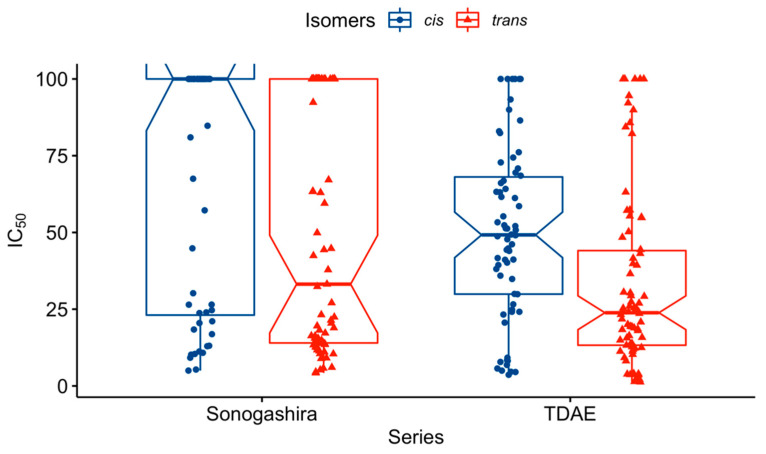
IC_50_ distribution between isomers of both series.

Visualization of the distribution between diastereoisomers of both series as above seems to indicate a difference between them (Figure 5). Statistical comparison of TDAE series distribution and Sonogashira’s returned *p*-value < 0.01. Regarding the difference in activity between *cis* and *trans*, the same test was performed and returned a *p*-value < 0.01. This significant difference in IC_50_ suggested that *trans* isomers are significantly more active than *cis* isomers. All these results support our hypothesis that stereochemistry is key to our compounds’ antiproliferative activity. Therefore, we divided into four groups each isomer from both series for further statistical comparisons: *trans* isomers of the TDAE series, *cis* isomers of the TDAE series, *trans* isomers of the Sonogashira series, and *cis* isomers of the Sonogashira series. Since compounds **13** and **25** are neither *trans* isomers nor *cis* ones, we compared them in each group according to their belonging series.

**Figure 6:**
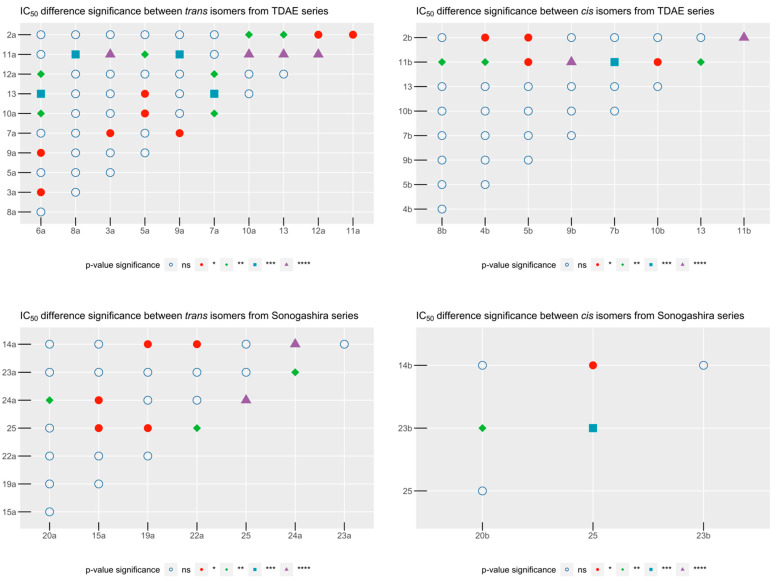
Dunn’s test results. *p*-value significance: *p*-value significances are represented as different shapes going from the highest *p*-value to the lowest as follows: ns (blue circle): *p*-value > 5 × 10^−2^; * (red full circle): *p*-value < 5 × 10^−2^; ** (green diamond): *p*-value < 5 × 10^−3^; *** (blue square): *p*-value < 5 × 10^−4^; **** (purple triangle): *p*-value < 5 × 10^−5^.

Accordingly, when assessing the influence of epoxide substitution, we compared cytotoxic activity within the four previously formed groups. Unsurprisingly, these four tests returned significant *p*-values < 0.03 which led us to further investigate these differences by pairwise comparisons between all substituents using Dunn’s test (Figure 6).

Among the *trans* isomers of the TDAE series, compounds **2a**, **5a**, **6a**, **7a**, and **11a** displayed the highest activity. From these, halogenated benzene substituents (**5a**, **6a**, **7a**) displayed similar activities. Interestingly, fluorine in *meta* position (**8a**) differed significantly from them, with poorer cytotoxic activity. Unsubstituted benzene (**2a**) comparisons with the majority of the other chemical groups were inconclusive probably because of its important IC_50_ standard deviation. It only showed significant differences with *p*-nitrobenzene (**10a**) and ester substituents (**12a**, **13**), making them bad drug candidates; but also, with 5-nitrofuran (**11a**) which is a significantly better substituent. In the end, IC_50_ of 5-nitrofuran (**11a**), fluorine in *para* (**7a**) and chlorine in *ortho* (**6a**) substituents were not significantly different from each other, making them the best substituents in this group.

Among the *cis* isomers of the TDAE series, only the 5-nitrofuran substituent (**11b**) stood out from the others as the best one. Interestingly, in both *cis* and *trans* isomers, the introduction of the *p*-nitrobenzene ring (**10a**, **10b**), as an analog of 5-nitrofuran (**11a**, **11b**) on the epoxide ring, led to an almost complete loss of activity. This suggests that the antiproliferative activity of these compounds is not the result of the nitro group which is often associated with cytotoxic implications.

Among the *trans* isomers of the Sonogashira series, 3 substituents stood out as the most active ones with no difference between them: unsubstituted benzene (**14a**), *m*-fluorobenzene (**20a**), and 5-nitrofuran substituent (**23a**). There is also no significant difference between these isomers and the ester derivative **25** which also has good activity. Comparisons to *p*-methylbenzene (**15a**) are not very conclusive when categorizing it in this good activity compounds group since it did not show a significant difference with *p*-nitrobenzene (**22a**) and *p*-fluorobenzene (**19a**), compounds that did not display any activity on SK-N-SH cell line. Interestingly, the *trans* isomer with the ester substituent **24a** is significantly less active than its analog **25**. This difference could be explained by higher lipophilicity of **25** than **24a**.

Lastly, *m*-fluorobenzene (**20b**) and ester (**25**) appear the best compounds from the *cis* isomers of the Sonogashira series comparison group. Unsubstituted benzene (**14b**) and 5-nitrofuran (**23b**) substituents are significantly less active than the carboxylate **25**, but still might be grouped as active compounds with IC_50_ < 30 μM.

### 2.3. In Silico Evaluation

#### 2.3.1. Molecular Docking

To predict the molecular mechanism involved in the antiproliferative activity of these molecules, molecular docking of best compounds **11a** and **11b** from the TDAE series, and best compounds **14a** and **25** from the Sonogashira series, was performed on both crystallographic structures of human Topoisomerase IIβ (3QX3) and human Tissue Transglutaminase (4PYG) obtained from the Protein Data Bank. We selected these drug representatives because they have the lowest IC_50_ of the TDAE and Sonogashira series, respectively. Molecular docking was also performed on 3QX3 for compound XK-469, and on 4PYG for compound GK13 as references on each protein, respectively. Since no crystallized structure of topoisomerase complexed with XK-469 is available, the 3QX3 entry was chosen for Topoisomerase IIβ because of its complexation with a well-known topoisomerase II inhibitor, etoposide [31]. Similarly, since no crystallized structure of human tissue transglutaminase combined with GK13 was found, the 4PYG entry was chosen for this protein because of its complexation with guanosine triphosphate (GTP) since this enzyme notably possesses a GTPase enzymatic [32]. To perform molecular docking, affinities between compounds and protein targets were calculated by a “blind-docking approach” without “a priori” binding site information, for all our docking simulations [33,34]. We evaluated the binding modes of these compounds to each protein and calculated the binding affinities using the open-source program AutoDock Vina [35,36] which uses a scoring function relying on the Broyden-Fletcher-Goldfarb-Shanno algorithm for the local optimization. The quality of protein-ligand interactions was visually examined from the resulting conformation binding mode of the lowest level of Gibbs free energy of binding (ΔG).

**Figure 7:**
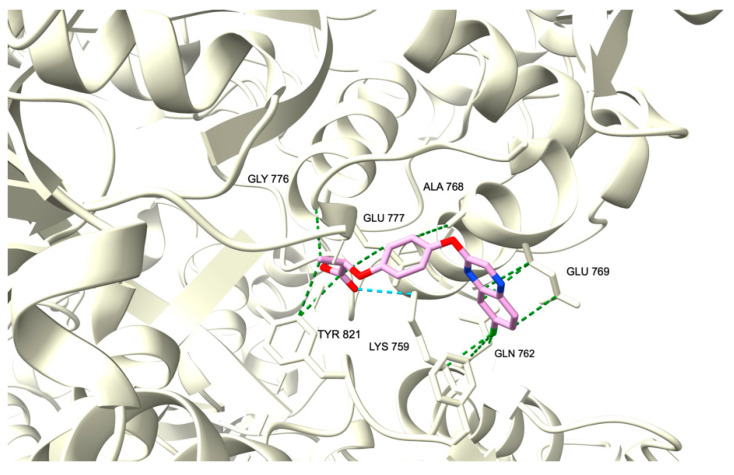

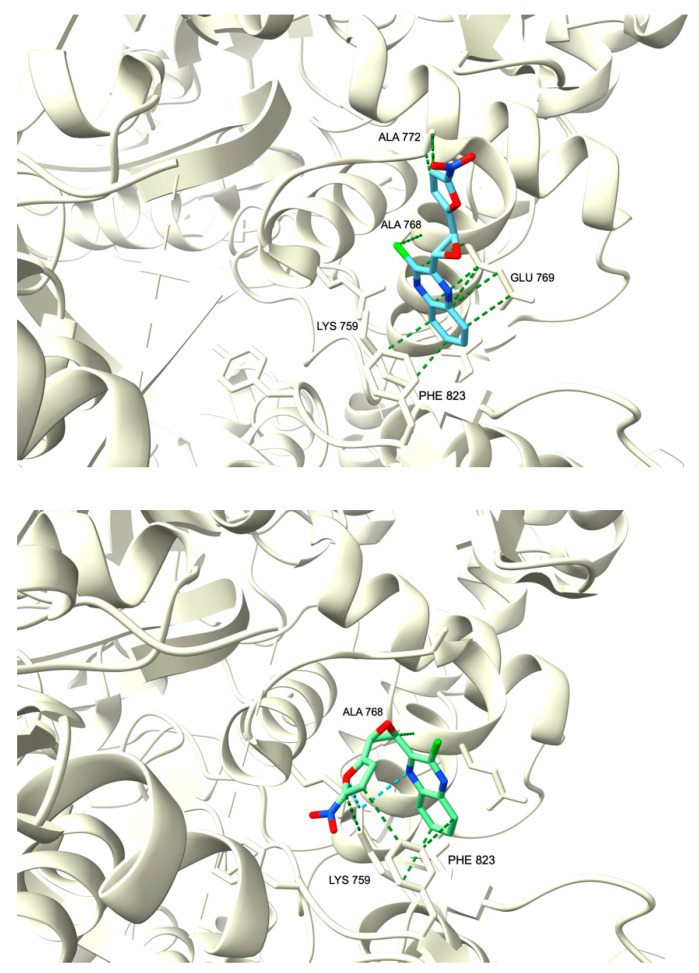
Ribbon representation of the binding modes of compounds **11a**, **11b**, and XK-469 to human topoisomerase II β. The protein is represented as light yellow ribbons. Compound XK-469 is drawn as light purple sticks while compound **11a** is sky blue and compound **11b** is light green.

On topoisomerase, this docking experiment revealed a very similar site of binding, close to the catalytic site of DNA cleavage, for all our compounds and XK-469 (Figure 7). This binding to the 3QX3 protein appears to be with high energy (Table 2), mostly by hydrophobic interactions. In particular, XK-469 interacts with 3QX3 primarily through Van Der Waals interaction with Tyr821 which has been described as an essential residue for the physiological activity of this enzyme [37]. All compounds interact with Lys759 which belongs to the winged helix domain (WHD) of the protein containing the catalytic tyrosine. Compound **11a** also displays Glu769 and Ala768 in common with **14a** and XK-469. Likewise, compound **11b** displays Ala768 in common with our drug reference. Representatives from the Sonogashira series **14a** and **25** both interact in the same way with His774 and Phe823, which are also amino acids interacting with **11b**. In particular, compound **25** has its interaction with Gln762 in common with XK-469. All these amino acids belong to the WHD domain of the Topoisomerase IIβ which could be an explanation for the mechanism of action of these compounds. As all our compounds interact, mainly through the same residues from the same domain as the proven antitopoisomerase XK-469, we strongly suggest that they share the same mechanism of action.

**Figure 8:**
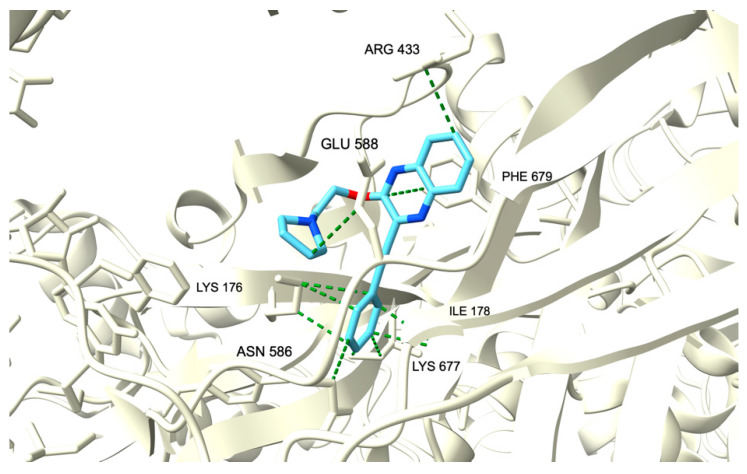

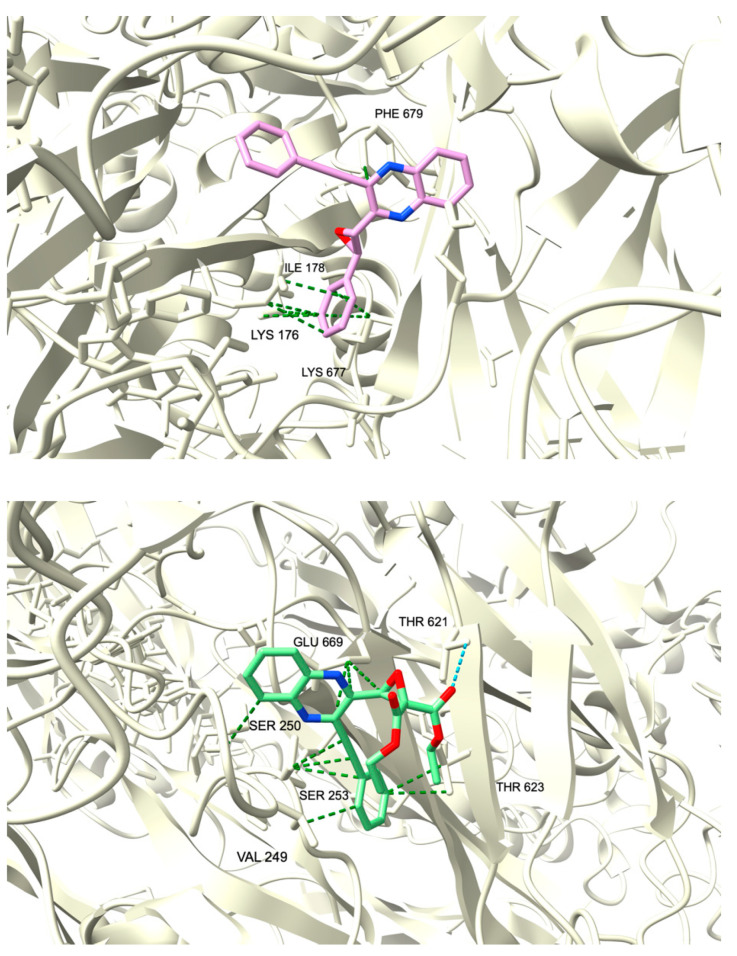
Ribbon representation of the binding modes of compounds **14a**, **25,** and GK-13 to human tissue transglutaminase. The protein is represented as light yellow ribbons. Compound GK-13 is drawn as sky blue sticks while compound **14a** is light purple and compound **25** is light green.

Regarding the molecular docking of these compounds on human tissue transglutaminase, all ligands but **25** interact with the protein in the same binding site as the reference inhibitor GK13. The binding energies were also in the same range as our reference (Table 3). The amino acids involved in this interaction are Lys176, Ile178, Arg433, Asn586, Glu588, Lys677, and Phe679, all by hydrophobic linking for compound GK13. (Figure 8). These amino acids belonging to the catalytic core of 4PYG could explain the inhibitory effect of GK13. Compound **14a** shares Lys176, Lys677, and Phe679 with GK13 in its interaction with the protein, also through Van der Waals linking. Surprisingly, compounds **11a** and **11b** from the TDAE series, which do not have the arylethynyl moiety, were also able to bind the protein domain like our reference. As for compound **25**, molecular docking revealed that it does not share the same protein binding domain as all other compounds. Indeed, this derivative from the Sonogashira series interacts with the protein through Val249, Ser250, Ser253, Thr621, Thr623, and Glu669.

#### 2.3.2. ADMET Predictions

Pharmacokinetics (PK) parameters are essential in the selection process of hit compounds in a hit-to-lead approach. Thus, we evaluated in silico these features with Simulations Plus software, ADMET Predictor^®,^ and GastroPlus^®^. The results of this evaluation did not reveal any differences between *cis* and *trans* diastereoisomers since the software does not consider conformation during its calculations. Therefore, they are presented as one molecule in this section and not as **a** and **b** compounds.

None of these compounds violate Lipinski’s “Rule of Five”, also known as Pfizer’s rule of five, for potential drug candidates (Table 3). These rules allow us to evaluate the drug likeliness of our compounds. They state that most orally active drugs with good bioavailability have no more than one violation of its four criteria: molecular weight (MW) ≥ 500 Da, limited lipophilicity expressed as logP ≥ 5, number of hydrogen bond acceptors ≥ 10, and number of hydrogen bond donors (H-BD) ≥ 5. In addition, simulations of logD at a physiological pH of 7.4 returned identical values to logP, as presented in Table 3. These parameters were calculated with Simulations Plus software ADMET Predictor^®^.

During the absorption phase, this modeling revealed that compounds from the TDAE series result in a predicted bioavailability (F) ranging from 82.71% for compounds **13** to 32.69% for compounds **4**. The hit compounds **11** returned a bioavailability value of 35.48% from this simulation. For aromatic substituted epoxide derivatives from the Sonogashira series, F values were ranging from 12.56% for compounds **14** to 1.535 for compounds **21**. In this series, only the non-aromatic substituted epoxide derivatives displayed high bioavailability with 90.05% for compounds **24** and 89.24% for compound **25**. This loss of absorption between TDAE and Sonogashira series derivatives can be explained by the increase of drug lipophilicity when adding the arylethynyl moiety. 

During the distribution phase, all our derivatives are susceptible to being bound by proteins in the plasma such as albumin because of their lipophilicity. Percentages of unbound drug to blood plasma proteins were less than 10%, except for compounds **11**, **12,** and **13**, which returned 15%, 22%, and 20%, respectively. These percentages range from 71% for compound **14** to 99% for our hit compound **11**. Furthermore, corroborating our in vitro results, none of our compounds are susceptible to being substrates of the P-gp except for compound **25**. Interestingly, this compound is susceptible with 97% accuracy to being a P-gp inhibitor. Similarly, this modeling suggests that all compounds in the Sonogashira series are susceptible to be P-gp inhibitors.

**Figure 9:**
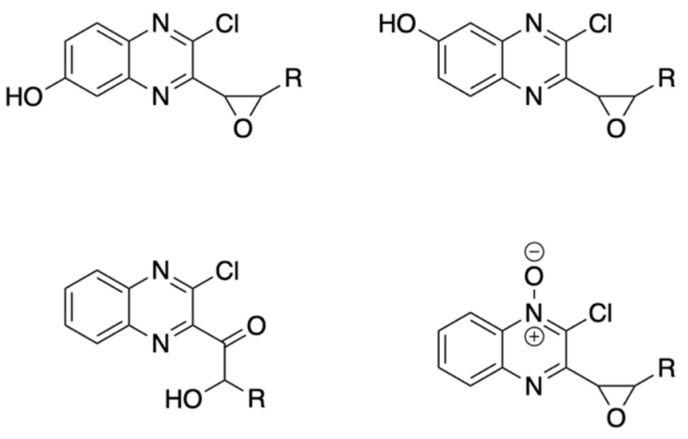
Possible metabolites of quinoxalines from the TDAE series generated by CYP3A4 metabolism.

According to our compounds’ metabolism phase prediction, all derivatives are susceptible to being substrates of the superfamily of cytochromes P450 (CYPs). This metabolism may result in various metabolites with hydroxylation of the quinoxaline core or even the oxirane ring with its opening (Figure 9).

AUC values predicted were ranging from 3819.6 ng-h/mL (**8**) to 24000 ng-h/mL (**10**) for compounds from the TDAE series, and from 495.3 ng-h/mL (**21**) to 180000 ng-h/mL (**14**) for compounds from the Sonogashira series.

## 3. Materials and Methods

### 3.1. Chemistry

#### 3.1.1. Generality

Melting points were determined on a Büchi melting point apparatus (BUCHI Corporation, New Castle, United States) and were uncorrected. High-resolution mass spectrometry analyses were carried out at the Spectropôle, Faculté des Sciences de Saint-Jérôme (Marseille, France) with a mass spectrometer SYNAPT G2 HDMS Waters (Milford, MA, United States) equipped with an electrospray ionization source (electrospray tension: 2.8 kV; orifice tension: 20 V; nebulization gas flow (nitrogen): 100 L/h). Samples were dissolved in 300 μL of dichloromethane, diluted at 1/102 in methanol solution at 0.1 mM sodium chloride, and introduced into the ionization source at 10 μL/min. High-resolution mass spectra were obtained with a time-of-flight (TOF) analyzer. Exact mass measurements were repeated in triplicate with external calibration. NMR spectra were recorded on a Bruker Avance NEO 400 MHz NanoBay spectrometer at the Faculté de Pharmacie of Marseille. (1H-NMR: reference CDCl_3_ δ = 7.26 ppm, reference DMSO-d6 δ = 2.50 ppm and 13C-NMR: reference CDCl_3_ δ = 76.9 ppm, reference DMSO-d6 δ = 39.52 ppm). They are presented in Supplementary Materials. TLC was performed on 5 cm × 10 cm aluminum plates coated with silica gel 60F-254 (Merck) in an appropriate eluent. Visualization was performed with ultraviolet light (234 nm). Reagents were purchased and used without further purifications from Sigma-Aldrich or Fluorochem. Ultra-High Performance Liquid Chromatography (UHPLC) analyses were performed using an Agilent 1290 Series apparatus (binary pump G4220A, autosampler G1330B, column oven G1316C, photodiode array detector G4212A). The system was piloted by OpenLAB CDS ChemStation Edition C.01.07 computer software. The chromatographic separation was achieved using a Zorbax Eclipse Plus C18 column 50 × 2.1 mm, 1.8 μm Agilent (Santa Clara, CA, United States) protected by a Zorbax Eclipse Plus C18 (5 × 2.1 mm, 1.8 μm) guard column. Water acidified with 0.1% of formic acid (*v/v*) as Solvent A and Acetonitrile acidified with 0.1% of formic acid (*v/v*) as Solvent B was used for the gradient elution at 0.5 mL.min^−1^. The gradient program was: 5% B (from 0 to 2.0 min), 5% to 100% B (from 2.0 min to 10.0 min), 100% B (from 10.0 min to 13.0 min with post time of 2.0 min. UV detection wavelength set at 254 nm and injection volume of 1.0 µL.

#### 3.1.2. General Procedure for Compounds **2** to **13**

To 2-chloro-3-(dibromomethyl)quinoxaline (**1**) (1 g, 2.97 mmol), appropriate carbonyl derivative (5.94 mmol, 2 eq.) in THF (20 mL) was added in a two-necked flask under inert gas. The reaction mixture was stirred for 1 h at −25 °C and 2 h at room temperature. Then, the mixture was extracted in ethyl acetate (3 × 40 mL) and washed with H_2_O (3 × 40 mL) before being dried with sodium sulfate. Each diastereoisomer was then purified by flash chromatography puriFlash^®^ using an IR-50SI-F0080 regular silica column and a dichloromethane/cyclohexane gradient (40:60 to 60:40).

2-chloro-3-(3-phenyloxiran-2-yl)quinoxaline (**2**)

*trans* isomer **2a**: yield: 43%, white solid, mp = 133 °C. 1H-NMR (400 MHz, CDCl_3_): δ (ppm) = 4.41 (d, J = 1.76 Hz, 1H), 4.59 (d, J = 1.84 Hz, 1H), 7.38–7.47 (m, 5H), 7.78–7.80 (m, 2H), 8.00–8.03 (m, 1H), 8.16–8.19 (m, 1H). 13C-NMR (101 MHz, CDCl_3_): δ (ppm) = 58.88, 61.32, 125.97 (2C), 128.26, 128.73 (2C), 128.89, 129.31, 130.70, 131.31, 135.95, 140.98, 141.57, 146.38, 148.80. HRMS-ESI: *m/z* calcd for C16H11ClN2O [M+Na]+: 305.0452; Found: 305.0450.

*cis* isomer **2b**: yield: 42%, yellow solid, mp = 103 °C. 1H-NMR (400 MHz, CDCl_3_): δ (ppm) = 4.62 (d, J = 4.32 Hz, 1H), 4.73 (d, J = 4.36 Hz, 1H), 7.06 (q, J = 5.74 Hz, 3H), 7.29 (q, J = 3.04 Hz, 2H), 7.68–7.76 (m, 2H), 7.88 (q, J = 3.15 Hz, 1H), 8.19 (q, J = 3.12 Hz, 1H). 13C-NMR (101 MHz, CDCl_3_): δ (ppm) = 58.87, 59.31, 126.61 (2C), 127.84 (2C), 128.14 (2C), 129.21, 130.46, 130.98, 132.86, 140.34, 141.12, 145.68, 147.67. HRMS-ESI: *m/z* calcd for C16H11ClN2O [M+Na]+: 305.0452; Found: 305.0449.

2-chloro-3-[3-(*p*-tolyl)oxiran-2-yl]quinoxaline (**3**)

*trans* isomer **3a**: yield: 31%, yellow solid, mp = 143 °C. 1H-NMR (400 MHz, CDCl_3_): δ (ppm) = 2.38 (s, 3H), 4.36 (d, J = 1.76 Hz, 1H), 4.59 (d, J = 1.84 Hz, 1H), 7.23 (d, J = 7.92 Hz, 2H), 7.35 (d, J = 8.08 Hz, 2H), 7.78–7.80 (m, 2H), 8.01–8.03 (m, 1H), 8.17–8.19 (m, 1H). ^13^C- NMR (101 MHz, CDCl_3_): δ (ppm) = 21.32, 58.81, 61.45, 125.94 (2C), 128.25, 129.31, 129.42 (2C), 130.67, 131.25, 132.95, 138.83, 141.00, 141.55, 146.38, 148.95. HRMS-ESI: *m/z* calcd for C17H13ClN2O [M+Na]+: 319.0609; Found: 319.0606.

2-chloro-3-{3-[4-(trifluoromethyl)phenyl]oxiran-2-yl}quinoxaline (**4**)

*trans* isomer **4a**: yield: 49%, white solid, mp = 154 °C. 1H-NMR (400 MHz, CDCl_3_): δ (ppm) = 4.49 (d, J = 1.60 Hz, 1H), 4.56 (d, J = 1.80 Hz, 1H), 7.58 (d, J = 8.20 Hz, 2H), 7.68 (d, J = 8.20 Hz, 2H), 7.79–7.82 (m, 2H), 8.01–8.04 (m, 1H), 8.14–8.18 (m, 1H). 13C-NMR (101 MHz, CDCl_3_): δ (ppm) = 59.06, 60.29, 123.97 (q, J = 272.12 Hz), 125.74 (q, J = 3.69 Hz, 2C), 126.25, 128.30, 129.29, 130.82, 131.00 (q, J = 32.46 Hz, 2C), 131.53, 140.01, 140.93, 141.67, 146.30, 148.14. HRMS-ESI: *m/z* calcd for C17H10ClF3N2O [M+H]+: 351.0507; Found: 351.0504.

*cis* isomer **4b**: yield: 48%, yellow solid, mp = 109 °C. 1H-NMR (400 MHz, CDCl_3_): δ (ppm) = 4.66 (d, J = 4.40 Hz, 1H), 4.78 (d, J = 4.40 Hz, 1H), 7.35 (d, J = 8.28 Hz, 2H), 7.45 (d, J = 8.52 Hz, 2H), 7.71–7.79 (m, 2H), 7.89–7.92 (m, 1H), 8.15–8.18 (m, 1H). 13C-NMR (101 MHz, CDCl_3_): δ (ppm) = 58.73, 58.83, 123.79 (q, J = 272.24 Hz), 124.86 (q, J = 3.79 Hz, 2C), 127.06, 128.21, 129.15, 130.30 (q, J = 32.49 Hz, 2C), 130.70, 131.29, 137.02, 140.27, 141.24, 145.49, 146.91. HRMS-ESI: *m/z* calcd for C17H10ClF3N2O [M+H]+: 351.0507; Found: 351.0503.

2-chloro-3-[3-(4-chlorophenyl)oxiran-2-yl]quinoxaline (**5**)

*trans* isomer **5a**: yield: 32%, white solid, mp = 152 °C. 1H-NMR (400 MHz, CDCl_3_): δ (ppm) = 4.39 (d, J = 1.80 Hz, 1H), 4.55 (d, J = 1.80 Hz, 1H), 7.40 (s, 4H), 7.81 (q, J = 3.28 Hz, 2H), 8.02–8.05 (m, 1H), 8.15–8.19 (m, 1H). 13C-NMR (101 MHz, CDCl_3_): δ (ppm) = 58.93, 60.55, 127.29 (2C), 128.27, 128.97 (2C), 129.27, 130.76, 131.41, 134.50, 134.74, 140.92, 141.60, 146.31, 148.41. HRMS-ESI: *m/z* calcd for C16H10Cl2N2O [M+Na]+: 339.0062; Found: 339.0059.

*cis* isomer **5b**: yield: 31%, yellow solid, mp = 113 °C. 1H-NMR (400 MHz, CDCl_3_): δ (ppm) = 4.58 (d, J = 4.36 Hz, 1H), 4.73 (d, J = 4.32 Hz, 1H), 7.05 (q, J = 2.84 Hz, 2H), 7.24 (d, J = 8.48 Hz, 2H), 7.73–7.78 (m, 2H), 7.90–7.92 (m, 1H), 8.16–8.18 (m, 1H). 13C-NMR (101 MHz, CDCl_3_): δ (ppm) = 58.72, 58.81, 127.99 (2C), 128.13 (2C), 128.22, 129.16, 130.63, 131.18, 131.45, 134.07, 140.29, 141.20, 145.55, 147.24. HRMS-ESI: *m/z* calcd for C16H10Cl2N2O [M+Na]^+^: 339.0062; Found: 339.0062.

2-chloro-3-[3-(2-chlorophenyl)oxiran-2-yl]quinoxaline (**6**)

*trans* isomer **6a**: yield: 42%, white solid, mp = 136 °C. 1H-NMR (400 MHz, CDCl_3_): δ (ppm) = 4.47 (d, J = 1.88 Hz, 1H), 4.70 (d, J = 1.76 Hz, 1H), 7.27–7.35 (m, 2H), 7.39 (q, J = 3.05 Hz, 1H), 7.47 (q, J = 3.11 Hz, 1H), 7.79 (q, J = 3.29 Hz, 2H), 8.00–8.03 (m, 1H), 8.16–8.19 (m, 1H). 13C-NMR (101 MHz, CDCl_3_): δ (ppm) = 58.28, 58.95, 126.25, 127.21, 128.26, 129.32, 129.40, 129.61, 130.68, 131.37, 133.47, 134.11, 141.00, 141.67, 146.32, 148.37. HRMS-ESI: *m/z* calcd for C16H10Cl2N2O [M+Na]+: 339.0062; Found: 339.0062.

2-chloro-3-[3-(4-fluorophenyl)oxiran-2-yl]quinoxaline (**7**)

*trans* isomer **7a**: yield: 32%, white solid, mp = 136 °C. 1H-NMR (400 MHz, CDCl_3_): δ (ppm) = 4.40 (d, J = 1.28 Hz, 1H), 4.56 (d, J = 1.28 Hz, 1H), 7.12 (t, J = 8.58 Hz, 2H), 7.44 (q, J = 4.60 Hz, 2H), 7.79–7.84 (m, 2H), 8.02–0.06 (m, 1H), 8.17–9.21 (m, 1H). 13C-NMR (101 MHz, CDCl_3_): δ (ppm) = 59.00, 60.81, 115.93 (d, J = 21.83 Hz, 2C), 127.85 (d, J = 8.48 Hz, 2C), 128.42, 129.43, 130.88, 131.52, 131.85 (d, J = 2.93 Hz), 141.11, 141.75, 146.46, 148.68, 163.25 (d, J = 247.52 Hz). HRMS-ESI: *m/z* calcd for C16H10ClFN2O [M+Na]+: 323.0358; Found: 323.0351.

*cis* isomer **7b**: yield: 36%, yellow solid, mp = 98 °C. 1H-NMR (400 MHz, CDCl_3_): δ (ppm) = 4.60 (d, J = 4.32 Hz, 1H), 4.72 (d, J = 4.32 Hz, 1H), 6.77 (q, J = 5.80 Hz, 2H), 7.27– 7.29 (m, 2H), 7.71–7.78 (m, 2H), 7.90–7.92 (m, 1H), 8.16–8.18 (m, 1H). 13C-NMR (101 MHz, CDCl_3_): δ (ppm) = 58.83, 58.87, 115.03 (d, J = 21.78 Hz, 2C), 128.28, 128.45 (d, J = 8.21 Hz, 2C), 128.73 (d, J = 2.93 Hz), 129.25, 130.68, 131.21, 140.40, 141.25, 145.69, 147.51, 162.59 (d, J = 247.00 Hz). HRMS-ESI: *m/z* calcd for C16H10ClFN2O [M+Na]+: 323.0358; Found: 323.0358.

2-chloro-3-[3-(3-fluorophenyl)oxiran-2-yl]quinoxaline (**8**)

*trans* isomer **8a**: yield: 37%, yellow solid, mp = 132 °C. 1H-NMR (400 MHz, CDCl_3_): δ (ppm) = 4.40 (d, J = 1.72 Hz, 1H), 4.56 (d, J = 1.80 Hz, 1H), 7.12 (t, J = 8.66 Hz, 1H), 7.42– 7.46 (m, 2H), 7.80–7.83 (m, 3H), 8.02–8.06 (m, 1H), 8.16–8.20 (m, 1H). 13C-NMR (101 MHz, CDCl_3_): δ (ppm) = 58.94, 60.44 (d, J = 2.23 Hz), 112.75 (d, J = 22.61 Hz), 115.86 (d, J = 21.35 Hz), 121.76 (d, J = 2.89 Hz), 128.28, 129.30, 130.39 (d, J = 8.22 Hz), 130.77, 131.44, 138.64 (d, J = 7.48 Hz), 140.94, 141.64, 146.33, 148.34, 163.15 (d, J = 246.88 Hz). HRMS-ESI: *m/z* calcd for C16H10ClFN2O [M+Na]+: 323.0358; Found: 323.0361.

*cis* isomer **8b**: yield: 38%, brown solid, mp = 128 °C. 1H-NMR (400 MHz, CDCl_3_): δ (ppm) = 4.61 (d, J = 4.36 Hz, 1H), 4.75 (d, J = 4.37 Hz, 1H), 6.72–6.78 (m, 1H), 7.02–7.10 (m, 3H), 7.71–7.78 (m, 2H), 7.89–7.92 (m, 1H), 8.17–8.20 (m, 1H). 13C-NMR (101 MHz, CDCl_3_): δ (ppm) = 58.71 (d, J = 2.17 Hz), 58.74, 113.83 (d, J = 23.20 Hz), 115.19 (d, J = 21.24 Hz), 122.38 (d, J = 2.91 Hz), 128.15, 129.22, 129.51 (d, J = 8.20 Hz), 130.62, 131.17, 135.54 (d, J = 7.86 Hz), 140.30, 141.18, 145.57, 147.16, 162.31 (d, J = 246.28 Hz). HRMS-ESI: *m/z* calcd for C16H10ClFN2O [M+Na]+: 323.0358; Found: 323.0357.

4-[3-(3-chloroquinoxalin-2-yl)oxiran-2-yl]benzonitrile (**9**)

*trans* isomer **9a**: yield: 44%, white solid, mp = 162 °C. 1H-NMR (400 MHz, CDCl_3_): δ (ppm) = 4.51 (d, J = 1.71 Hz, 1H), 4.55 (d, J = 1.79 Hz, 1H), 7.58 (t, J = 4.13 Hz, 2H), 7.73 (q, J = 2.79 Hz, 2H), 7.82–7.85 (m, 2H), 8.05–8.07 (m, 1H), 8.17–8.19 (m, 1H). 13C-NMR (101 MHz, CDCl_3_): δ (ppm) = 59.22, 60.04, 112.70, 118.47, 126.59 (3C), 128.35, 129.31, 130.92, 131.67, 132.61 (2C), 140.94, 141.30, 141.76, 147.82. HRMS-ESI: *m/z* calcd for C17H10ClN3O [M+Na]+: 330.0405; Found: 330.0403.

*cis* isomer **9b**: yield: 38%, yellow solid, mp = 103 °C. 1H-NMR (400 MHz, CDCl_3_): δ (ppm) = 4.66 (d, J = 4.40 Hz, 1H), 4.81 (d, J = 4.36 Hz, 1H), 7.39–7.47 (m, 4H), 7.77–7.80 (m, 2H), 7.92–7.95 (m, 1H), 8.14–8.17 (m, 1H). 13C-NMR (101 MHz, CDCl_3_): δ (ppm) = 58.56, 58.76, 112.12, 118.30, 127.44 (2C), 128.28, 129.12, 130.85, 131.47, 131.68 (2C), 138.35, 140.22, 141.28, 145.42, 146.60. HRMS-ESI: *m/z* calcd for C17H10ClN3O [M+Na]+: 330.0405; Found: 330.0404.

2-chloro-3-[3-(4-nitrophenyl)oxiran-2-yl]quinoxaline (**10**)

*trans* isomer **10a**: yield: 37%, yellow solid, mp = 212 °C. 1H-NMR (400 MHz, CDCl_3_): δ (ppm) = 4.58 (d, J = 1.78 Hz, 2H), 7.65 (q, J = 2.91 Hz, 2H), 7.83–7.85 (m, 2H), 8.05–8.08 (m, 1H), 8.18–8.20 (m, 1H), 8.30 (q, J = 2.92 Hz, 2H). 13C-NMR (101 MHz, CDCl_3_): δ (ppm) = 59.30, 59.82, 124.08 (2C), 126.75 (2C), 128.36, 129.32, 130.94, 131.72, 140.94, 141.79, 143.21, 146.29, 147.71, 148.27. HRMS-ESI: *m/z* calcd for C16H10ClN3O3 [M+Na]+: 350.0303; Found: 350.0298.

*cis* isomer **10b**: yield: 21%, yellow solid, mp = 220 °C. 1H-NMR (400 MHz, CDCl_3_): δ (ppm) = 4.71 (d, J = 4.39 Hz, 1H), 4.84 (d, J = 4.40 Hz, 1H), 7.53 (q, J = 2.88 Hz, 2H), 7.76–7.81 (m, 2H), 7.91–7.94 (m, 1H), 7.97 (q, J = 2.95 Hz, 2H), 8.15–8.18 (m, 1H). 13C-NMR (101 MHz, CDCl_3_): δ (ppm) = 58.44, 58.80, 123.13 (2C), 127.66 (2C), 128.28, 129.14, 130.90, 131.51, 140.21, 140.30, 141.30, 145.38, 146.48, 147.75. HRMS-ESI: *m/z* calcd for C16H10ClN3O3 [M+Na]+: 350.0303; Found: 350.0298.

2-chloro-3-[3-(5-nitrofuran-2-yl)oxiran-2-yl]quinoxaline (**11**)

*trans* isomer **11a**: yield: 37%, orange solid, mp = 141 °C. 1H-NMR (400 MHz, CDCl_3_): δ (ppm) = 4.64 (d, J = 1.81 Hz, 1H), 5.04 (d, J = 1.83 Hz, 1H), 6.80 (d, J = 3.70 Hz, 1H), 7.36 (d, J = 3.69 Hz, 1H), 7.83–7.86 (m, 2H), 8.05–8.07 (m, 1H), 8.13–8.16 (m, 1H). 13C-NMR (101 MHz, CDCl_3_): δ (ppm) = 52.82, 58.30, 111.83, 112.68, 128.37, 129.21, 130.99, 131.77 (2C), 140.30, 141.62, 145.67, 146.03, 150.84. HRMS-ESI: *m/z* calcd for C14H8ClN3O4 [M+Na]+: 340.0096; Found: 340.0087.

*cis* isomer **11b**: yield: 27%, brown solid, mp = 157 °C. 1H-NMR (400 MHz, CDCl_3_): δ (ppm) = 4.62 (d, J = 3.96 Hz, 1H), 4.91 (d, J = 4.00 Hz, 1H), 6.38 (d, J = 3.72 Hz, 1H), 7.01 (d, J = 3.72 Hz, 1H), 7.82–7.86 (m, 2H), 8.03 (q, J = 3.24 Hz, 1H), 8.20 (q, J = 3.26 Hz, 1H). 13C-NMR (101 MHz, CDCl_3_): δ (ppm) = 53.34, 56.92, 112.35, 112.71, 128.36, 129.26, 131.01 (2C), 131.95, 140.81, 141.89, 146.40, 146.79, 152.46. HRMS-ESI: *m/z* calcd for C14H8ClN3O4 [M+Na]+: 340.0096; Found: 340.0092.

ethyl 3-(3-chloroquinoxalin-2-yl)oxirane-2-carboxylate (**12**)

*trans* isomer **12a**: yield: 48%, red solid, mp = 112 °C. 1H-NMR (400 MHz, CDCl_3_): δ (ppm) = 1.35 (t, J = 7.16 Hz, 3H), 4.13 (d, J = 1.76 Hz, 1H), 4.30–4.37 (m, 2H), 4.78 (d, J = 1.72 Hz, 1H), 7.77–7.81 (m, 2H), 7.99–0.02 (m, 1H), 8.07–8.10 (m, 1H). 13C-NMR (101 MHz, CDCl_3_): δ (ppm) = 14.14, 54.42, 54.71, 62.18, 128.27, 129.30, 130.86, 131.81, 140.76, 141.80, 146.47, 146.86, 167.57. HRMS-ESI: *m/z* calcd for C13H11ClN2O3 [M+Na]+: 301.0350; Found: 301.0352.

diethyl 3-(3-chloroquinoxalin-2-yl)oxirane-2,2-dicarboxylate (**13**)

yield: 67%, yellow solid, mp = 64 °C. 1H-NMR (400 MHz, CDCl_3_): δ (ppm) = 1.03 (t, J = 7.14 Hz, 3H), 1.36 (t, J = 7.14 Hz, 3H), 4.11 (q, J = 7.14 Hz, 2H), 4.35–4.41 (m, 2H), 5.04 (s, 1H), 7.75–7.83 (m, 2H), 8.00–8.02 (m, 1H), 8.07 (t, J = 4.82 Hz, 1H). 13C-NMR (101 MHz, CDCl_3_): δ (ppm) = 13.79, 13.99, 58.83, 62.02, 62.16, 63.30, 128.33, 129.25, 130.90, 131.90, 140.26, 141.70, 145.38, 146.19, 163.04, 164.77. HRMS-ESI: *m/z* calcd for C16H15ClN2O5 [M+Na]+: 373.0562; Found: 373.0552.

#### 3.1.3. General Procedure for Compounds **14** to **25**

To each quinoxaline of the previous series (1 eq., 100 mg), dichlorobis(triphenylphosphine)palladium(II) (0.05 eq.) and cuprous iodide (0.1 eq.) dissolved in acetonitrile in a two-necked flask, were added triethylamine (10 eq.) and phenylacetylene (1.5 eq.). The reaction mixture was stirred for 2h at room temperature. Then, the mixture was extracted in dichloromethane (3 × 40 mL) and washed with H_2_O (3 × 40 mL) before being dried with sodium sulfate. Each compound was then purified by flash chromatography puriFlash^®^ using an IR-50SI-F0040 regular silica column and a dichloromethane/cyclohexane gradient (40:60 to 60:40).

2-(phenylethynyl)-3-(3-phenyloxiran-2-yl)quinoxaline (**14**)

*trans* isomer **14a**: yield: 81%, brown solid, mp = 122 °C. 1H-NMR (400 MHz, CDCl_3_): δ (ppm) = 4.43 (d, J = 1.81 Hz, 1H), 4.77 (d, J = 1.87 Hz, 1H), 7.28–7.52 (m, 10H), 7.80 (q, J = 3.29 Hz, 2H), 8.10–8.13 (m, 1H), 8.16–8.20 (m, 1H). 13C-NMR (101 MHz, CDCl_3_): δ (ppm) = 60.37, 61.07, 85.53, 97.04, 121.03, 125.94 (2C), 128.52 (2C), 128.78 (3C), 128.93, 129.32, 129.91, 130.72, 130.91, 132.21 (2C), 136.46, 138.63, 140.60, 141.68, 151.54. HRMS-ESI: *m/z* calcd for C_24_H_16_N_2_O [M+Na]+: 371.1155; Found: 371.1147.

*cis* isomer **14b**: yield: 80%, orange solid, mp = 125 °C. 1H-NMR (400 MHz, CDCl_3_): δ (ppm) = 4.65 (d, J = 4.40 Hz, 1H), 4.93 (d, J = 4.40 Hz, 1H), 7.07 (q, J = 2.34 Hz, 3H), 7.29–7.31 (m, 2H), 7.47–7.50 (m, 3H), 7.70–7.75 (m, 4H), 7.98–8.00 (m, 1H), 8.15–8.18 (m, 1H). 13C-NMR (101 MHz, CDCl_3_): δ (ppm) = 59.24, 59.38, 85.37, 96.80, 121.35, 126.66 (2C), 127.80 (2C), 128.02, 128.77 (2C), 128.84, 129.22, 130.05, 130.45, 130.69, 132.23 (2C), 133.24, 138.08, 140.02, 141.17, 150.28. HRMS-ESI: *m/z* calcd for C_24_H_16_N_2_O [M+Na]+: 371.1155; Found: 371.1147.

2-(phenylethynyl)-3-[3-(*p*-tolyl)oxiran-2-yl]quinoxaline (**15**)

*trans* isomer **15a**: yield: 78%, orange solid, mp = 129 °C. 1H-NMR (400 MHz, CDCl_3_): δ (ppm) = 2.40 (s, 3H), 4.39 (d, J = 1.80 Hz, 1H), 4.76 (d, J = 1.89 Hz, 1H), 7.22–7.30 (m, 4H), 7.37 (q, J = 4.60 Hz, 5H), 7.77 (q, J = 3.29 Hz, 2H), 8.10 (q, J = 3.25 Hz, 1H), 8.15–8.17 (m, 1H). 13C-NMR (101 MHz, CDCl_3_): δ (ppm) = 21.32, 60.24, 61.19, 85.58, 96.96, 121.10, 125.94 (3C), 128.48 (2C), 128.94, 129.35, 129.44 (2C), 129.88, 130.66, 130.88, 132.25 (2C), 133.47, 138.66, 140.66, 141.68, 151.70. HRMS-ESI: *m/z* calcd for C25H18N2O [M+H]+: 363.1492; Found: 363.1488.

2-(phenylethynyl)-3-{3-[4-(trifluoromethyl)phenyl]oxiran-2-yl}quinoxaline (**16**)

*trans* isomer **16a**: yield: 86%, white solid, mp = 173 °C. 1H-NMR (400 MHz, CDCl_3_): δ (ppm) = 4.50 (d, J = 1.52 Hz, 1H), 4.72 (d, J = 1.80 Hz, 1H), 7.31 (t, J = 4.53 Hz, 4H), 7.38–7.42 (m, 1H), 7.61 (d, J = 8.18 Hz, 2H), 7.70 (d, J = 8.21 Hz, 2H), 7.80–7.82 (m, 2H), 8.11–8.14 (m, 1H), 8.15–8.19 (m, 1H). 13C-NMR (101 MHz, CDCl_3_): δ (ppm) = 60.08, 60.62, 85.33, 97.14, 120.91, 124.02 (q, J = 271.77 Hz), 125.79 (q, J = 3.75 Hz, 2C), 126.18 (2C), 128.57 (2C), 129.00, 129.36, 130.09, 130.95 (q, J = 32.46 Hz, 2C), 130.95, 131.06, 132.11 (2C), 138.61, 140.60, 141.82, 150.87. HRMS-ESI: *m/z* calcd for C25H15F3N2O [M+Na]+: 439.1029; Found: 439.1023.

*cis* isomer **16b**: yield: 83%, white solid, mp = 159 °C. 1H-NMR (400 MHz, CDCl_3_): δ (ppm) = 4.67 (d, J = 4.39 Hz, 1H), 4.98 (d, J = 4.41 Hz, 1H), 7.35 (d, J = 8.29 Hz, 2H), 7.43– 7.50 (m, 5H), 7.71–7.77 (m, 4H), 8.00–8.02 (m, 1H), 8.13–8.15 (m, 1H). 13C-NMR (101 MHz, CDCl_3_): δ (ppm) = 58.82, 59.19, 85.17, 97.01, 121.20, 123.84 (q, J = 272.41 Hz), 124.81 (q, J = 3.66 Hz, 2C), 127.09, 128.84 (2C), 128.92, 129.19, 130.20, 130.22 (q, J = 32.21 Hz, 2C), 130.77, 130.95, 132.23 (2C), 137.37, 137.96, 139.97, 141.30, 149.53. HRMS-ESI: *m/z* calcd for C25H15F3N2O [M+Na]+: 439.1029; Found: 439.1024.

2-[3-(4-chlorophenyl)oxiran-2-yl]-3-(phenylethynyl)quinoxaline (**17**)

*trans* isomer **17a**: yield: 75%, orange solid, mp = 150 °C. 1H-NMR (400 MHz, CDCl_3_): δ (ppm) = 4.41 (d, J = 1.84 Hz, 1H), 4.70 (d, J = 1.84 Hz, 1H), 7.30–7-40 (m, 9H), 7.77–7.79 (m, 2H), 8.09–8.12 (m, 1H), 8.13–8.16 (m, 1H). 13C-NMR (101 MHz, CDCl_3_): δ (ppm) = 60.35, 60.41, 85.44, 97.04, 120.98, 127.25 (2C), 128.57 (2C), 128.98, 129.01 (2C), 129.34, 130.03, 130.83, 130.98, 132.16 (2C), 134.63, 135.07, 138.61, 140.61, 141.75, 151.14. HRMS-ESI: *m/z* calcd for C24H15ClN2O [M+Na]+: 405.0765; Found: 405.0762.

*cis* isomer **17b**: yield: 58%, red solid, mp = 160 °C. 1H-NMR (400 MHz, CDCl_3_): δ (ppm) = 4.60 (d, J = 4.36 Hz, 1H), 4.92 (d, J = 4.36 Hz, 1H), 7.04 (q, J = 2.84 Hz, 2H), 7.24 (t, J = 4.22 Hz, 2H), 7.46 (t, J = 6.06 Hz, 3H), 7.68–7.77 (m, 4H), 7.98–8.01 (m, 1H), 8.13–8.15 (m, 1H). 13C-NMR (101 MHz, CDCl_3_): δ (ppm) = 58.80, 59.17, 85.26, 96.92, 121.24, 128.03 (2C), 128.07 (2C), 128.81 (2C), 128.91, 129.20, 130.14, 130.65, 130.86, 131.82, 132.22 (2C), 133.95, 137.99, 139.98, 141.25, 149.86. HRMS-ESI: *m/z* calcd for C24H15ClN2O [M+Na]+: 405.0765; Found: 405.0764.

2-[3-(2-chlorophenyl)oxiran-2-yl]-3-(phenylethynyl)quinoxaline (**18**)

*trans* isomer **18a**: yield: 58%, beige solid, mp = 163 °C. 1H-NMR (400 MHz, CDCl_3_): δ (ppm) = 4.65 (d, J = 1.88 Hz, 1H), 4.77 (d, J = 1.64 Hz, 1H), 7.28–7.44 (m, 8H), 7.53 (q, J = 3.04 Hz, 1H), 7.79 (q, J = 3.28 Hz, 2H), 8.10–8.13 (m, 1H), 8.16–8.20 (m, 1H). 13C-NMR (101 MHz, CDCl_3_): δ (ppm) = 58.77, 59.56, 85.45, 96.94, 121.18, 126.19, 127.26, 128.51 (2C), 128.97, 129.32, 129.46, 129.48, 129.88, 130.81, 130.93, 132.19 (2C), 133.55, 134.56, 138.64, 140.73, 141.83, 151.05. HRMS-ESI: *m/z* calcd for C24H15ClN2O [M+Na]+: 405.0765; Found: 405.0759.

2-[3-(4-fluorophenyl)oxiran-2-yl]-3-(phenylethynyl)quinoxaline (**19**)

*trans* isomer **19a**: yield: 74%, orange solid, mp = 155 °C. 1H-NMR (400 MHz, CDCl_3_): δ (ppm) = 4.42 (d, J = 1.80 Hz, 1H), 4.72 (d, J = 1.84 Hz, 1H), 7.09–7.14 (m, 2H), 7.30–7.40 (m, 5H), 7.44–7.48 (m, 2H), 7.79 (q, J = 3.28 Hz, 2H), 8.09–8.13 (m, 1H), 8.14–8.17 (m, 1H). 13C-NMR (101 MHz, CDCl_3_): δ (ppm) = 60.28, 60.46, 85.48, 96.95, 115.80 (d, J = 21.82 Hz, 2C), 121.02, 127.68 (d, J = 8.25 Hz, 2C), 128.57 (2C), 128.97, 129.34, 130.00, 130.80, 130.97, 132.16 (2C), 132.27 (d, J = 3.07 Hz), 138.61, 140.63, 141.74, 151.27, 163.09 (d, J = 247.59 Hz). HRMS-ESI: *m/z* calcd for C24H15FN2O [M+H]+: 367.1241; Found: 367.1234.

*cis* isomer **19b**: yield: 90%, brown solid, mp = 156 °C. 1H-NMR (400 MHz, CDCl_3_): δ (ppm) = 4.62 (d, J = 4.32 Hz, 1H), 4.91 (d, J = 4.36 Hz, 1H), 6.74–6.78 (m, 2H), 7.27–7.29 (m, 2H), 7.45–7.48 (m, 3H), 7.69–7.75 (m, 4H), 7.98–8.01 (m, 1H), 8.13–8.15 (m, 1H). 13C-NMR (101 MHz, CDCl_3_): δ (ppm) = 58.83, 59.12, 85.29, 96.86, 114.88 (d, J = 21.72 Hz, 2C), 121.28, 128.39 (d, J = 8.44 Hz, 2C), 128.80 (2C), 128.89, 128.99 (d, J = 3.00 Hz), 129.19, 130.12, 130.60, 130.82, 132.22 (2C), 138.04, 139.99, 141.22, 150.03, 162.46 (d, J = 246.74 Hz). HRMS-ESI: *m/z* calcd for C24H15FN2O [M+H]+: 367.1241; Found: 367.1236.

2-[3-(3-fluorophenyl)oxiran-2-yl]-3-(phenylethynyl)quinoxaline (**20**)

*trans* isomer **20a**: yield: 68%, orange solid, mp = 133 °C. 1H-NMR (400 MHz, CDCl_3_): δ (ppm) = 4.62 (d, J = 4.40 Hz, 1H), 4.94 (d, J = 4.40 Hz, 1H), 6.72–6.77 (m, 1H), 7.03–7.10 (m, 3H), 7.46–7.49 (m, 3H), 7.69–7.75 (m, 4H), 7.98–8.01 (m, 1H), 8.14–8.16 (m, 1H). 13C-NMR (101 MHz, CDCl_3_): δ (ppm) = 58.80 (d, J = 2.12 Hz), 59.10, 85.22, 96.91, 113.91 (d, J = 23.20 Hz), 115.08 (d, J = 21.15 Hz), 121.24, 122.40 (d, J = 2.92 Hz), 128.80 (2C), 128.85, 129.25, 129.43 (d, J = 8.16 Hz), 130.12, 130.64, 130.86, 132.23 (2C), 135.90 (d, J = 7.91 Hz), 138.01, 139.98, 141.24, 149.78, 162.31 (d, J = 246.08 Hz). HRMS-ESI: *m/z* calcd for C24H15FN2O [M+H]+: 367.1241; Found: 367.1233.

*cis* isomer **20b**: yield: 90%, brown solid, mp = 124 °C. 1H-NMR (400 MHz, CDCl_3_): δ (ppm) = 4.44 (d, J = 1.64 Hz, 1H), 4.72 (d, J = 1.80 Hz, 1H), 7.06–7.11 (m, 1H), 7.17–7.20 (m, 1H), 7.29–7.41 (m, 7H), 7.77–7.80 (m, 2H), 8.09–8.11 (m, 1H), 8.14–8.16 (m, 1H). 13C-NMR (101 MHz, CDCl_3_): δ (ppm) = 60.25 (d, J = 2.11 Hz), 60.30, 85.44, 97.00, 112.68 (d, J = 22.61 Hz), 115.71 (d, J = 21.38 Hz), 121.00, 121.78 (d, J = 2.88 Hz), 128.58 (2C), 128.97, 129.34, 130.00, 130.42 (d, J = 8.13 Hz), 130.85, 130.98, 132.18 (2C), 138.63, 139.20 (d, J = 7.36 Hz), 140.61, 141.77, 151.04, 163.22 (d, J = 246.93 Hz). HRMS-ESI: *m/z* calcd for C24H15FN2O [M+H]+: 367.1241; Found: 367.1233.

4-{3-[3-(phenylethynyl)quinoxalin-2-yl]oxiran-2-yl}benzonitrile (**21**)

*trans* isomer **21a**: yield: 91%, yellow solid, mp = 211 °C. 1H-NMR (400 MHz, DMSO): δ (ppm) = 4.57 (d, J = 1.76 Hz, 1H), 4.90 (d, J = 1.88 Hz, 1H), 7.34–7.42 (m, 4H), 7.49–7.53 (m, 1H), 7.74 (d, J = 8.32 Hz, 2H), 7.92–7.95 (m, 4H), 8.12–8.16 (m, 2H). 13C-NMR (101 MHz, DMSO): δ (ppm) = 59.37, 61.01, 86.04, 96.56, 111.78, 119.11, 120.69, 127.60 (2C), 129.12, 129.33 (3C), 130.89, 131.66, 131.99, 132.31 (2C), 133.11 (2C), 138.49, 140.22, 141.47, 142.61, 151.84. HRMS-ESI: *m/z* calcd for C25H15N3O [M+Na]+: 396.1107; Found: 396.1106.

*cis* isomer **21b**: yield: 86%, yellow solid, mp = 190 °C. 1H-NMR (400 MHz, DMSO): δ (ppm) = 4.99 (d, J = 4.58 Hz, 1H), 5.15 (d, J = 4.58 Hz, 1H), 7.40 (d, J = 8.40 Hz, 2H), 7.57–7.60 (m, 5H), 7.84–7.91 (m, 4H), 8.00 (q, J = 3.26 Hz, 1H), 8.12–8.15 (m, 1H). 13C-NMR (101 MHz, DMSO): δ (ppm) = 57.89, 59.72, 85.58, 96.83, 111.10, 118.81, 120.87, 127.76 (2C), 128.98, 129.30, 129.59 (2C), 131.04, 131.57, 131.87, 132.14 (2C), 132.78 (2C), 137.95, 139.67, 140.21, 140.96, 150.54. HRMS-ESI: *m/z* calcd for C25H15N3O [M+Na]+: 396.1107; Found: 396.1104.

2-[3-(4-nitrophenyl)oxiran-2-yl]-3-(phenylethynyl)quinoxaline (**22**)

*trans* isomer **22a**: yield: 74%, yellow solid, mp = 183 °C. 1H-NMR (400 MHz, CDCl_3_): δ (ppm) = 4.61 (d, J = 1.38 Hz, 1H), 4.74 (d, J = 1.58 Hz, 1H), 7.33 (t, J = 7.57 Hz, 2H), 7.42 (t, J = 7.14 Hz, 3H), 7.66 (d, J = 8.67 Hz, 2H), 7.81–7.83 (m, 2H), 8.12–8.18 (m, 2H), 8.29 (d, J = 8.64 Hz, 2H). 13C-NMR (101 MHz, CDCl_3_): δ (ppm) = 58.52, 59.16, 85.08, 97.14, 121.08, 123.06 (2C), 127.68 (2C), 128.89 (2C), 128.95, 129.15, 130.30, 130.96, 131.13, 132.24 (2C), 137.90, 139.87, 140.70, 141.34, 147.68, 149.09. HRMS-ESI: *m/z* calcd for C24H15N3O3 [M+Na]+: 416.1006; Found: 416.0997.

*cis* isomer **22b**: yield: 75%, yellow solid, mp = 210 °C. 1H-NMR (400 MHz, CDCl_3_): δ (ppm) = 4.71 (d, J = 4.40 Hz, 1H), 5.02 (d, J = 4.40 Hz, 1H), 7.50 (q, J = 5.68 Hz, 5H), 7.70–7.78 (m, 4H), 7.96 (d, J = 8.80 Hz, 2H), 7.99–8.01 (m, 1H), 8.10–8.13 (m, 1H). 13C-NMR (101 MHz, CDCl_3_): δ (ppm) = 59.71, 60.55, 85.27, 96.97, 120.89, 124.07 (2C), 126.69 (2C), 128.64 (2C), 129.02, 129.35, 130.17, 131.14, 131.18, 132.10 (2C), 138.62, 140.64, 141.89, 143.79, 148.19, 150.34. HRMS-ESI: *m/z* calcd for C24H15N3O3 [M+Na]+: 416.1006; Found: 416.0996.

2-[3-(5-nitrofuran-2-yl)oxiran-2-yl]-3-(phenylethynyl)quinoxaline (**23**)

*trans* isomer **23a**: yield: 81%, red solid, mp = 177 °C. 1H-NMR (400 MHz, CDCl_3_): δ (ppm) = 4.65 (d, J = 1.87 Hz, 1H), 5.39 (d, J = 1.88 Hz, 1H), 6.83 (d, J = 3.67 Hz, 1H), 7.35 (d, J = 3.67 Hz, 1H), 7.43–7.47 (m, 3H), 7.68–7.71 (m, 2H), 7.81–7.74 (m, 2H), 8.11–8.15 (m, 2H). 13C-NMR (101 MHz, CDCl_3_): δ (ppm) = 53.33, 57.35, 85.24, 97.49, 112.34, 113.32, 120.89, 128.69, 128.79 (2C), 129.06, 129.29, 130.29, 131.18, 131.30, 132.35 (2C), 138.90, 140.54, 142.02, 149.48, 152.62. HRMS-ESI: *m/z* calcd for C22H13N3O4 [M+Na]+: 406.0798; Found: 406.0794.

*cis* isomer **23b**: yield: 24%, red solid, mp = 150 °C. 1H-NMR (400 MHz, CDCl_3_): δ (ppm) = 5.55 (d, J = 5.83 Hz, 1H), 6.05 (d, J = 5.80 Hz, 1H), 6.75 (d, J = 3.68 Hz, 1H), 7.24 (d, J = 3.71 Hz, 1H), 7.47–7.52 (m, 3H), 7.79–7.86 (m, 4H), 8.02–8.05 (m, 1H), 8.16–8.19 (m, 1H). 13C-NMR (101 MHz, CDCl_3_): δ (ppm) = 36.92, 69.25, 84.82, 98.34, 112.04, 112.96, 120.69, 128.46, 128.89 (3C), 129.11, 130.46, 130.92, 131.23, 132.61 (2C), 137.22, 139.12, 142.10, 155.42, 156.33. HRMS-ESI: *m/z* calcd for C22H13N3O4 [M+Na]+: 406.0798; Found: 406.0792.

ethyl 3-[3-(phenylethynyl)quinoxalin-2-yl]oxirane-2-carboxylate (**24**)

*trans* isomer **24a**: yield: 80%, yellow solid, mp = 150 °C. 1H-NMR (400 MHz, CDCl_3_): δ (ppm) = 1.34 (t, J = 7.16 Hz, 3H), 4.19 (d, J = 1.76 Hz, 1H), 4.28–4.39 (m, 2H), 5.00 (d, J = 1.76 Hz, 1H), 7.40–7.46 (m, 3H), 7.66–7.68 (m, 2H), 7.78–7.82 (m, 2H), 8.08–8.12 (m, 2H). 13C-NMR (101 MHz, CDCl_3_): δ (ppm) = 14.14, 54.84, 55.32, 62.10, 85.18, 96.99, 121.06, 128.66 (2C), 128.98, 129.36, 130.13, 131.10, 131.23, 132.34 (2C), 138.84, 140.55, 141.97, 149.48, 167.94. HRMS-ESI: *m/z* calcd for C21H16N2O3 [M+Na]+: 367.1053; Found: 367.1051.

diethyl 3-[3-(phenylethynyl)quinoxalin-2-yl]oxirane-2,2-dicarboxylate (**25**)

yield: 84%, yellow solid, mp = 113 °C. 1H-NMR (400 MHz, CDCl_3_): δ (ppm) = 1.05 (t, J = 7.14 Hz, 3H), 1.31 (t, J = 7.14 Hz, 3H), 4.15 (q, J = 7.12 Hz, 2H), 4.30–4.39 (m, 2H), 5.32 (s, 1H), 7.38–7.44 (m, 3H), 7.66 (q, J = 3.08 Hz, 2H), 7.73–7.80 (m, 2H), 8.04 (q, J = 3.16 Hz, 1H), 8.08 (t, J = 4.74 Hz, 1H). 13C-NMR (101 MHz, CDCl_3_): δ (ppm) = 13.83, 13.95, 59.56, 62.07, 62.22, 63.21, 85.00, 97.58, 121.03, 128.64 (2C), 128.99, 129.28, 130.16, 131.10, 131.33, 132.35 (2C), 138.85, 139.95, 141.80, 147.94, 163.24, 165.18. HRMS-ESI: *m/z* calcd for C24H20N2O5 [M+H]+: 417.1445; Found: 417.1445.

### 3.2. In Vitro Biological Evaluation

The antiproliferative activity of each compound was evaluated through the number of viable cells, estimated using colorimetric 3-(4,5-dimethylthiazol-2-yl)- 2,5-diphenyltetra-zolium bromide (MTT; Sigma-Aldrich, Saint-Quentin-Fallavier, France) assay according to our previous work [22].

#### 3.2.1. Culture

Neuroblastoma cancer cells, namely SK-N-SH (ATCC, ref. HTB-11) and IMR-32 (ATCC, ref. CCL-127) cells, were purchased from the American Type Culture Collection and routinely maintained in standard RPMI 1640 (L-glutamine +) culture medium (Fisher Gibco^™^ RPMI-1640 Glutamax^™^) supplemented with 10% fetal bovine serum (Lonza) and 1% penicillin-streptomycin 5000 U/mL (Fisher Gibco^™^ Pen Strep) at 37 °C and 5% CO_2_. Cell cultures between 3 and 17 passages from defrosting were used for MTT assays.

#### 3.2.2. Drugs

Stock solutions of quinoxaline derivatives at 2.5 mM were prepared in dimethylsulfoxide (DMSO; Sigma-Aldrich, Saint-Quentin-Fallavier, France). The purity of all compounds was determined over 95%, before testing, by integrations on 1H-NMR spectra, and confirmed by UHPLC. Stock solutions were aliquoted and stored at −20 °C. For culture and experiments in living cells, the drugs were freshly diluted at an appropriate concentration in a culture medium.

#### 3.2.3. MTT Assay

Exponentially growing cells (37,500 cells/cm^2^ for SK-N-SH and 31,250 cells/cm^2^ for IMR-32 respectively) were detached with 20% trypsin Fisher Gibco^™^ (Waltham, MA, USA) and seeded by 150 μL/well of a 96-well plate (Falcon^®^ 96-well Clear Flat Bottom TC-treated Culture Microplate) for 24h for SK-N-SH, and 72h for IMR-32. The culture medium was then replaced by the same volume of freshly diluted drugs at an appropriate concentration (1 μM, 5 μM, 10 μM, 25 μM, 50 μM, 100 μM), or fresh culture medium for control wells. These concentrations were diluted 2-fold for drugs with IC_50_ < 10 μM for more accurate IC_50_ determination. Each of the 6 concentration points of the dilution range was iterated 4 times. To avoid more than 4% DSMO at the highest concentration, which could have an impact on cell viability, the maximal tested concentration was 100 μM. After 72h of drug treatment, the medium of each well was replaced by 150 μL of fresh medium containing MTT at 0.5 mg/mL, and cells were incubated at 37 °C for 4h. Then, the MTT solution was removed and 150 μL/well of DMSO was used to dilute the formazan crystals formed by the mitochondrial reductase of surviving cells. Finally, absorbance was measured at both 550 and 600 nm with a POLARstar Omega BMG LABTECH (Champigny s/Marne, France) plate reader. At least three independent experiments (in quadruplicate) were performed, and data were expressed as mean ± SD.

#### 3.2.4. Data Analysis

The analysis of the data obtained with the POLARstar Omega BMG LABTECH plate reader was carried out with RStudio [30] using packages from the tidyverse [38] for data visualization [39,40,41]. The unit of raw data obtained by plate reading is absorbance (UA), which was converted to the percentage of living cells by normalizing each absorbance well by the mean absorbance of control wells (Equation (1)).

(1) $${\mathrm{Living}\;\mathrm{cells}}_{i}\left(\%\right)=\frac{x_{i}\left(\mathrm{UA}\right)}{{\bar{x}}_{\mathrm{control}}\left(\mathrm{UA}\right)}\times 100$$

Outliers were identified by univariate analysis estimating location and scale. Thus, two *z*-scores were calculated to estimate data dispersion from mean (Equation (2)) and *median* (Equation (3)) allowing us to remove outliers with a 1.96 cut-off for 95% data accuracy [42].

(2) $$z_{i}=\frac{x_{i}-\bar{x}}{s}$$

(3) $$w_{i}=\frac{x_{i}-median\left(x_{i}\right)}{MAD}$$

IC_50_ was determined a by 4-parameter logistic regression fitting model using R package ’drm’ [43], as shown in equation (Equation (4)) [44], where *Y* is the response, *a* is the lower asymptote, *d* is the upper asymptote, *X* is the concentration, *c* is the EC50, *b* is the slope factor of the curve.

(4) $$Y=\frac{a-d}{1+{\left(\frac{X}{c}\right)}^{b}}+d$$

#### 3.2.5. Data Analysis

Statistical analyses were performed using R programming. *p*-values were calculated to quantify statistical significance, with the criterion set at *p* < 0.05. The IC_50_ obtained for each experiment was statistically analyzed first by the nonparametric Wilcoxon Mann–Whitney test to compare the IC_50_ distribution between the cell lines, between *cis* isomers and *trans* isomers, and between TDAE and Sonogashira series. Secondly, we performed a nonparametric Kruskal–Wallis one-way analysis of variance and finally a nonparametric post-hoc Dunn’s test for pairwise comparison to assess whether the IC_50_ of each isomer from each series differs significantly with varying epoxide substituents. All these tests were performed in RStudio using the native “R Stat Package” and “rstatix” package [45] for Dunn’s test.

### 3.3. In Silico Evaluation

#### 3.3.1. Molecular Docking

Crystal structures of human Topoisomerase IIβ in complex with DNA (PDB code: 3QX3) [31] and human Tissue Transglutaminase (PDB code: 4PYG) [32] were used as targets for docking simulations. The formula of the docked compounds was designed, and energy was minimized using the program MarvinSketch ChemAxon Ltd. (Basel, Switzerland) to evaluate possible binding modes. Proteins and ligands were prepared using ADFR and Python scripts provided with AutoDock-Vina. Hydrogens were not added to the proteins, but Gasteiger charges were added as the default set-up. We considered our target proteins as rigid objects while all ligands were handled as flexible. All simulations were performed with the AutoDock-Vina program [35,36] on a macOS terminal using the Vina forcefield. Center inputs for setting up search space were determined with ChimeraX [46,47] measuring the center of mass of each protein. The input sizes for the definition of search space were set up to a 27,000 Ångström^3^ cube, and exhaustiveness to 64. The other parameters were adopted as the program’s default values. Analysis of the results was performed by ranking the different ligand poses accordingly to their binding energy. We considered the molecule adopting the lowest energetic conformation as a promising compound. Visual analysis of the lowest energy solutions for each compound allowed us to identify the protein binding site. All the figures were drawn using the program ChimeraX.

#### 3.3.2. Pharmacokinetics Modeling with Simulation Plus Software Suite

The drug database for pharmacokinetic modeling was set up with MedChem Studio™ 4.0 from the Simulation-Plus software suite. Drug likeliness parameters were determined with ADMET Predictor^®^ 10.3. Pharmacokinetic parameters were determined with GastroPlus^®^ 9.8.2. From GastroPlus^®^, a compartmental model was repeated for each drug as an administration to a 70 kg fasted human with normal gut physiology in a 100 mg immediate-release tablet dosage form.

## 4. Conclusions

In this work, three chemical aspects of our synthesized compounds were evaluated. Firstly, we demonstrated the influence of stereochemistry on the antiproliferative activity of our compounds. The *trans* derivatives were significantly more active than *cis* ones from both TDAE and Sonogashira series. Secondly, we evaluated the influence of the substitution of position 2 on the quinoxaline core. Combining the epoxide and the arylethynyl group within the same structure in the Sonogashira series improved the antiproliferative activity of 6 out of the 20 compounds synthesized in the TDAE series. Since this induced a loss of activity for the other quinoxaline derivatives, it seems to demonstrate that the activity of most compounds is negatively influenced by the steric hindrance from the 2-arylethynyl substituent. Thirdly, we evaluated the influence of a variety of substituents on the oxirane ring in both the TDAE and Sonogashira series. As in the previously described TDAE series [22], the lowest IC_50_ was observed for the derivatives on which the epoxide is substituted by 5-nitrofuran (**11a**, **11b**). Our analysis also revealed that the nature of the epoxide’s substituent and the substitution pattern of the benzene ring can have a considerable impact on the antiproliferative activity of the synthesized compounds. Indeed, halogenated phenyl (**5, 6, 7**) and 5-nitrofuran **11** seem to be the most appropriate options from the TDAE series. Likewise, unsubstituted benzene **14**, fluorinated phenyl **20**, 5-nitrofuran **23,** and carboxylate **25** are the most active compounds in the Sonogashira series.

Moreover, we evaluated each compound against two neuroblastoma cell lines that were different in many aspects, more specifically by their expression of the efflux pump P-gp and the MYCN gene amplification. Since no significant difference could be demonstrated between IC_50_ against SK-N-SH and IMR-32, we could think that our compounds are not substrates of the P-gp which is an encouraging feature. Furthermore, most compounds are active against aggressive MYCN amplified cell line IMR-32, which is also an encouraging feature for further evaluations. In conclusion, we presented in this work multiple quinoxaline derivatives that display antiproliferative activity against resistant cell lines and aggressive ones. 

Further work will allow us to dig into the mechanism of action of these molecules. From our work, several hypotheses are made. From the similarity of structure with compounds XK-469 and CQS, which are both topoisomerase IIβ inhibitors, we could think that our products have the same target. Based on the results of our molecular docking study and the structural similarity with compounds XK-469 and CQS, which are both topoisomerase IIβ inhibitors, we were able to suggest that our products have the same target. Similarly, it allowed us to identify another potential target: the tissue transglutaminase responsible for tumor resistance. According to other oxirane ring carrier molecules described in the literature [23], other mechanisms could be at stake such as intracellular epoxide opening generating reactive oxygen species inducing apoptosis or DNA alkylation.

## Figures and Tables

**Table 1:** General results of antiproliferative activities.

		TDAE Series			Sonogashira Series		
R_1_	R_2_	Compound	IC_50_ ± SD μM		Compound	IC_50_ ± SD μM	
			SK-N-SH	IMR-32		SK-N-SH	IMR-32
H	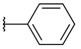	**2a**	26.9 ± 18.83	17.34 ± 8.58	**14a**	10.04 ± 8.26	9.18 ± 3.44
		**2b**	84.08 ± 35.27	66.93 ± 16.19	**14b**	17.75 ± 7.26	15.08 ± 5.25
H	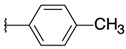	**3a**	38.01 ± 7.13	29.47 ± 6.98	**15a**	20.77 ± 10.06	15.27 ± 7.45
H	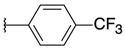	**4a**	>100	>100	**16a**	>100	>100
		**4b**	55.54 ± 27.05	42.12 ± 11.86	**16b**	>100	>100
H	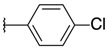	**5a**	20.25 ± 4.45	22.69 ± 7.49	**17a**	>100	>100
		**5b**	44.09 ± 12.49	40.12 ± 5.69	**17b**	>100	>100
H	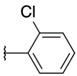	**6a**	19.86 ± 5.96	12.98 ± 3.39	**18a**	>100	>100
H	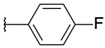	**7a**	15.94 ± 5.83	13.08 ± 0.69	**19a**	>100	29.93 ± 14.82
		**7b**	60.29 ± 11.04	44.58 ± 3.31	**19b**	>100	>100
H	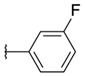	**8a**	36.21 ± 15.49	24.8 ± 11.81	**20a**	12.08 ± 2.15	14.81 ± 2.93
		**8b**	33 ± 22.3	71.64 ± 25.6	**20b**	9.5 ± 4.12	10.55 ± 4.79
H	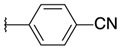	**9a**	39.29 ± 14.87	25.59 ± 5.37	**21a**	>100	>100
		**9b**	66.24 ± 7.13	59.14 ± 15.68	**21b**	>100	>100
H	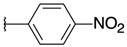	**10a**	74.29 ± 16.84	>100	**22a**	>100	27.62 ± 13.18
		**10b**	47.93 ± 6.73	>100	**22b**	>100	>100
H	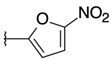	**11a**	2.49 ± 1.33	3.96 ± 2.03	**23a**	10.95 ± 0.62	14.31 ± 2.35
		**11b**	5.3 ± 2.12	7.12 ± 1.59	**23b**	25.35 ± 6.87	25.65 ± 1.46
H	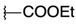	**12a**	47.05 ± 22.91	38.26 ± 18.28	**24a**	58.87 ± 7.79	63.2 ± 3.82
COOEt	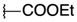	**13**	55.77 ± 26.37	47.81 ± 7.52	**25**	10.31 ± 2.4	7.26 ± 2.19
		XK-469	4.6 ± 1.0	13.0 ± 2.9			

**Table 2:** Binding energies from in silico simulations towards human topoisomerase II β and tissue transglutaminase.

Binding Energies (kcal/mol)		
Compounds	h Topoisomerase II β	h Tissue Transglutaminase 2
XK-469	−7.489	-
GK-13	-	−7.750
**11a**	−6.993	−7.147
**11b**	−6.564	−6.368
**14a**	−8.294	−7.758
**25**	−7.527	−6.507

**Table 3:** Lipinski’s rule of 5.

TDAE Series					Sonogashira Series				
Compound	MW	LogP	H-BA	H-BD	Compound	MW	LogP	H-BA	H-BD
**2**	282.731	3.280	3	0	14	348.407	4.317	3	0
**3**	296.758	3.778	3	0	15	362.434	4.831	3	0
**4**	350.729	4.113	3	0	16	416.405	5.172	3	0
**5**	317.176	3.982	3	0	17	382.852	5.026	3	0
**6**	317.176	3.854	3	0	18	382.852	4.845	3	0
**7**	300.722	3.630	3	0	19	366.397	4.679	3	0
**8**	300.722	3.549	3	0	20	366.397	4.606	3	0
**9**	307.741	3.192	4	0	21	373.417	4.184	4	0
**10**	327.729	2.912	5	0	22	393.404	4.170	5	0
**11**	317.69	2.281	6	0	23	383.366	3.550	6	0
**12**	278.697	2.064	5	0	24	344.372	3.145	5	0
**13**	350.761	2.207	7	0	25	416.436	3.505	7	0

## Data Availability

Data is contained within the article and Supplementary Material.

## Supplementary Materials

The following supporting information can be downloaded at: https://www.mdpi.com/article/10.3390/ph15070781/s1, Supplementary data involving ^1^H-NMR and ^13^C-NMR data of all the synthesized compounds.
