# Supplementary material for: Synthesis, In Vitro Antiproliferative Activity, and In Silico Evaluation of Novel Oxiranyl-Quinoxaline Derivatives
Source: Pharmaceuticals (Basel). 2022 Jun 23;15(7):781. doi: 10.3390/ph15070781 (PMC9319868; doi:10.3390/ph15070781)
Supplement: Supplementary file 1 [file pharmaceuticals-15-00781-s001.zip › pharmaceuticals-1671926-supplementary.pdf]

pharmaceuticals

Article

Synthesis, In Vitro Antiproliferative Activity, and In Silico Evaluation of Novel Oxiranyl-Quinoxaline Derivatives

Vincent Montero [1], Marc Montana [1,2], Omar Khoumeri [1], Florian Correard [3,4], Marie-Anne Estève [3,4] and Patrice Vanelle [1,5,*]

[1] Aix Marseille Univ, CNRS, ICR UMR 7273, Equipe Pharmaco-Chimie Radicalaire, Faculté de Pharmacie, 13385 Marseille Cedex 05, France; vincent.montero@etu.univ-amu.fr (V.M.); marc.montana@univ-amu.fr (M.M.); omar.khoumeri@univ-amu.fr (O.K.)
[2] APHM, Hôpital Timone, Oncopharma, 13005 Marseille, France
[3] Aix Marseille Univ, CNRS, INP, Inst Neurophysiopathol, 13385 Marseille Cedex 05, France; florian.correard@univ-amu.fr (F.C.); marie-anne.esteve@univ-amu.fr (M.-A.E.)
[4] APHM, Hôpital Timone, Service Pharmacie, 13005 Marseille, France
[5] APHM, Hôpital Conception, Service Central de la Qualité et de l'Information Pharmaceutiques, 13005 Marseille, France
* Correspondence: patrice.vanelle@univ-amu.fr; Tel.: +33-4-91-83-55-80

SUPPLEMENTARY DATA

- Compound 2a
- Compound 2b
- Compound 3a
- Compound 4a
- Compound 4b
- Compound 5a
- Compound 5b
- Compound 6a
- Compound 7a
- Compound 7b
- Compound 8a
- Compound 8b
- Compound 9a
- Compound 9b
- Compound 10a
- Compound 10b
- Compound 11a
- Compound 11b
- Compound 12a
- Compound 13
- Compound 14a
- Compound 14b
- Compound 15a
- Compound 16a
- Compound 16b
- Compound 17a
- Compound 17b
- Compound 18a
- Compound 19a
- Compound 19b
- Compound 20a
- Compound 20b
- Compound 21a
- Compound 21b
- Compound 22a
- Compound 22b
- Compound 23a
- Compound 23b
- Compound 24a
- Compound 25

Compound 2a

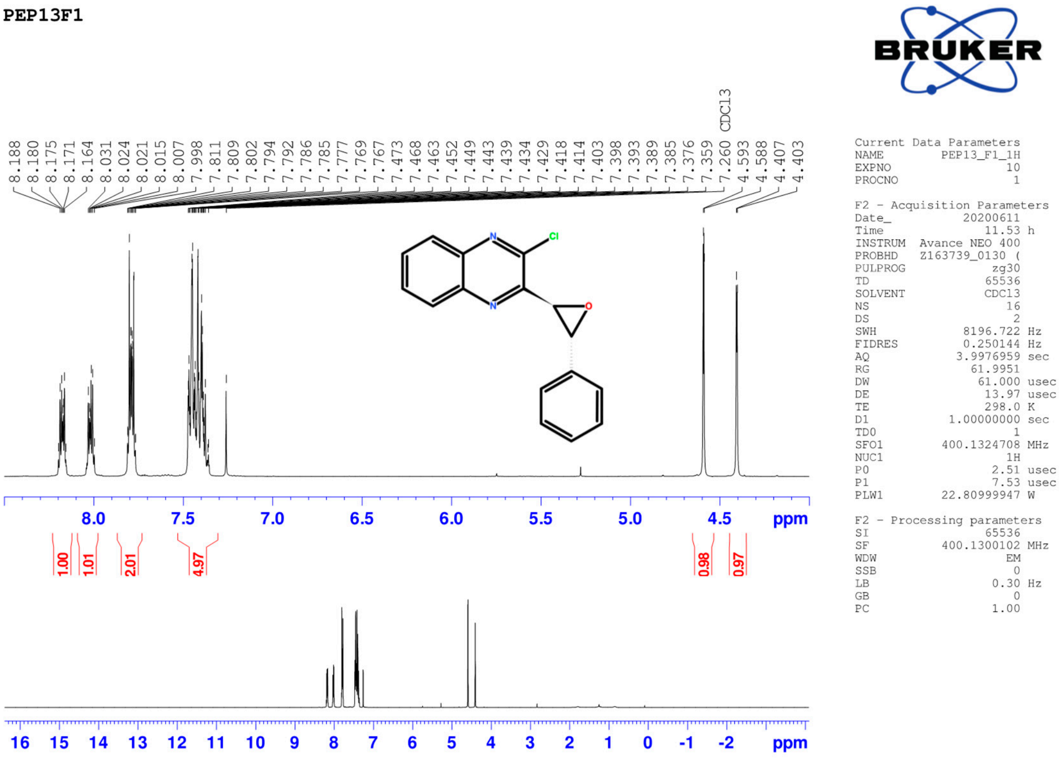
PEP13F1
BRUKER
8.188 8.180 8.175 8.171 8.164 8.031 8.024 8.021 8.015 8.007 7.998 7.811 7.809 7.802 7.794 7.792 7.786 7.785 7.777 7.769 7.767 7.473 7.468 7.463 7.452 7.449 7.443 7.439 7.434 7.429 7.418 7.414 7.403 7.398 7.393 7.389 7.385 7.376 7.359 7.260 CDCl3 4.593 4.588 4.407 4.403
Cl
N
N
O
1.00 1.01 2.01 4.97 0.98 0.97
8.0 7.5 7.0 6.5 6.0 5.5 5.0 4.5 ppm
16 15 14 13 12 11 10 9 8 7 6 5 4 3 2 1 0 -1 -2 ppm
Current Data Parameters
NAME PEP13_F1_1H
EXPNO 10
PROCNO 1
F2 - Acquisition Parameters
Date_ 20200611
Time 11.53 h
INSTRUM Avance NEO 400
PROBHD Z163739_0130 (
PULPROG zg30
TD 65536
SOLVENT CDCl3
NS 16
DS 2
SWH 8196.722 Hz
FIDRES 0.250144 Hz
AQ 3.9976959 sec
RG 61.9951
DW 61.000 usec
DE 13.97 usec
TE 298.0 K
D1 1.00000000 sec
TD0 1
SFO1 400.1324708 MHz
NUC1 1H
P0 2.51 usec
P1 7.53 usec
PLW1 22.80999947 W
F2 - Processing parameters
SI 65536
SF 400.1300102 MHz
WDW EM
SSB 0
LB 0.30 Hz
GB 0
PC 1.00

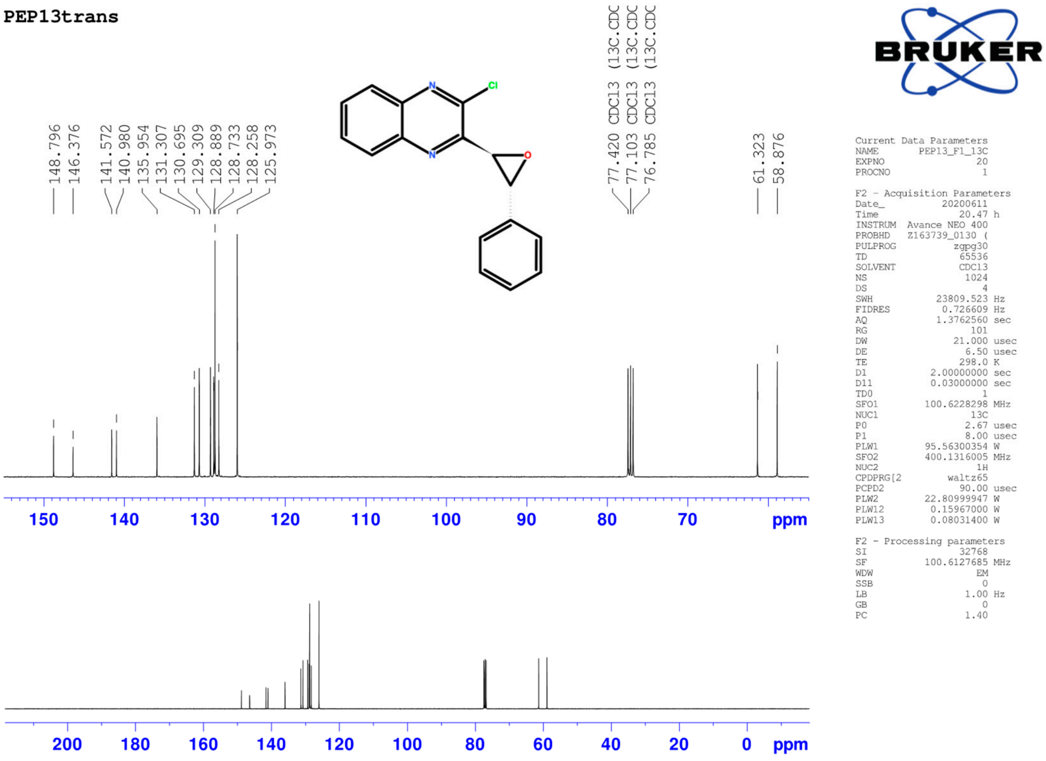
PEP13trans
BRUKER
148.796 146.376 141.572 140.980 135.954 131.307 130.695 129.309 128.889 128.733 128.258 125.973
77.420 CDCl3 (13C.CDC
77.103 CDCl3 (13C.CDC
76.785 CDCl3 (13C.CDC
61.323 58.876
Cl
N
N
O
150 140 130 120 110 100 90 80 70 ppm
200 180 160 140 120 100 80 60 40 20 0 ppm
Current Data Parameters
NAME PEP13_F1_13C
EXPNO 20
PROCNO 1
F2 - Acquisition Parameters
Date_ 20200611
Time 20.47 h
INSTRUM Avance NEO 400
PROBHD Z163739_0130 (
PULPROG zgpg30
TD 65536
SOLVENT CDCl3
NS 1024
DS 4
SWH 23809.523 Hz
FIDRES 0.726609 Hz
AQ 1.3762560 sec
RG 101
DW 21.000 usec
DE 6.50 usec
TE 298.0 K
D1 2.00000000 sec
D11 0.03000000 sec
TD0 1
SFO1 100.6228298 MHz
NUC1 13C
P0 2.67 usec
P1 8.00 usec
PLW1 95.56300354 W
SFO2 400.1316005 MHz
NUC2 1H
CPDPRG[2 waltz65
PCPD2 90.00 usec
PLW2 22.80999947 W
PLW12 0.15967000 W
PLW13 0.08031400 W
F2 - Processing parameters
SI 32768
SF 100.6127685 MHz
WDW EM
SSB 0
LB 1.00 Hz
GB 0
PC 1.40

Data File C:\Chem32\...rganique\prestation_chimie_organique 2022-05-31\OnlineEdited--016.D
Sample Name: PEP13F1

===

Acq. Operator : SYSTEM Seq. Line : 16
Acq. Instrument : LC1290 Location : P1-B6
Injection Date : 31/5/2022 5:05:54 PM Inj : 1
Inj Volume : 1.000 µl
Method : C:\Chem32\1\Data\prestation_chimie_organique\prestation_chimie_organique 2022-05-31\prestation_chimie_org.M (Sequence Method)
Last changed : 31/5/2022 1:07:56 PM by SYSTEM
Additional Info : Peak(s) manually integrated

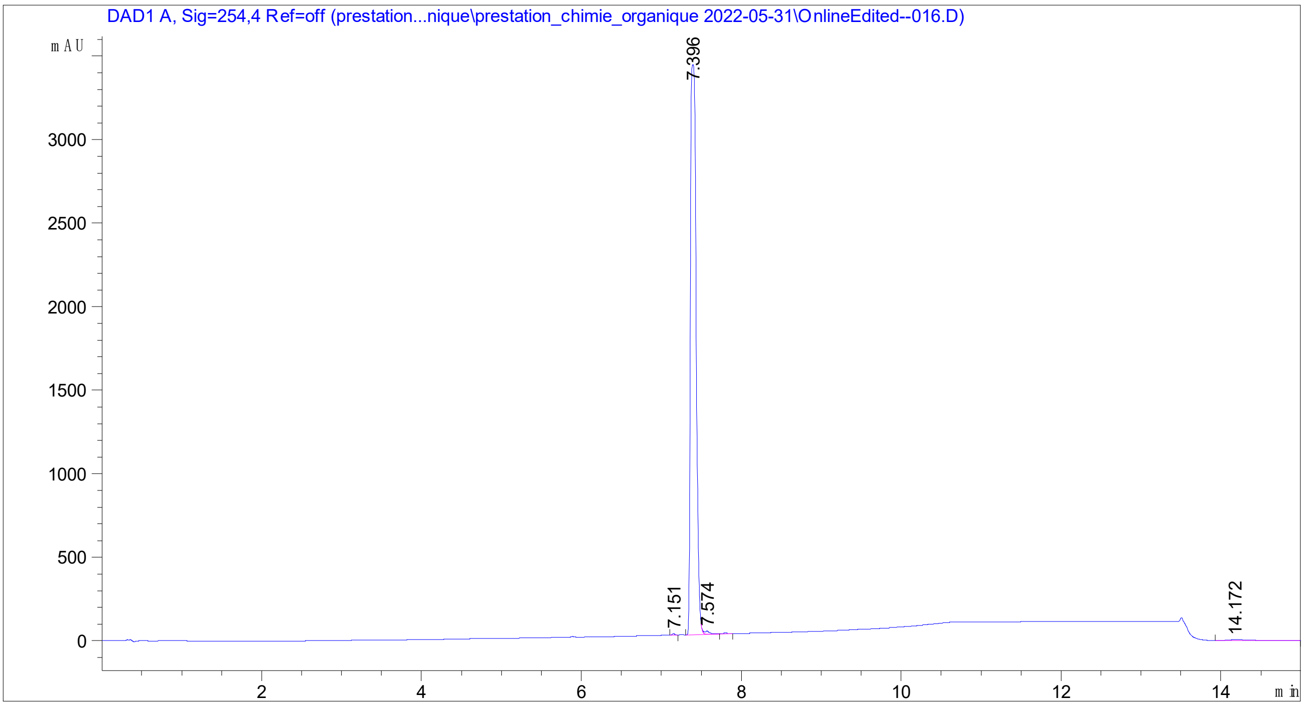

===

Area Percent Report

===

Sorted By : Signal
Multiplier : 1.0000
Dilution : 1.0000
Use Multiplier & Dilution Factor with ISTDs

Signal 1: DAD1 A, Sig=254,4 Ref=off

Peak #	RetTime [min]	Type	Width [min]	Area [mAU*s]	Height [mAU]	Area %
1	7.151	BV	0.0378	22.95683	9.30063	0.1344
2	7.396	BV R	0.0785	1.68333e4	3412.13135	98.5352
3	7.574	VV E	0.0666	96.25467	19.49225	0.5634
4	14.172	BBA	0.2724	131.02750	6.17944	0.7670

Totals : 1.70835e4 3447.10367

===

Compound 2b

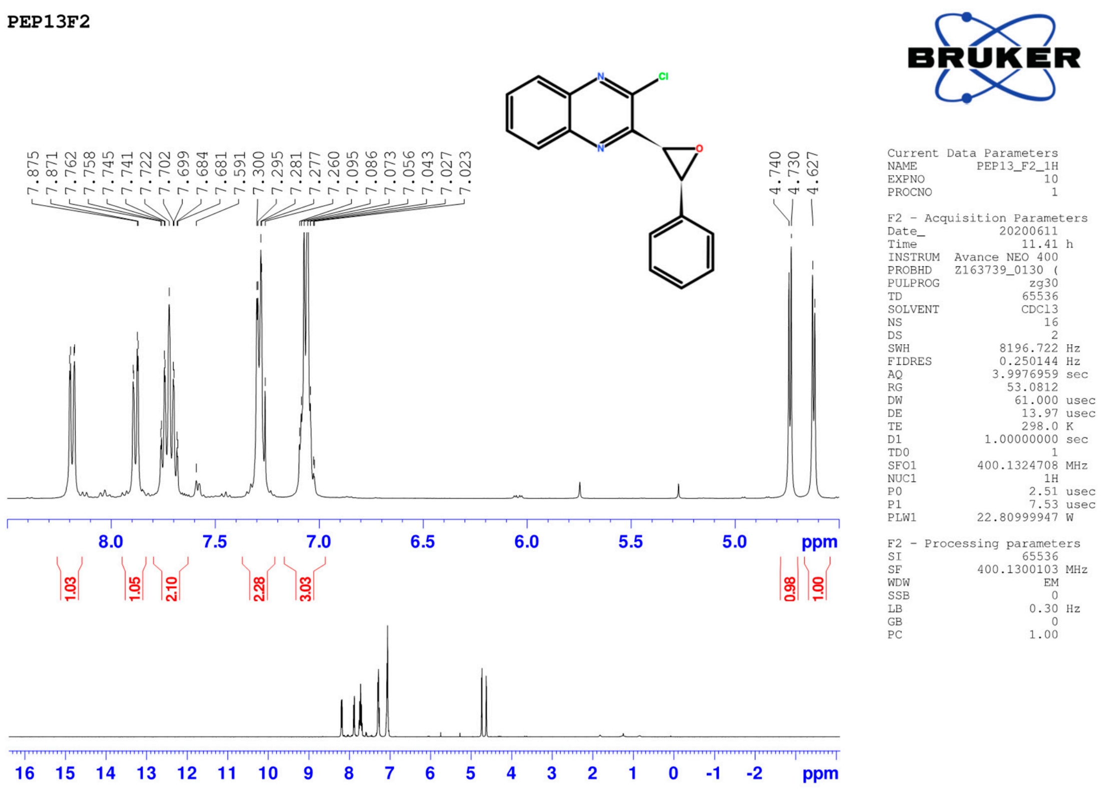
PEP13F2
BRUKER
7.875 7.871 7.762 7.758 7.745 7.741 7.722 7.702 7.699 7.684 7.681 7.591 7.300 7.295 7.281 7.277 7.260 7.095 7.086 7.073 7.056 7.043 7.027 7.023
4.740 4.730 4.627
N N Cl O
1.03 1.05 2.10 2.28 3.03 0.98 1.00
8.0 7.5 7.0 6.5 6.0 5.5 5.0 ppm
16 15 14 13 12 11 10 9 8 7 6 5 4 3 2 1 0 -1 -2 ppm
Current Data Parameters
NAME PEP13_F2_1H
EXPNO 10
PROCNO 1
F2 - Acquisition Parameters
Date_ 20200611
Time 11.41 h
INSTRUM Avance NEO 400
PROBHD Z163739_0130 (
PULPROG zg30
TD 65536
SOLVENT CDCl3
NS 16
DS 2
SWH 8196.722 Hz
FIDRES 0.250144 Hz
AQ 3.9976959 sec
RG 53.0812
DW 61.000 usec
DE 13.97 usec
TE 298.0 K
D1 1.00000000 sec
TD0 1
SFO1 400.1324708 MHz
NUC1 1H
P0 2.51 usec
P1 7.53 usec
PLW1 22.80999947 W
F2 - Processing parameters
SI 65536
SF 400.1300103 MHz
WDW EM
SSB 0
LB 0.30 Hz
GB 0
PC 1.00

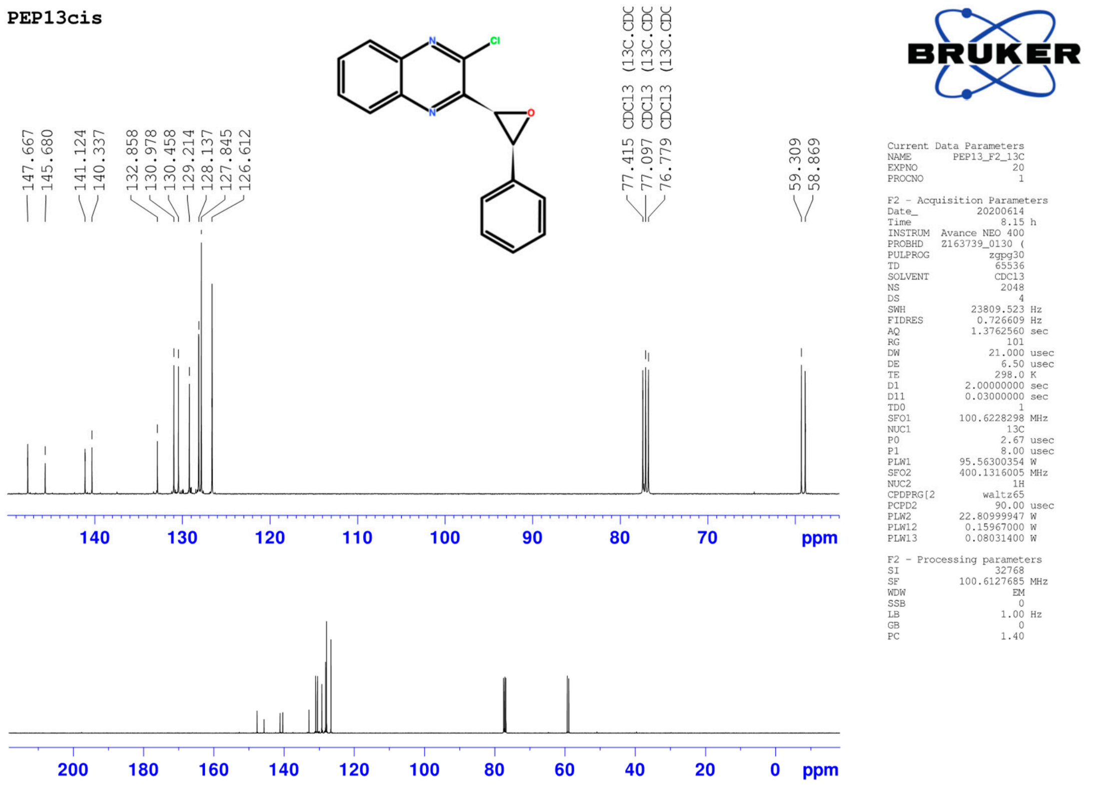
PEP13cis
BRUKER
147.667 145.680 141.124 140.337 132.858 130.978 130.458 129.214 128.137 127.845 126.612
77.415 CDCl3 (13C.CDC
77.097 CDCl3 (13C.CDC
76.779 CDCl3 (13C.CDC
59.309 58.869
N N Cl O
140 130 120 110 100 90 80 70 ppm
200 180 160 140 120 100 80 60 40 20 0 ppm
Current Data Parameters
NAME PEP13_F2_13C
EXPNO 20
PROCNO 1
F2 - Acquisition Parameters
Date_ 20200614
Time 8.15 h
INSTRUM Avance NEO 400
PROBHD Z163739_0130 (
PULPROG zgpg30
TD 65536
SOLVENT CDCl3
NS 2048
DS 4
SWH 23809.523 Hz
FIDRES 0.726609 Hz
AQ 1.3762560 sec
RG 101
DW 21.000 usec
DE 6.50 usec
TE 298.0 K
D1 2.00000000 sec
D11 0.03000000 sec
TD0 1
SFO1 100.6228298 MHz
NUC1 13C
P0 2.67 usec
P1 8.00 usec
PLW1 95.56300354 W
SFO2 400.1316005 MHz
NUC2 1H
CPDPRG[2 waltz65
PCPD2 90.00 usec
PLW2 22.80999947 W
PLW12 0.15967000 W
PLW13 0.08031400 W
F2 - Processing parameters
SI 32768
SF 100.6127685 MHz
WDW EM
SSB 0
LB 1.00 Hz
GB 0
PC 1.40

Data File C:\Chem32\...rganique\prestation_chimie_organique 2022-05-31\OnlineEdited--017.D
Sample Name: PEP13F2

===
Acq. Operator : SYSTEM Seq. Line : 17
Acq. Instrument : LC1290 Location : P1-B7
Injection Date : 31/5/2022 5:21:42 PM Inj : 1
Inj Volume : 1.000 µl
Method : C:\Chem32\1\Data\prestation_chimie_organique\prestation_chimie_organique 2022-05-31\prestation_chimie_org.M (Sequence Method)
Last changed : 31/5/2022 1:07:56 PM by SYSTEM
Additional Info : Peak(s) manually integrated

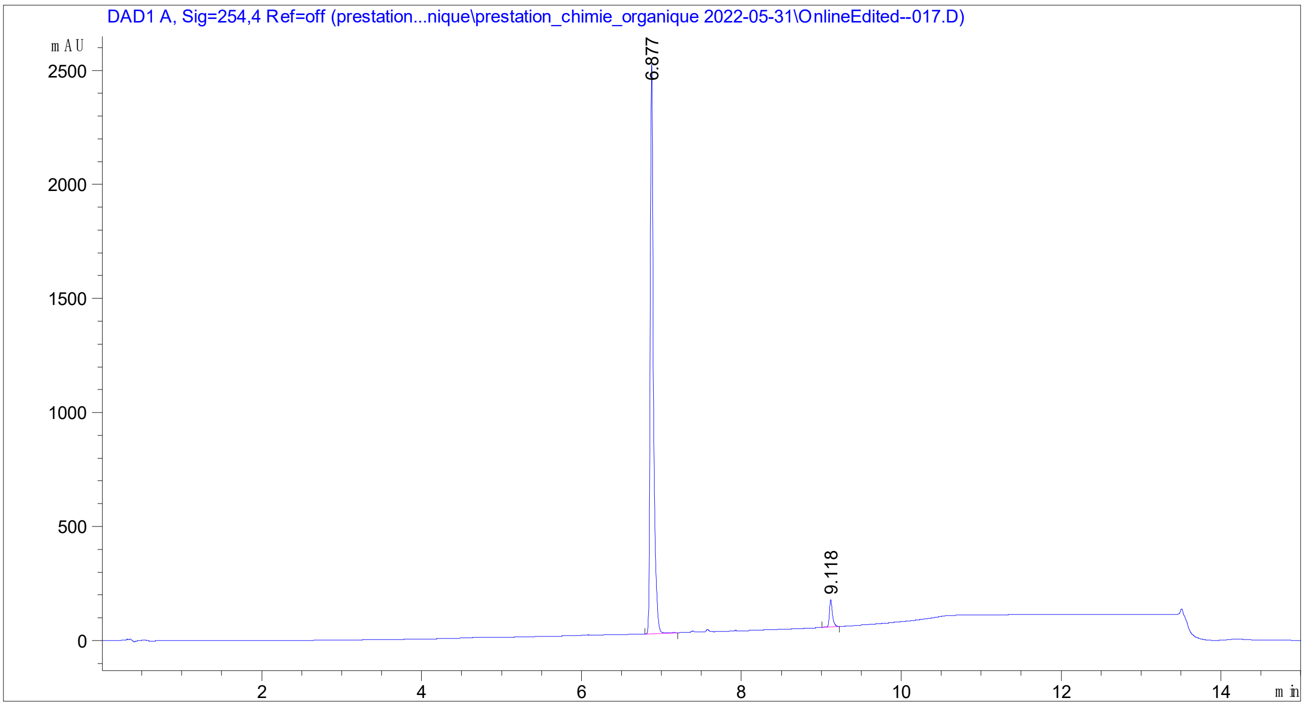

===
Area Percent Report
===

Sorted By : Signal
Multiplier : 1.0000
Dilution : 1.0000
Use Multiplier & Dilution Factor with ISTDs

Signal 1: DAD1 A, Sig=254,4 Ref=off

Peak #	RetTime [min]	Type	Width [min]	Area [mAU*s]	Height [mAU]	Area %
1	6.877	BV R	0.0424	7131.84277	2494.35254	95.8686
2	9.118	VB R	0.0377	307.34372	120.69427	4.1314
Totals :				7439.18649	2615.04681	

===
*** End of Report ***

Compound 3a

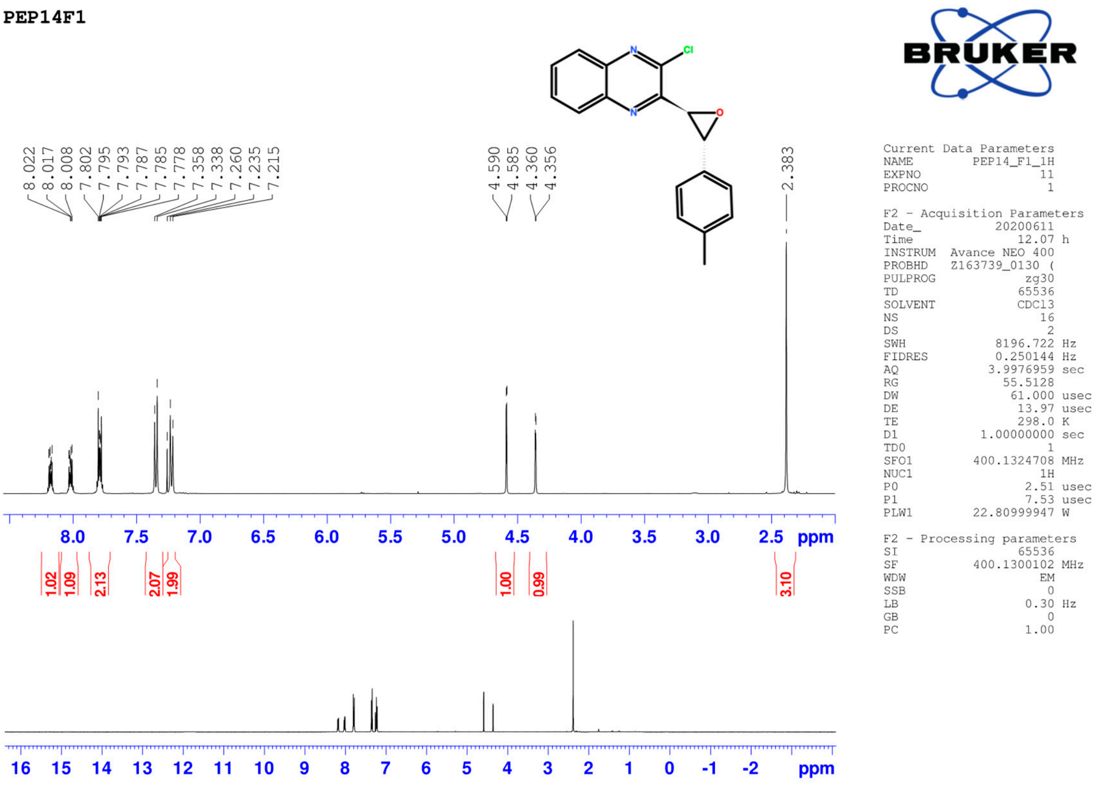
PEP14F1
8.022
8.017
8.008
7.802
7.795
7.793
7.787
7.785
7.778
7.358
7.338
7.260
7.235
7.215
4.590
4.585
4.360
4.356
2.383
N
Cl
N
O
BRUKER
Current Data Parameters
NAME PEP14_F1_1H
EXPNO 11
PROCNO 1
F2 - Acquisition Parameters
Date_ 20200611
Time 12.07 h
INSTRUM Avance NEO 400
PROBHD Z163739_0130 (
PULPROG zg30
TD 65536
SOLVENT CDCl3
NS 16
DS 2
SWH 8196.722 Hz
FIDRES 0.250144 Hz
AQ 3.9976959 sec
RG 55.5128
DW 61.000 usec
DE 13.97 usec
TE 298.0 K
D1 1.00000000 sec
TD0 1
SFO1 400.1324708 MHz
NUC1 1H
P0 2.51 usec
P1 7.53 usec
PLW1 22.80999947 W
F2 - Processing parameters
SI 65536
SF 400.1300102 MHz
WDW EM
SSB 0
LB 0.30 Hz
GB 0
PC 1.00
8.0 7.5 7.0 6.5 6.0 5.5 5.0 4.5 4.0 3.5 3.0 2.5 ppm
1.02 1.09 2.13 2.07 1.99 1.00 0.99 3.10
16 15 14 13 12 11 10 9 8 7 6 5 4 3 2 1 0 -1 -2 ppm

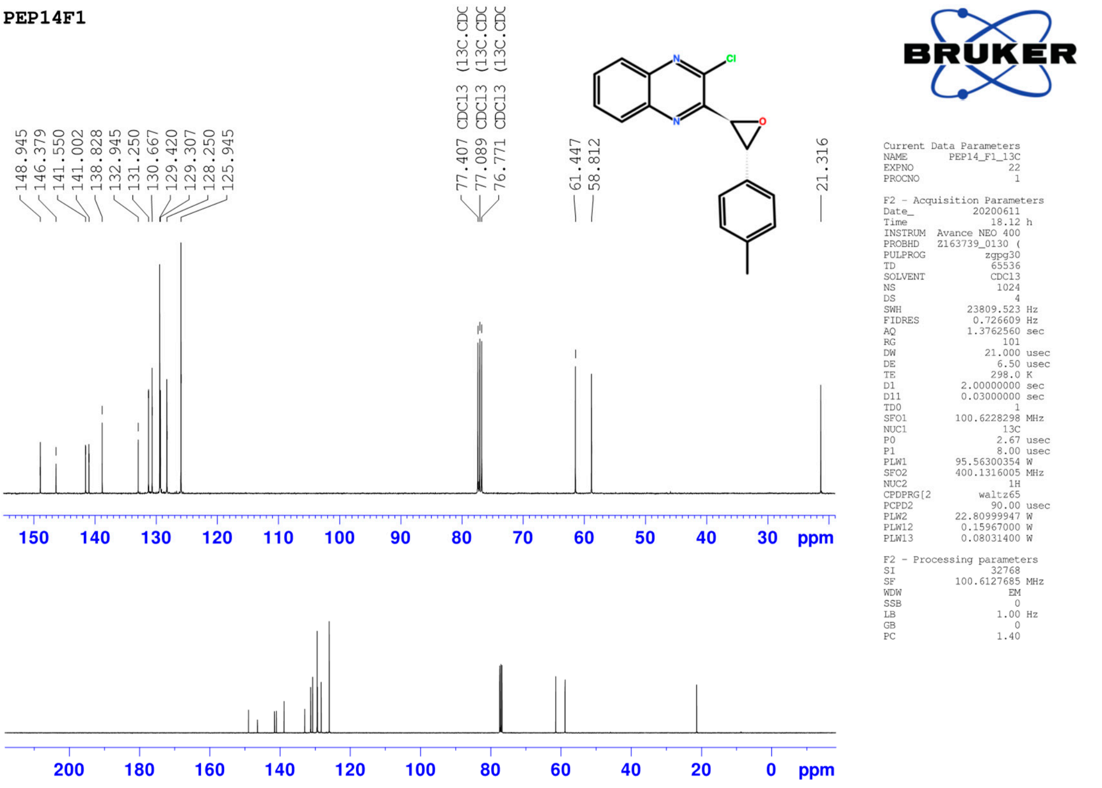
PEP14F1
148.945
146.379
141.550
141.002
138.828
132.945
131.250
130.667
129.420
129.307
128.250
125.945
77.407 CDCl3 (13C.CDC
77.089 CDCl3 (13C.CDC
76.771 CDCl3 (13C.CDC
61.447
58.812
21.316
N
Cl
N
O
BRUKER
Current Data Parameters
NAME PEP14_F1_13C
EXPNO 22
PROCNO 1
F2 - Acquisition Parameters
Date_ 20200611
Time 18.12 h
INSTRUM Avance NEO 400
PROBHD Z163739_0130 (
PULPROG zgpg30
TD 65536
SOLVENT CDCl3
NS 1024
DS 4
SWH 23809.523 Hz
FIDRES 0.726609 Hz
AQ 1.3762560 sec
RG 101
DW 21.000 usec
DE 6.50 usec
TE 298.0 K
D1 2.00000000 sec
D11 0.03000000 sec
TD0 1
SFO1 100.6228298 MHz
NUC1 13C
P0 2.67 usec
P1 8.00 usec
PLW1 95.56300354 W
SFO2 400.1316005 MHz
NUC2 1H
CPDPRG[2 waltz65
PCPD2 90.00 usec
PLW2 22.80999947 W
PLW12 0.15967000 W
PLW13 0.08031400 W
F2 - Processing parameters
SI 32768
SF 100.6127685 MHz
WDW EM
SSB 0
LB 1.00 Hz
GB 0
PC 1.40
150 140 130 120 110 100 90 80 70 60 50 40 30 ppm
200 180 160 140 120 100 80 60 40 20 0 ppm

```
Data File C:\Chem32\...rganique\prestation_chimie_organique 2022-05-31\OnlineEdited--018.D
Sample Name: PEP14F1

    =====================================================================
    Acq. Operator   : SYSTEM                        Seq. Line :  18
    Acq. Instrument : LC1290                         Location :   P1-B8
    Injection Date  : 31/5/2022 5:37:31 PM                Inj :   1
                                                   Inj Volume : 1.000 µl
    Method          : C:\Chem32\1\Data\prestation_chimie_organique\prestation_chimie_organique
                      2022-05-31\prestation_chimie_org.M (Sequence Method)
    Last changed    : 31/5/2022 1:07:56 PM by SYSTEM
    Additional Info : Peak(s) manually integrated
```

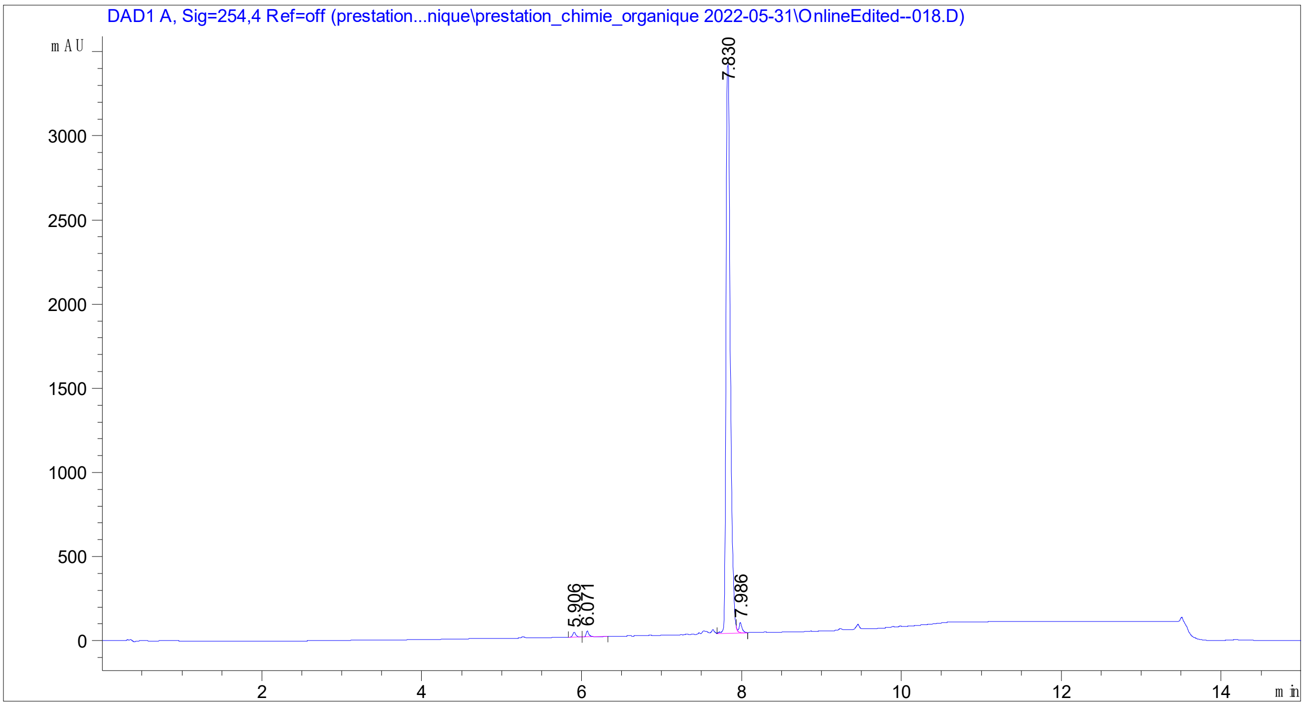


```
    =====================================================================
                             Area Percent Report
    =====================================================================

    Sorted By             :      Signal
    Multiplier            :      1.0000
    Dilution              :      1.0000
    Use Multiplier & Dilution Factor with ISTDs


    Signal 1: DAD1 A, Sig=254,4 Ref=off

    Peak RetTime Type  Width     Area      Height     Area
      #   [min]        [min]   [mAU*s]     [mAU]        %
    ----|-------|----|-------|----------|----------|--------|
       1   5.906 BV    0.0412   80.47663   29.15832   0.6037
       2   6.071 VV R  0.0389   92.81735   35.06199   0.6962
       3   7.830 VV R  0.0599 1.29985e4  3377.83521  97.5022
       4   7.986 VB E  0.0404  159.69933   59.38504   1.1979

    Totals :                  1.33315e4  3501.44055


    =====================================================================
```

Compound 4a

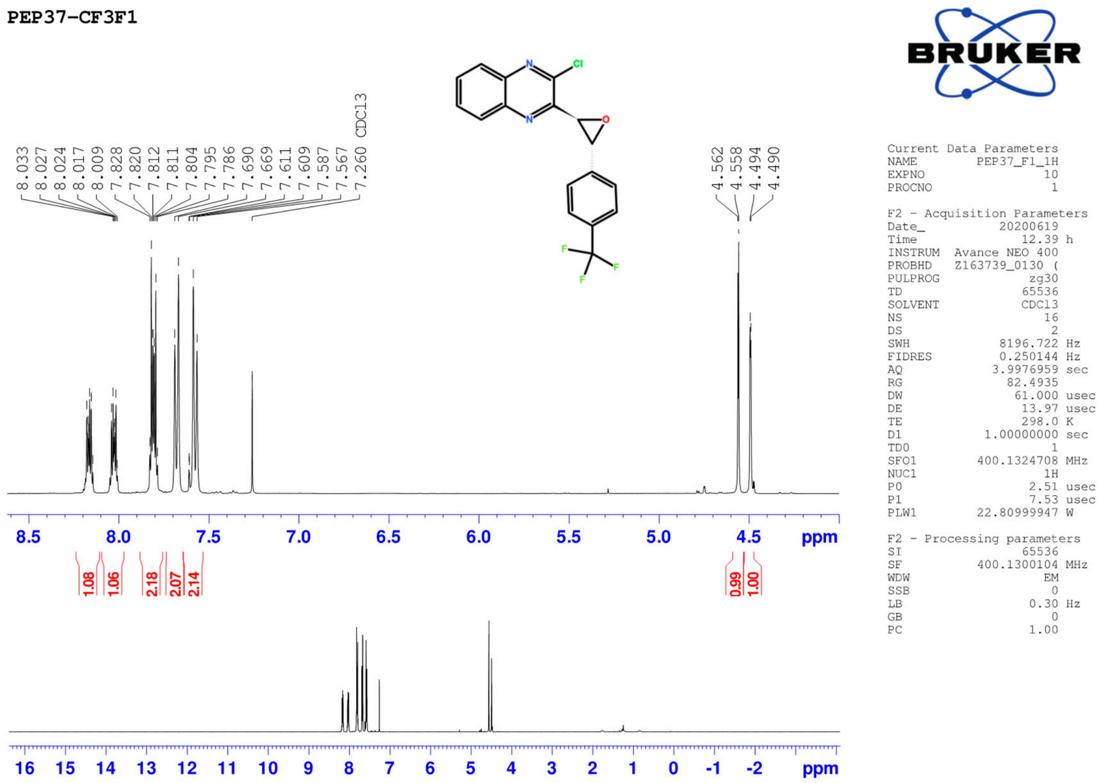
PEP37-CF3F1
8.033 8.027 8.024 8.017 8.009 7.828 7.820 7.812 7.811 7.804 7.795 7.786 7.690 7.669 7.611 7.609 7.587 7.567 7.260 CDCl3
4.562 4.558 4.494 4.490
Cl
N
N
O
F
F
F
BRUKER
Current Data Parameters
NAME PEP37_F1_1H
EXPNO 10
PROCNO 1
F2 - Acquisition Parameters
Date_ 20200619
Time 12.39 h
INSTRUM Avance NEO 400
PROBHD Z163739_0130 (
PULPROG zg30
TD 65536
SOLVENT CDCl3
NS 16
DS 2
SWH 8196.722 Hz
FIDRES 0.250144 Hz
AQ 3.9976959 sec
RG 82.4935
DW 61.000 usec
DE 13.97 usec
TE 298.0 K
D1 1.00000000 sec
TD0 1
SFO1 400.1324708 MHz
NUC1 1H
P0 2.51 usec
P1 7.53 usec
PLW1 22.80999947 W
F2 - Processing parameters
SI 65536
SF 400.1300104 MHz
WDW EM
SSB 0
LB 0.30 Hz
GB 0
PC 1.00
8.5 8.0 7.5 7.0 6.5 6.0 5.5 5.0 4.5 ppm
1.08 1.06 2.18 2.07 2.14 0.99 1.00
16 15 14 13 12 11 10 9 8 7 6 5 4 3 2 1 0 -1 -2 ppm

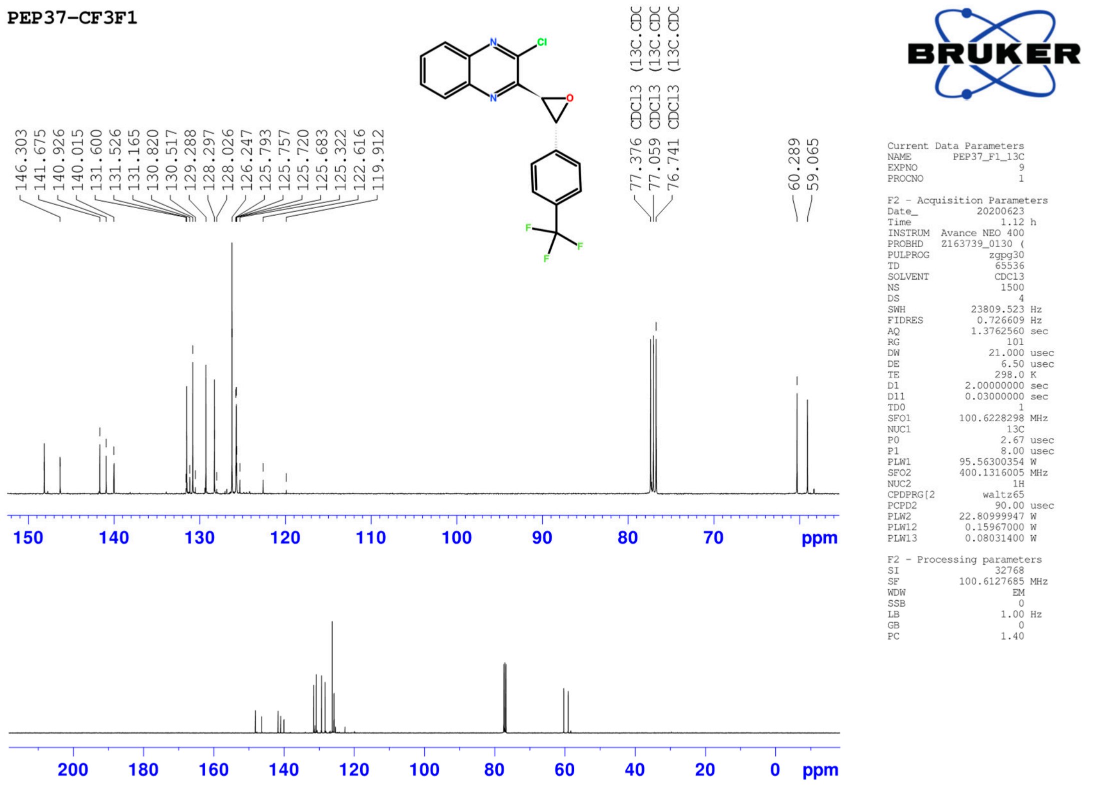
PEP37-CF3F1
146.303 141.675 140.926 140.015 131.600 131.526 131.165 130.820 130.517 129.288 128.297 128.026 126.247 125.793 125.757 125.720 125.683 125.322 122.616 119.912
77.376 CDCl3 (13C.CDC
77.059 CDCl3 (13C.CDC
76.741 CDCl3 (13C.CDC
60.289 59.065
Cl
N
N
O
F
F
F
BRUKER
Current Data Parameters
NAME PEP37_F1_13C
EXPNO 9
PROCNO 1
F2 - Acquisition Parameters
Date_ 20200623
Time 1.12 h
INSTRUM Avance NEO 400
PROBHD Z163739_0130 (
PULPROG zgpg30
TD 65536
SOLVENT CDCl3
NS 1500
DS 4
SWH 23809.523 Hz
FIDRES 0.726609 Hz
AQ 1.3762560 sec
RG 101
DW 21.000 usec
DE 6.50 usec
TE 298.0 K
D1 2.00000000 sec
D11 0.03000000 sec
TD0 1
SFO1 100.6228298 MHz
NUC1 13C
P0 2.67 usec
P1 8.00 usec
PLW1 95.56300354 W
SFO2 400.1316005 MHz
NUC2 1H
CPDPRG[2 waltz65
PCPD2 90.00 usec
PLW2 22.80999947 W
PLW12 0.15967000 W
PLW13 0.08031400 W
F2 - Processing parameters
SI 32768
SF 100.6127685 MHz
WDW EM
SSB 0
LB 1.00 Hz
GB 0
PC 1.40
150 140 130 120 110 100 90 80 70 ppm
200 180 160 140 120 100 80 60 40 20 0 ppm

Data File C:\Chem32\...rganique\prestation_chimie_organique 2022-05-31\OnlineEdited--019.D
Sample Name: PEP37F1

===

Acq. Operator	: SYSTEM	Seq. Line :	19
Acq. Instrument	: LC1290	Location :	P1-B9
Injection Date	: 31/5/2022 5:56:32 PM	Inj :	1
		Inj Volume :	1.000 µl

Method : C:\Chem32\1\Data\prestation_chimie_organique\prestation_chimie_organique 2022-05-31\prestation_chimie_org.M (Sequence Method)
Last changed : 31/5/2022 1:07:56 PM by SYSTEM
Additional Info : Peak(s) manually integrated

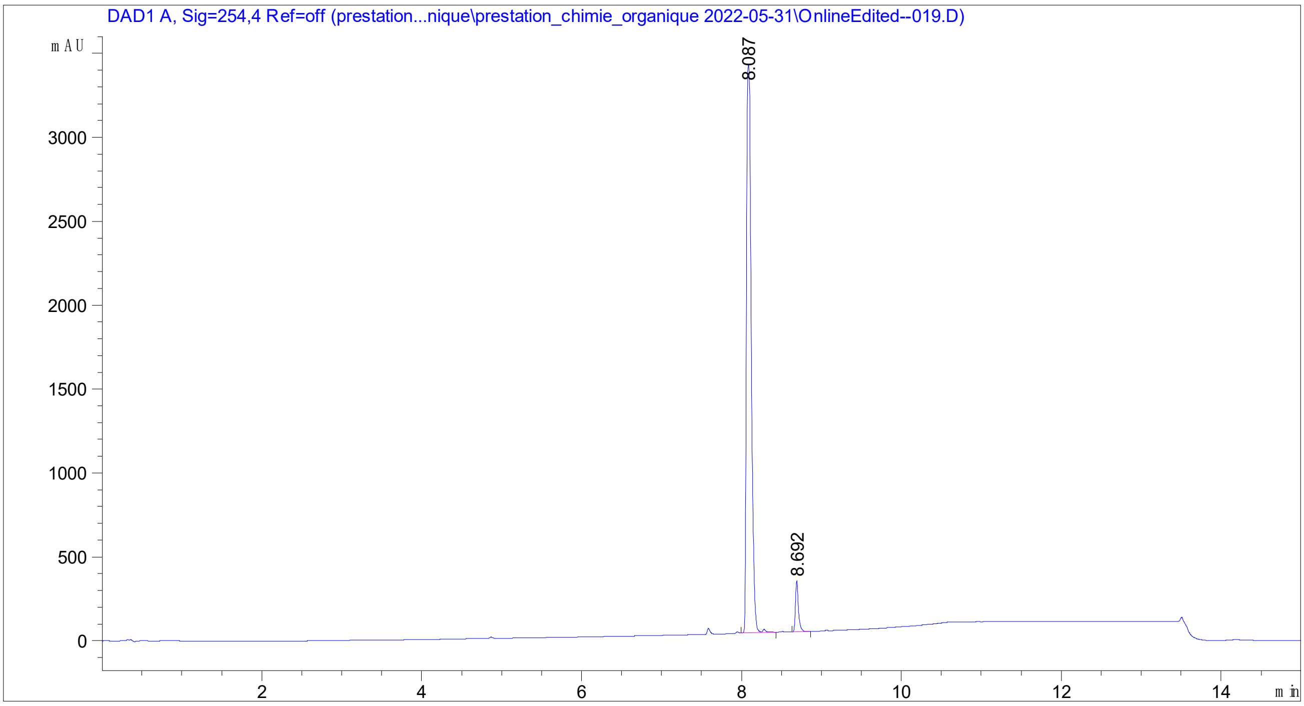

===
Area Percent Report
===

Sorted By : Signal
Multiplier : 1.0000
Dilution : 1.0000
Use Multiplier & Dilution Factor with ISTDs

Signal 1: DAD1 A, Sig=254,4 Ref=off

Peak #	RetTime [min]	Type	Width [min]	Area [mAU*s]	Height [mAU]	Area %
1	8.087	BV R	0.0660	1.42849e4	3385.43384	94.9498
2	8.692	BV R	0.0385	759.79028	300.77063	5.0502

Totals : 1.50447e4 3686.20447

===
*** End of Report ***

Compound 4b

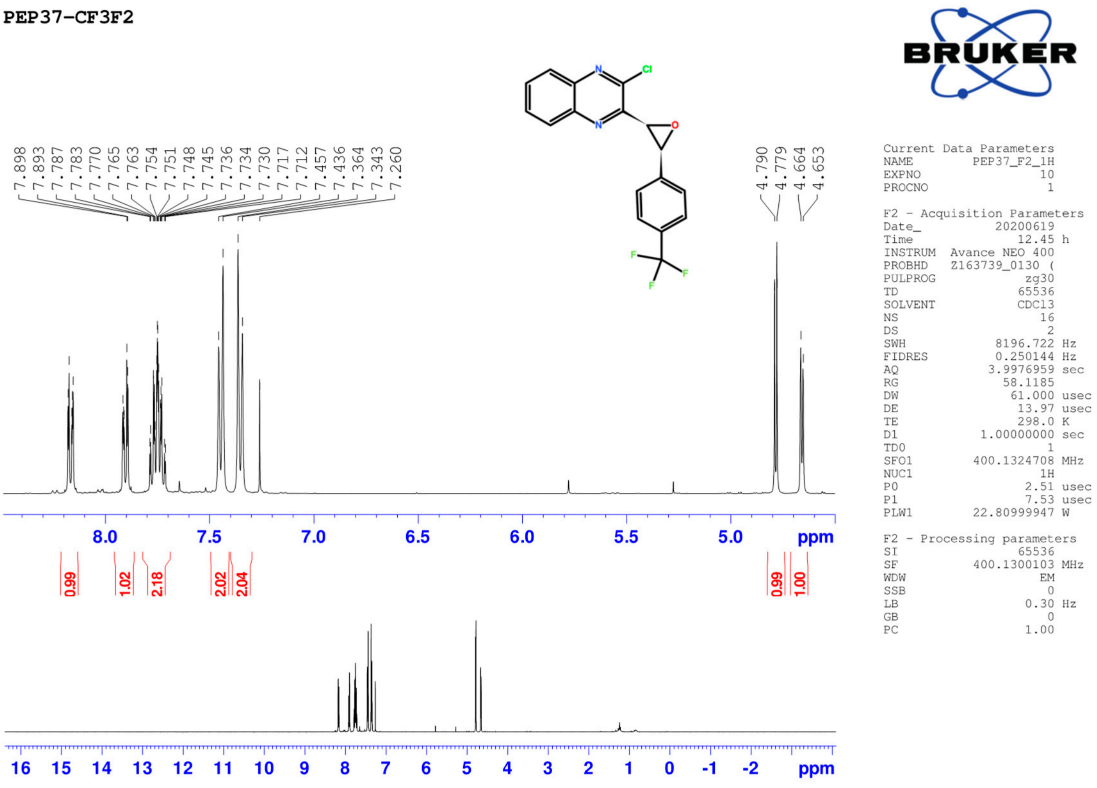
PEP37-CF3F2
7.898 7.893 7.787 7.783 7.770 7.765 7.763 7.754 7.751 7.748 7.745 7.736 7.734 7.730 7.717 7.712 7.457 7.436 7.364 7.343 7.260
4.790 4.779 4.664 4.653
N
Cl
O
F
8.0 7.5 7.0 6.5 6.0 5.5 5.0 ppm
0.99 1.02 2.18 2.02 2.04 0.99 1.00
16 15 14 13 12 11 10 9 8 7 6 5 4 3 2 1 0 -1 -2 ppm
Current Data Parameters
NAME PEP37_F2_1H
EXPNO 10
PROCNO 1
F2 - Acquisition Parameters
Date_ 20200619
Time 12.45 h
INSTRUM Avance NEO 400
PROBHD Z163739_0130 (
PULPROG zg30
TD 65536
SOLVENT CDCl3
NS 16
DS 2
SWH 8196.722 Hz
FIDRES 0.250144 Hz
AQ 3.9976959 sec
RG 58.1185
DW 61.000 usec
DE 13.97 usec
TE 298.0 K
D1 1.00000000 sec
TD0 1
SFO1 400.1324708 MHz
NUC1 1H
P0 2.51 usec
P1 7.53 usec
PLW1 22.80999947 W
F2 - Processing parameters
SI 65536
SF 400.1300103 MHz
WDW EM
SSB 0
LB 0.30 Hz
GB 0
PC 1.00

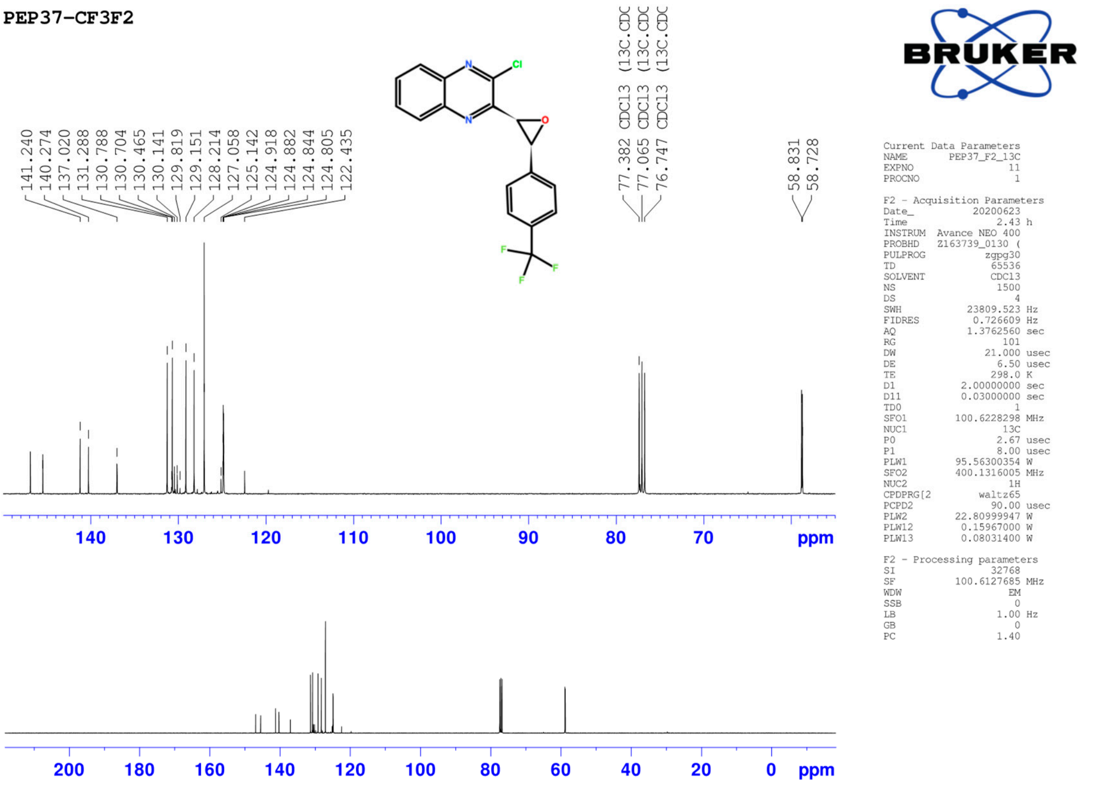
PEP37-CF3F2
141.240 140.274 137.020 131.288 130.788 130.704 130.465 130.141 129.819 129.151 128.214 127.058 125.142 124.918 124.882 124.844 124.805 122.435
77.382 CDCl3 (13C.CDC
77.065 CDCl3 (13C.CDC
76.747 CDCl3 (13C.CDC
58.831 58.728
N
Cl
O
F
140 130 120 110 100 90 80 70 ppm
200 180 160 140 120 100 80 60 40 20 0 ppm
Current Data Parameters
NAME PEP37_F2_13C
EXPNO 11
PROCNO 1
F2 - Acquisition Parameters
Date_ 20200623
Time 2.43 h
INSTRUM Avance NEO 400
PROBHD Z163739_0130 (
PULPROG zgpg30
TD 65536
SOLVENT CDCl3
NS 1500
DS 4
SWH 23809.523 Hz
FIDRES 0.726609 Hz
AQ 1.3762560 sec
RG 101
DW 21.000 usec
DE 6.50 usec
TE 298.0 K
D1 2.00000000 sec
D11 0.03000000 sec
TD0 1
SFO1 100.6228298 MHz
NUC1 13C
P0 2.67 usec
P1 8.00 usec
PLW1 95.56300354 W
SFO2 400.1316005 MHz
NUC2 1H
CPDPRG[2 waltz65
PCPD2 90.00 usec
PLW2 22.80999947 W
PLW12 0.15967000 W
PLW13 0.08031400 W
F2 - Processing parameters
SI 32768
SF 100.6127685 MHz
WDW EM
SSB 0
LB 1.00 Hz
GB 0
PC 1.40

```
Data File C:\Chem32\...rganique\prestation_chimie_organique 2022-05-31\OnlineEdited--020.D
Sample Name: PEP37F2

    =====================================================================
    Acq. Operator   : SYSTEM                          Seq. Line :  20
    Acq. Instrument : LC1290                           Location :   P1-C1
    Injection Date  : 31/5/2022 6:12:17 PM                  Inj :   1
                                                     Inj Volume : 1.000 µl
    Method          : C:\Chem32\1\Data\prestation_chimie_organique\prestation_chimie_organique
                      2022-05-31\prestation_chimie_org.M (Sequence Method)
    Last changed    : 31/5/2022 1:07:56 PM by SYSTEM
    Additional Info : Peak(s) manually integrated
```

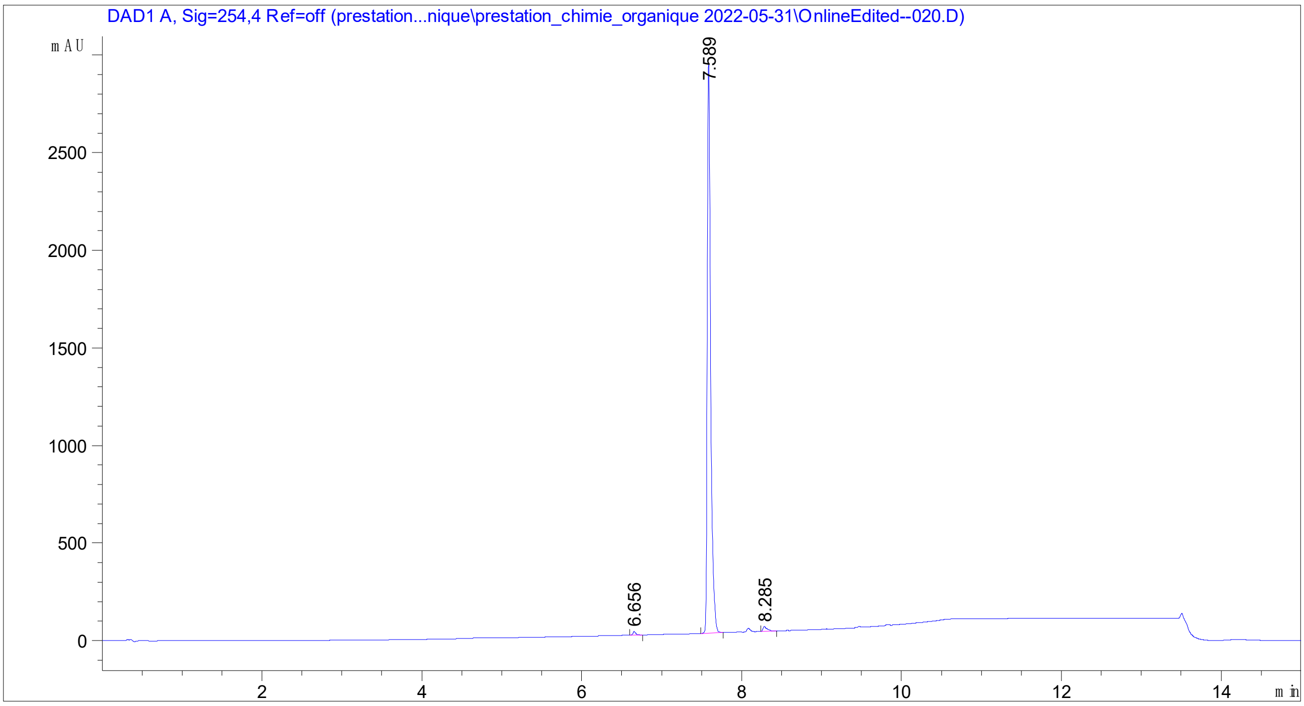


```
    =====================================================================
                             Area Percent Report
    =====================================================================

    Sorted By             :      Signal
    Multiplier            :      1.0000
    Dilution              :      1.0000
    Use Multiplier & Dilution Factor with ISTDs


    Signal 1: DAD1 A, Sig=254,4 Ref=off

    Peak RetTime Type  Width      Area       Height     Area
      #   [min]        [min]    [mAU*s]      [mAU]        %
    ----|-------|----|-------|----------|----------|--------|
       1   6.656 BB    0.0385   48.42534   18.51770   0.5551
       2   7.589 BB    0.0443 8580.26172 2918.09277  98.3610
       3   8.285 BV R  0.0511   94.55002   25.56572   1.0839

    Totals :                  8723.23708 2962.17620


    =====================================================================
                              *** End of Report ***
```

Compound 5a

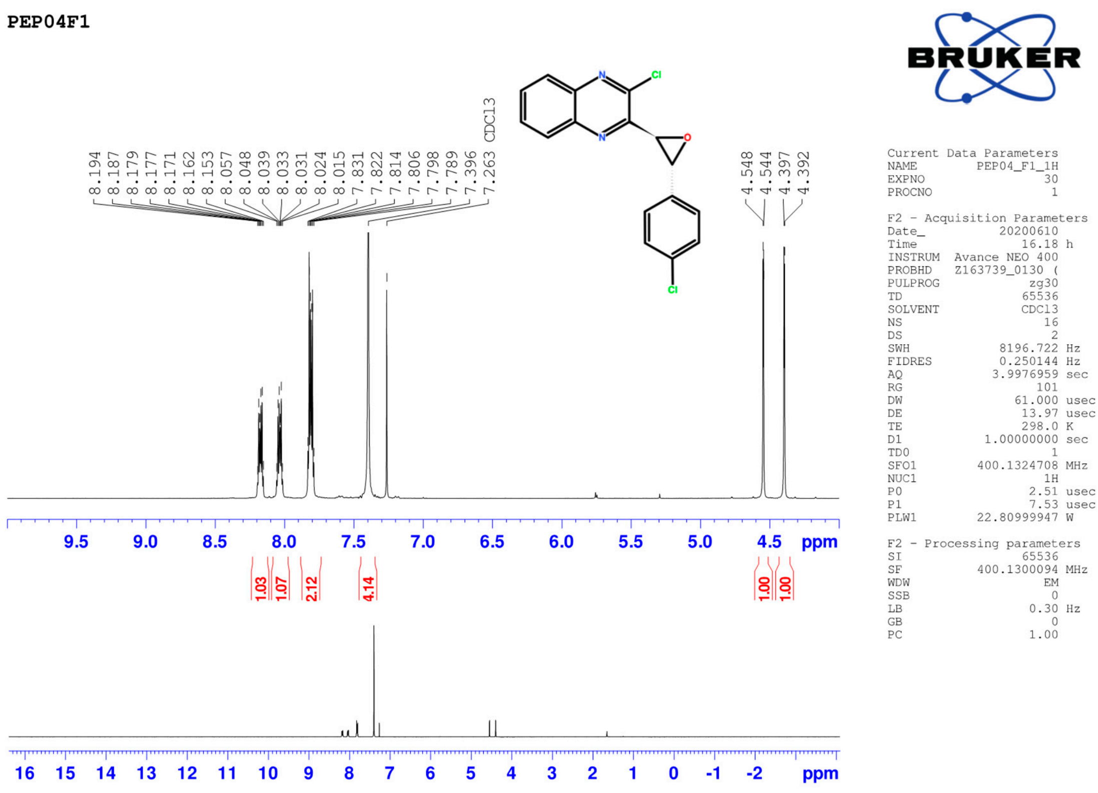

PEP04F1
8.194
8.187
8.179
8.177
8.171
8.162
8.153
8.057
8.048
8.039
8.033
8.031
8.024
8.015
7.831
7.822
7.814
7.806
7.798
7.789
7.396
7.263 CDCl3
4.548
4.544
4.397
4.392
N
Cl
O
Cl
Current Data Parameters
NAME PEP04_F1_1H
EXPNO 30
PROCNO 1
F2 - Acquisition Parameters
Date_ 20200610
Time 16.18 h
INSTRUM Avance NEO 400
PROBHD Z163739_0130 (
PULPROG zg30
TD 65536
SOLVENT CDCl3
NS 16
DS 2
SWH 8196.722 Hz
FIDRES 0.250144 Hz
AQ 3.9976959 sec
RG 101
DW 61.000 usec
DE 13.97 usec
TE 298.0 K
D1 1.00000000 sec
TD0 1
SFO1 400.1324708 MHz
NUC1 1H
P0 2.51 usec
P1 7.53 usec
PLW1 22.80999947 W
F2 - Processing parameters
SI 65536
SF 400.1300094 MHz
WDW EM
SSB 0
LB 0.30 Hz
GB 0
PC 1.00
9.5 9.0 8.5 8.0 7.5 7.0 6.5 6.0 5.5 5.0 4.5 ppm
1.03 1.07 2.12 4.14 1.00 1.00
16 15 14 13 12 11 10 9 8 7 6 5 4 3 2 1 0 -1 -2 ppm

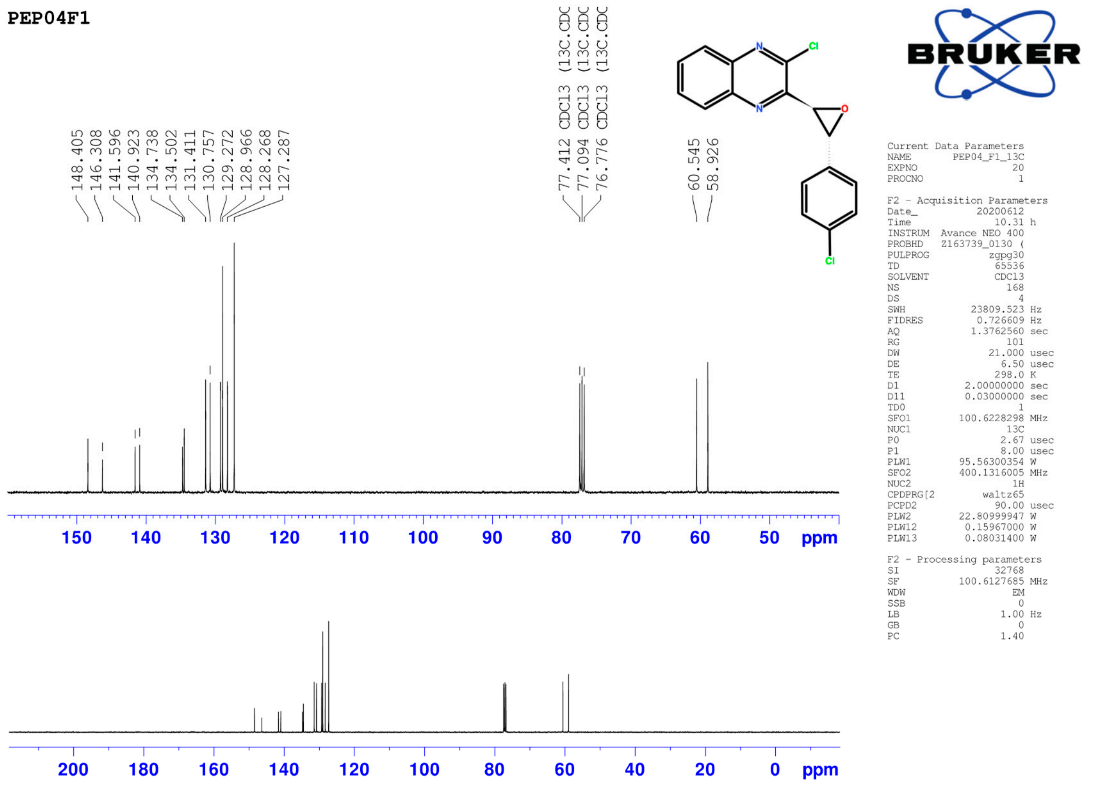

PEP04F1
148.405
146.308
141.596
140.923
134.738
134.502
131.411
130.757
129.272
128.966
128.268
127.287
77.412 CDCl3 (13C.CDC
77.094 CDCl3 (13C.CDC
76.776 CDCl3 (13C.CDC
60.545
58.926
N
Cl
O
Cl
Current Data Parameters
NAME PEP04_F1_13C
EXPNO 20
PROCNO 1
F2 - Acquisition Parameters
Date_ 20200612
Time 10.31 h
INSTRUM Avance NEO 400
PROBHD Z163739_0130 (
PULPROG zgpg30
TD 65536
SOLVENT CDCl3
NS 168
DS 4
SWH 23809.523 Hz
FIDRES 0.726609 Hz
AQ 1.3762560 sec
RG 101
DW 21.000 usec
DE 6.50 usec
TE 298.0 K
D1 2.00000000 sec
D11 0.03000000 sec
TD0 1
SFO1 100.6228298 MHz
NUC1 13C
P0 2.67 usec
P1 8.00 usec
PLW1 95.56300354 W
SFO2 400.1316005 MHz
NUC2 1H
CPDPRG[2 waltz65
PCPD2 90.00 usec
PLW2 22.80999947 W
PLW12 0.15967000 W
PLW13 0.08031400 W
F2 - Processing parameters
SI 32768
SF 100.6127685 MHz
WDW EM
SSB 0
LB 1.00 Hz
GB 0
PC 1.40
150 140 130 120 110 100 90 80 70 60 50 ppm
200 180 160 140 120 100 80 60 40 20 0 ppm

```
Data File C:\Chem32\...rganique\prestation_chimie_organique 2022-05-31\OnlineEdited--003.D
Sample Name: PEP04F1

    =====================================================================
    Acq. Operator   : SYSTEM                          Seq. Line :    3
    Acq. Instrument : LC1290                           Location :    P1-A2
    Injection Date  : 31/5/2022 1:40:30 PM                  Inj :    1
                                                     Inj Volume : 1.000 µl
    Method          : C:\Chem32\1\Data\prestation_chimie_organique\prestation_chimie_organique
                      2022-05-31\prestation_chimie_org.M (Sequence Method)
    Last changed    : 31/5/2022 1:07:56 PM by SYSTEM
    Additional Info : Peak(s) manually integrated
```

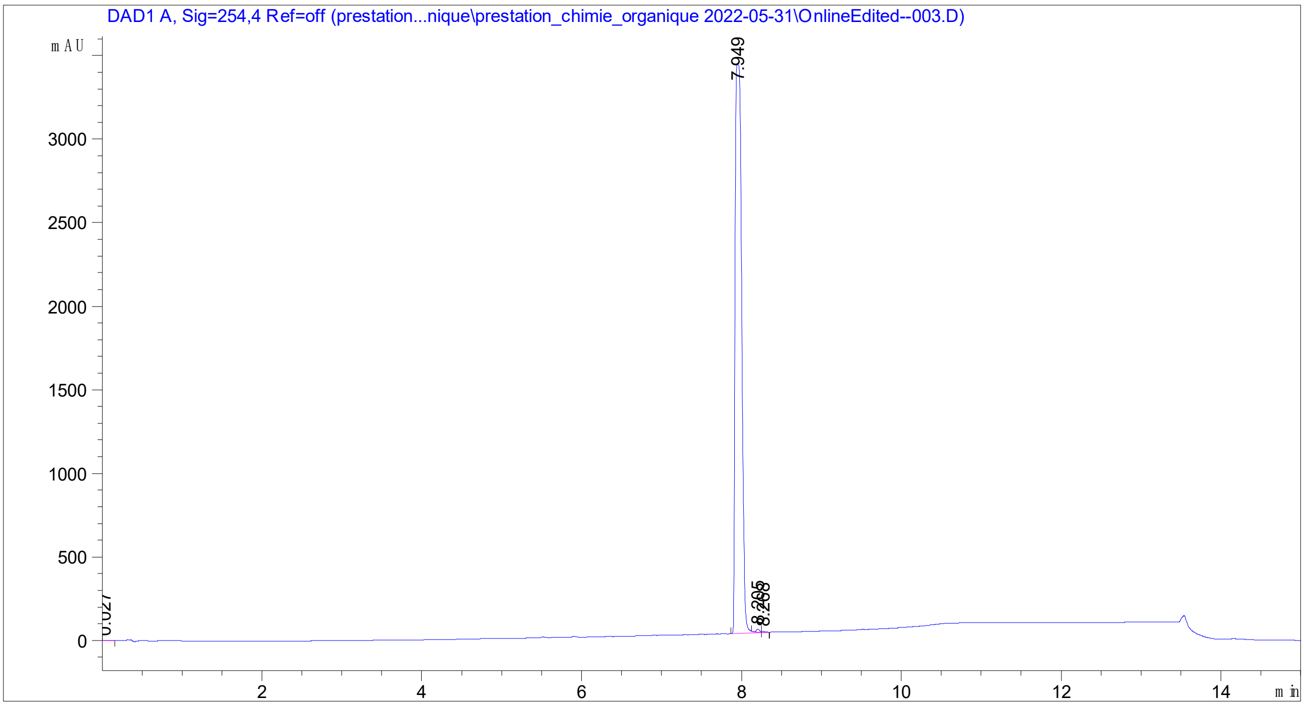


```
    =====================================================================
                             Area Percent Report
    =====================================================================

    Sorted By             :      Signal
    Multiplier            :      1.0000
    Dilution              :      1.0000
    Use Multiplier & Dilution Factor with ISTDs


    Signal 1: DAD1 A, Sig=254,4 Ref=off
```

Peak #	RetTime [min]	Type	Width [min]	Area [mAU*s]	Height [mAU]	Area %
1	0.027	BB	0.0446	5.80733	1.85338	0.0309
2	7.949	BV R	0.0731	1.87015e4	3402.69727	99.5685
3	8.205	VV E	0.0425	54.93636	19.11710	0.2925
4	8.268	VB E	0.0388	20.29902	7.45067	0.1081
Totals :				1.87826e4	3431.11842	

```
    =====================================================================
```

Compound 5b

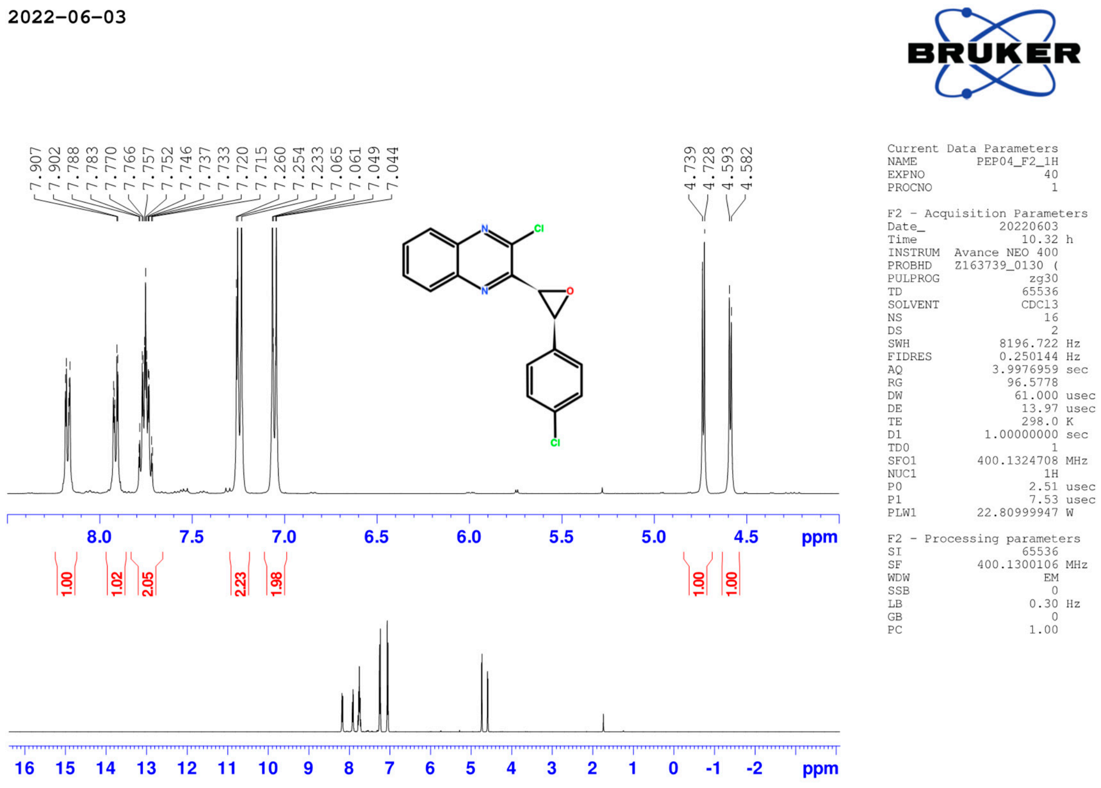
2022-06-03
7.907
7.902
7.788
7.783
7.770
7.766
7.757
7.752
7.746
7.737
7.733
7.720
7.715
7.260
7.254
7.233
7.065
7.061
7.049
7.044
4.739
4.728
4.593
4.582
N
N
Cl
O
Cl
1.00
1.02
2.05
2.23
1.98
1.00
1.00
8.0
7.5
7.0
6.5
6.0
5.5
5.0
4.5
ppm
16 15 14 13 12 11 10 9 8 7 6 5 4 3 2 1 0 -1 -2 ppm
BRUKER
Current Data Parameters
NAME PEP04_F2_1H
EXPNO 40
PROCNO 1
F2 - Acquisition Parameters
Date_ 20220603
Time 10.32 h
INSTRUM Avance NEO 400
PROBHD Z163739_0130 (
PULPROG zg30
TD 65536
SOLVENT CDCl3
NS 16
DS 2
SWH 8196.722 Hz
FIDRES 0.250144 Hz
AQ 3.9976959 sec
RG 96.5778
DW 61.000 usec
DE 13.97 usec
TE 298.0 K
D1 1.00000000 sec
TD0 1
SFO1 400.1324708 MHz
NUC1 1H
P0 2.51 usec
P1 7.53 usec
PLW1 22.80999947 W
F2 - Processing parameters
SI 65536
SF 400.1300106 MHz
WDW EM
SSB 0
LB 0.30 Hz
GB 0
PC 1.00

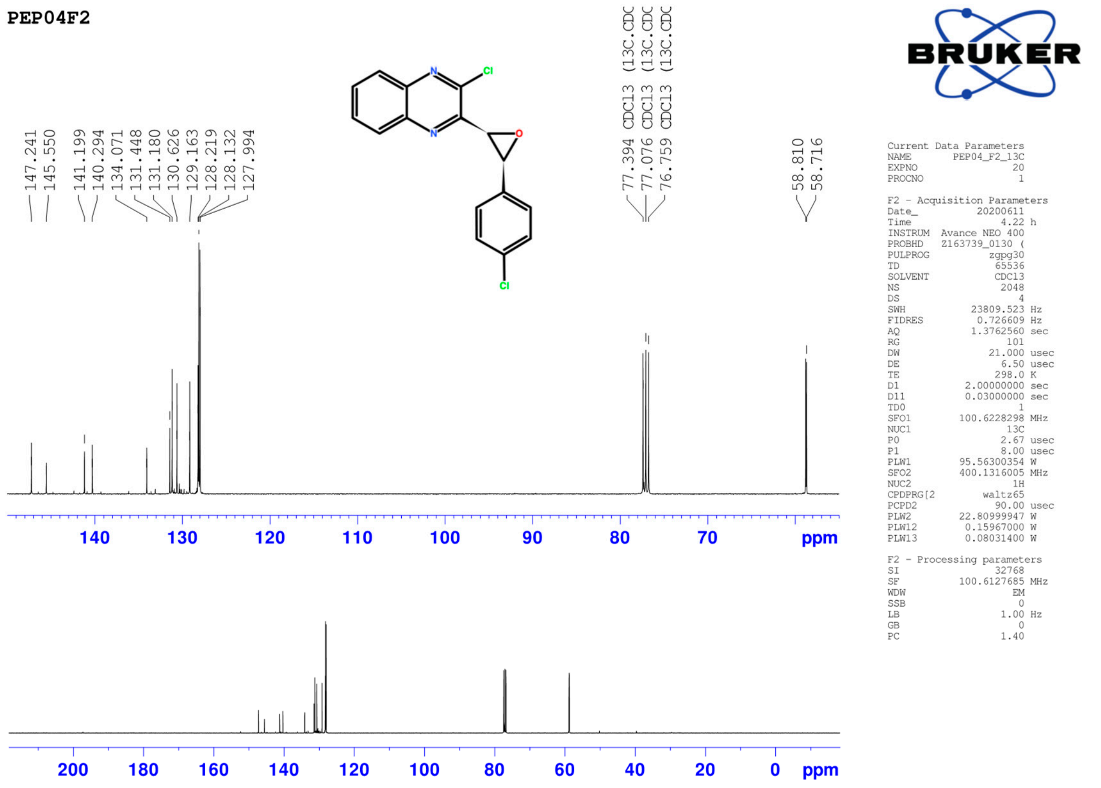
PEP04F2
147.241
145.550
141.199
140.294
134.071
131.448
131.180
130.626
129.163
128.219
128.132
127.994
77.394 CDCl3 (13C.CDC
77.076 CDCl3 (13C.CDC
76.759 CDCl3 (13C.CDC
58.810
58.716
N
N
Cl
O
Cl
140
130
120
110
100
90
80
70
ppm
200 180 160 140 120 100 80 60 40 20 0 ppm
BRUKER
Current Data Parameters
NAME PEP04_F2_13C
EXPNO 20
PROCNO 1
F2 - Acquisition Parameters
Date_ 20200611
Time 4.22 h
INSTRUM Avance NEO 400
PROBHD Z163739_0130 (
PULPROG zgpg30
TD 65536
SOLVENT CDCl3
NS 2048
DS 4
SWH 23809.523 Hz
FIDRES 0.726609 Hz
AQ 1.3762560 sec
RG 101
DW 21.000 usec
DE 6.50 usec
TE 298.0 K
D1 2.00000000 sec
D11 0.03000000 sec
TD0 1
SFO1 100.6228298 MHz
NUC1 13C
P0 2.67 usec
P1 8.00 usec
PLW1 95.56300354 W
SFO2 400.1316005 MHz
NUC2 1H
CPDPRG[2 waltz65
PCPD2 90.00 usec
PLW2 22.80999947 W
PLW12 0.15967000 W
PLW13 0.08031400 W
F2 - Processing parameters
SI 32768
SF 100.6127685 MHz
WDW EM
SSB 0
LB 1.00 Hz
GB 0
PC 1.40

Compound 6a

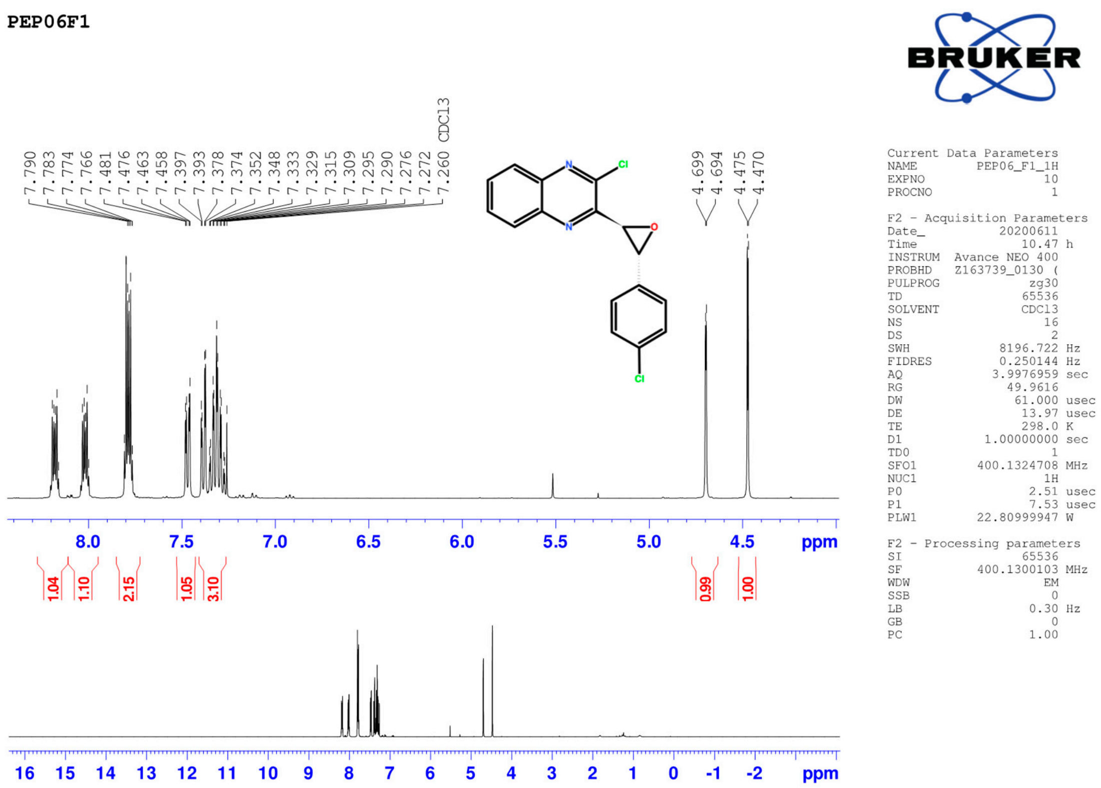
PEP06F1
7.790
7.783
7.774
7.766
7.481
7.476
7.463
7.458
7.397
7.393
7.378
7.374
7.352
7.348
7.333
7.329
7.315
7.309
7.295
7.290
7.276
7.272
7.260 CDCl3
4.699
4.694
4.475
4.470
N
N
Cl
O
Cl
1.04
1.10
2.15
1.05
3.10
0.99
1.00
ppm
BRUKER
Current Data Parameters
NAME PEP06_F1_1H
EXPNO 10
PROCNO 1
F2 - Acquisition Parameters
Date_ 20200611
Time 10.47 h
INSTRUM Avance NEO 400
PROBHD Z163739_0130 (
PULPROG zg30
TD 65536
SOLVENT CDCl3
NS 16
DS 2
SWH 8196.722 Hz
FIDRES 0.250144 Hz
AQ 3.9976959 sec
RG 49.9616
DW 61.000 usec
DE 13.97 usec
TE 298.0 K
D1 1.00000000 sec
TD0 1
SFO1 400.1324708 MHz
NUC1 1H
P0 2.51 usec
P1 7.53 usec
PLW1 22.80999947 W
F2 - Processing parameters
SI 65536
SF 400.1300103 MHz
WDW EM
SSB 0
LB 0.30 Hz
GB 0
PC 1.00

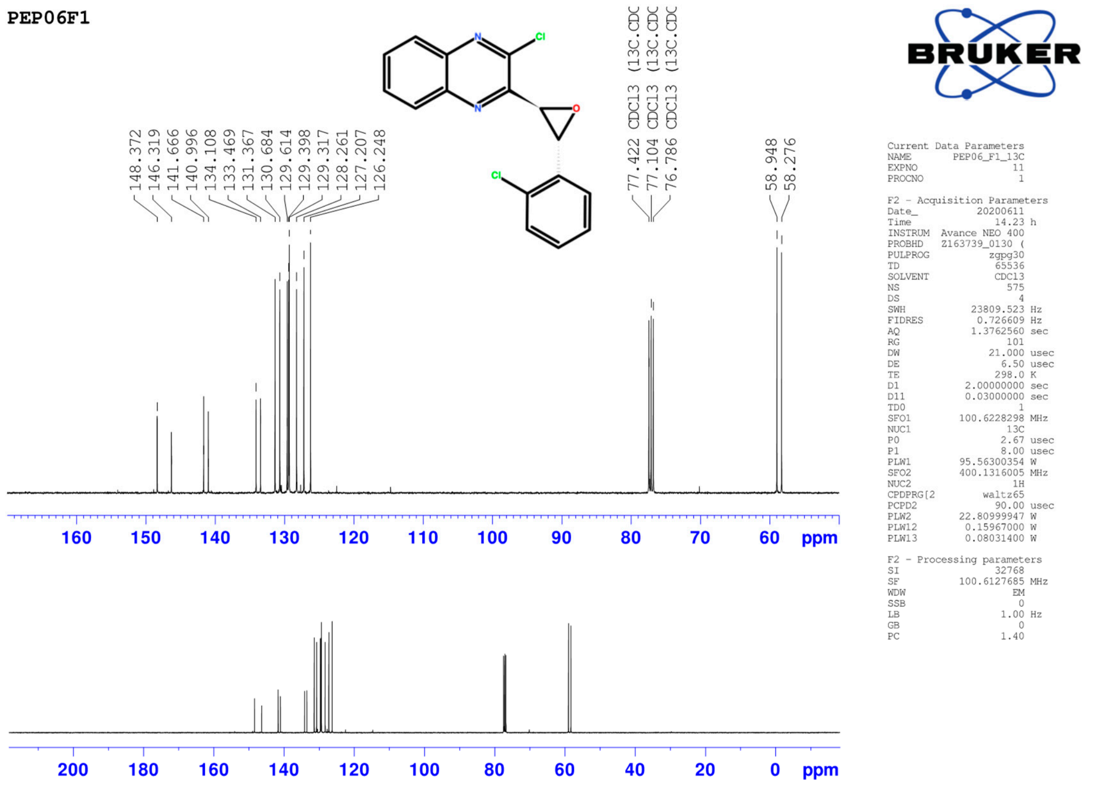
PEP06F1
148.372
146.319
141.666
140.996
134.108
133.469
131.367
130.684
129.614
129.398
129.317
128.261
127.207
126.248
77.422 CDCl3 (13C.CDC
77.104 CDCl3 (13C.CDC
76.786 CDCl3 (13C.CDC
58.948
58.276
N
N
Cl
O
Cl
ppm
BRUKER
Current Data Parameters
NAME PEP06_F1_13C
EXPNO 11
PROCNO 1
F2 - Acquisition Parameters
Date_ 20200611
Time 14.23 h
INSTRUM Avance NEO 400
PROBHD Z163739_0130 (
PULPROG zgpg30
TD 65536
SOLVENT CDCl3
NS 575
DS 4
SWH 23809.523 Hz
FIDRES 0.726609 Hz
AQ 1.3762560 sec
RG 101
DW 21.000 usec
DE 6.50 usec
TE 298.0 K
D1 2.00000000 sec
D11 0.03000000 sec
TD0 1
SFO1 100.6228298 MHz
NUC1 13C
P0 2.67 usec
P1 8.00 usec
PLW1 95.56300354 W
SFO2 400.1316005 MHz
NUC2 1H
CPDPRG[2 waltz65
PCPD2 90.00 usec
PLW2 22.80999947 W
PLW12 0.15967000 W
PLW13 0.08031400 W
F2 - Processing parameters
SI 32768
SF 100.6127685 MHz
WDW EM
SSB 0
LB 1.00 Hz
GB 0
PC 1.40

Data File C:\Chem32\...rganique\prestation_chimie_organique 2022-05-31\OnlineEdited--007.D
Sample Name: PEP06F1

===

Acq. Operator : SYSTEM Seq. Line : 7
Acq. Instrument : LC1290 Location : P1-A6
Injection Date : 31/5/2022 2:43:40 PM Inj : 1
Inj Volume : 1.000 µl
Method : C:\Chem32\1\Data\prestation_chimie_organique\prestation_chimie_organique 2022-05-31\prestation_chimie_org.M (Sequence Method)
Last changed : 31/5/2022 1:07:56 PM by SYSTEM
Additional Info : Peak(s) manually integrated

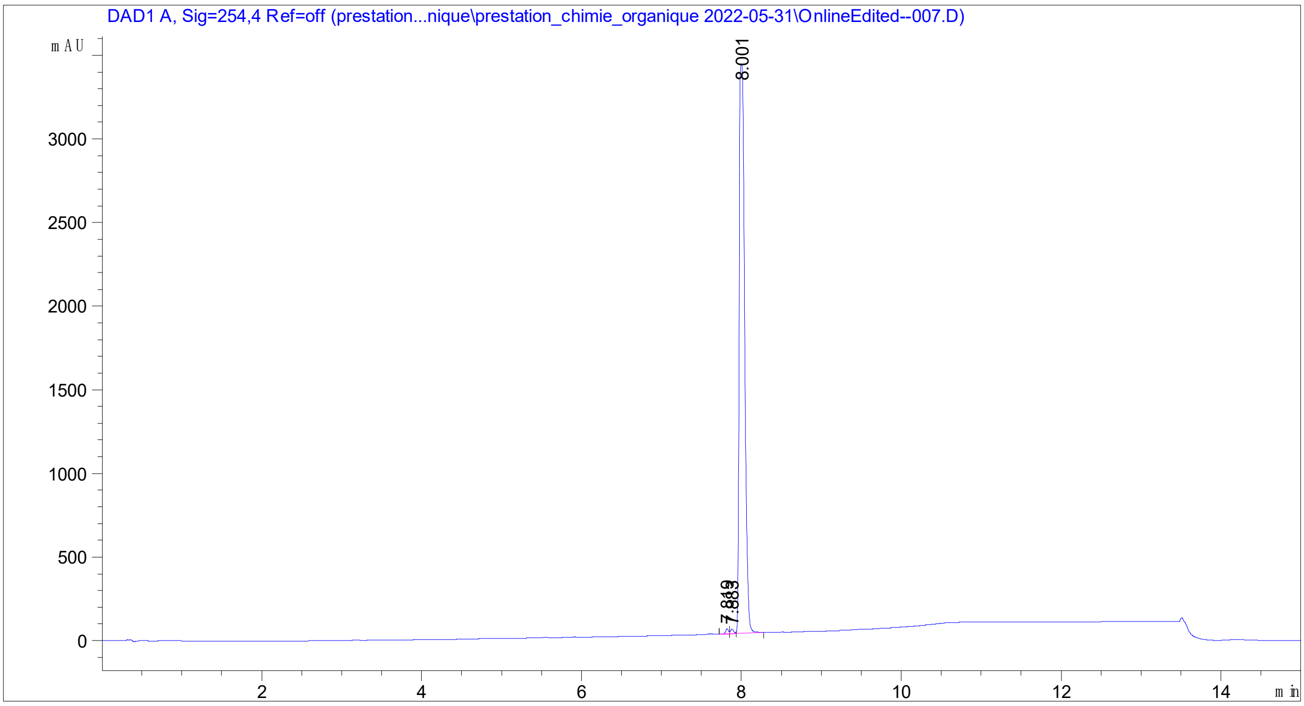

===

Area Percent Report

===

Sorted By : Signal
Multiplier : 1.0000
Dilution : 1.0000
Use Multiplier & Dilution Factor with ISTDs

Signal 1: DAD1 A, Sig=254,4 Ref=off

Peak #	RetTime [min]	Type	Width [min]	Area [mAU*s]	Height [mAU]	Area %
1	7.819	BV E	0.0357	79.28677	33.43105	0.4858
2	7.883	VV E	0.0394	75.01073	28.80846	0.4596
3	8.001	VB R	0.0755	1.61668e4	3400.07349	99.0546

Totals : 1.63211e4 3462.31299

===

*** End of Report ***

Compound 7a

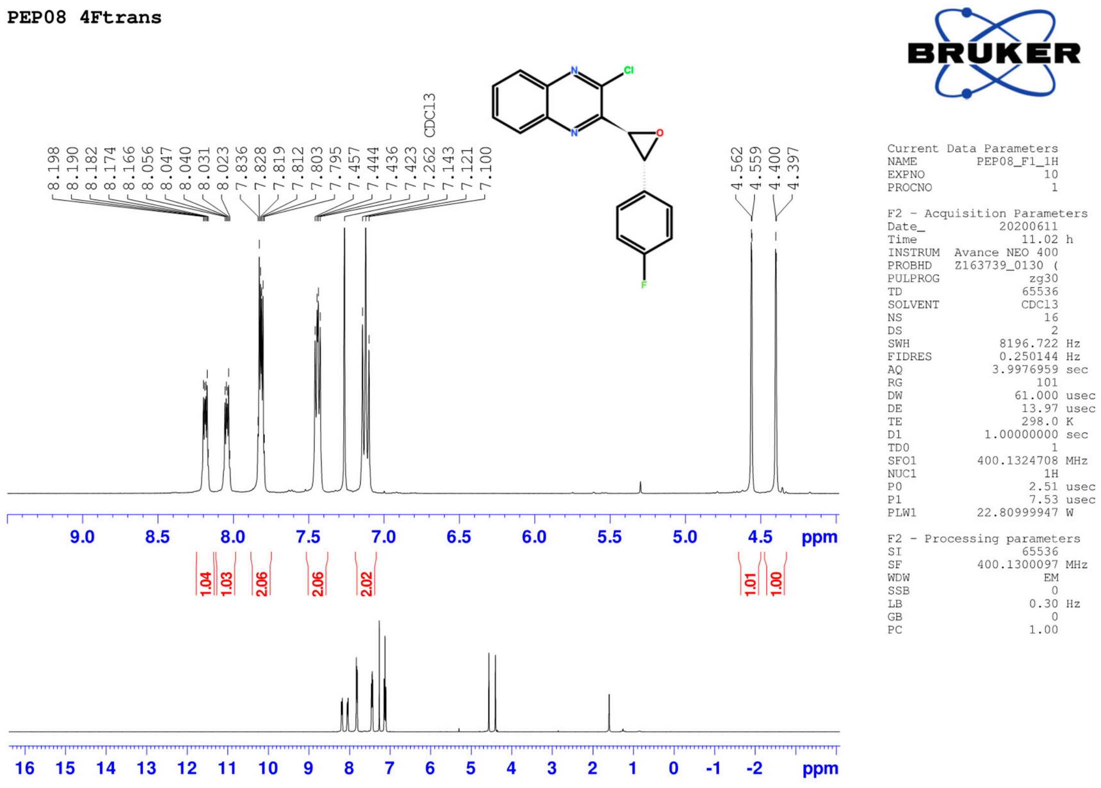
PEP08 4Ftrans
8.198
8.190
8.182
8.174
8.166
8.056
8.047
8.040
8.031
8.023
7.836
7.828
7.819
7.812
7.803
7.795
7.457
7.444
7.436
7.423
7.262 CDCl3
7.143
7.121
7.100
4.562
4.559
4.400
4.397
N
Cl
O
F
9.0 8.5 8.0 7.5 7.0 6.5 6.0 5.5 5.0 4.5 ppm
1.04 1.03 2.06 2.06 2.02 1.01 1.00
16 15 14 13 12 11 10 9 8 7 6 5 4 3 2 1 0 -1 -2 ppm
BRUKER
Current Data Parameters
NAME PEP08_F1_1H
EXPNO 10
PROCNO 1
F2 - Acquisition Parameters
Date_ 20200611
Time 11.02 h
INSTRUM Avance NEO 400
PROBHD Z163739_0130 (
PULPROG zg30
TD 65536
SOLVENT CDCl3
NS 16
DS 2
SWH 8196.722 Hz
FIDRES 0.250144 Hz
AQ 3.9976959 sec
RG 101
DW 61.000 usec
DE 13.97 usec
TE 298.0 K
D1 1.00000000 sec
TD0 1
SFO1 400.1324708 MHz
NUC1 1H
P0 2.51 usec
P1 7.53 usec
PLW1 22.80999947 W
F2 - Processing parameters
SI 65536
SF 400.1300097 MHz
WDW EM
SSB 0
LB 0.30 Hz
GB 0
PC 1.00

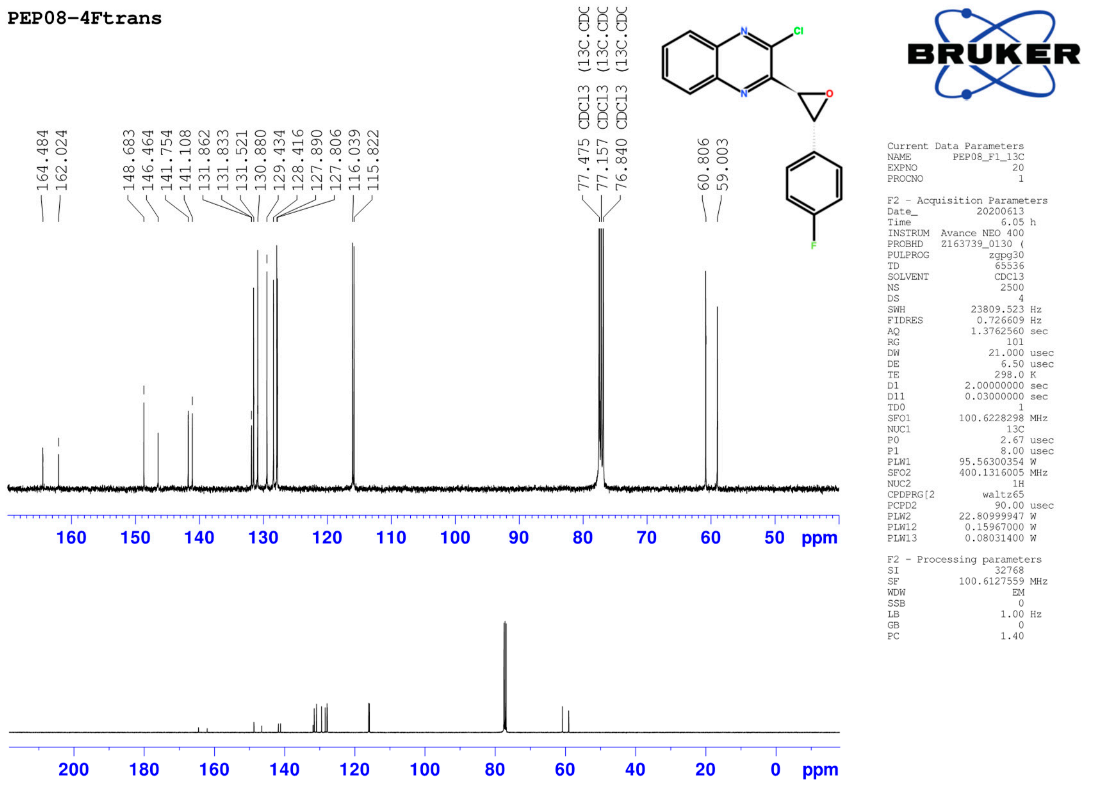
PEP08-4Ftrans
164.484
162.024
148.683
146.464
141.754
141.108
131.862
131.833
131.521
130.880
129.434
128.416
127.890
127.806
116.039
115.822
77.475 CDCl3 (13C.CDC
77.157 CDCl3 (13C.CDC
76.840 CDCl3 (13C.CDC
60.806
59.003
N
Cl
O
F
160 150 140 130 120 110 100 90 80 70 60 50 ppm
200 180 160 140 120 100 80 60 40 20 0 ppm
BRUKER
Current Data Parameters
NAME PEP08_F1_13C
EXPNO 20
PROCNO 1
F2 - Acquisition Parameters
Date_ 20200613
Time 6.05 h
INSTRUM Avance NEO 400
PROBHD Z163739_0130 (
PULPROG zgpg30
TD 65536
SOLVENT CDCl3
NS 2500
DS 4
SWH 23809.523 Hz
FIDRES 0.726609 Hz
AQ 1.3762560 sec
RG 101
DW 21.000 usec
DE 6.50 usec
TE 298.0 K
D1 2.00000000 sec
D11 0.03000000 sec
TD0 1
SFO1 100.6228298 MHz
NUC1 13C
P0 2.67 usec
P1 8.00 usec
PLW1 95.56300354 W
SFO2 400.1316005 MHz
NUC2 1H
CPDPRG[2 waltz65
PCPD2 90.00 usec
PLW2 22.80999947 W
PLW12 0.15967000 W
PLW13 0.08031400 W
F2 - Processing parameters
SI 32768
SF 100.6127559 MHz
WDW EM
SSB 0
LB 1.00 Hz
GB 0
PC 1.40

Compound 7b

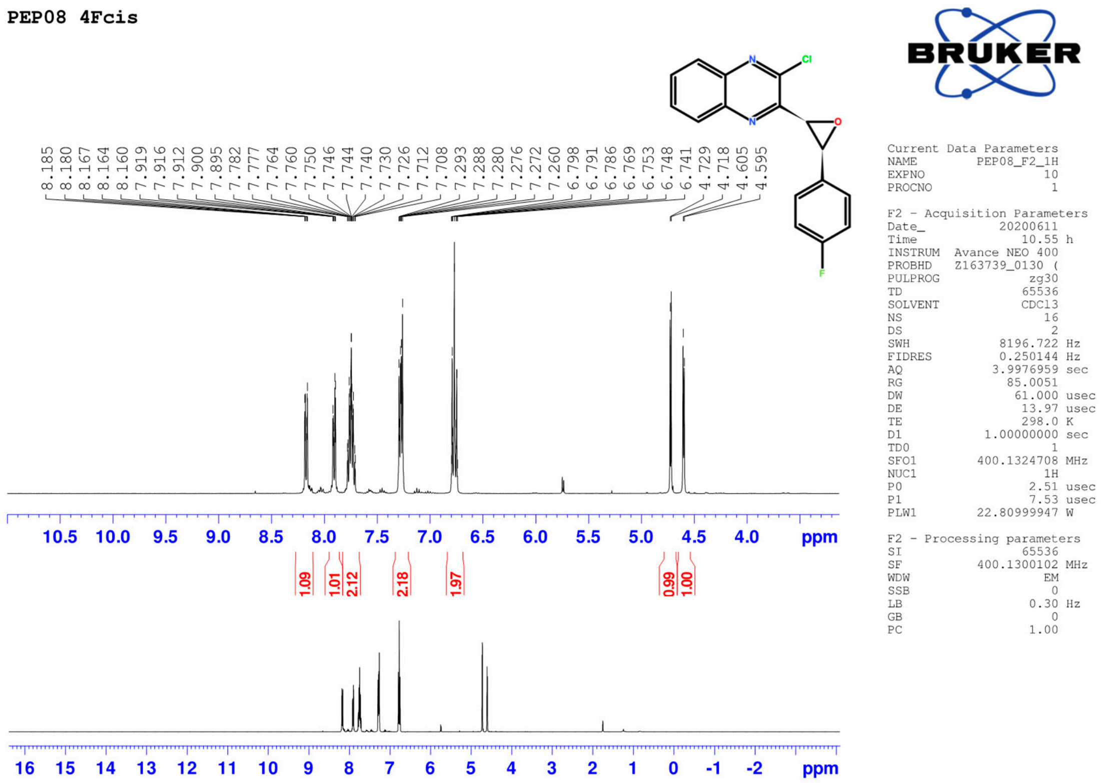
PEP08 4Fcis
8.185
8.180
8.167
8.164
8.160
7.919
7.916
7.912
7.900
7.895
7.782
7.777
7.764
7.760
7.750
7.746
7.744
7.740
7.730
7.726
7.712
7.708
7.293
7.288
7.280
7.276
7.272
7.260
6.798
6.791
6.786
6.769
6.753
6.748
6.741
4.729
4.718
4.605
4.595
N
Cl
O
F
1.09
1.01
2.12
2.18
1.97
0.99
1.00
10.5 10.0 9.5 9.0 8.5 8.0 7.5 7.0 6.5 6.0 5.5 5.0 4.5 4.0 ppm
16 15 14 13 12 11 10 9 8 7 6 5 4 3 2 1 0 -1 -2 ppm
Current Data Parameters
NAME PEP08_F2_1H
EXPNO 10
PROCNO 1
F2 - Acquisition Parameters
Date_ 20200611
Time 10.55 h
INSTRUM Avance NEO 400
PROBHD Z163739_0130 (
PULPROG zg30
TD 65536
SOLVENT CDCl3
NS 16
DS 2
SWH 8196.722 Hz
FIDRES 0.250144 Hz
AQ 3.9976959 sec
RG 85.0051
DW 61.000 usec
DE 13.97 usec
TE 298.0 K
D1 1.00000000 sec
TD0 1
SFO1 400.1324708 MHz
NUC1 1H
P0 2.51 usec
P1 7.53 usec
PLW1 22.80999947 W
F2 - Processing parameters
SI 65536
SF 400.1300102 MHz
WDW EM
SSB 0
LB 0.30 Hz
GB 0
PC 1.00

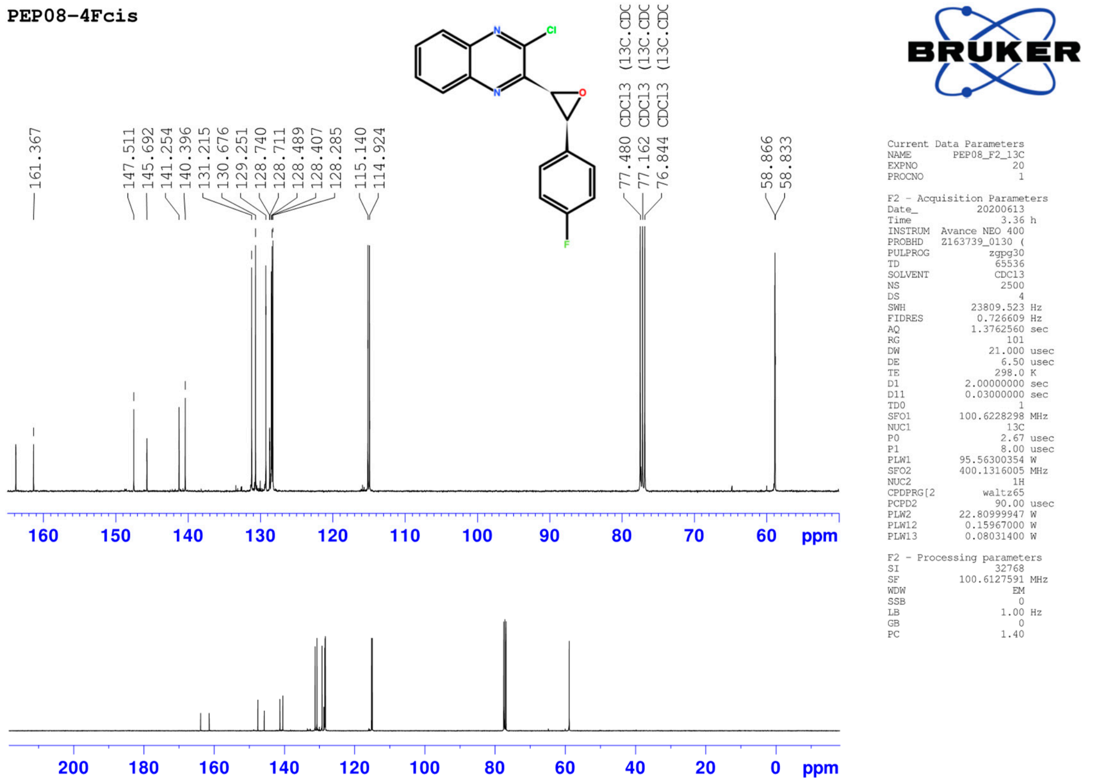
PEP08-4Fcis
161.367
147.511
145.692
141.254
140.396
131.215
130.676
129.251
128.740
128.711
128.489
128.407
128.285
115.140
114.924
77.480 CDCl3 (13C.CDC
77.162 CDCl3 (13C.CDC
76.844 CDCl3 (13C.CDC
58.866
58.833
N
Cl
O
F
160 150 140 130 120 110 100 90 80 70 60 ppm
200 180 160 140 120 100 80 60 40 20 0 ppm
Current Data Parameters
NAME PEP08_F2_13C
EXPNO 20
PROCNO 1
F2 - Acquisition Parameters
Date_ 20200613
Time 3.36 h
INSTRUM Avance NEO 400
PROBHD Z163739_0130 (
PULPROG zgpg30
TD 65536
SOLVENT CDCl3
NS 2500
DS 4
SWH 23809.523 Hz
FIDRES 0.726609 Hz
AQ 1.3762560 sec
RG 101
DW 21.000 usec
DE 6.50 usec
TE 298.0 K
D1 2.00000000 sec
D11 0.03000000 sec
TD0 1
SFO1 100.6228298 MHz
NUC1 13C
P0 2.67 usec
P1 8.00 usec
PLW1 95.56300354 W
SFO2 400.1316005 MHz
NUC2 1H
CPDPRG[2 waltz65
PCPD2 90.00 usec
PLW2 22.80999947 W
PLW12 0.15967000 W
PLW13 0.08031400 W
F2 - Processing parameters
SI 32768
SF 100.6127591 MHz
WDW EM
SSB 0
LB 1.00 Hz
GB 0
PC 1.40

Data File C:\Chem32\...rganique\prestation_chimie_organique 2022-05-31\OnlineEdited--011.D
Sample Name: PEP08F2

===

Acq. Operator : SYSTEM Seq. Line : 11
Acq. Instrument : LC1290 Location : P1-B1
Injection Date : 31/5/2022 3:46:47 PM Inj : 1
Inj Volume : 1.000 µl
Method : C:\Chem32\1\Data\prestation_chimie_organique\prestation_chimie_organique 2022-05-31\prestation_chimie_org.M (Sequence Method)
Last changed : 31/5/2022 1:07:56 PM by SYSTEM
Additional Info : Peak(s) manually integrated

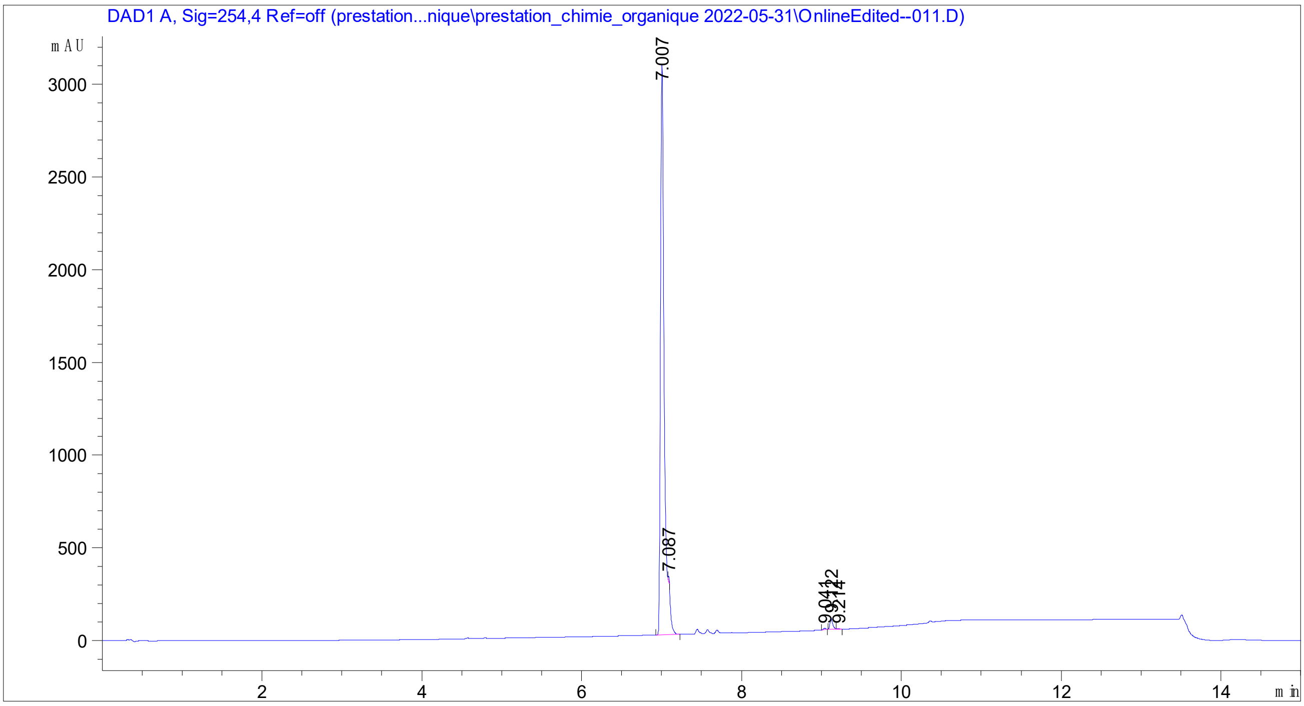

===

Area Percent Report

===

Sorted By : Signal
Multiplier : 1.0000
Dilution : 1.0000
Use Multiplier & Dilution Factor with ISTDs

Signal 1: DAD1 A, Sig=254,4 Ref=off

Peak #	RetTime [min]	Type	Width [min]	Area [mAU*s]	Height [mAU]	Area %
1	7.007	BV R	0.0486	9923.04688	3075.07031	98.0261
2	7.087	VB E	0.0170	19.77497	23.11082	0.1953
3	9.041	BB	0.0340	19.14015	8.91607	0.1891
4	9.122	BV R	0.0365	155.43683	63.62344	1.5355
5	9.214	VB E	0.0297	5.46008	2.80016	0.0539

Totals : 1.01229e4 3173.52080

Compound 8a

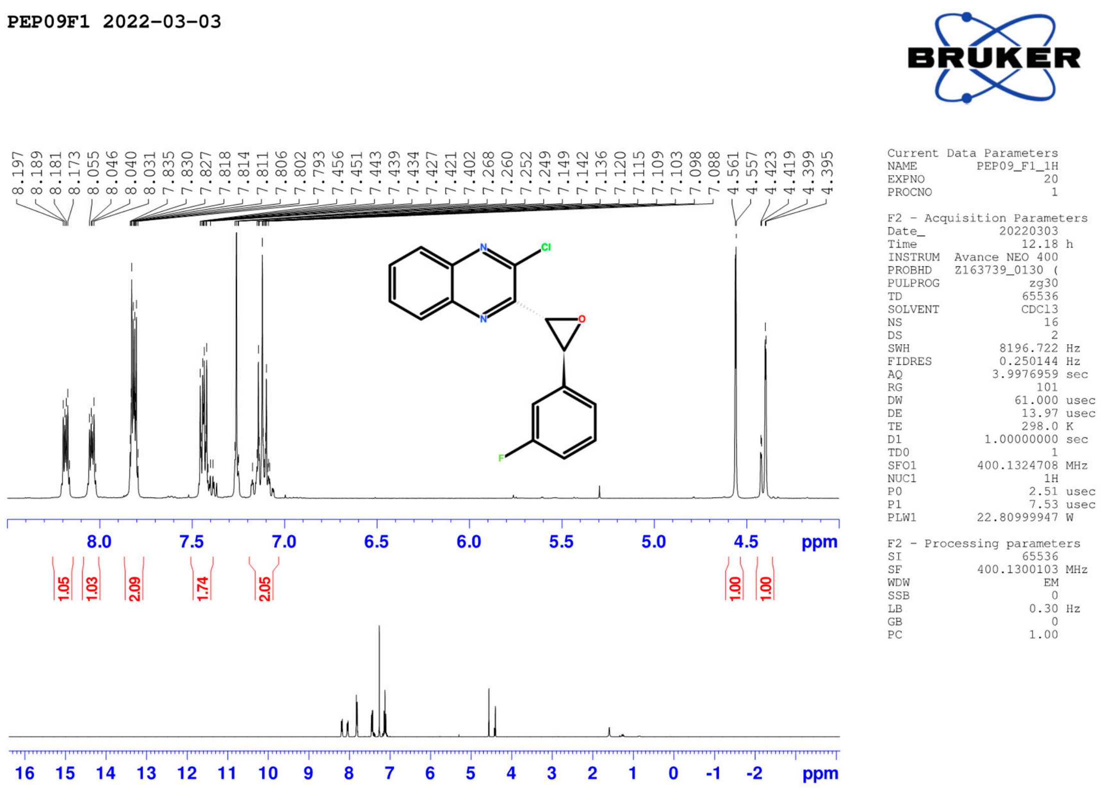
PEP09F1 2022-03-03
BRUKER
8.197 8.189 8.181 8.173 8.055 8.046 8.040 8.031 7.835 7.830 7.827 7.818 7.814 7.811 7.806 7.802 7.793 7.456 7.451 7.443 7.439 7.434 7.427 7.421 7.402 7.268 7.260 7.252 7.249 7.149 7.142 7.136 7.120 7.115 7.109 7.103 7.098 7.088 4.561 4.557 4.423 4.419 4.399 4.395
N
Cl
N
O
F
8.0 7.5 7.0 6.5 6.0 5.5 5.0 4.5 ppm
1.05 1.03 2.09 1.74 2.05 1.00 1.00
16 15 14 13 12 11 10 9 8 7 6 5 4 3 2 1 0 -1 -2 ppm
Current Data Parameters
NAME PEP09_F1_1H
EXPNO 20
PROCNO 1
F2 - Acquisition Parameters
Date_ 20220303
Time 12.18 h
INSTRUM Avance NEO 400
PROBHD Z163739_0130 (
PULPROG zg30
TD 65536
SOLVENT CDCl3
NS 16
DS 2
SWH 8196.722 Hz
FIDRES 0.250144 Hz
AQ 3.9976959 sec
RG 101
DW 61.000 usec
DE 13.97 usec
TE 298.0 K
D1 1.00000000 sec
TD0 1
SFO1 400.1324708 MHz
NUC1 1H
P0 2.51 usec
P1 7.53 usec
PLW1 22.80999947 W
F2 - Processing parameters
SI 65536
SF 400.1300103 MHz
WDW EM
SSB 0
LB 0.30 Hz
GB 0
PC 1.00

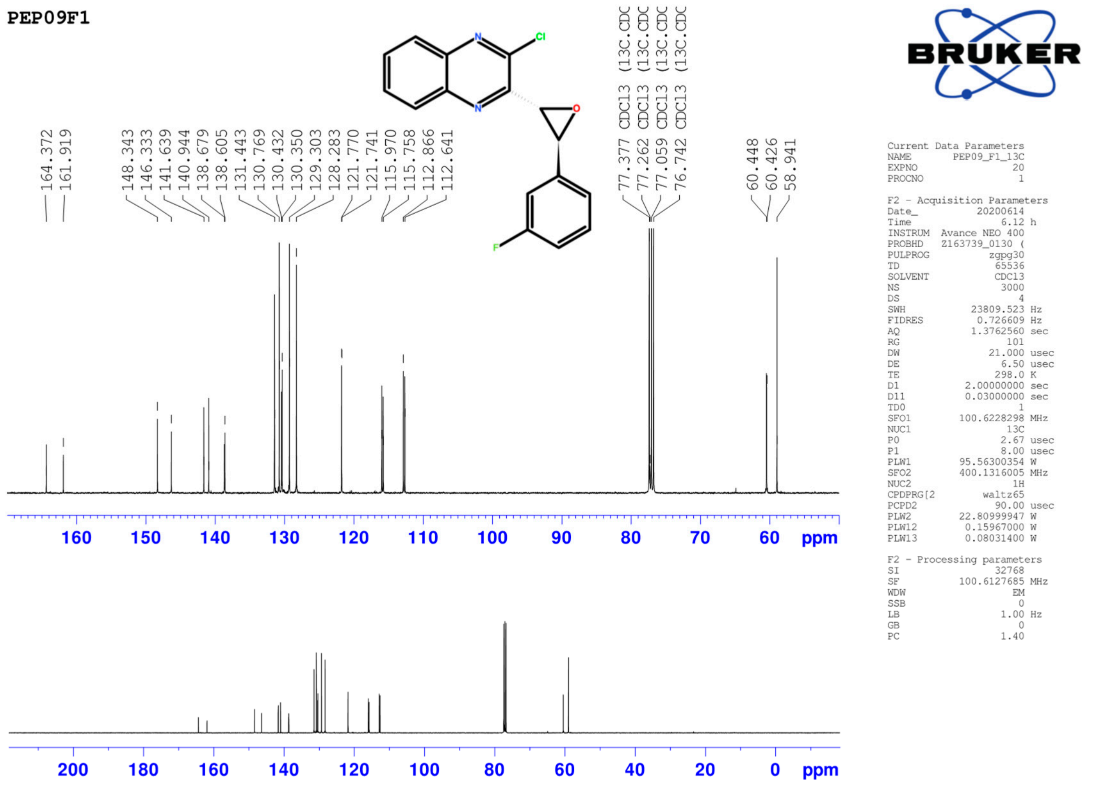
PEP09F1
BRUKER
164.372 161.919 148.343 146.333 141.639 140.944 138.679 138.605 131.443 130.769 130.432 130.350 129.303 128.283 121.770 121.741 115.970 115.758 112.866 112.641
77.377 CDCl3 (13C.CDC
77.262 CDCl3 (13C.CDC
77.059 CDCl3 (13C.CDC
76.742 CDCl3 (13C.CDC
60.448 60.426 58.941
N
Cl
N
O
F
160 150 140 130 120 110 100 90 80 70 60 ppm
200 180 160 140 120 100 80 60 40 20 0 ppm
Current Data Parameters
NAME PEP09_F1_13C
EXPNO 20
PROCNO 1
F2 - Acquisition Parameters
Date_ 20200614
Time 6.12 h
INSTRUM Avance NEO 400
PROBHD Z163739_0130 (
PULPROG zgpg30
TD 65536
SOLVENT CDCl3
NS 3000
DS 4
SWH 23809.523 Hz
FIDRES 0.726609 Hz
AQ 1.3762560 sec
RG 101
DW 21.000 usec
DE 6.50 usec
TE 298.0 K
D1 2.00000000 sec
D11 0.03000000 sec
TD0 1
SFO1 100.6228298 MHz
NUC1 13C
P0 2.67 usec
P1 8.00 usec
PLW1 95.56300354 W
SFO2 400.1316005 MHz
NUC2 1H
CPDPRG[2 waltz65
PCPD2 90.00 usec
PLW2 22.80999947 W
PLW12 0.15967000 W
PLW13 0.08031400 W
F2 - Processing parameters
SI 32768
SF 100.6127685 MHz
WDW EM
SSB 0
LB 1.00 Hz
GB 0
PC 1.40

```
Data File C:\Chem32\...rganique\prestation_chimie_organique 2022-05-31\OnlineEdited--012.D
Sample Name: PEP09F1

    =====================================================================
    Acq. Operator   : SYSTEM                          Seq. Line :  12
    Acq. Instrument : LC1290                           Location :   P1-B2
    Injection Date  : 31/5/2022 4:02:45 PM                  Inj :   1
                                                     Inj Volume : 1.000 µl
    Method          : C:\Chem32\1\Data\prestation_chimie_organique\prestation_chimie_organique
                      2022-05-31\prestation_chimie_org.M (Sequence Method)
    Last changed    : 31/5/2022 1:07:56 PM by SYSTEM
    Additional Info : Peak(s) manually integrated
```

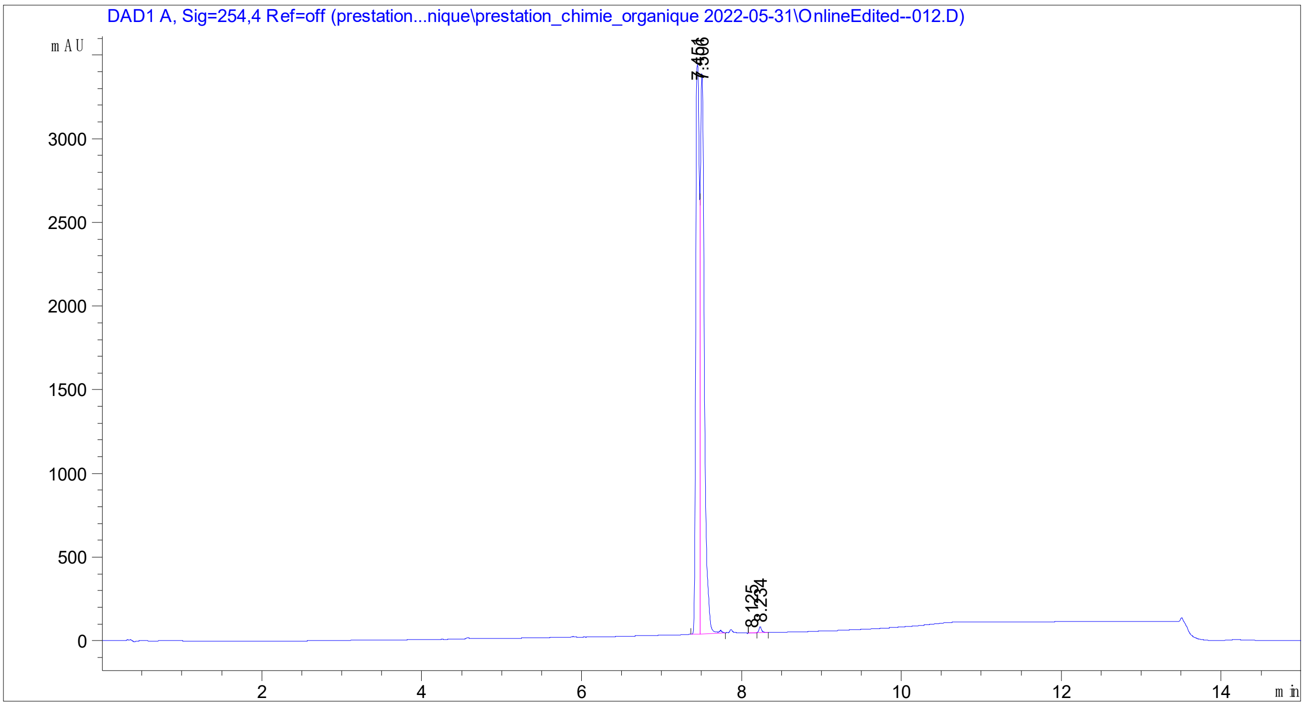


```
    =====================================================================
                             Area Percent Report
    =====================================================================

    Sorted By             :      Signal
    Multiplier            :      1.0000
    Dilution              :      1.0000
    Use Multiplier & Dilution Factor with ISTDs


    Signal 1: DAD1 A, Sig=254,4 Ref=off

    Peak RetTime Type  Width     Area      Height     Area
      #   [min]        [min]   [mAU*s]     [mAU]        %
    ----|-------|----|-------|----------|----------|--------|
       1   7.451 BV    0.0511 1.08159e4  3402.30518  47.4057
       2   7.506 VV R  0.0534 1.18947e4  3347.82104  52.1343
       3   8.125 BV E  0.0416    6.88685    2.46230   0.0302
       4   8.234 VB R  0.0405   98.07407   36.34929   0.4299

    Totals :                  2.28156e4  6788.93781


    =====================================================================
```

Compound 8b

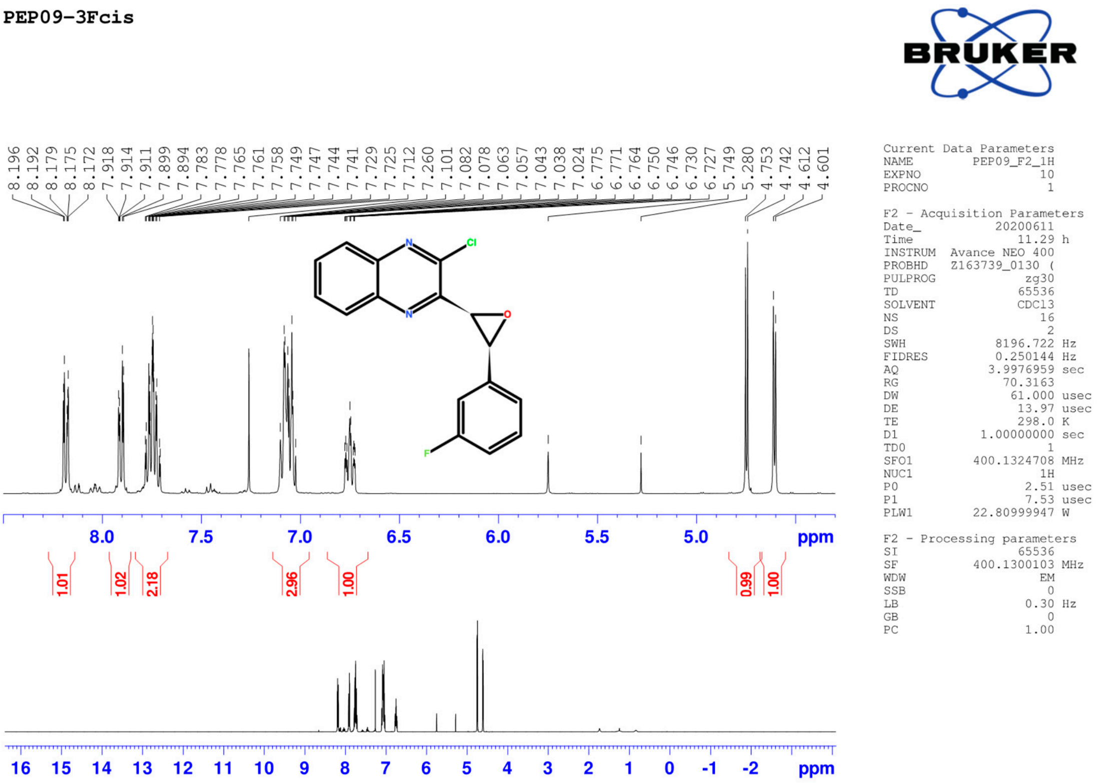
PEP09-3Fcis
8.196 8.192 8.179 8.175 8.172 7.918 7.914 7.911 7.899 7.894 7.783 7.778 7.765 7.761 7.758 7.749 7.747 7.744 7.741 7.729 7.725 7.712 7.260 7.101 7.082 7.078 7.063 7.057 7.043 7.038 7.024 6.775 6.771 6.764 6.750 6.746 6.730 6.727 5.749 5.280 4.753 4.742 4.612 4.601
N Cl N O F
8.0 7.5 7.0 6.5 6.0 5.5 5.0 ppm
1.01 1.02 2.18 2.96 1.00 0.99 1.00
16 15 14 13 12 11 10 9 8 7 6 5 4 3 2 1 0 -1 -2 ppm
Current Data Parameters
NAME PEP09_F2_1H
EXPNO 10
PROCNO 1
F2 - Acquisition Parameters
Date_ 20200611
Time 11.29 h
INSTRUM Avance NEO 400
PROBHD Z163739_0130 (
PULPROG zg30
TD 65536
SOLVENT CDCl3
NS 16
DS 2
SWH 8196.722 Hz
FIDRES 0.250144 Hz
AQ 3.9976959 sec
RG 70.3163
DW 61.000 usec
DE 13.97 usec
TE 298.0 K
D1 1.00000000 sec
TD0 1
SFO1 400.1324708 MHz
NUC1 1H
P0 2.51 usec
P1 7.53 usec
PLW1 22.80999947 W
F2 - Processing parameters
SI 65536
SF 400.1300103 MHz
WDW EM
SSB 0
LB 0.30 Hz
GB 0
PC 1.00

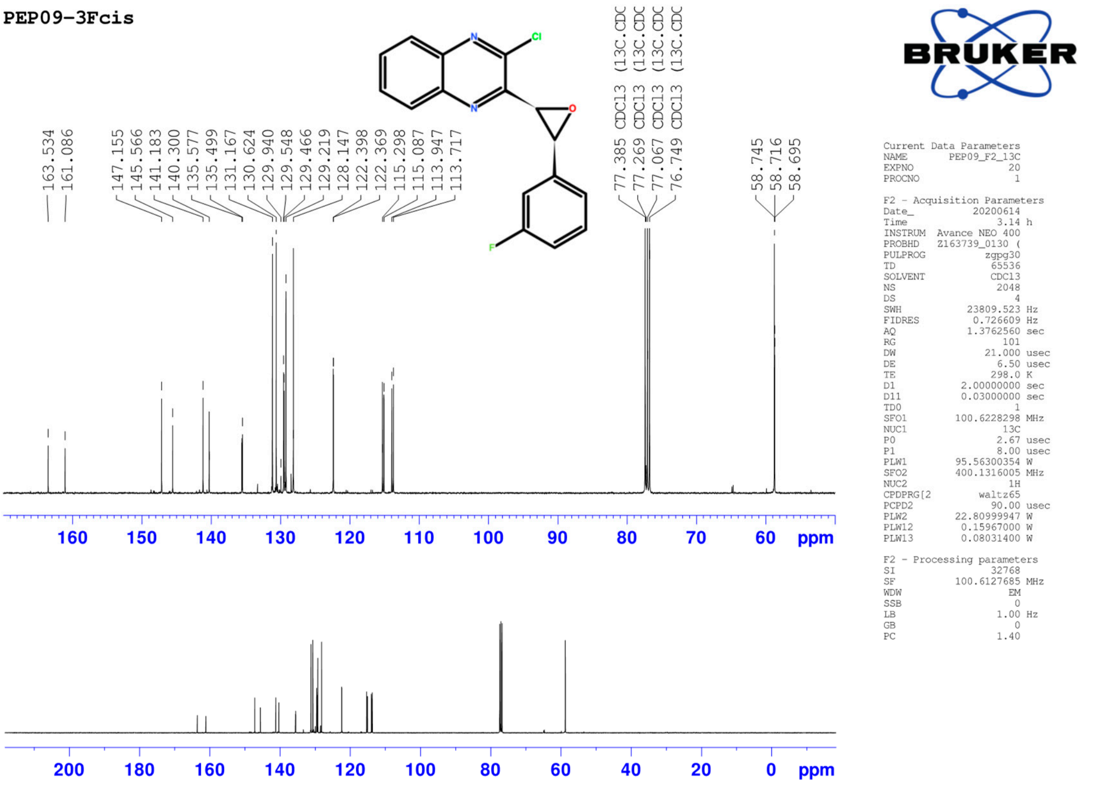
PEP09-3Fcis
163.534 161.086 147.155 145.566 141.183 140.300 135.577 135.499 131.167 130.624 129.940 129.548 129.466 129.219 128.147 122.398 122.369 115.298 115.087 113.947 113.717
77.385 CDCl3 (13C.CDC 77.269 CDCl3 (13C.CDC 77.067 CDCl3 (13C.CDC 76.749 CDCl3 (13C.CDC
58.745 58.716 58.695
N Cl N O F
160 150 140 130 120 110 100 90 80 70 60 ppm
200 180 160 140 120 100 80 60 40 20 0 ppm
Current Data Parameters
NAME PEP09_F2_13C
EXPNO 20
PROCNO 1
F2 - Acquisition Parameters
Date_ 20200614
Time 3.14 h
INSTRUM Avance NEO 400
PROBHD Z163739_0130 (
PULPROG zgpg30
TD 65536
SOLVENT CDCl3
NS 2048
DS 4
SWH 23809.523 Hz
FIDRES 0.726609 Hz
AQ 1.3762560 sec
RG 101
DW 21.000 usec
DE 6.50 usec
TE 298.0 K
D1 2.00000000 sec
D11 0.03000000 sec
TD0 1
SFO1 100.6228298 MHz
NUC1 13C
P0 2.67 usec
P1 8.00 usec
PLW1 95.56300354 W
SFO2 400.1316005 MHz
NUC2 1H
CPDPRG[2 waltz65
PCPD2 90.00 usec
PLW2 22.80999947 W
PLW12 0.15967000 W
PLW13 0.08031400 W
F2 - Processing parameters
SI 32768
SF 100.6127685 MHz
WDW EM
SSB 0
LB 1.00 Hz
GB 0
PC 1.40

```
Data File C:\Chem32\...rganique\prestation_chimie_organique 2022-05-31\OnlineEdited--013.D
Sample Name: PEP09F2

    =====================================================================
    Acq. Operator   : SYSTEM                        Seq. Line :  13
    Acq. Instrument : LC1290                         Location :   P1-B3
    Injection Date  : 31/5/2022 4:18:32 PM                Inj :   1
                                                  Inj Volume : 1.000 µl
    Method          : C:\Chem32\1\Data\prestation_chimie_organique\prestation_chimie_organique
                      2022-05-31\prestation_chimie_org.M (Sequence Method)
    Last changed    : 31/5/2022 1:07:56 PM by SYSTEM
    Additional Info : Peak(s) manually integrated
```

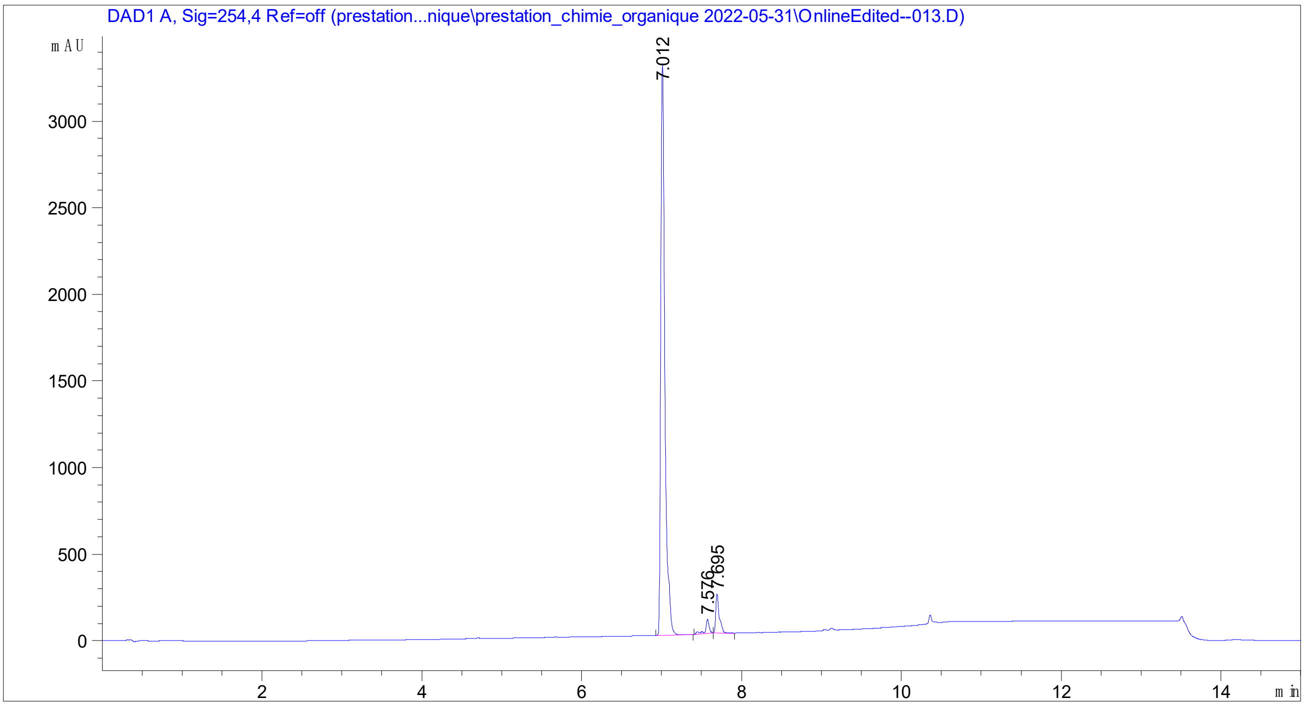


```
    =====================================================================
                             Area Percent Report
    =====================================================================

    Sorted By             :      Signal
    Multiplier            :      1.0000
    Dilution              :      1.0000
    Use Multiplier & Dilution Factor with ISTDs


    Signal 1: DAD1 A, Sig=254,4 Ref=off

    Peak RetTime Type  Width     Area      Height     Area
      #   [min]        [min]   [mAU*s]     [mAU]        %
    ----|-------|----|-------|----------|----------|--------|
       1   7.012 BV R  0.0528 1.15331e4  3299.14502  92.3663
       2   7.576 VB R  0.0371  265.32071   82.35912   2.1249
       3   7.695 BV R  0.0441  687.83380  226.55388   5.5087

    Totals :                  1.24862e4  3608.05801


    =====================================================================
                              *** End of Report ***
```

Compound 9a

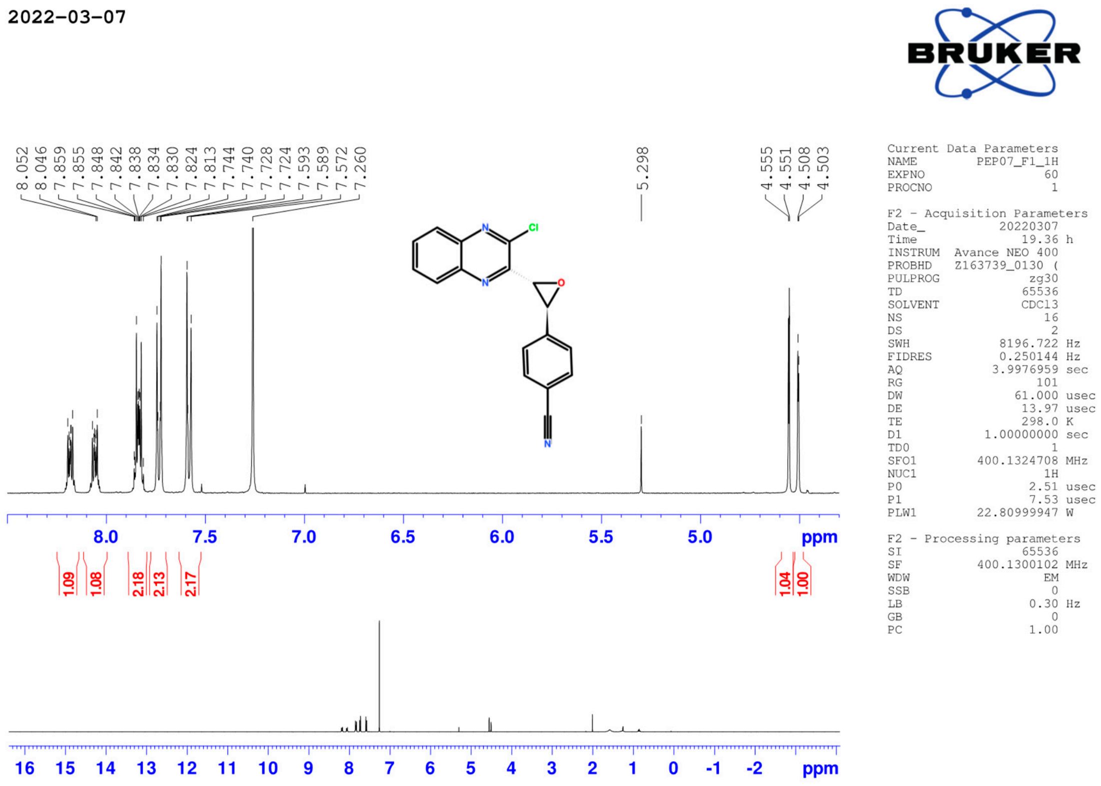

2022-03-07
BRUKER
8.052 8.046 7.859 7.855 7.848 7.842 7.838 7.834 7.830 7.824 7.813 7.744 7.740 7.728 7.724 7.593 7.589 7.572 7.260
5.298
4.555 4.551 4.508 4.503
N
Cl
O
1.09 1.08 2.18 2.13 2.17
1.04 1.00
ppm
Current Data Parameters
NAME PEP07_F1_1H
EXPNO 60
PROCNO 1
F2 - Acquisition Parameters
Date_ 20220307
Time 19.36 h
INSTRUM Avance NEO 400
PROBHD Z163739_0130 (
PULPROG zg30
TD 65536
SOLVENT CDCl3
NS 16
DS 2
SWH 8196.722 Hz
FIDRES 0.250144 Hz
AQ 3.9976959 sec
RG 101
DW 61.000 usec
DE 13.97 usec
TE 298.0 K
D1 1.00000000 sec
TD0 1
SFO1 400.1324708 MHz
NUC1 1H
P0 2.51 usec
P1 7.53 usec
PLW1 22.80999947 W
F2 - Processing parameters
SI 65536
SF 400.1300102 MHz
WDW EM
SSB 0
LB 0.30 Hz
GB 0
PC 1.00

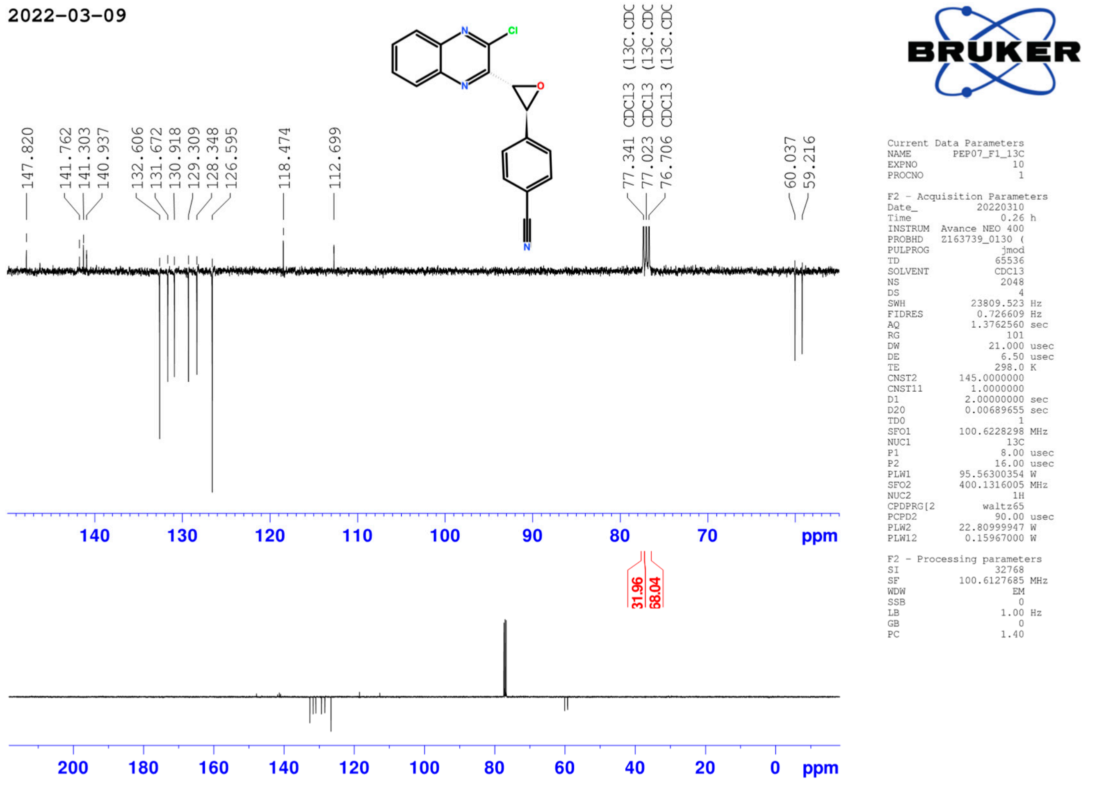

2022-03-09
BRUKER
147.820
141.762 141.303 140.937
132.606 131.672 130.918 129.309 128.348 126.595
118.474
112.699
77.341 CDCl3 (13C.CDC
77.023 CDCl3 (13C.CDC
76.706 CDCl3 (13C.CDC
60.037 59.216
N
Cl
O
31.96 68.04
ppm
Current Data Parameters
NAME PEP07_F1_13C
EXPNO 10
PROCNO 1
F2 - Acquisition Parameters
Date_ 20220310
Time 0.26 h
INSTRUM Avance NEO 400
PROBHD Z163739_0130 (
PULPROG jmod
TD 65536
SOLVENT CDCl3
NS 2048
DS 4
SWH 23809.523 Hz
FIDRES 0.726609 Hz
AQ 1.3762560 sec
RG 101
DW 21.000 usec
DE 6.50 usec
TE 298.0 K
CNST2 145.0000000
CNST11 1.0000000
D1 2.00000000 sec
D20 0.00689655 sec
TD0 1
SFO1 100.6228298 MHz
NUC1 13C
P1 8.00 usec
P2 16.00 usec
PLW1 95.56300354 W
SFO2 400.1316005 MHz
NUC2 1H
CPDPRG[2 waltz65
PCPD2 90.00 usec
PLW2 22.80999947 W
PLW12 0.15967000 W
F2 - Processing parameters
SI 32768
SF 100.6127685 MHz
WDW EM
SSB 0
LB 1.00 Hz
GB 0
PC 1.40

```
Data File C:\Chem32\...rganique\prestation_chimie_organique 2022-05-31\OnlineEdited--008.D
Sample Name: PEP07F1

    =====================================================================
    Acq. Operator   : SYSTEM                         Seq. Line :    8
    Acq. Instrument : LC1290                          Location :    P1-A7
    Injection Date  : 31/5/2022 2:59:28 PM                 Inj :    1
                                                    Inj Volume : 1.000 µl
    Method          : C:\Chem32\1\Data\prestation_chimie_organique\prestation_chimie_organique
                      2022-05-31\prestation_chimie_org.M (Sequence Method)
    Last changed    : 31/5/2022 1:07:56 PM by SYSTEM
    Additional Info : Peak(s) manually integrated
```

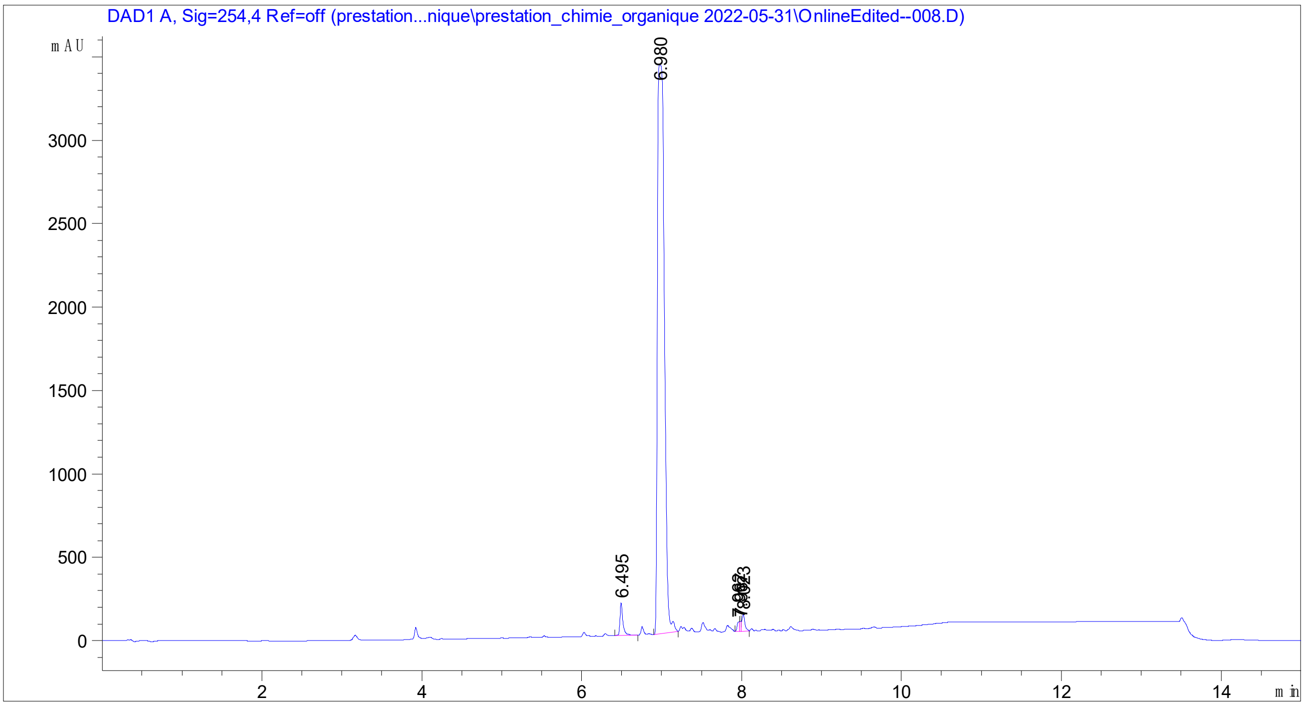


```
    =====================================================================
                             Area Percent Report
    =====================================================================

    Sorted By             :      Signal
    Multiplier            :      1.0000
    Dilution              :      1.0000
    Use Multiplier & Dilution Factor with ISTDs


    Signal 1: DAD1 A, Sig=254,4 Ref=off
```

Peak #	RetTime [min]	Type	Width [min]	Area [mAU*s]	Height [mAU]	Area %
1	6.495	VV R	0.0409	536.18005	195.82469	2.4979
2	6.980	BV R	0.0945	2.04915e4	3411.73242	95.4624
3	7.962	BV	0.0273	98.75806	56.66538	0.4601
4	7.987	VV	0.0210	84.59011	58.14962	0.3941
5	8.023	VB	0.0372	254.48518	98.30723	1.1856

```
    Totals :                  2.14655e4  3820.67934
```

Compound 9b

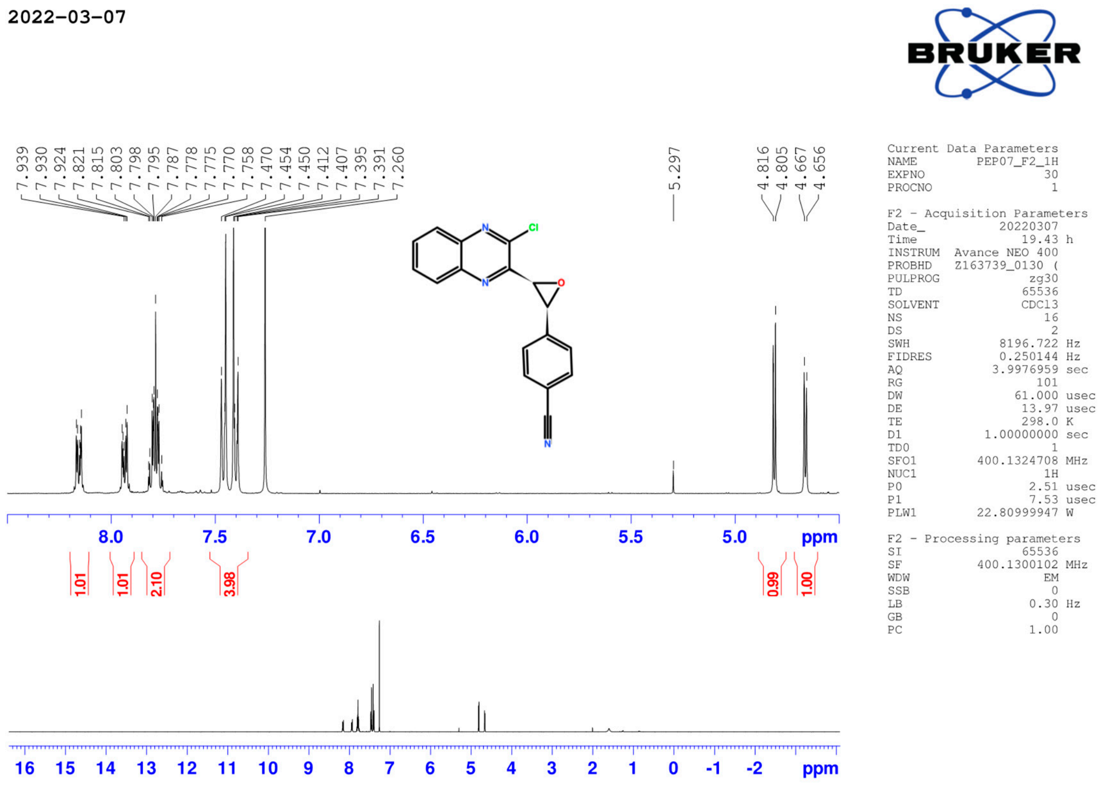
2022-03-07
7.939 7.930 7.924 7.821 7.815 7.803 7.798 7.795 7.787 7.778 7.775 7.770 7.758 7.470 7.454 7.450 7.412 7.407 7.395 7.391 7.260
5.297
4.816 4.805 4.667 4.656
N
Cl
O
N
BRUKER
Current Data Parameters
NAME PEP07_F2_1H
EXPNO 30
PROCNO 1
F2 - Acquisition Parameters
Date_ 20220307
Time 19.43 h
INSTRUM Avance NEO 400
PROBHD Z163739_0130 (
PULPROG zg30
TD 65536
SOLVENT CDCl3
NS 16
DS 2
SWH 8196.722 Hz
FIDRES 0.250144 Hz
AQ 3.9976959 sec
RG 101
DW 61.000 usec
DE 13.97 usec
TE 298.0 K
D1 1.00000000 sec
TD0 1
SFO1 400.1324708 MHz
NUC1 1H
P0 2.51 usec
P1 7.53 usec
PLW1 22.80999947 W
F2 - Processing parameters
SI 65536
SF 400.1300102 MHz
WDW EM
SSB 0
LB 0.30 Hz
GB 0
PC 1.00
8.0 7.5 7.0 6.5 6.0 5.5 5.0 ppm
1.01 1.01 2.10 3.98 0.99 1.00
16 15 14 13 12 11 10 9 8 7 6 5 4 3 2 1 0 -1 -2 ppm

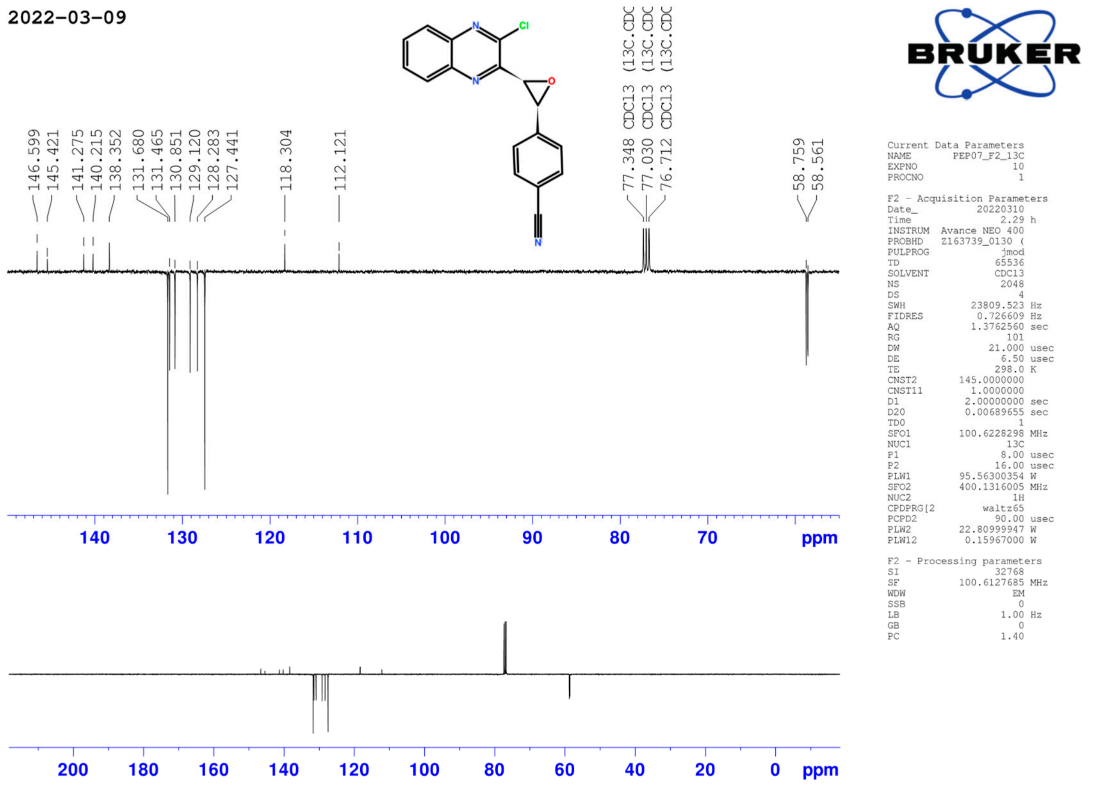
2022-03-09
146.599 145.421 141.275 140.215 138.352 131.680 131.465 130.851 129.120 128.283 127.441 118.304 112.121
77.348 CDCl3 (13C.CDC
77.030 CDCl3 (13C.CDC
76.712 CDCl3 (13C.CDC
58.759 58.561
N
Cl
O
N
BRUKER
Current Data Parameters
NAME PEP07_F2_13C
EXPNO 10
PROCNO 1
F2 - Acquisition Parameters
Date_ 20220310
Time 2.29 h
INSTRUM Avance NEO 400
PROBHD Z163739_0130 (
PULPROG jmod
TD 65536
SOLVENT CDCl3
NS 2048
DS 4
SWH 23809.523 Hz
FIDRES 0.726609 Hz
AQ 1.3762560 sec
RG 101
DW 21.000 usec
DE 6.50 usec
TE 298.0 K
CNST2 145.0000000
CNST11 1.0000000
D1 2.00000000 sec
D20 0.00689655 sec
TD0 1
SFO1 100.6228298 MHz
NUC1 13C
P1 8.00 usec
P2 16.00 usec
PLW1 95.56300354 W
SFO2 400.1316005 MHz
NUC2 1H
CPDPRG[2 waltz65
PCPD2 90.00 usec
PLW2 22.80999947 W
PLW12 0.15967000 W
F2 - Processing parameters
SI 32768
SF 100.6127685 MHz
WDW EM
SSB 0
LB 1.00 Hz
GB 0
PC 1.40
140 130 120 110 100 90 80 70 ppm
200 180 160 140 120 100 80 60 40 20 0 ppm

```
Data File C:\Chem32\...rganique\prestation_chimie_organique 2022-05-31\OnlineEdited--009.D
Sample Name: PEP07F2

    =====================================================================
    Acq. Operator   : SYSTEM                          Seq. Line :    9
    Acq. Instrument : LC1290                           Location :    P1-A8
    Injection Date  : 31/5/2022 3:15:15 PM                  Inj :    1
                                                     Inj Volume : 1.000 µl
    Method          : C:\Chem32\1\Data\prestation_chimie_organique\prestation_chimie_organique
                      2022-05-31\prestation_chimie_org.M (Sequence Method)
    Last changed    : 31/5/2022 1:07:56 PM by SYSTEM
    Additional Info : Peak(s) manually integrated
```

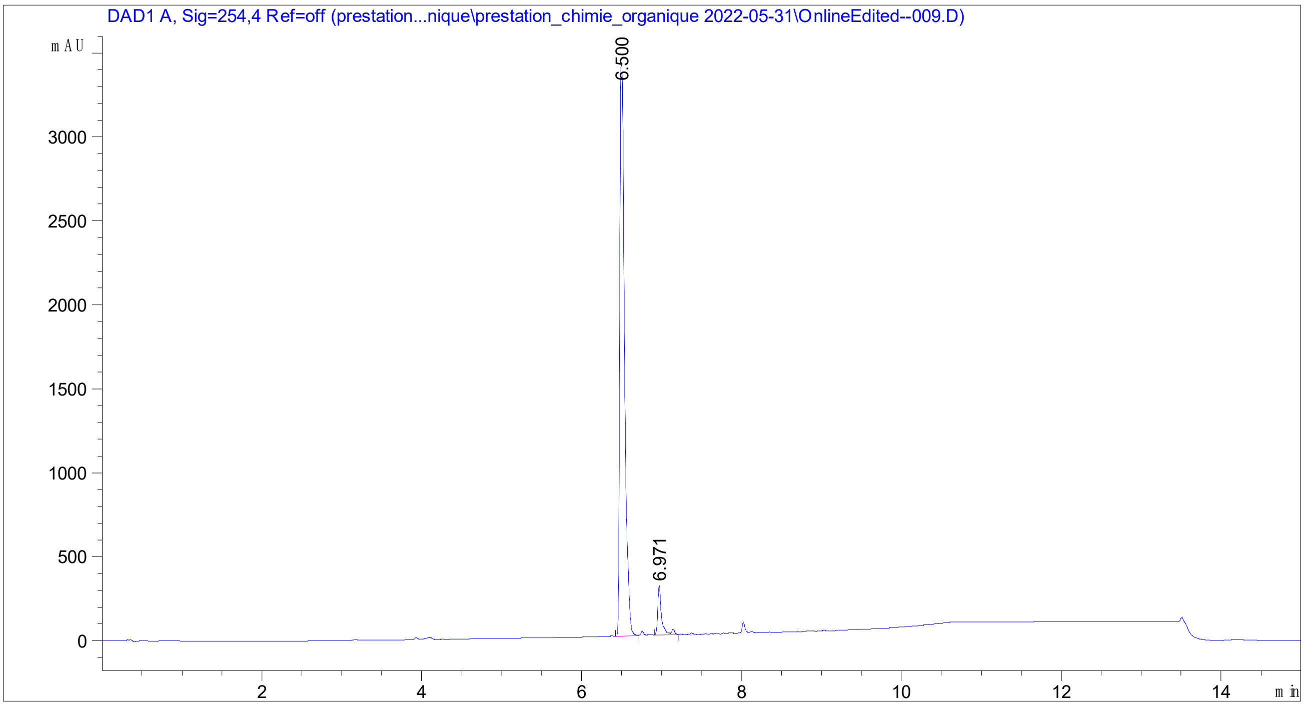


```
    =====================================================================
                             Area Percent Report
    =====================================================================

    Sorted By             :      Signal
    Multiplier            :      1.0000
    Dilution              :      1.0000
    Use Multiplier & Dilution Factor with ISTDs


    Signal 1: DAD1 A, Sig=254,4 Ref=off

    Peak RetTime Type  Width     Area      Height     Area
      #   [min]        [min]   [mAU*s]     [mAU]        %
    ----|-------|----|-------|----------|----------|--------|
       1   6.500 BV    0.0660 1.45766e4  3403.29370  93.5690
       2   6.971 BV R  0.0438 1001.83966  294.29852   6.4310

    Totals :                  1.55784e4  3697.59222


    =====================================================================
                              *** End of Report ***
```

Compound 10a

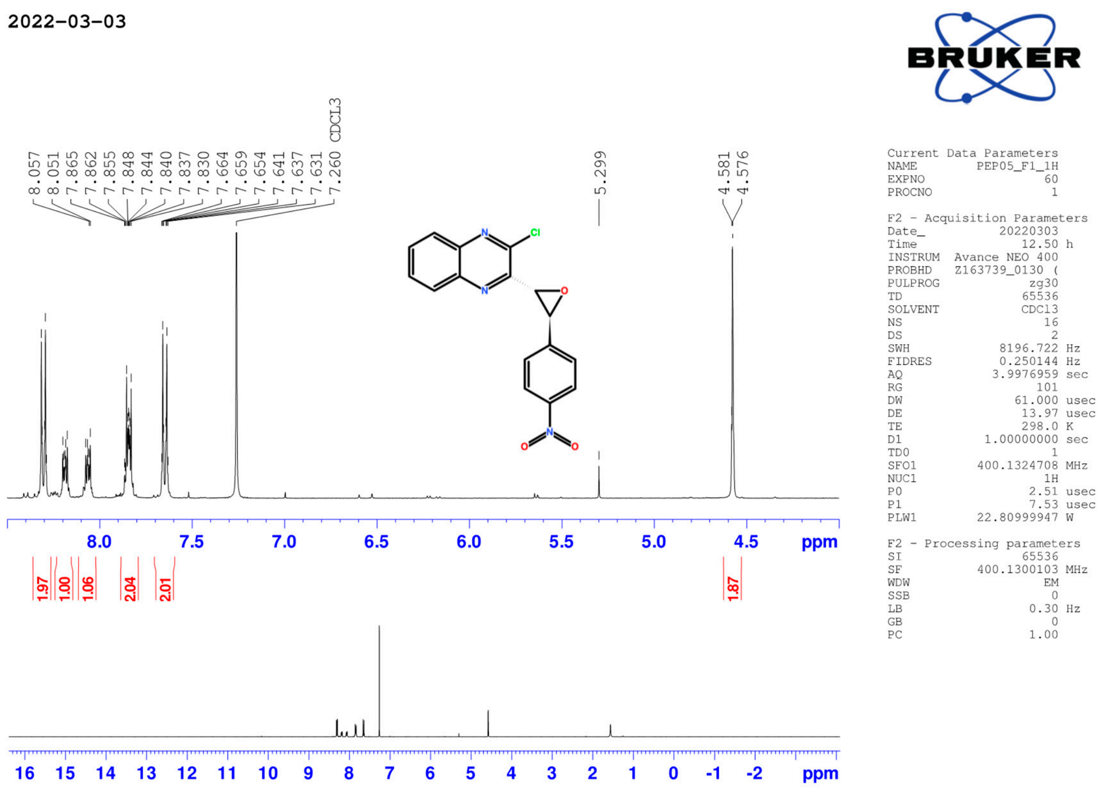

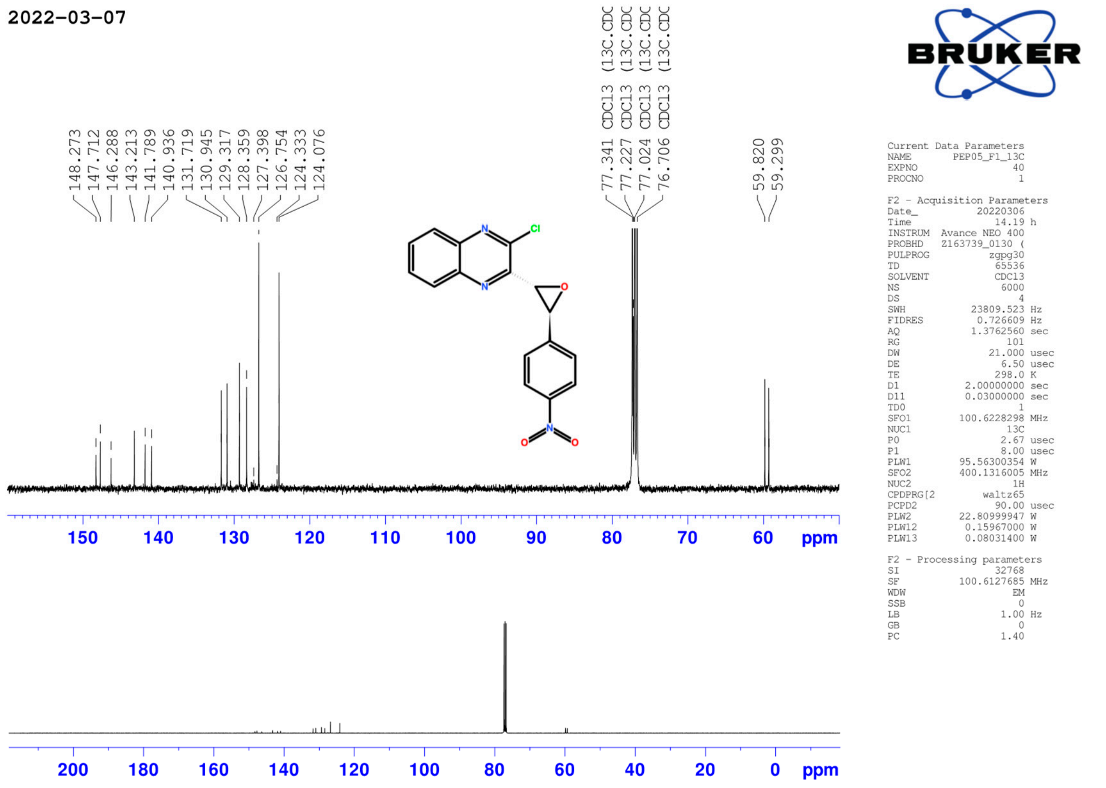

Compound 10b

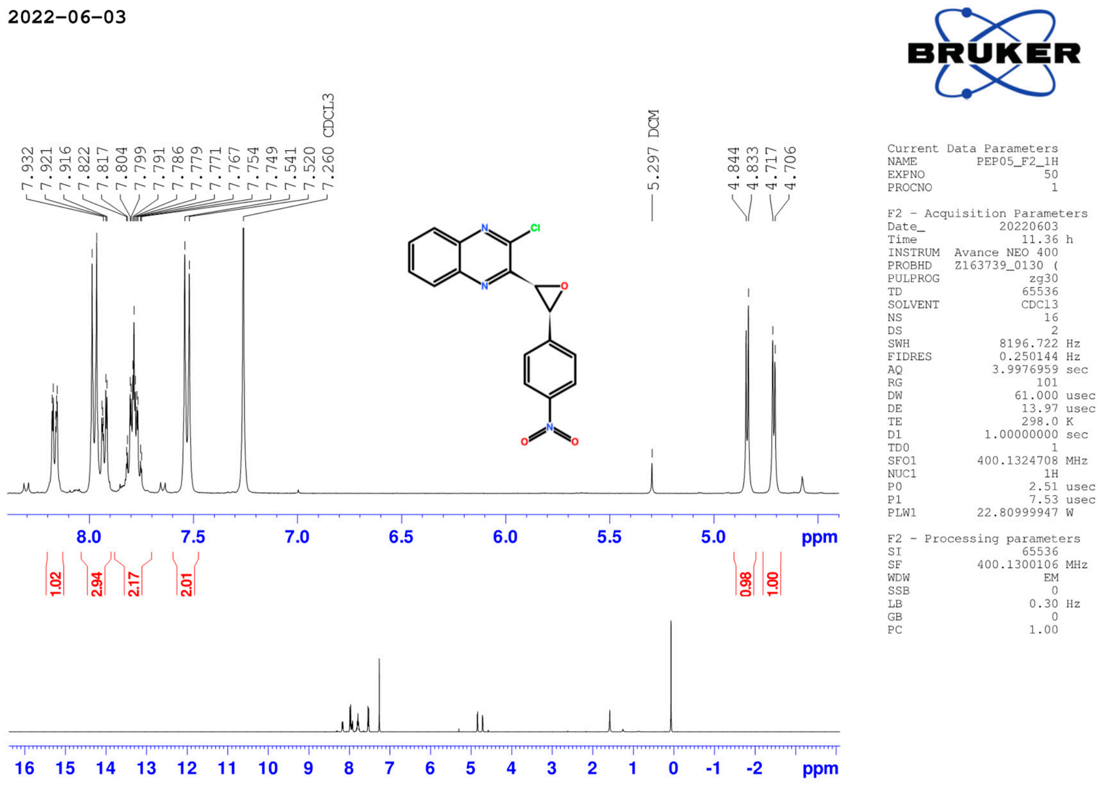

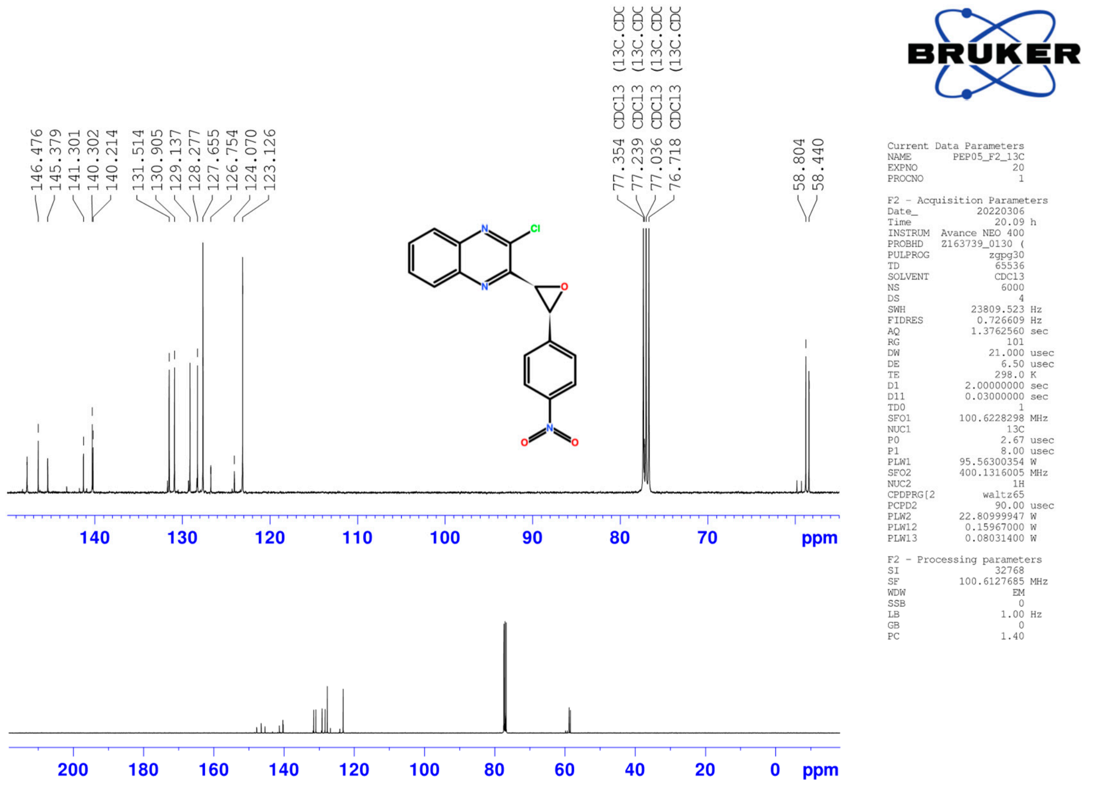

Compound 11a

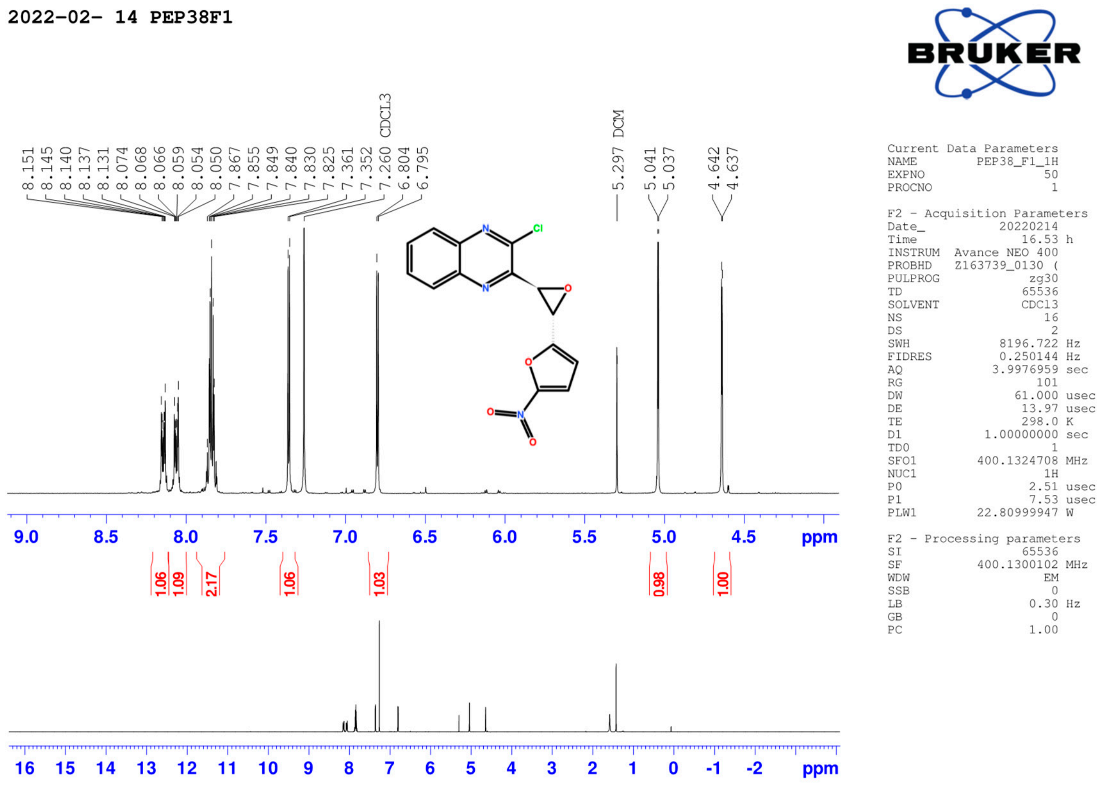

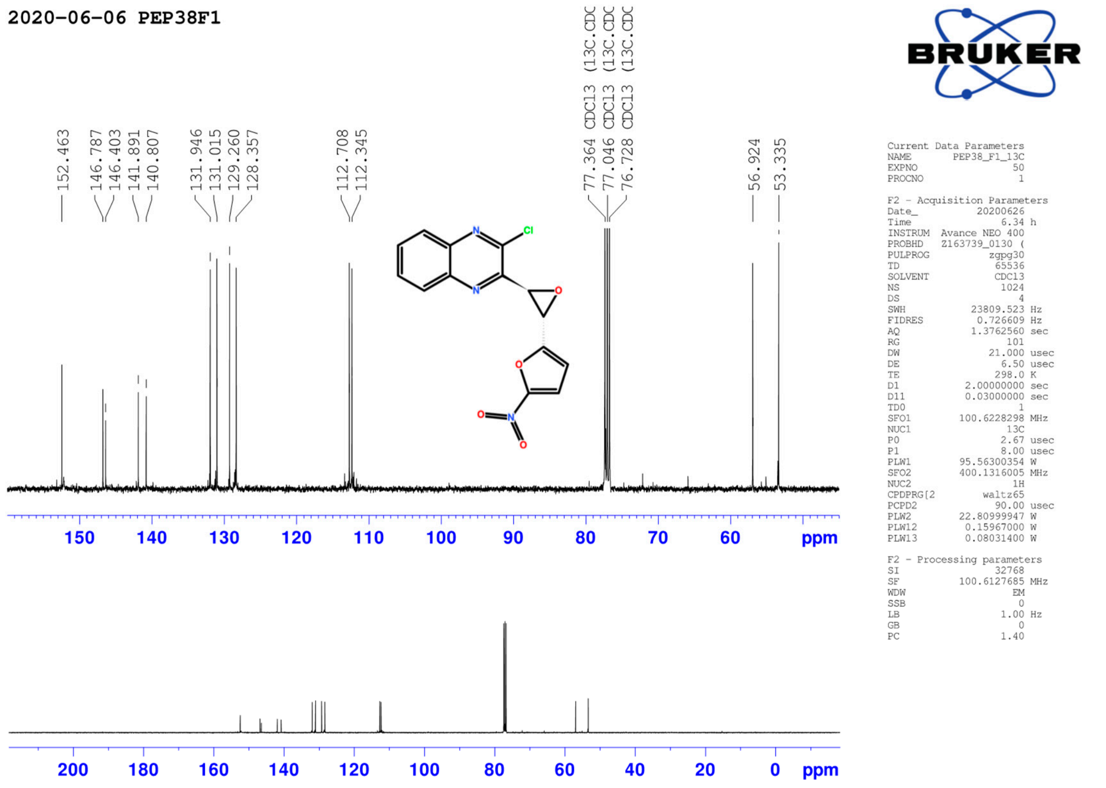

```
Data File C:\Chem32\...rganique\prestation_chimie_organique 2022-05-31\OnlineEdited--021.D
Sample Name: PEP38F1

    =====================================================================
    Acq. Operator   : SYSTEM                          Seq. Line :  21
    Acq. Instrument : LC1290                           Location :   P1-C2
    Injection Date  : 31/5/2022 6:28:05 PM                  Inj :   1
                                                     Inj Volume : 1.000 µl
    Method          : C:\Chem32\1\Data\prestation_chimie_organique\prestation_chimie_organique
                      2022-05-31\prestation_chimie_org.M (Sequence Method)
    Last changed    : 31/5/2022 1:07:56 PM by SYSTEM
    Additional Info : Peak(s) manually integrated
```

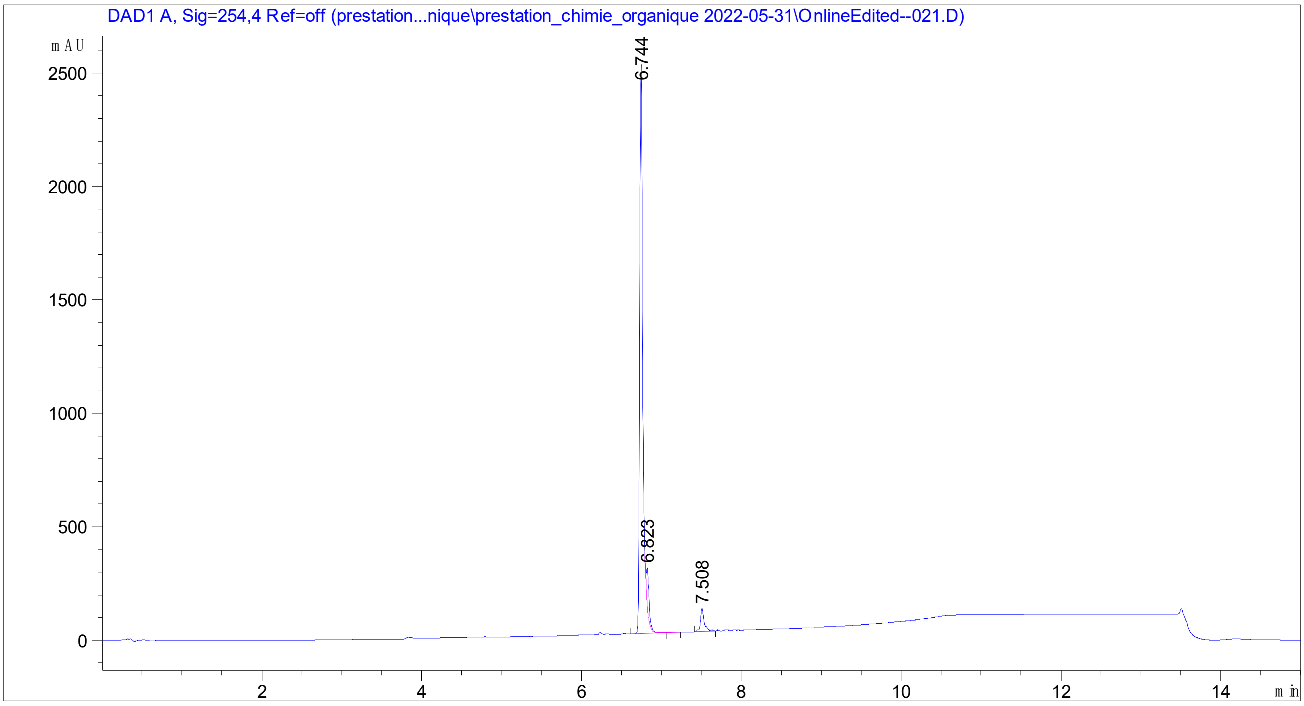


```
    =====================================================================
                             Area Percent Report
    =====================================================================

    Sorted By             :      Signal
    Multiplier            :      1.0000
    Dilution              :      1.0000
    Use Multiplier & Dilution Factor with ISTDs


    Signal 1: DAD1 A, Sig=254,4 Ref=off

    Peak RetTime Type  Width      Area      Height     Area
      #   [min]        [min]    [mAU*s]     [mAU]        %
    ----|-------|----|-------|----------|----------|--------|
       1   6.744 BV R  0.0422 7142.30420 2512.12720  90.6447
       2   6.823 VV E  0.0357  404.16541  164.43279   5.1294
       3   7.508 VV R  0.0452  332.97885  101.38938   4.2259

    Totals :                  7879.44846 2777.94936


    =====================================================================
                             *** End of Report ***
```

Compound 11b

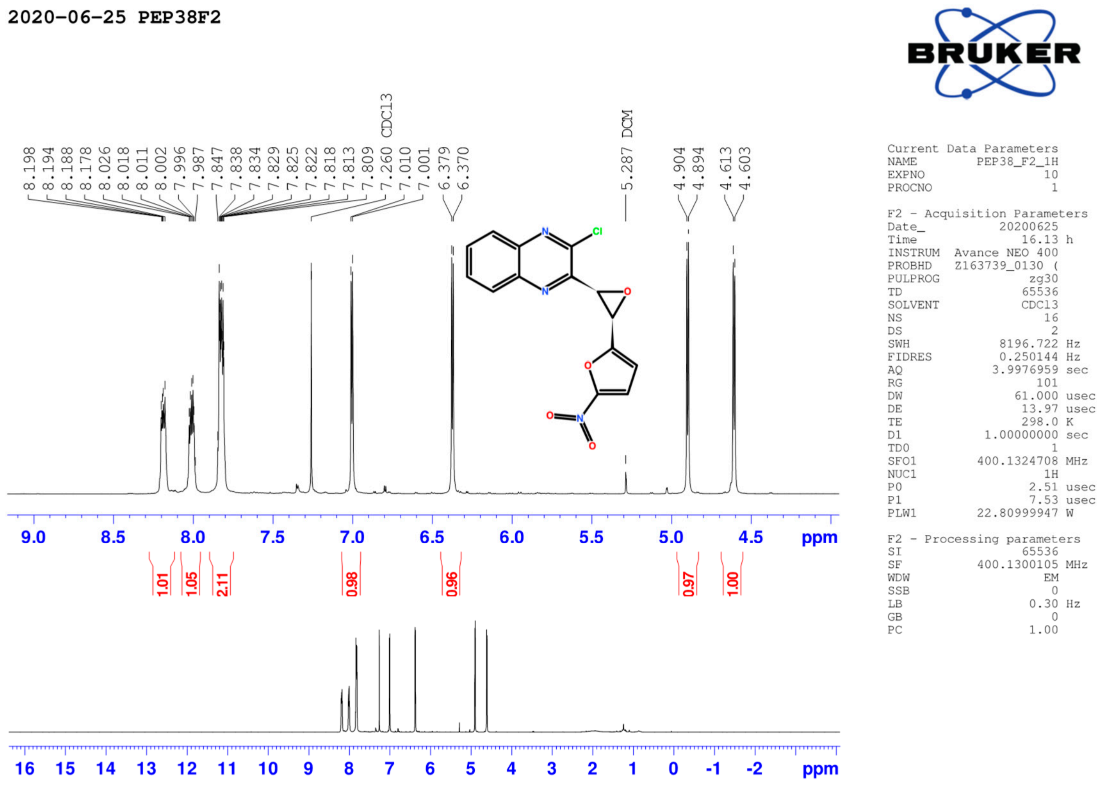

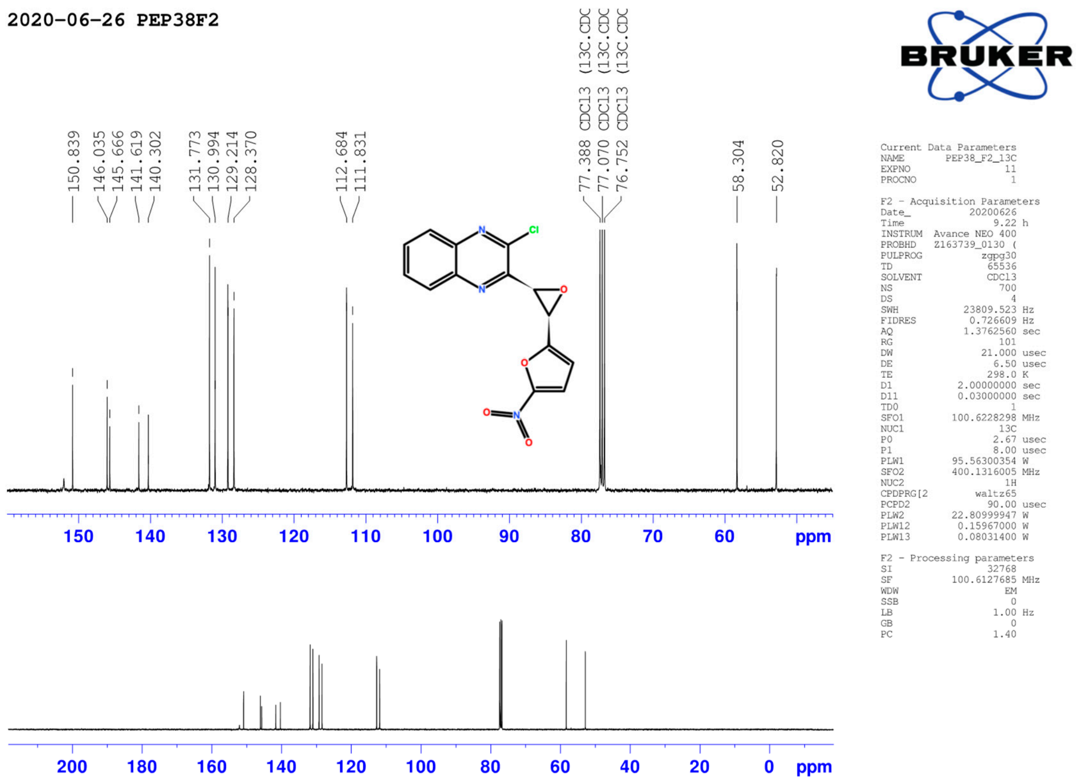

Compound 12a

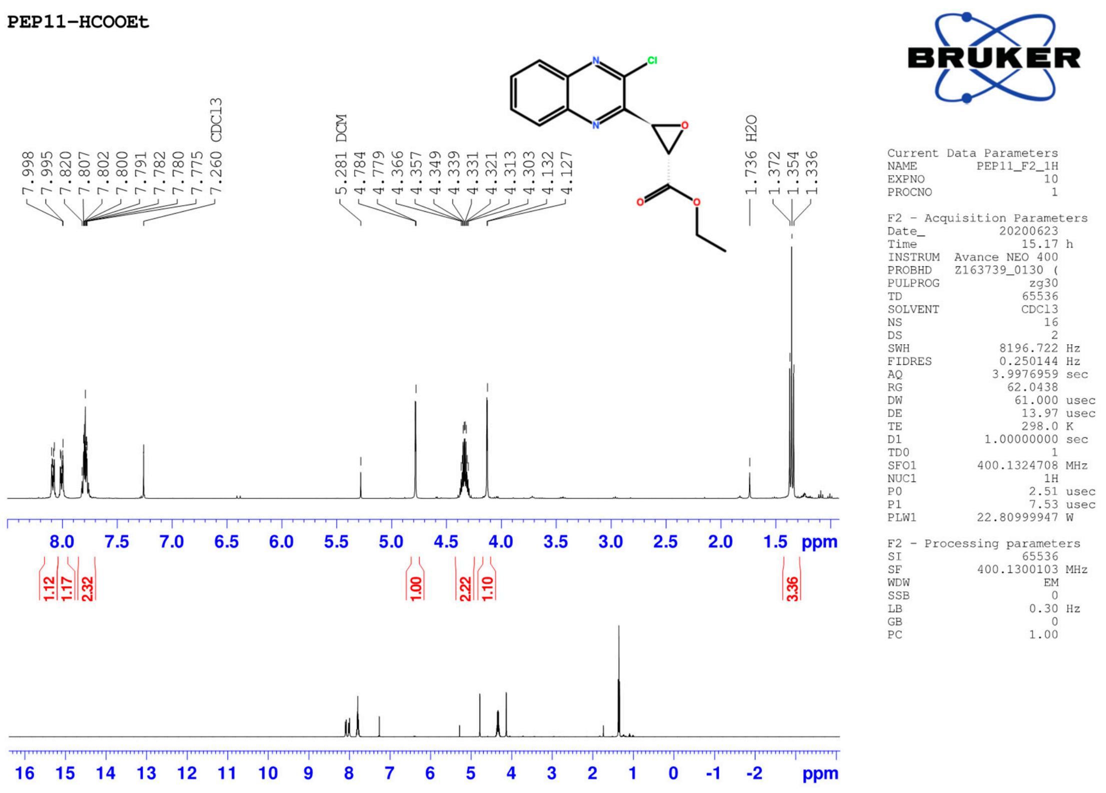

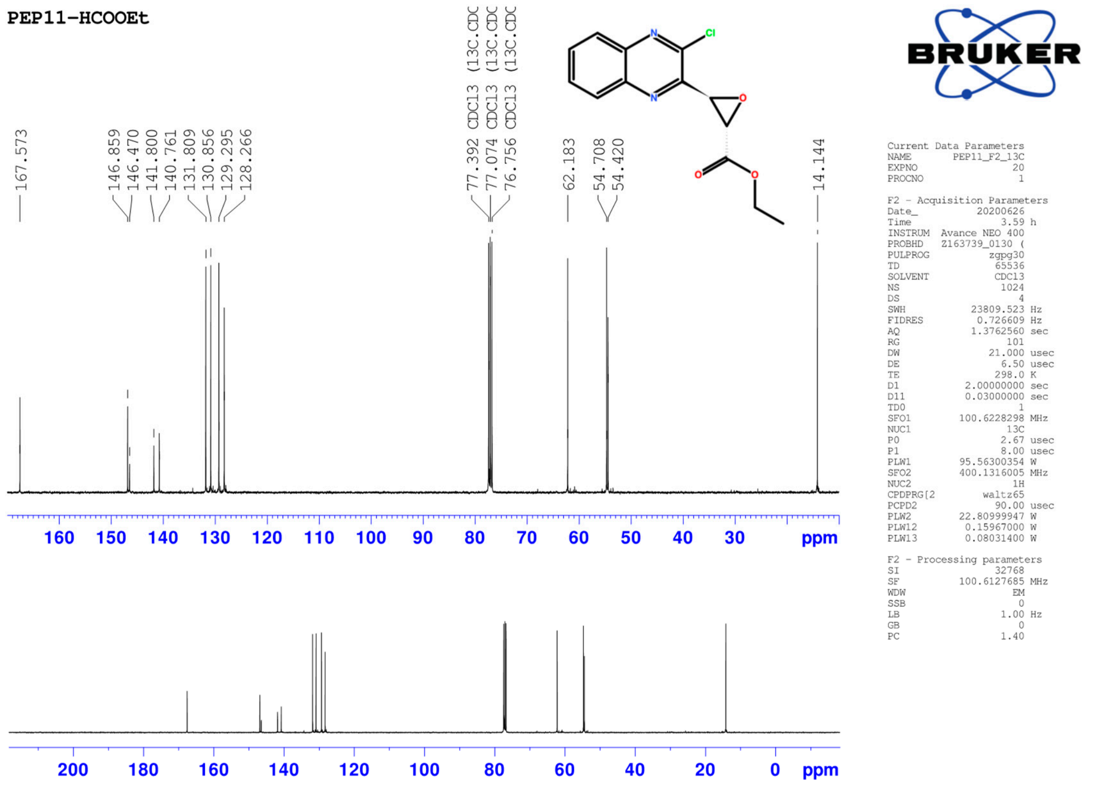

Compound 13

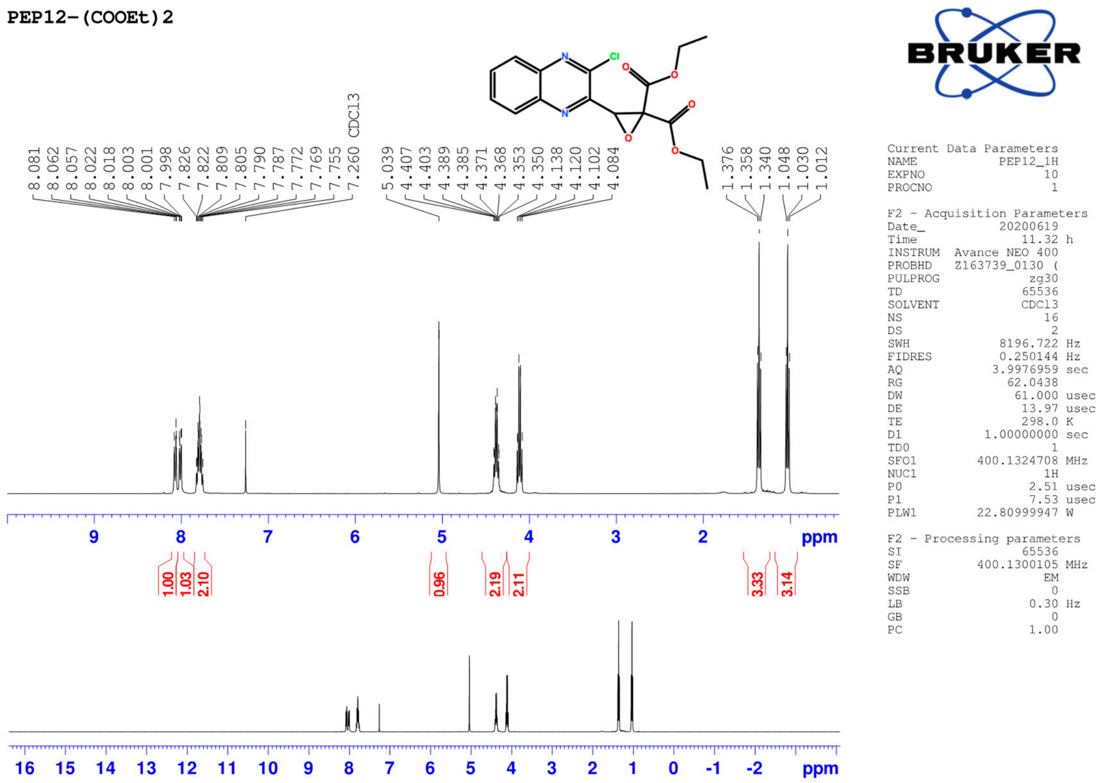
PEP12-(COOEt)2
8.081
8.062
8.057
8.022
8.018
8.003
8.001
7.998
7.826
7.822
7.809
7.805
7.790
7.787
7.772
7.769
7.755
7.260 CDCl3
5.039
4.407
4.403
4.389
4.385
4.371
4.368
4.353
4.350
4.138
4.120
4.102
4.084
1.376
1.358
1.340
1.048
1.030
1.012
BRUKER
Current Data Parameters
NAME PEP12_1H
EXPNO 10
PROCNO 1
F2 - Acquisition Parameters
Date_ 20200619
Time 11.32 h
INSTRUM Avance NEO 400
PROBHD Z163739_0130 (
PULPROG zg30
TD 65536
SOLVENT CDCl3
NS 16
DS 2
SWH 8196.722 Hz
FIDRES 0.250144 Hz
AQ 3.9976959 sec
RG 62.0438
DW 61.000 usec
DE 13.97 usec
TE 298.0 K
D1 1.00000000 sec
TD0 1
SFO1 400.1324708 MHz
NUC1 1H
P0 2.51 usec
P1 7.53 usec
PLW1 22.80999947 W
F2 - Processing parameters
SI 65536
SF 400.1300105 MHz
WDW EM
SSB 0
LB 0.30 Hz
GB 0
PC 1.00
1.00
1.03
2.10
0.96
2.19
2.11
3.33
3.14
ppm

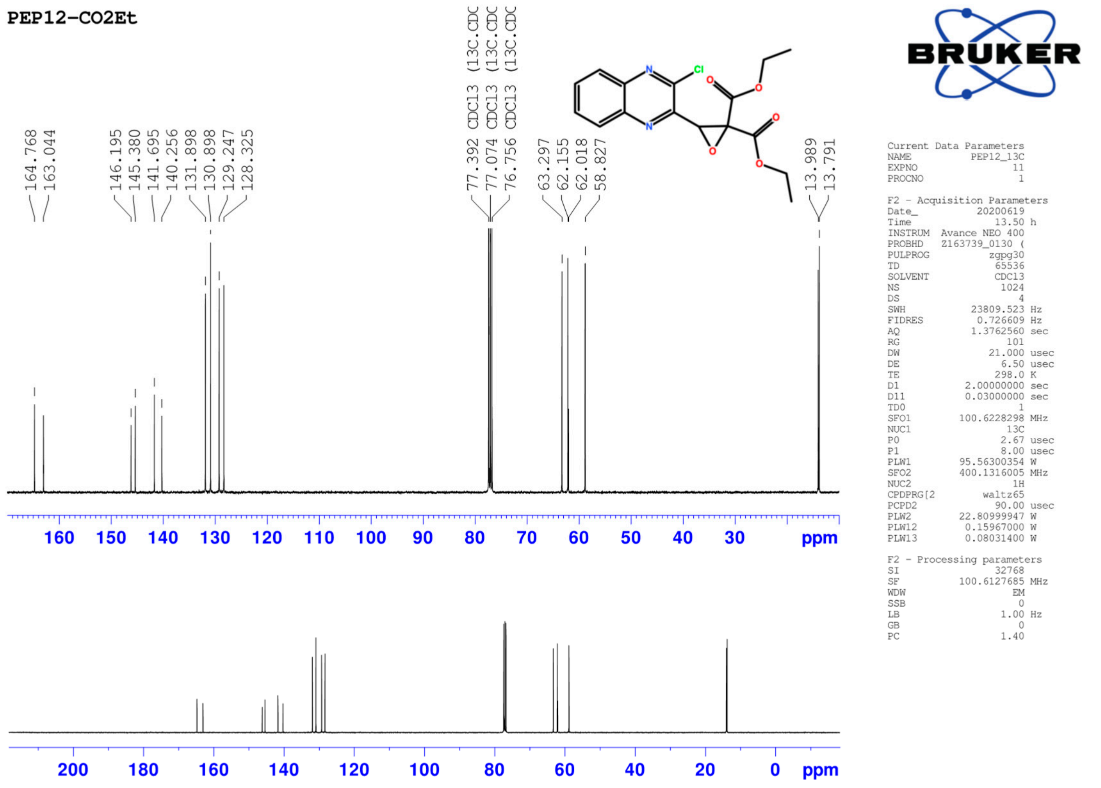
PEP12-CO2Et
164.768
163.044
146.195
145.380
141.695
140.256
131.898
130.898
129.247
128.325
77.392 CDCl3 (13C.CDC
77.074 CDCl3 (13C.CDC
76.756 CDCl3 (13C.CDC
63.297
62.155
62.018
58.827
13.989
13.791
BRUKER
Current Data Parameters
NAME PEP12_13C
EXPNO 11
PROCNO 1
F2 - Acquisition Parameters
Date_ 20200619
Time 13.50 h
INSTRUM Avance NEO 400
PROBHD Z163739_0130 (
PULPROG zgpg30
TD 65536
SOLVENT CDCl3
NS 1024
DS 4
SWH 23809.523 Hz
FIDRES 0.726609 Hz
AQ 1.3762560 sec
RG 101
DW 21.000 usec
DE 6.50 usec
TE 298.0 K
D1 2.00000000 sec
D11 0.03000000 sec
TD0 1
SFO1 100.6228298 MHz
NUC1 13C
P0 2.67 usec
P1 8.00 usec
PLW1 95.56300354 W
SFO2 400.1316005 MHz
NUC2 1H
CPDPRG[2 waltz65
PCPD2 90.00 usec
PLW2 22.80999947 W
PLW12 0.15967000 W
PLW13 0.08031400 W
F2 - Processing parameters
SI 32768
SF 100.6127685 MHz
WDW EM
SSB 0
LB 1.00 Hz
GB 0
PC 1.40
ppm

Compound 14a

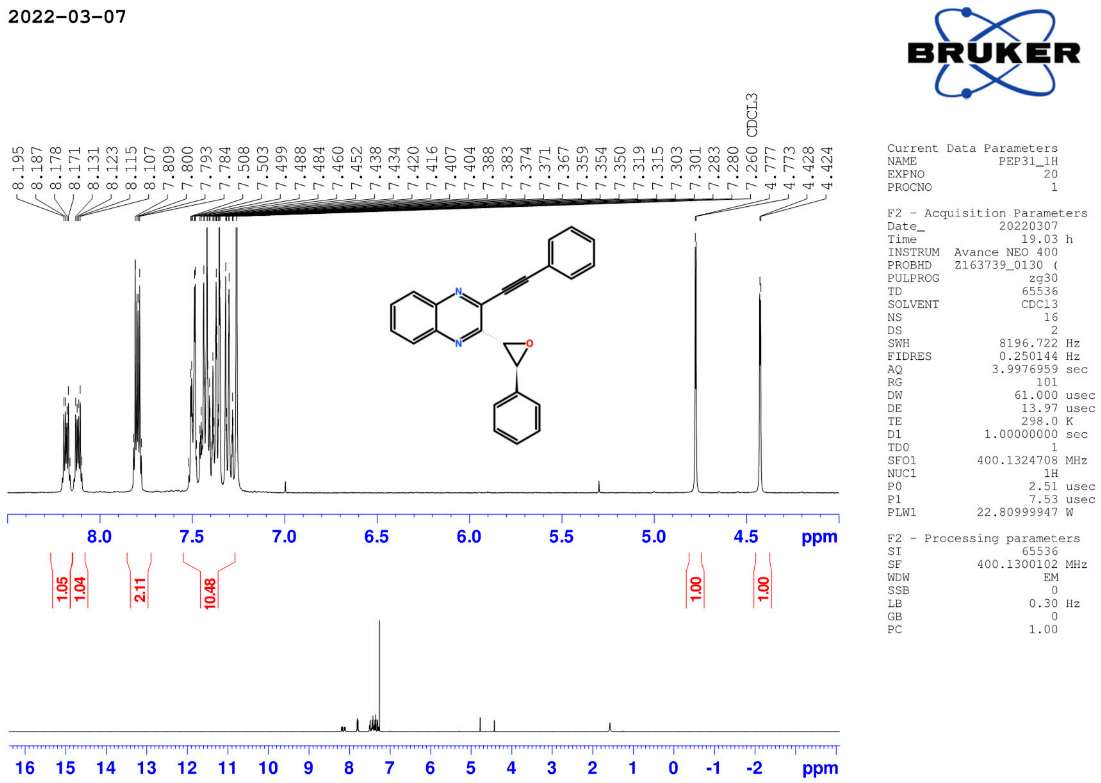

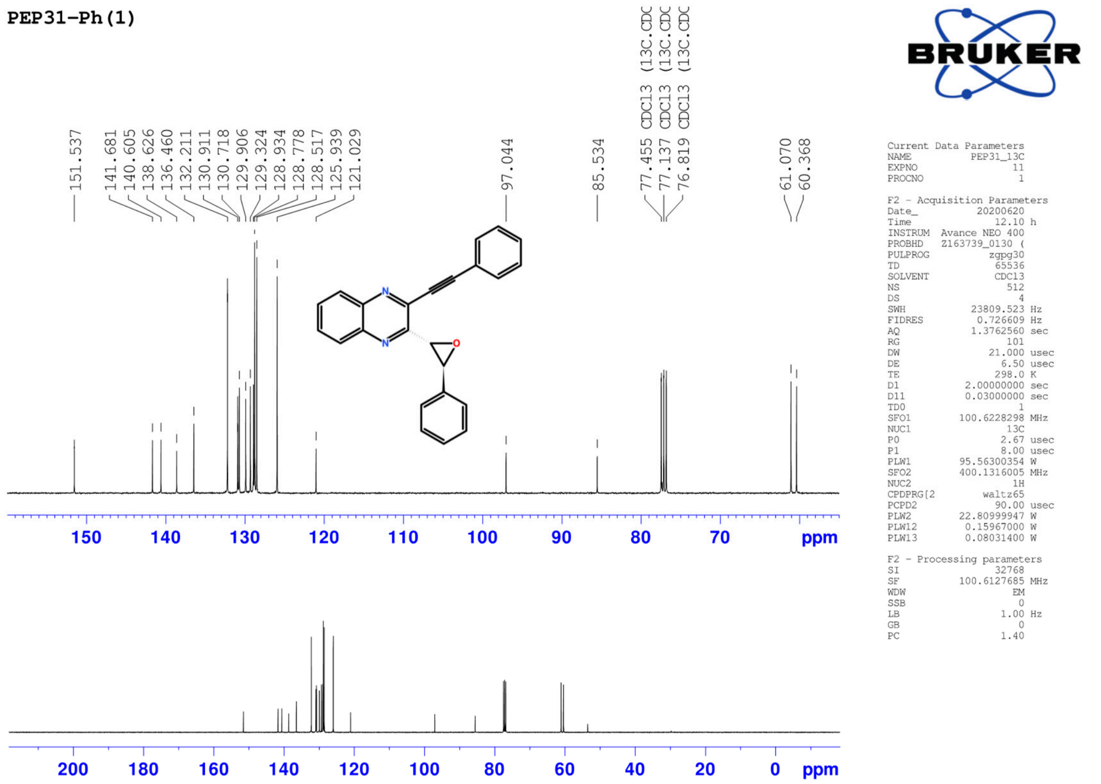

Data File C:\Chem32\...rganique\prestation_chimie_organique 2022-05-31\OnlineEdited--028.D
Sample Name: PEP31

===

Acq. Operator : SYSTEM Seq. Line : 28
Acq. Instrument : LC1290 Location : P1-C9
Injection Date : 31/5/2022 8:18:40 PM Inj : 1
Inj Volume : 1.000 µl
Method : C:\Chem32\1\Data\prestation_chimie_organique\prestation_chimie_organique 2022-05-31\prestation_chimie_org.M (Sequence Method)
Last changed : 31/5/2022 1:07:56 PM by SYSTEM
Additional Info : Peak(s) manually integrated

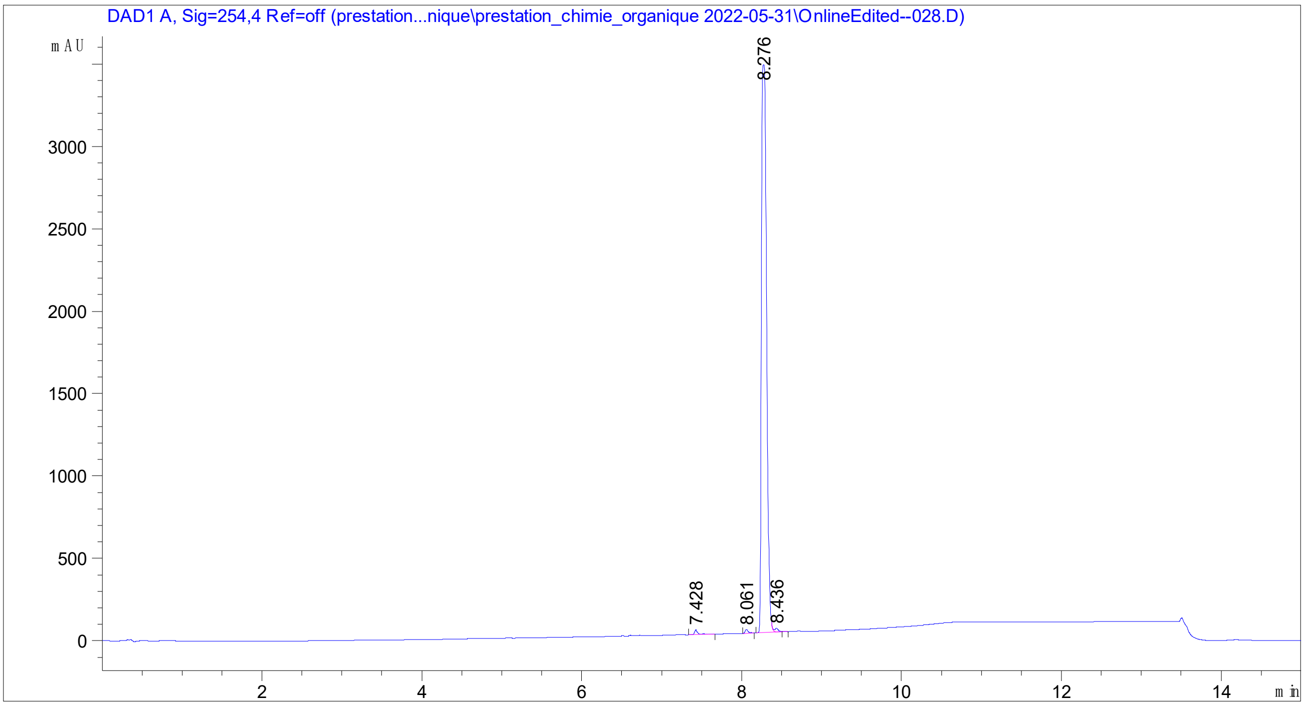

===

Area Percent Report

===

Sorted By : Signal
Multiplier : 1.0000
Dilution : 1.0000
Use Multiplier & Dilution Factor with ISTDs

Signal 1: DAD1 A, Sig=254,4 Ref=off

Peak #	RetTime [min]	Type	Width [min]	Area [mAU*s]	Height [mAU]	Area %
1	7.428	BV R	0.0406	97.75952	29.26268	0.6318
2	8.061	BV R	0.0385	61.14993	23.35368	0.3952
3	8.276	BV R	0.0706	1.52385e4	3445.06177	98.4887
4	8.436	VV E	0.0455	74.92388	23.92803	0.4842

Totals : 1.54724e4 3521.60615

===

Compound 14b

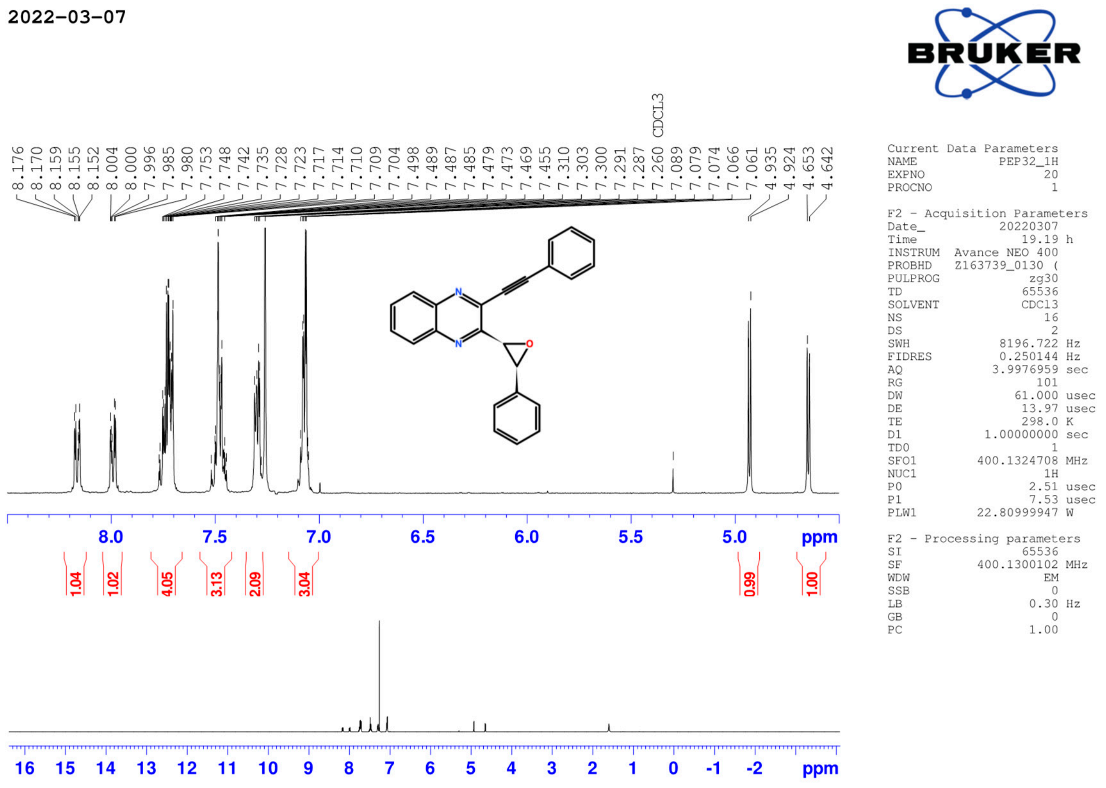

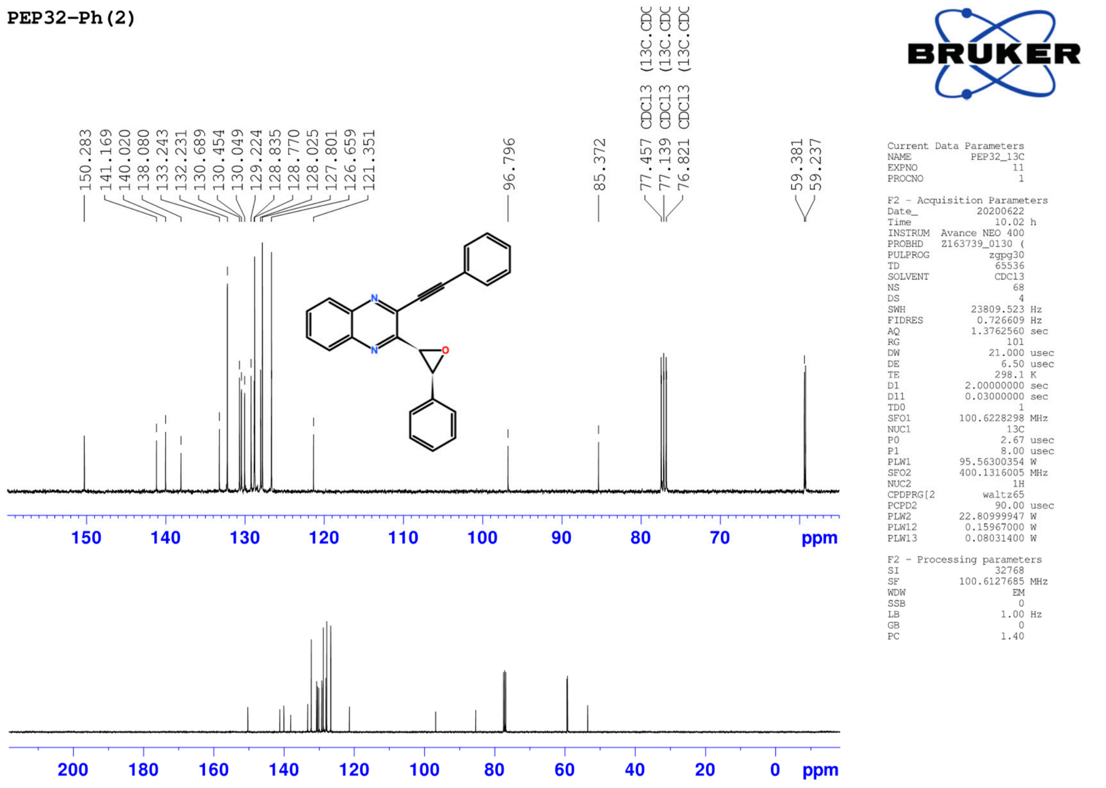

```
Data File C:\Chem32\...rganique\prestation_chimie_organique 2022-05-31\OnlineEdited--029.D
Sample Name: PEP32

    =====================================================================
    Acq. Operator   : SYSTEM                          Seq. Line :  29
    Acq. Instrument : LC1290                           Location :   P1-D1
    Injection Date  : 31/5/2022 8:34:27 PM                  Inj :   1
                                                     Inj Volume : 1.000 µl
    Method          : C:\Chem32\1\Data\prestation_chimie_organique\prestation_chimie_organique
                      2022-05-31\prestation_chimie_org.M (Sequence Method)
    Last changed    : 31/5/2022 1:07:56 PM by SYSTEM
    Additional Info : Peak(s) manually integrated
```

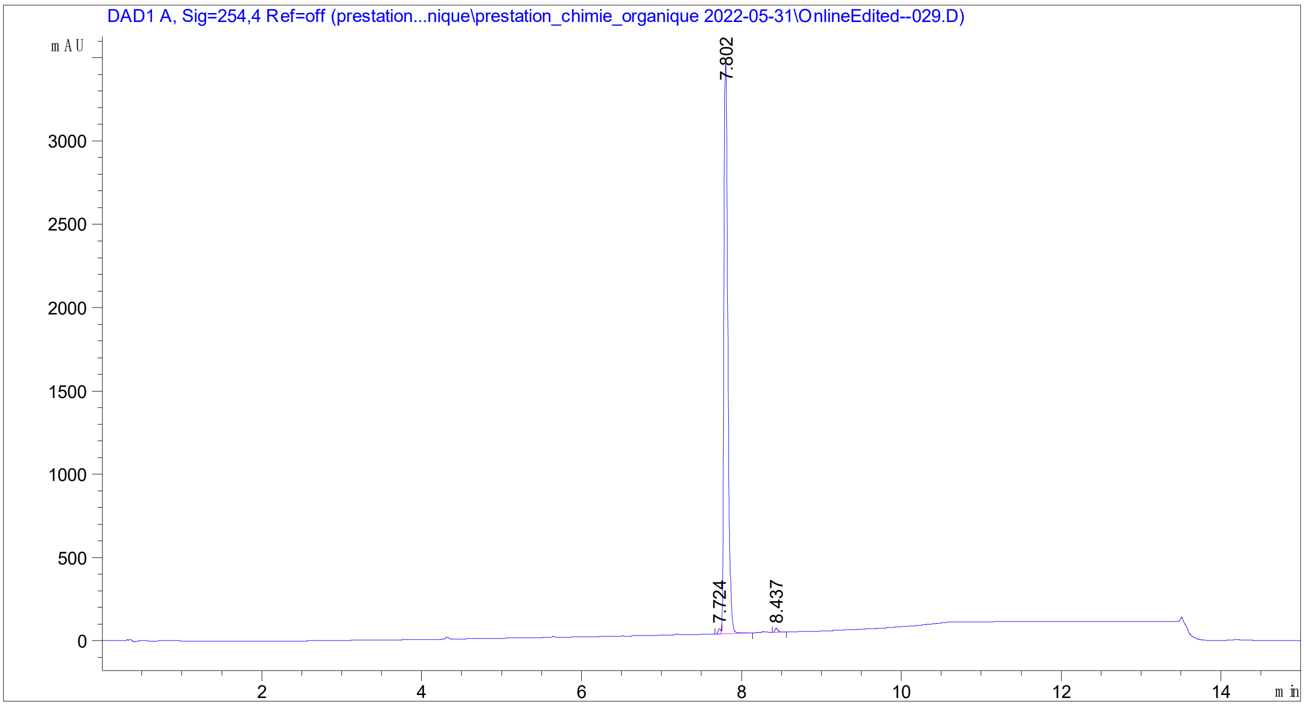


```
    =====================================================================
                             Area Percent Report
    =====================================================================

    Sorted By             :      Signal
    Multiplier            :      1.0000
    Dilution              :      1.0000
    Use Multiplier & Dilution Factor with ISTDs


    Signal 1: DAD1 A, Sig=254,4 Ref=off

    Peak RetTime Type  Width     Area      Height     Area
      #   [min]        [min]   [mAU*s]     [mAU]        %
    ----|-------|----|-------|----------|----------|--------|
       1   7.724 BV E  0.0311   66.60098   32.21184   0.5599
       2   7.802 VV R  0.0542 1.17595e4  3415.82910  98.8665
       3   8.437 BB    0.0407   68.22112   25.11445   0.5736

    Totals :                  1.18943e4  3473.15539


    =====================================================================
                              *** End of Report ***
```

Compound 15a

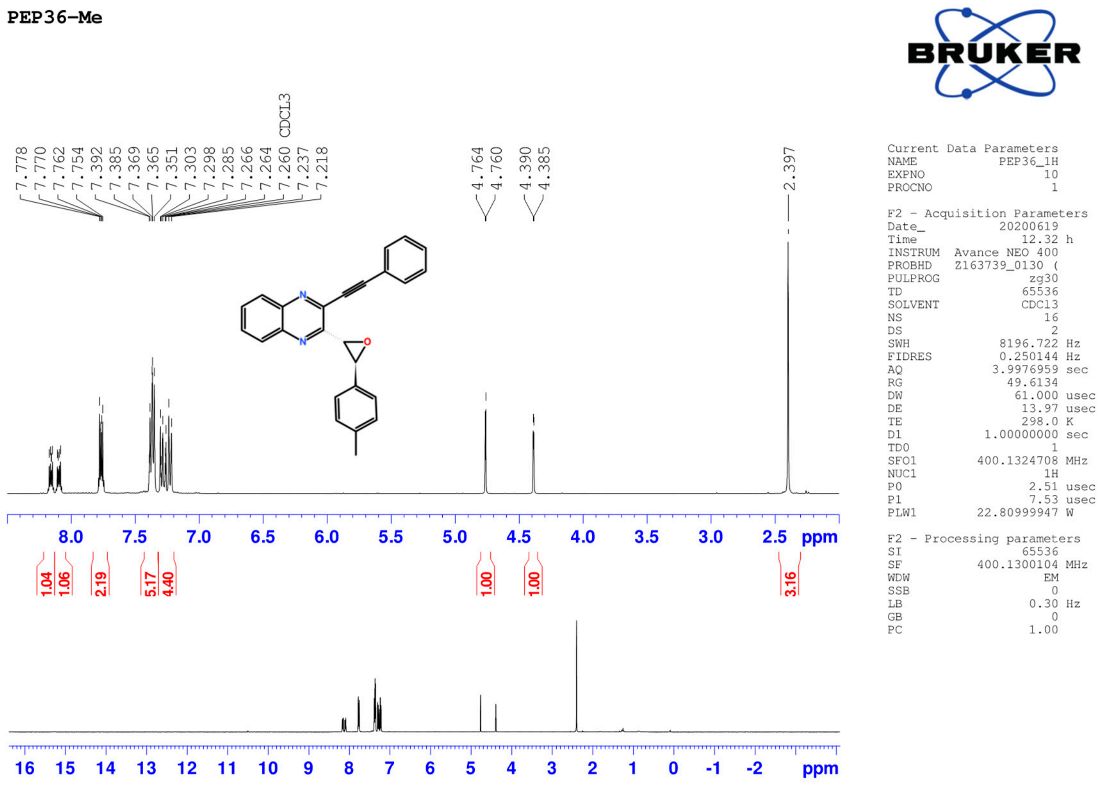

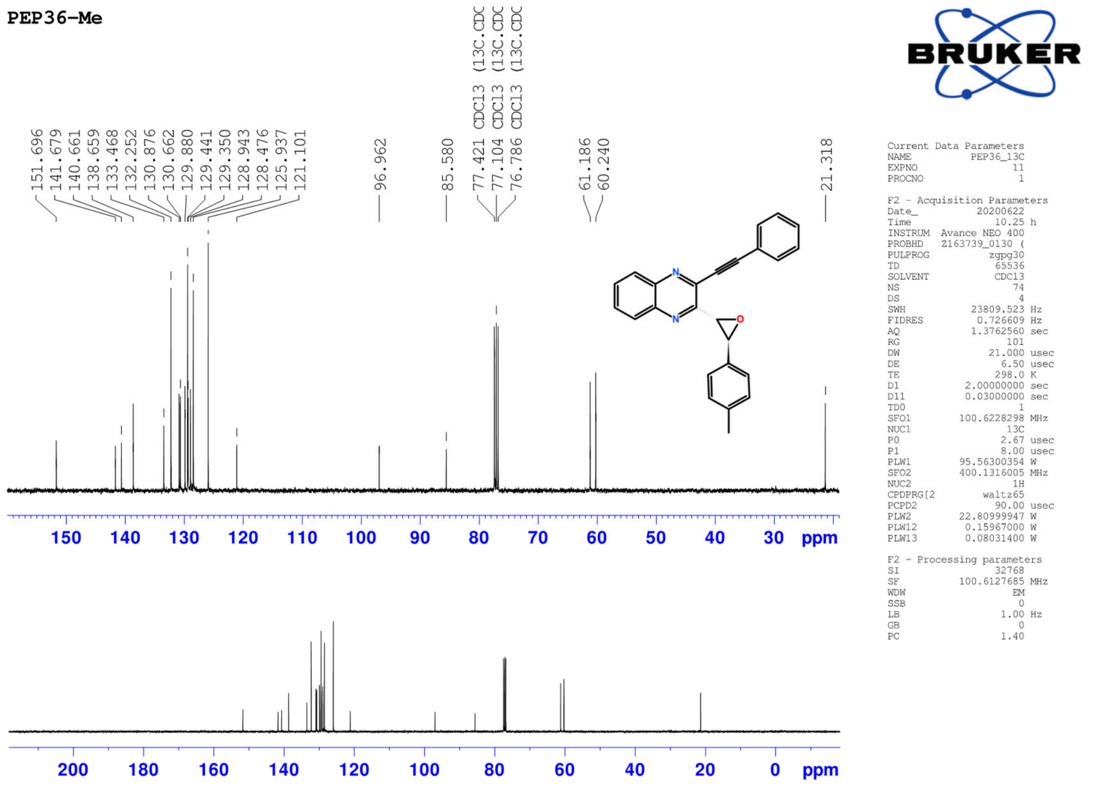

Data File C:\Chem32\...rganique\prestation_chimie_organique 2022-05-31\OnlineEdited--033.D
Sample Name: PEP36

===

Acq. Operator : SYSTEM Seq. Line : 33
Acq. Instrument : LC1290 Location : P1-D5
Injection Date : 31/5/2022 9:37:33 PM Inj : 1
Inj Volume : 1.000 µl
Method : C:\Chem32\1\Data\prestation_chimie_organique\prestation_chimie_organique 2022-05-31\prestation_chimie_org.M (Sequence Method)
Last changed : 31/5/2022 1:07:56 PM by SYSTEM
Additional Info : Peak(s) manually integrated

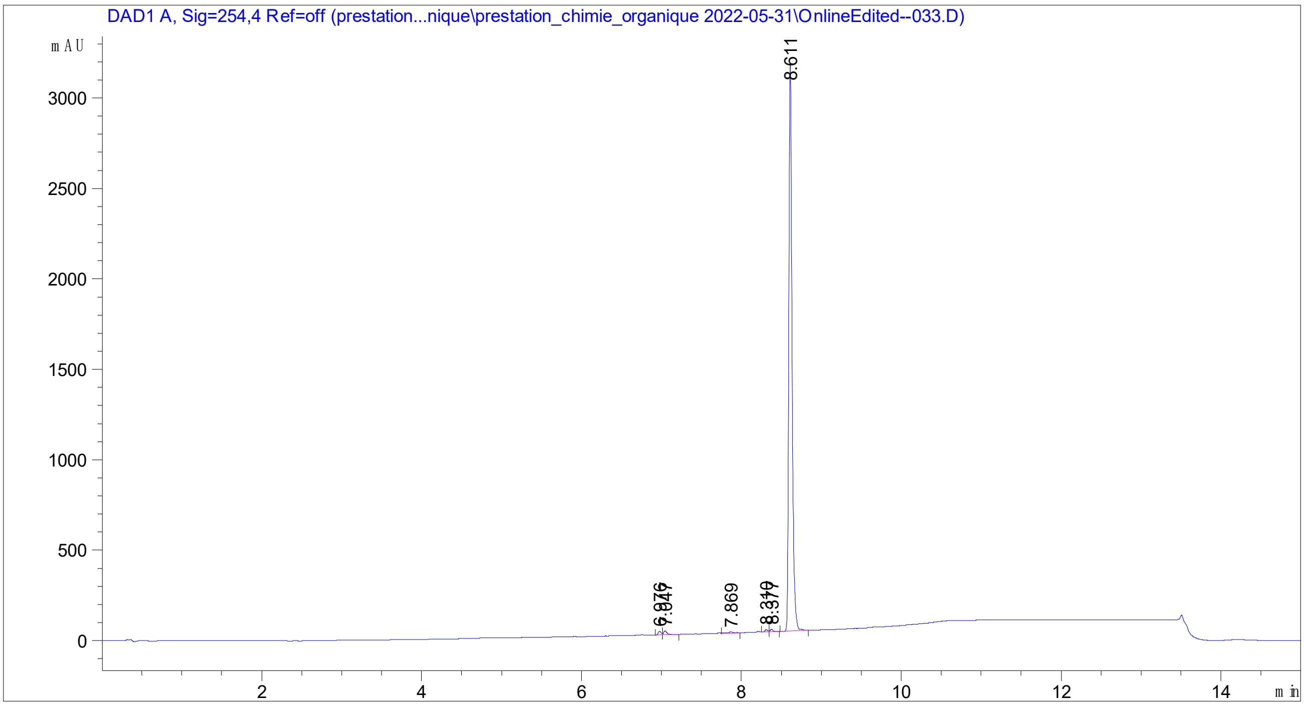

===

Area Percent Report

===

Sorted By : Signal
Multiplier : 1.0000
Dilution : 1.0000
Use Multiplier & Dilution Factor with ISTDs

Signal 1: DAD1 A, Sig=254,4 Ref=off

Peak #	RetTime [min]	Type	Width [min]	Area [mAU*s]	Height [mAU]	Area %
1	6.976	BV	0.0355	48.41981	20.56596	0.5397
2	7.047	VV R	0.0431	63.91540	21.88852	0.7124
3	7.869	BV R	0.0418	21.84160	7.54534	0.2434
4	8.310	VV	0.0395	31.09993	11.91477	0.3466
5	8.377	VB	0.0430	38.39138	13.18491	0.4279
6	8.611	BV R	0.0436	8768.56152	3133.93701	97.7300

Totals : 8972.22963 3209.03651

Compound 16a

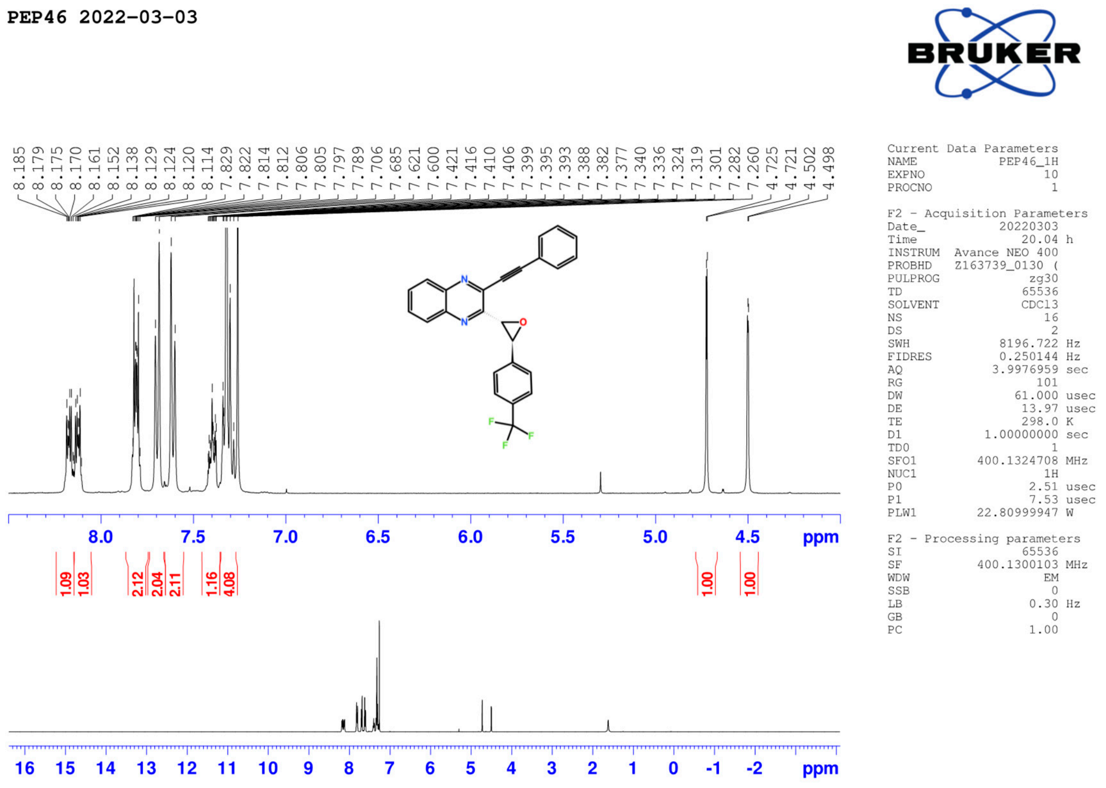

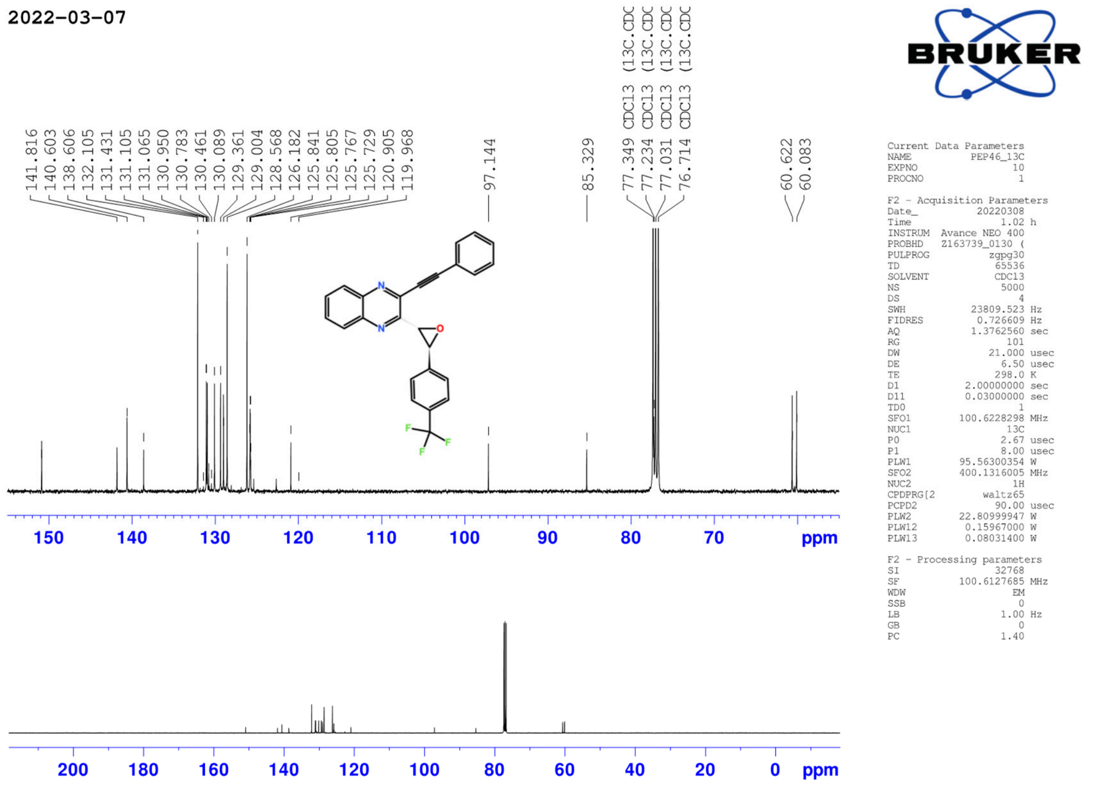

```
Data File C:\Chem32\...rganique\prestation_chimie_organique 2022-05-31\OnlineEdited--037.D
Sample Name: PEP46

    =====================================================================
    Acq. Operator   : SYSTEM                          Seq. Line :  37
    Acq. Instrument : LC1290                           Location :   P1-D9
    Injection Date  : 31/5/2022 10:40:39 PM                 Inj :   1
                                                     Inj Volume : 1.000 µl
    Method          : C:\Chem32\1\Data\prestation_chimie_organique\prestation_chimie_organique
                      2022-05-31\prestation_chimie_org.M (Sequence Method)
    Last changed    : 31/5/2022 1:07:56 PM by SYSTEM
    Additional Info : Peak(s) manually integrated
```

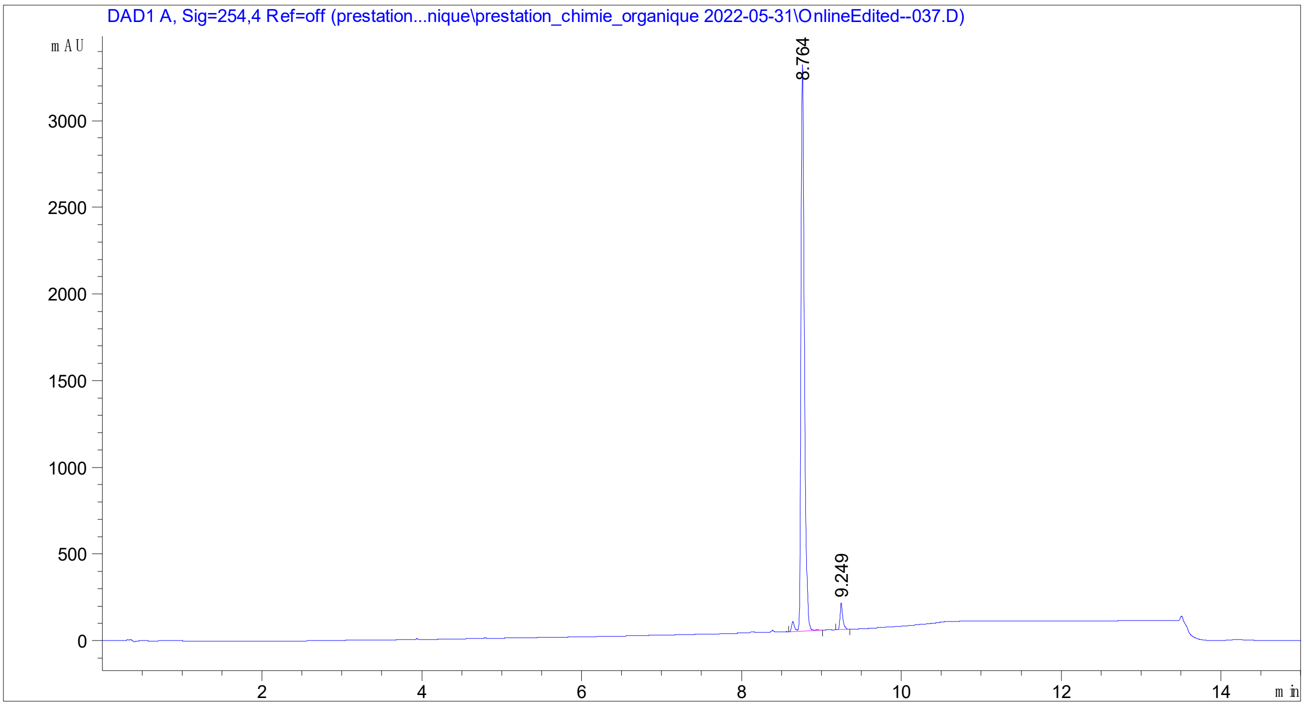


```
    =====================================================================
                             Area Percent Report
    =====================================================================

    Sorted By             :      Signal
    Multiplier            :      1.0000
    Dilution              :      1.0000
    Use Multiplier & Dilution Factor with ISTDs


    Signal 1: DAD1 A, Sig=254,4 Ref=off

    Peak RetTime Type  Width      Area      Height     Area
      #   [min]        [min]    [mAU*s]     [mAU]        %
    ----|-------|----|-------|----------|----------|--------|
       1   8.764 VV R  0.0447 9592.59277 3268.00439  96.1767
       2   9.249 BB    0.0366  381.33215  155.64145   3.8233

    Totals :                  9973.92493 3423.64584


    =====================================================================
                              *** End of Report ***
```

Compound 16b

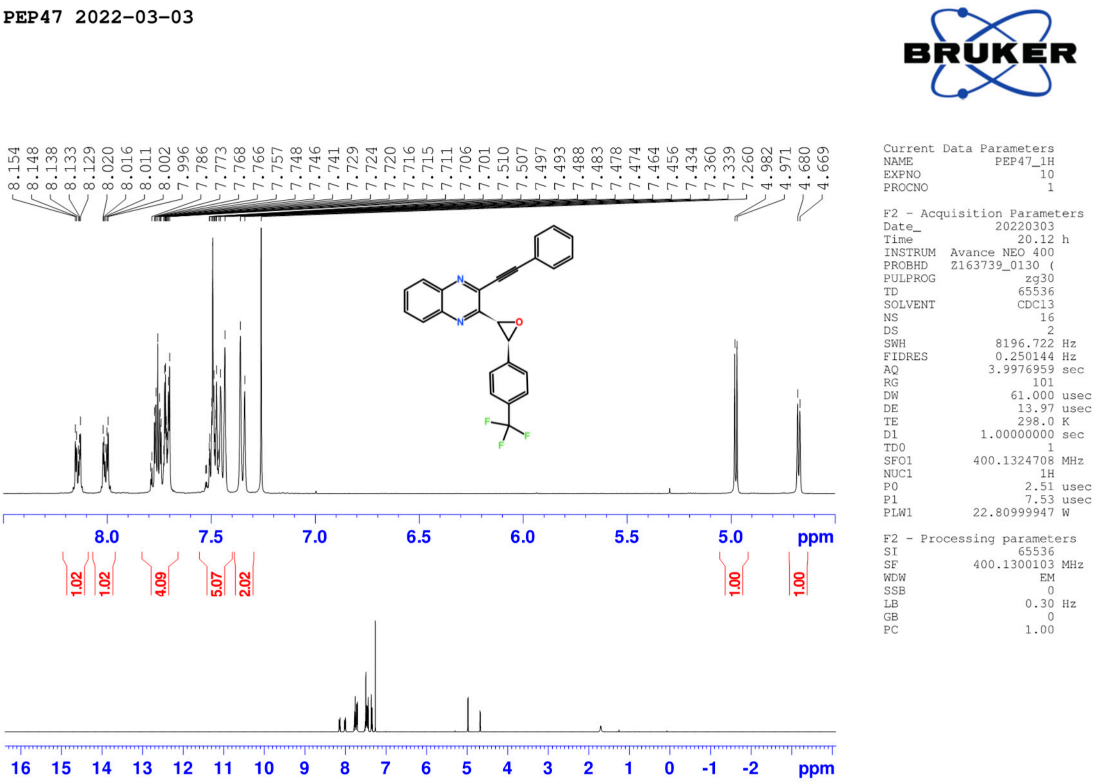

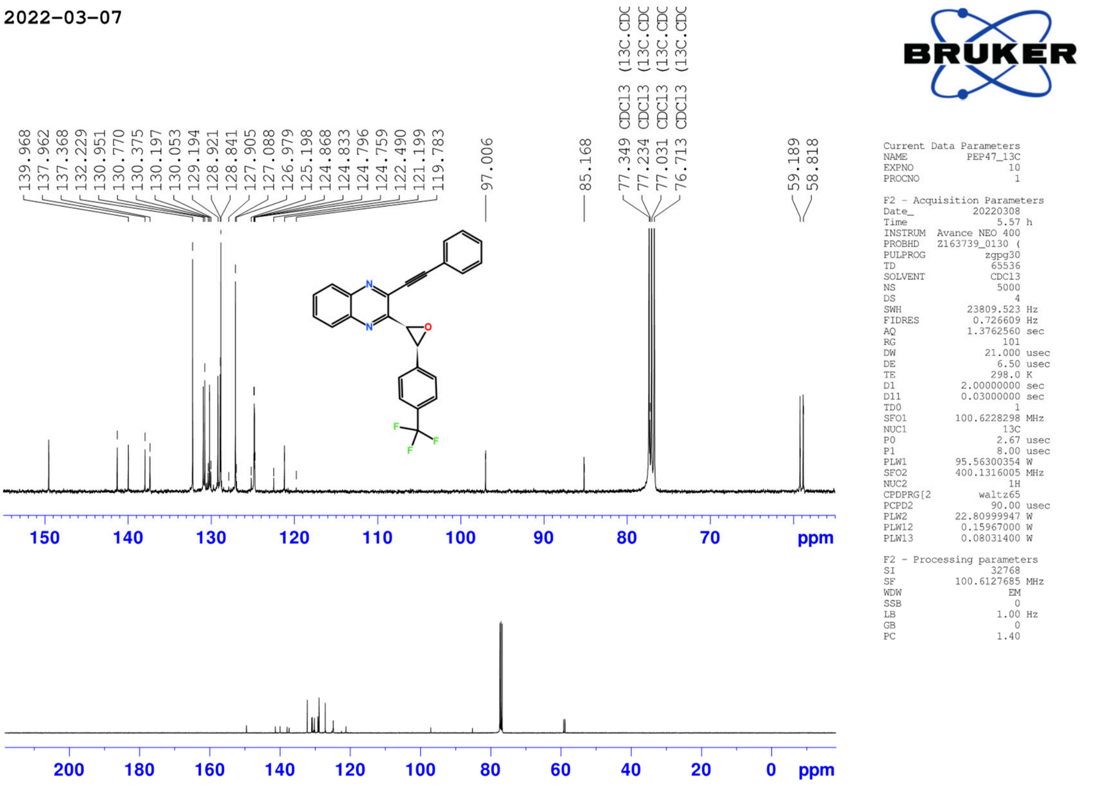

```
Data File C:\Chem32\...rganique\prestation_chimie_organique 2022-05-31\OnlineEdited--038.D
Sample Name: PEP47

    =====================================================================
    Acq. Operator   : SYSTEM                          Seq. Line :  38
    Acq. Instrument : LC1290                           Location :   P1-E1
    Injection Date  : 31/5/2022 10:56:24 PM                 Inj :   1
                                                      Inj Volume : 1.000 µl
    Method          : C:\Chem32\1\Data\prestation_chimie_organique\prestation_chimie_organique
                      2022-05-31\prestation_chimie_org.M (Sequence Method)
    Last changed    : 31/5/2022 1:07:56 PM by SYSTEM
    Additional Info : Peak(s) manually integrated
```

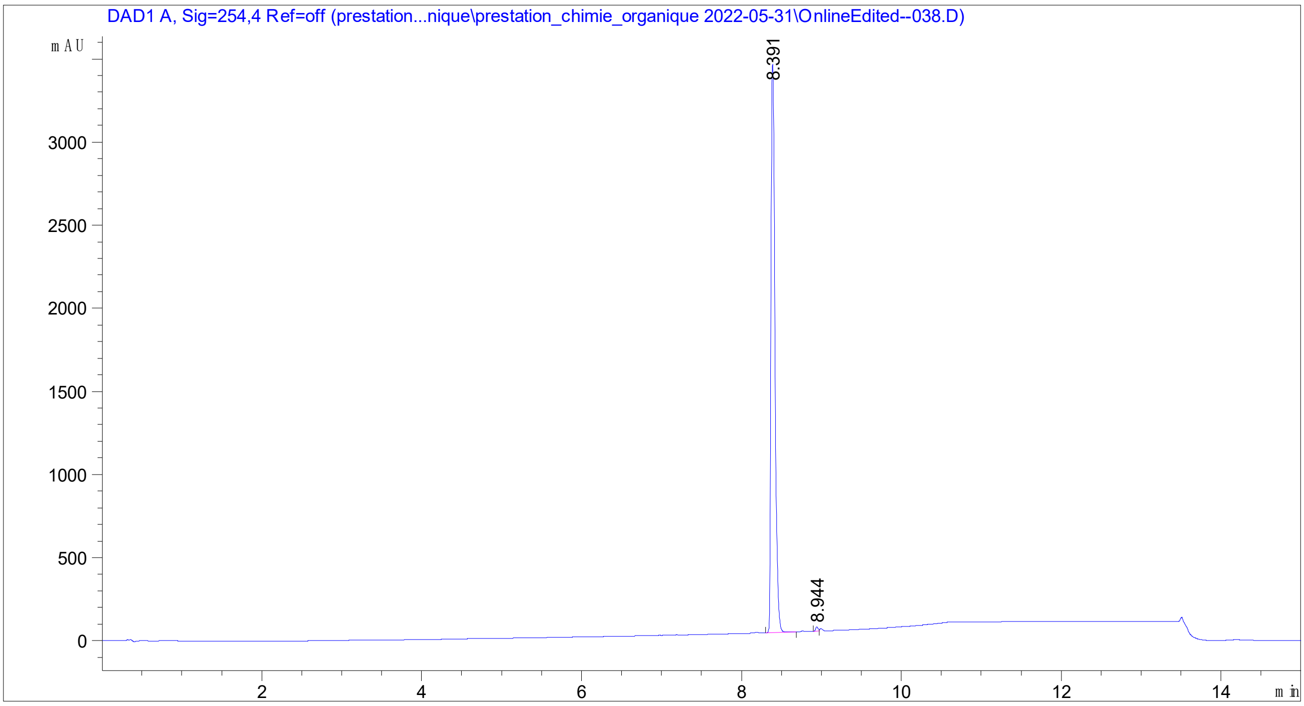


```
    =====================================================================
                             Area Percent Report
    =====================================================================

    Sorted By             :      Signal
    Multiplier            :      1.0000
    Dilution              :      1.0000
    Use Multiplier & Dilution Factor with ISTDs


    Signal 1: DAD1 A, Sig=254,4 Ref=off

    Peak RetTime Type  Width     Area      Height     Area
      #   [min]        [min]   [mAU*s]     [mAU]        %
    ----|-------|----|-------|----------|----------|--------|
       1   8.391 BV R  0.0553 1.21017e4  3417.00098  99.5291
       2   8.944 BV    0.0337   57.26093   26.04416   0.4709

    Totals :                  1.21589e4  3443.04514


    =====================================================================
                             *** End of Report ***
```

Compound 17a

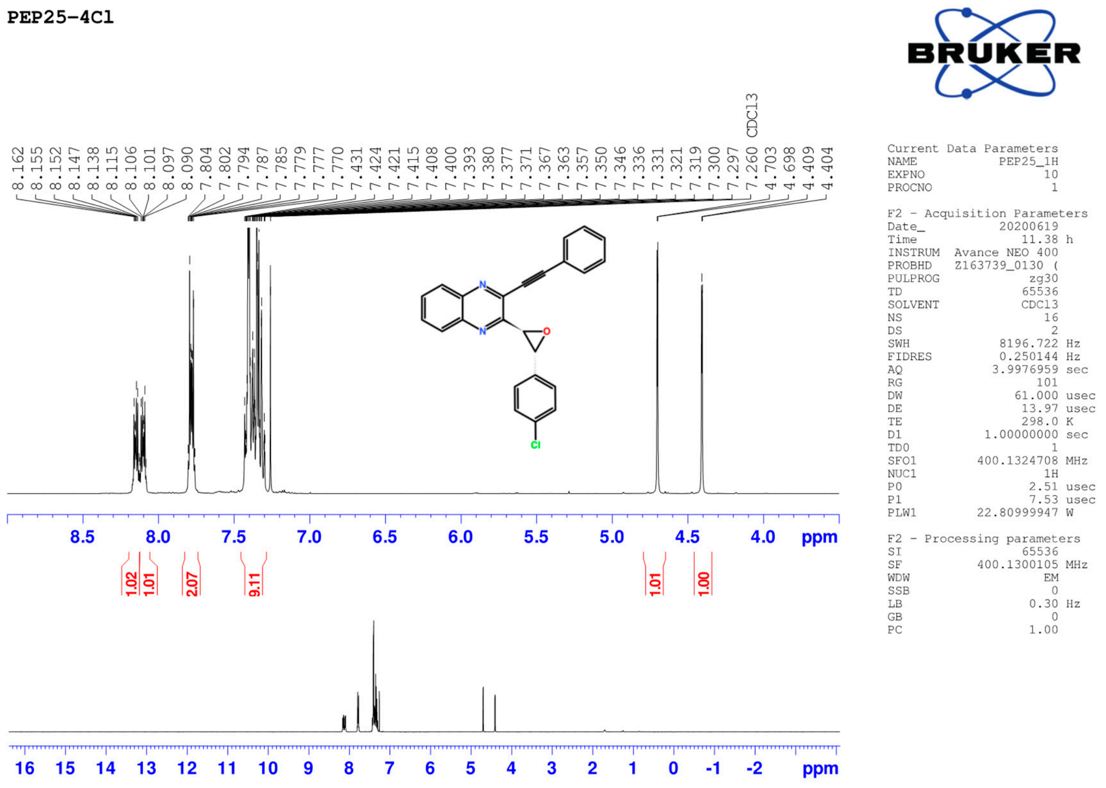

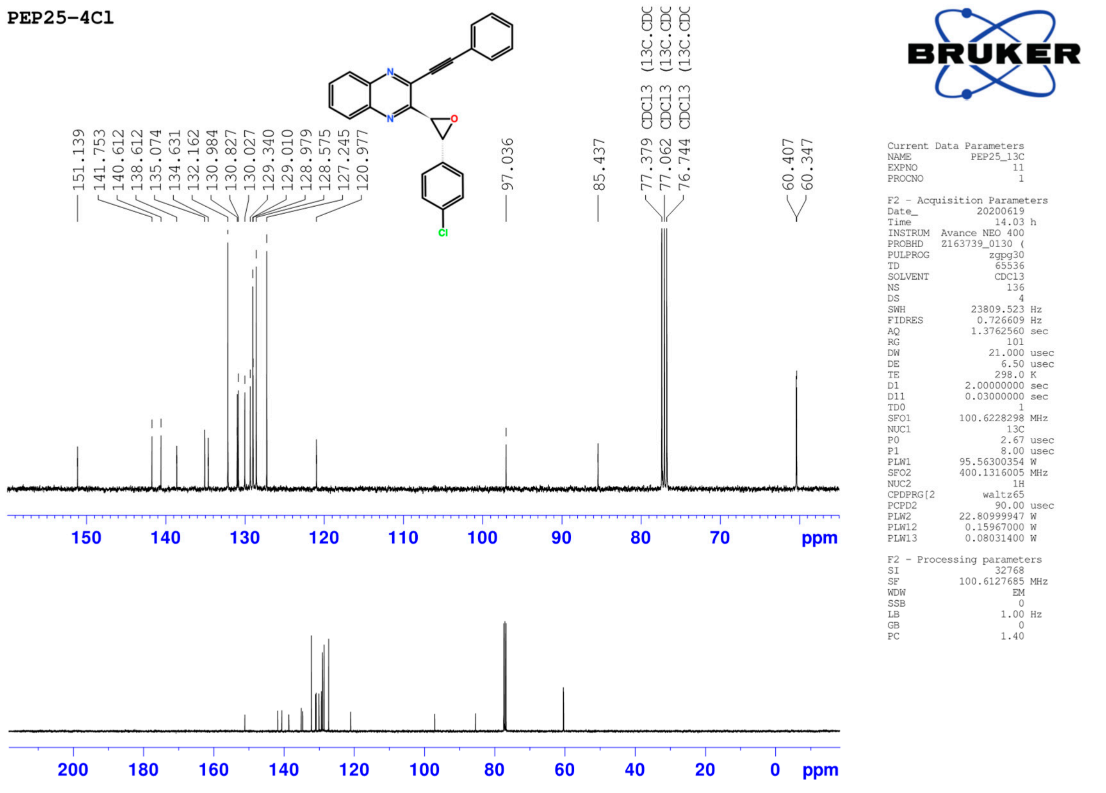

Data File C:\Chem32\...rganique\prestation_chimie_organique 2022-05-31\OnlineEdited--023.D
Sample Name: PEP25

===

Acq. Operator : SYSTEM Seq. Line : 23
Acq. Instrument : LC1290 Location : P1-C4
Injection Date : 31/5/2022 6:59:40 PM Inj : 1
Inj Volume : 1.000 µl
Method : C:\Chem32\1\Data\prestation_chimie_organique\prestation_chimie_organique 2022-05-31\prestation_chimie_org.M (Sequence Method)
Last changed : 31/5/2022 1:07:56 PM by SYSTEM
Additional Info : Peak(s) manually integrated

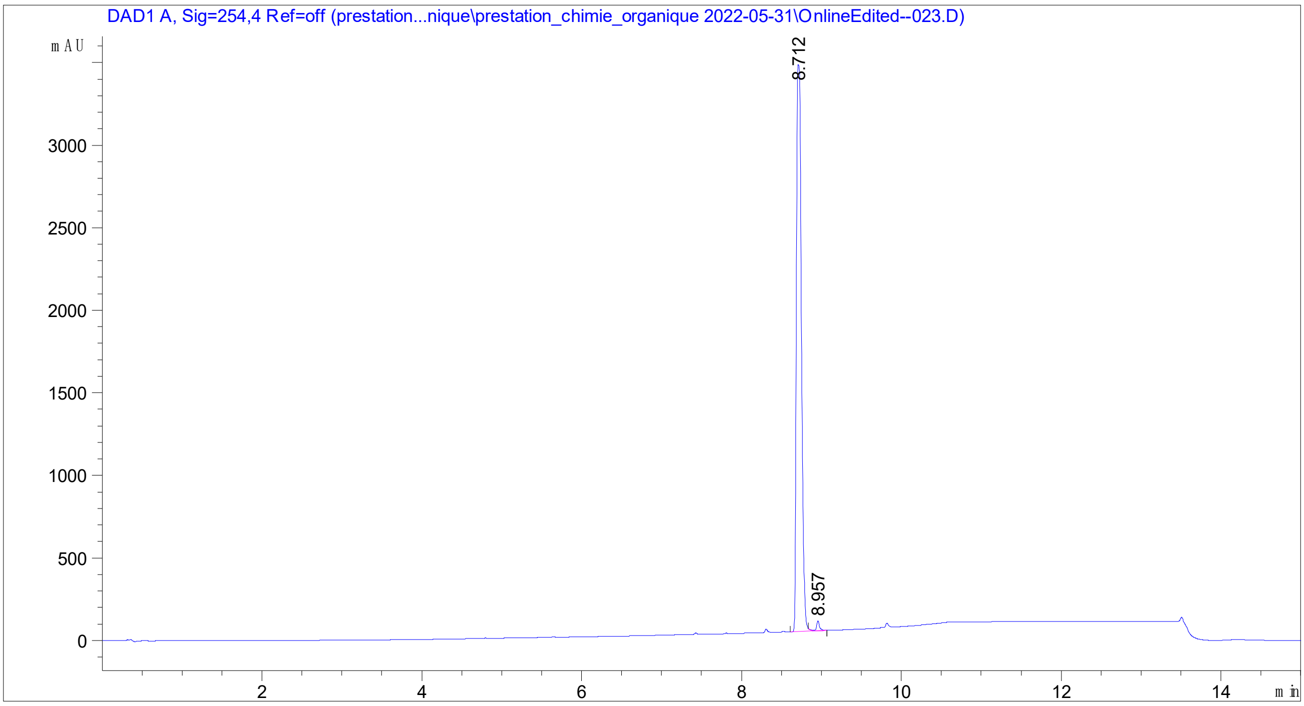

===

Area Percent Report

===

Sorted By : Signal
Multiplier : 1.0000
Dilution : 1.0000
Use Multiplier & Dilution Factor with ISTDs

Signal 1: DAD1 A, Sig=254,4 Ref=off

Peak #	RetTime [min]	Type	Width [min]	Area [mAU*s]	Height [mAU]	Area %
1	8.712	BV R	0.0699	1.52815e4	3433.02393	98.9401
2	8.957	VB E	0.0405	163.70430	58.79041	1.0599

Totals : 1.54452e4 3491.81433

===

*** End of Report ***

Compound 17b

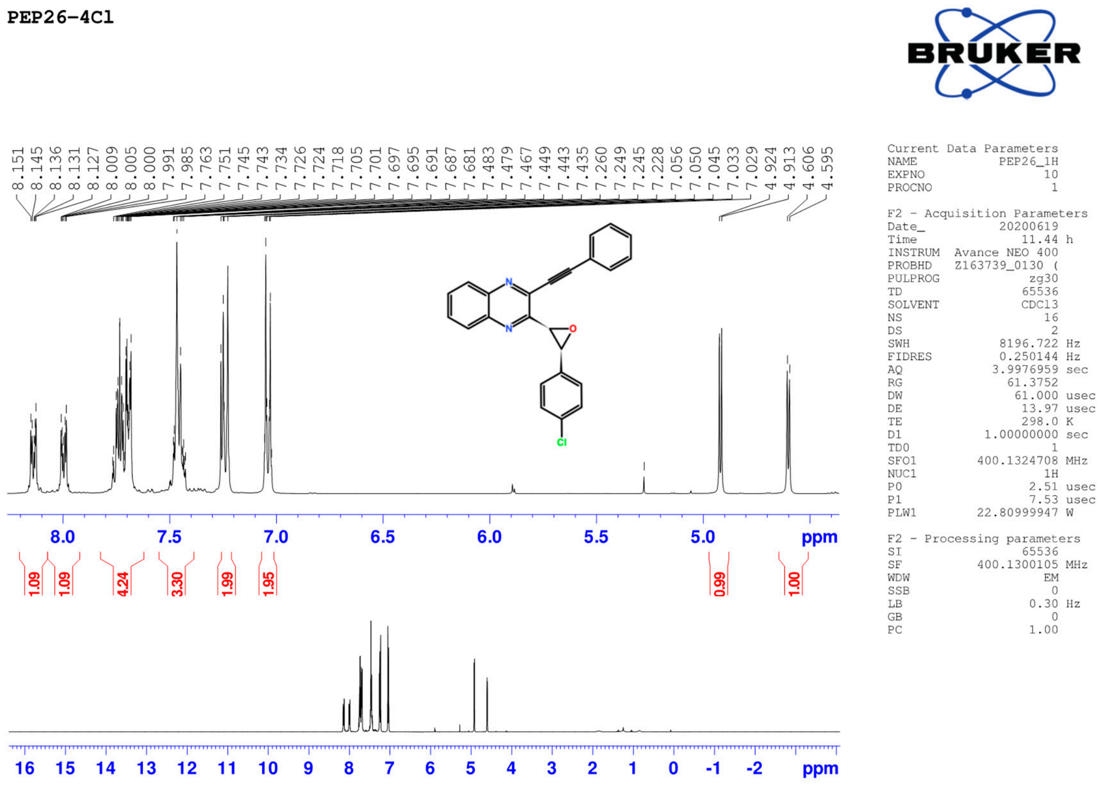

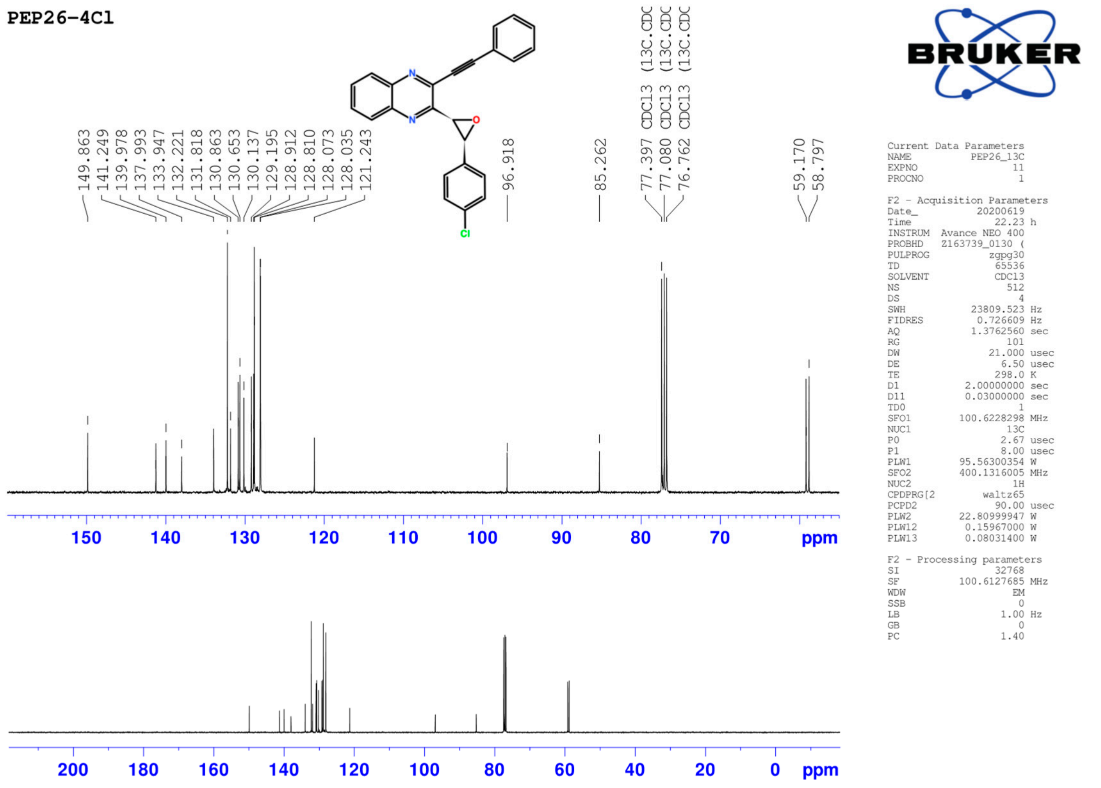

Data File C:\Chem32\...rganique\prestation_chimie_organique 2022-05-31\OnlineEdited--024.D
Sample Name: PEP26

===
Acq. Operator : SYSTEM Seq. Line : 24
Acq. Instrument : LC1290 Location : P1-C5
Injection Date : 31/5/2022 7:15:28 PM Inj : 1
Inj Volume : 1.000 µl
Method : C:\Chem32\1\Data\prestation_chimie_organique\prestation_chimie_organique 2022-05-31\prestation_chimie_org.M (Sequence Method)
Last changed : 31/5/2022 1:07:56 PM by SYSTEM
Additional Info : Peak(s) manually integrated

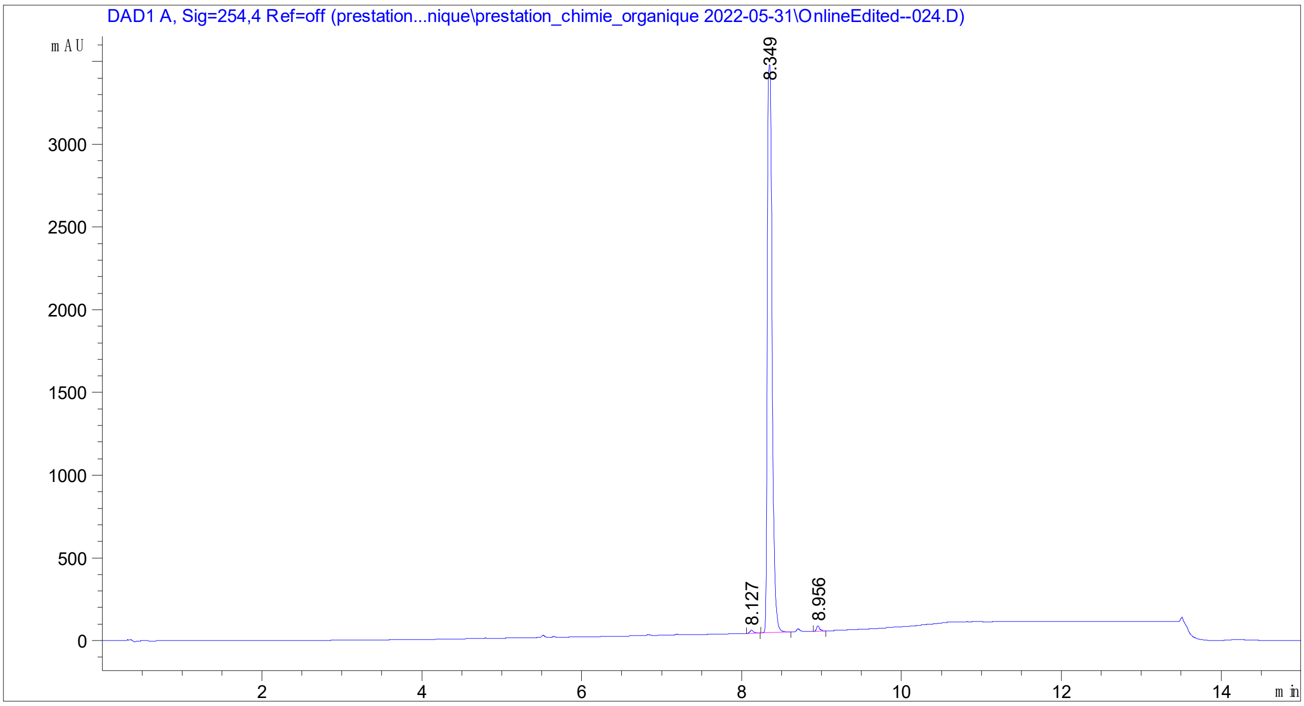

===
Area Percent Report
===

Sorted By : Signal
Multiplier : 1.0000
Dilution : 1.0000
Use Multiplier & Dilution Factor with ISTDs

Signal 1: DAD1 A, Sig=254,4 Ref=off

Peak #	RetTime [min]	Type	Width [min]	Area [mAU*s]	Height [mAU]	Area %
1	8.127	BB	0.0455	56.93033	18.73463	0.3922
2	8.349	BV R	0.0659	1.43769e4	3432.73145	99.0429
3	8.956	BB	0.0375	81.99546	32.43653	0.5649

Totals : 1.45158e4 3483.90261

===
*** End of Report ***

Compound 18a

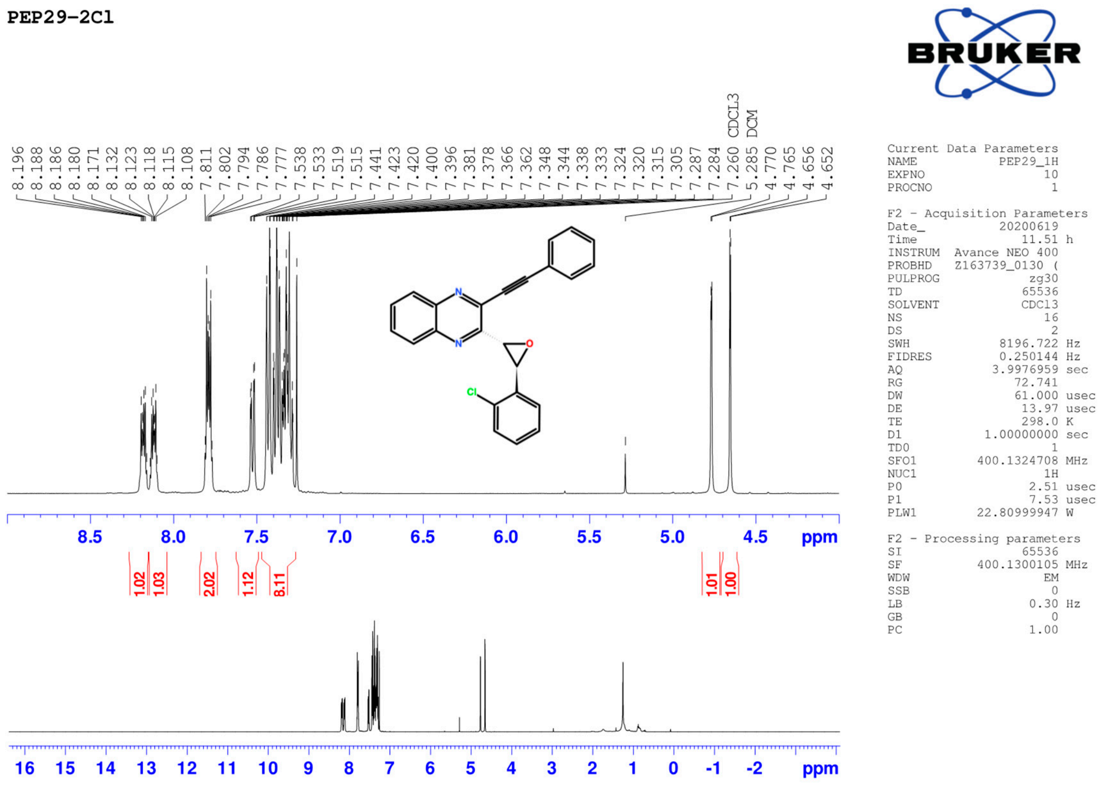

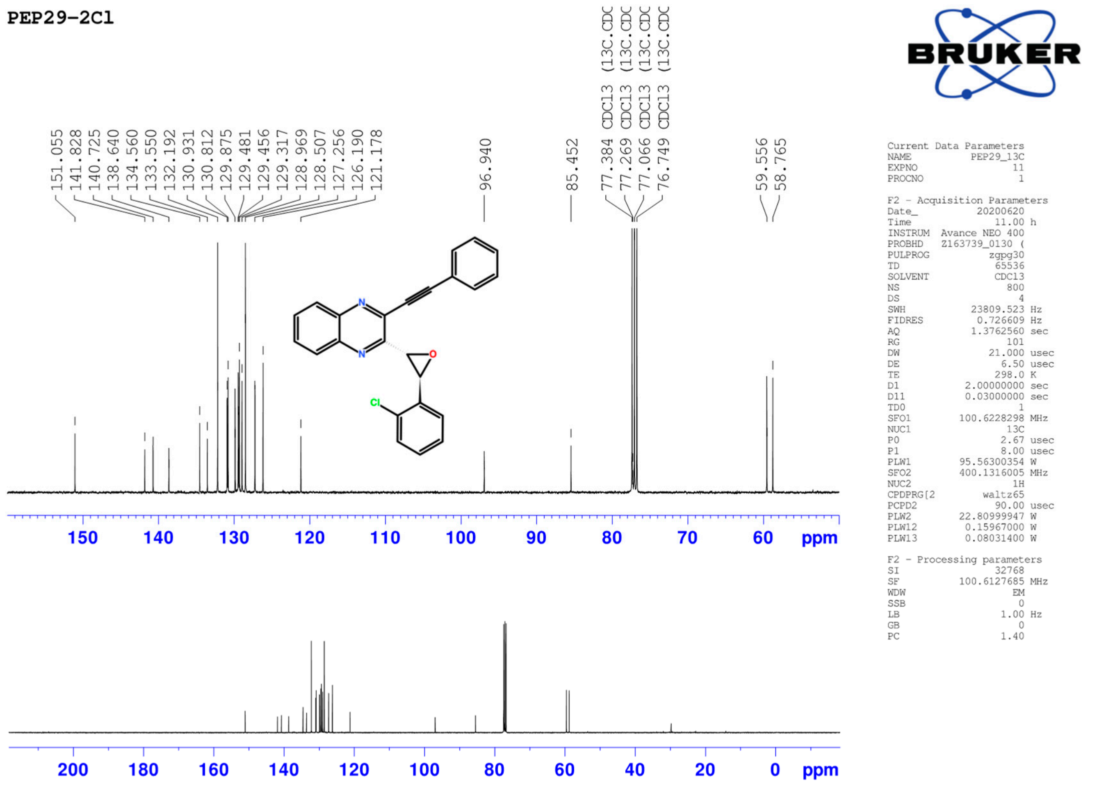

Data File C:\Chem32\...rganique\prestation_chimie_organique 2022-05-31\OnlineEdited--027.D
Sample Name: PEP29

===

Acq. Operator : SYSTEM Seq. Line : 27
Acq. Instrument : LC1290 Location : P1-C8
Injection Date : 31/5/2022 8:02:51 PM Inj : 1
Inj Volume : 1.000 µl
Method : C:\Chem32\1\Data\prestation_chimie_organique\prestation_chimie_organique 2022-05-31\prestation_chimie_org.M (Sequence Method)
Last changed : 31/5/2022 1:07:56 PM by SYSTEM
Additional Info : Peak(s) manually integrated

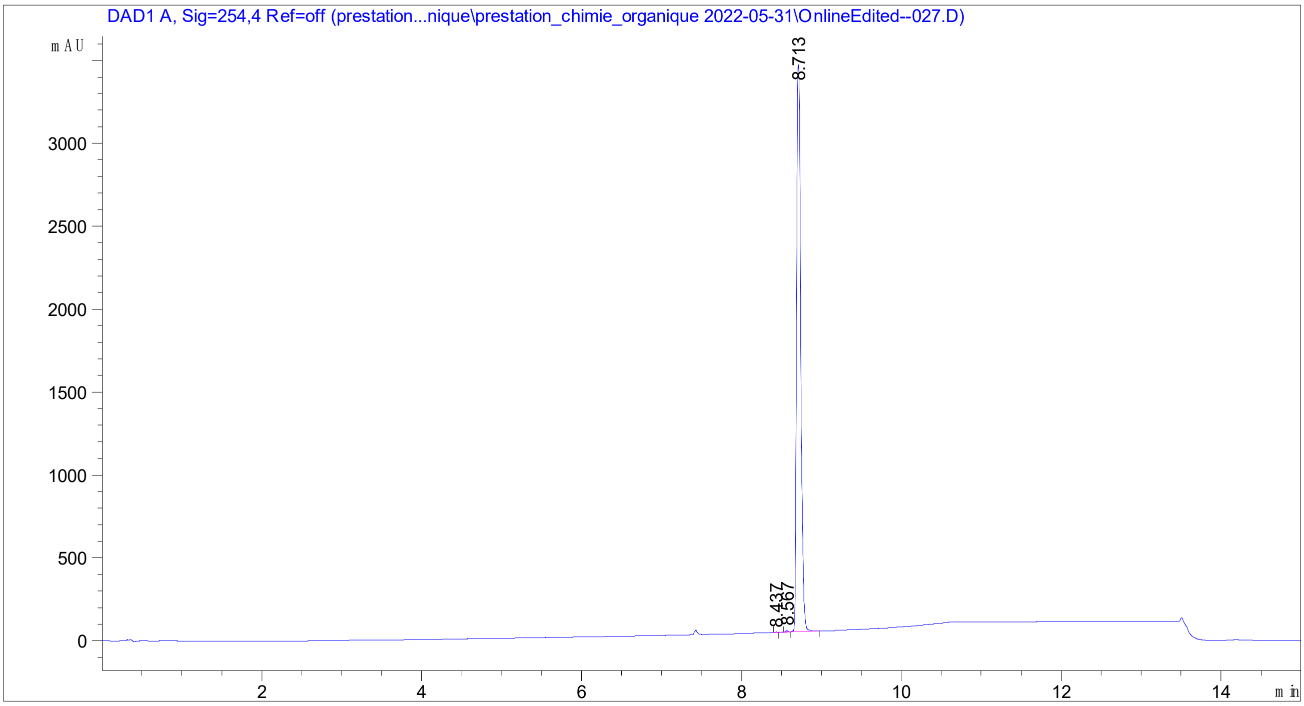

===

Area Percent Report

===

Sorted By : Signal
Multiplier : 1.0000
Dilution : 1.0000
Use Multiplier & Dilution Factor with ISTDs

Signal 1: DAD1 A, Sig=254,4 Ref=off

Peak #	RetTime [min]	Type	Width [min]	Area [mAU*s]	Height [mAU]	Area %
1	8.437	BV E	0.0319	5.31774	2.60060	0.0432
2	8.567	VV E	0.0336	21.93933	9.98431	0.1781
3	8.713	VB R	0.0570	1.22946e4	3418.70166	99.7788

Totals : 1.23218e4 3431.28657

===

*** End of Report ***

LC1290 1/6/2022 5:50:34 PM SYSTEM Page 1 of 1

Compound 19a

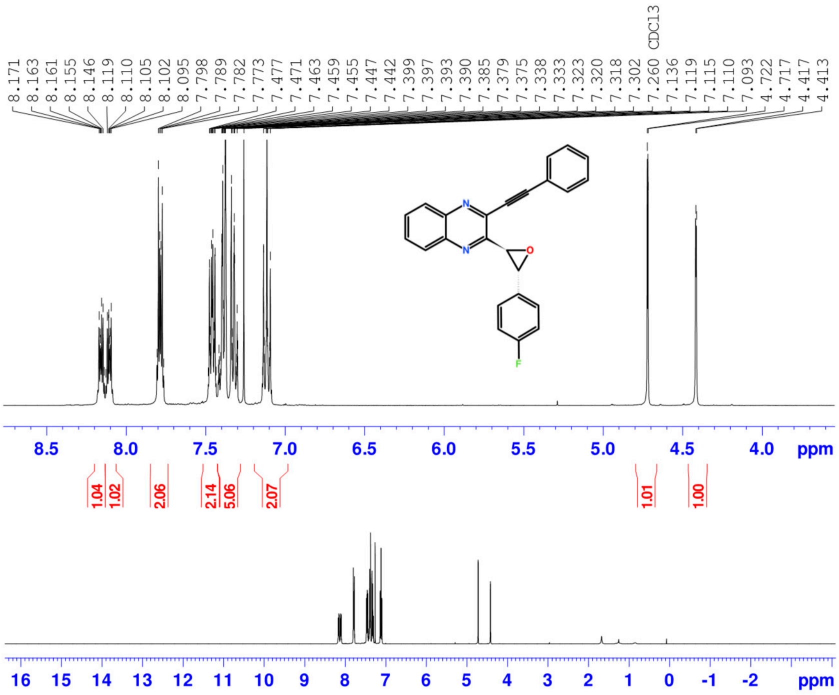


```
Current Data Parameters
NAME            PEP27_1H
EXPNO                 10
PROCNO                 1

F2 - Acquisition Parameters
Date_           20200619
Time               15.22 h
INSTRUM   Avance NEO 400
PROBHD   Z163739_0130 (
PULPROG             zg30
TD                 65536
SOLVENT            CDCl3
NS                    16
DS                     2
SWH             8196.722 Hz
FIDRES          0.250144 Hz
AQ             3.9976959 sec
RG                   101
DW                61.000 usec
DE                 13.97 usec
TE                 298.0 K
D1            1.00000000 sec
TD0                    1
SFO1         400.1324708 MHz
NUC1                  1H
P0                  2.51 usec
P1                  7.53 usec
PLW1         22.80999947 W

F2 - Processing parameters
SI                 65536
SF           400.1300104 MHz
WDW                   EM
SSB                    0
LB                  0.30 Hz
GB                     0
PC                  1.00
```

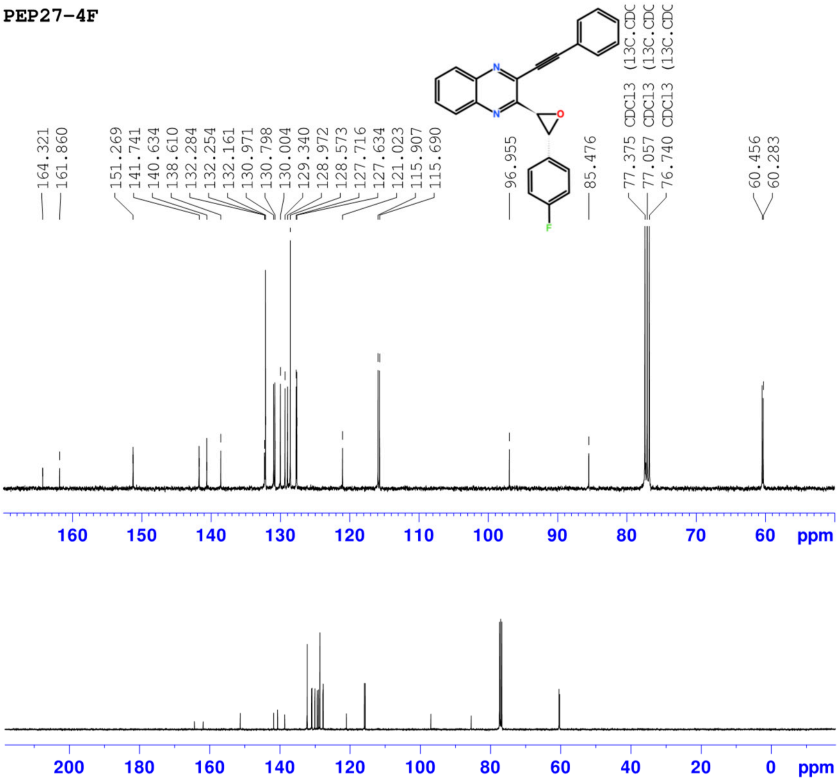


```
Current Data Parameters
NAME            PEP27_13C
EXPNO                 11
PROCNO                 1

F2 - Acquisition Parameters
Date_           20200622
Time               12.10 h
INSTRUM   Avance NEO 400
PROBHD   Z163739_0130 (
PULPROG           zgpg30
TD                 65536
SOLVENT            CDCl3
NS                   350
DS                     4
SWH            23809.523 Hz
FIDRES          0.726609 Hz
AQ             1.3762560 sec
RG                   101
DW                21.000 usec
DE                  6.50 usec
TE                 298.0 K
D1            2.00000000 sec
D11           0.03000000 sec
TD0                    1
SFO1         100.6228298 MHz
NUC1                 13C
P0                  2.67 usec
P1                  8.00 usec
PLW1         95.56300354 W
SFO2         400.1316005 MHz
NUC2                  1H
CPDPRG[2         waltz65
PCPD2              90.00 usec
PLW2         22.80999947 W
PLW12         0.15967000 W
PLW13         0.08031400 W

F2 - Processing parameters
SI                 32768
SF           100.6127685 MHz
WDW                   EM
SSB                    0
LB                  1.00 Hz
GB                     0
PC                  1.40
```

```
Data File C:\Chem32\...rganique\prestation_chimie_organique 2022-05-31\OnlineEdited--025.D
Sample Name: PEP27

=====================================================================
Acq. Operator   : SYSTEM                          Seq. Line :  25
Acq. Instrument : LC1290                           Location :   P1-C6
Injection Date  : 31/5/2022 7:31:17 PM                  Inj :   1
                                                 Inj Volume : 1.000 µl
Method          : C:\Chem32\1\Data\prestation_chimie_organique\prestation_chimie_organique
                  2022-05-31\prestation_chimie_org.M (Sequence Method)
Last changed    : 31/5/2022 1:07:56 PM by SYSTEM
Additional Info : Peak(s) manually integrated
```

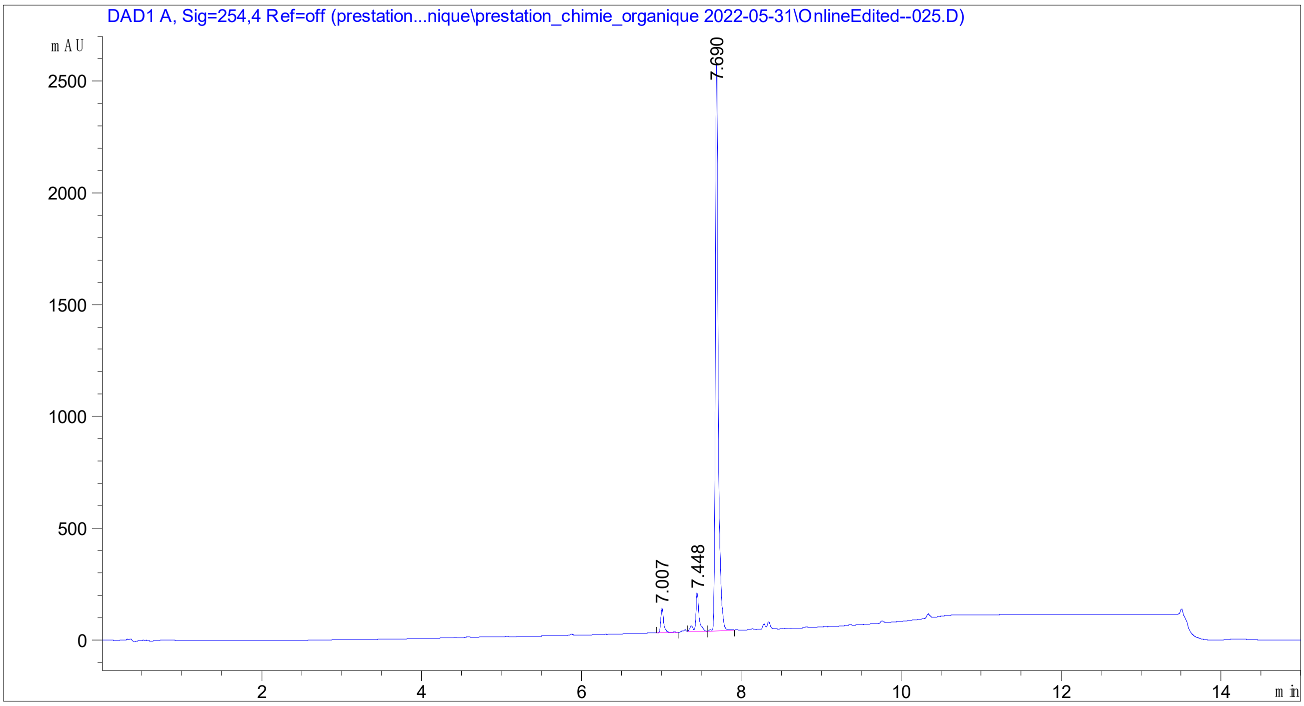


```
=====================================================================
                         Area Percent Report
=====================================================================

Sorted By             :      Signal
Multiplier            :      1.0000
Dilution              :      1.0000
Use Multiplier & Dilution Factor with ISTDs


Signal 1: DAD1 A, Sig=254,4 Ref=off

Peak RetTime Type  Width     Area      Height     Area
  #   [min]        [min]   [mAU*s]     [mAU]        %
----|-------|----|-------|----------|----------|--------|
   1   7.007 BV R  0.0396  292.25009  108.85187   3.9192
   2   7.448 VB R  0.0396  520.73712  170.16032   6.9834
   3   7.690 VV R  0.0395 6643.84668 2529.45068  89.0974

Totals :                  7456.83389 2808.46288


=====================================================================
                          *** End of Report ***
```

Compound 19b

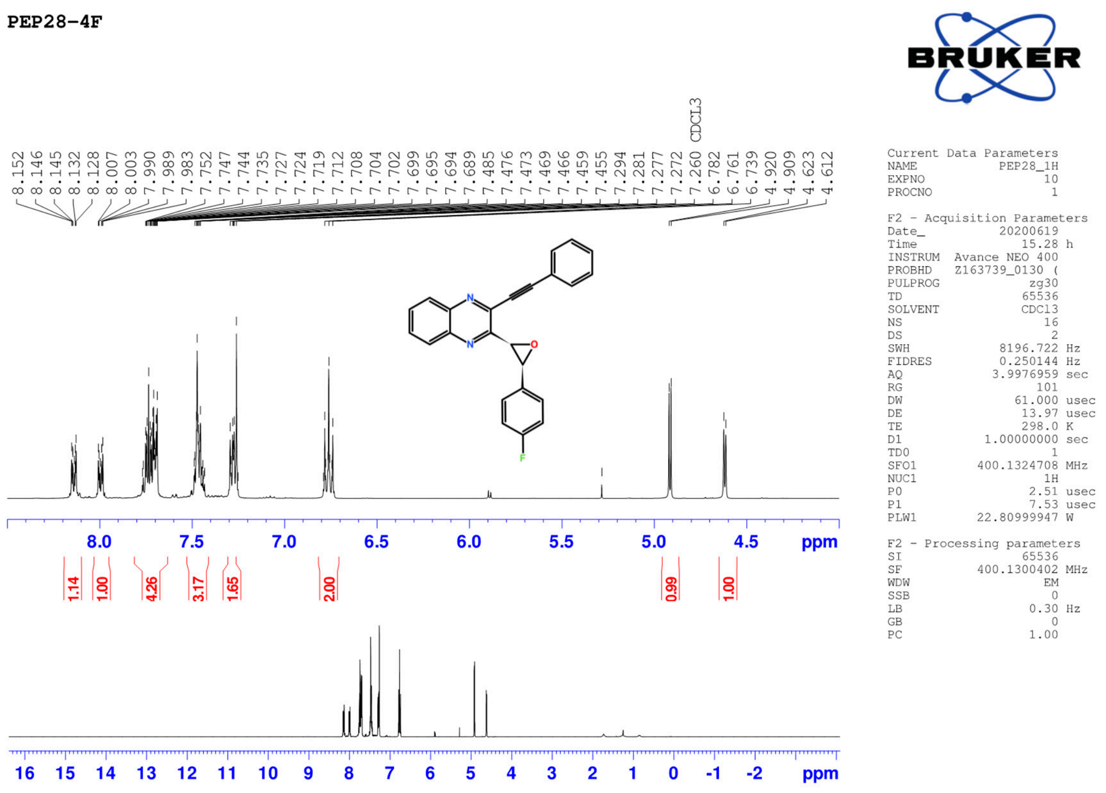

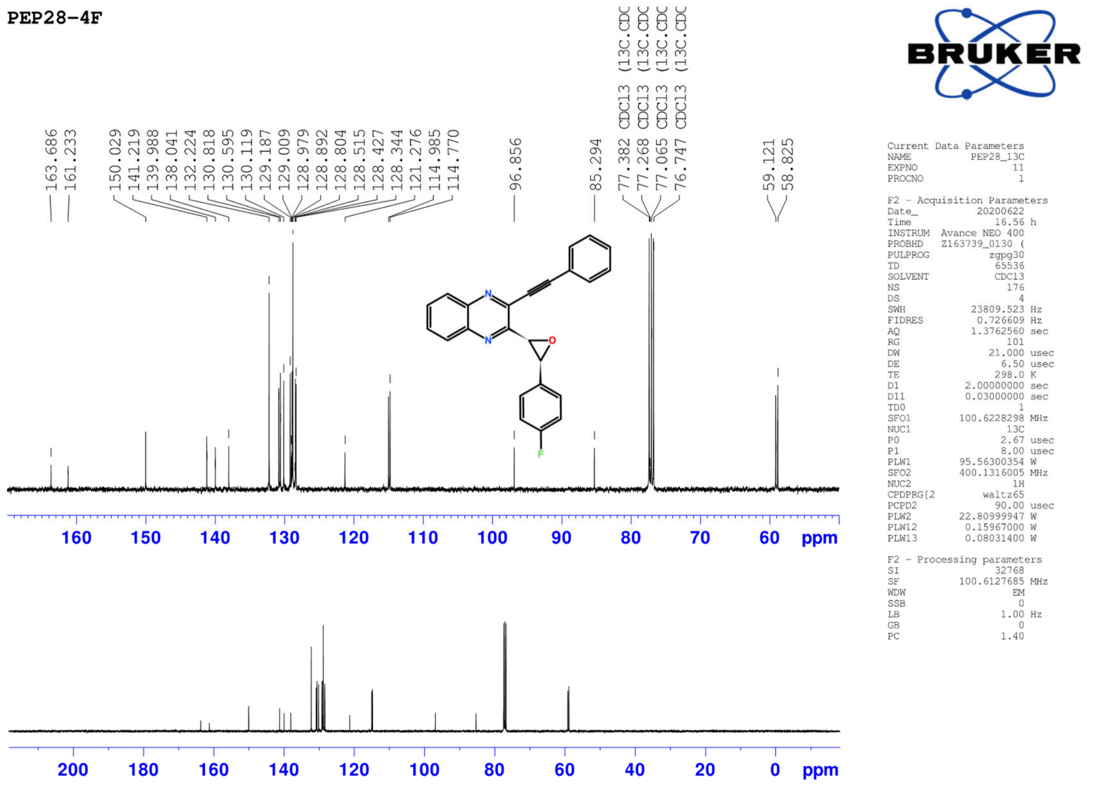

```
Data File C:\Chem32\...rganique\prestation_chimie_organique 2022-05-31\OnlineEdited--026.D
Sample Name: PEP28

=====================================================================
Acq. Operator   : SYSTEM                          Seq. Line :  26
Acq. Instrument : LC1290                           Location :   P1-C7
Injection Date  : 31/5/2022 7:47:03 PM                  Inj :   1
                                                Inj Volume : 1.000 µl
Method          : C:\Chem32\1\Data\prestation_chimie_organique\prestation_chimie_organique
                  2022-05-31\prestation_chimie_org.M (Sequence Method)
Last changed    : 31/5/2022 1:07:56 PM by SYSTEM
Additional Info : Peak(s) manually integrated
```

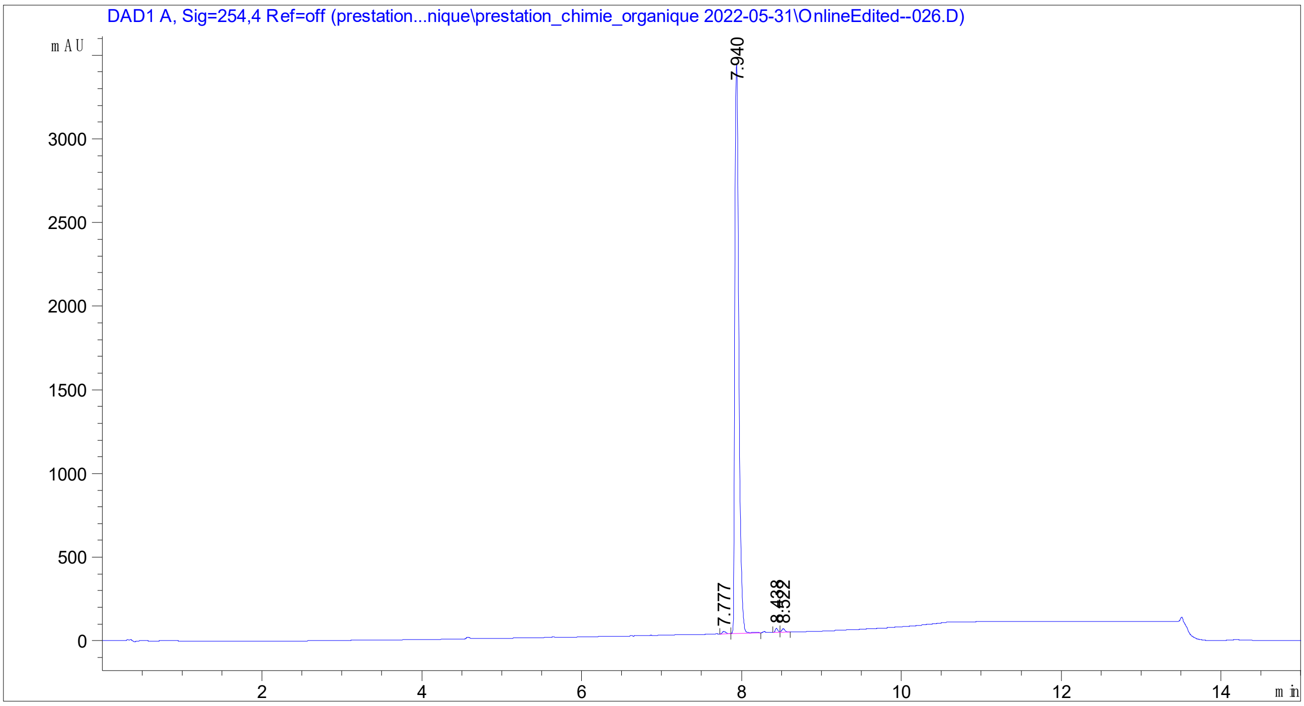


```
=====================================================================
                         Area Percent Report
=====================================================================

Sorted By             :      Signal
Multiplier            :      1.0000
Dilution              :      1.0000
Use Multiplier & Dilution Factor with ISTDs


Signal 1: DAD1 A, Sig=254,4 Ref=off

Peak RetTime Type  Width     Area      Height     Area
  #   [min]        [min]   [mAU*s]     [mAU]        %
----|-------|----|-------|----------|----------|--------|
   1   7.777 BB    0.0422   45.23986   15.91781   0.3853
   2   7.940 BV R  0.0537 1.15784e4 3399.18823  98.6096
   3   8.438 BV    0.0373   59.40652   24.52583   0.5059
   4   8.522 VB    0.0395   58.61246   21.73516   0.4992

Totals :                  1.17417e4  3461.36704


=====================================================================
```

Compound 20a

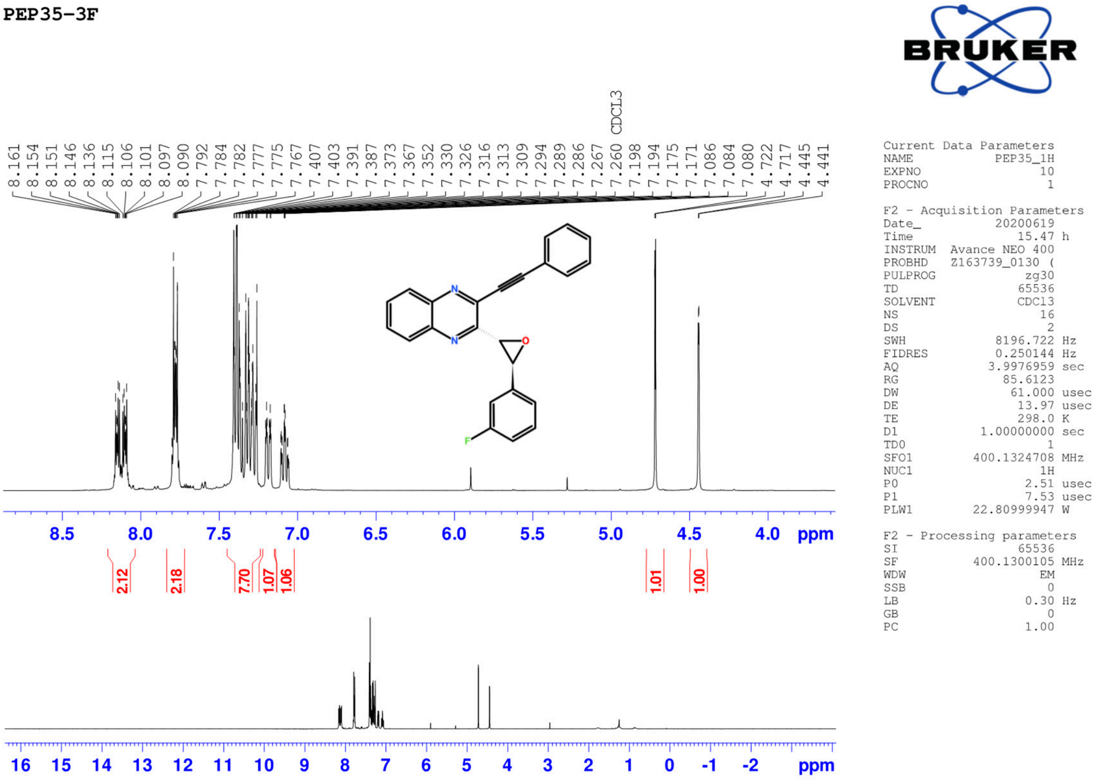

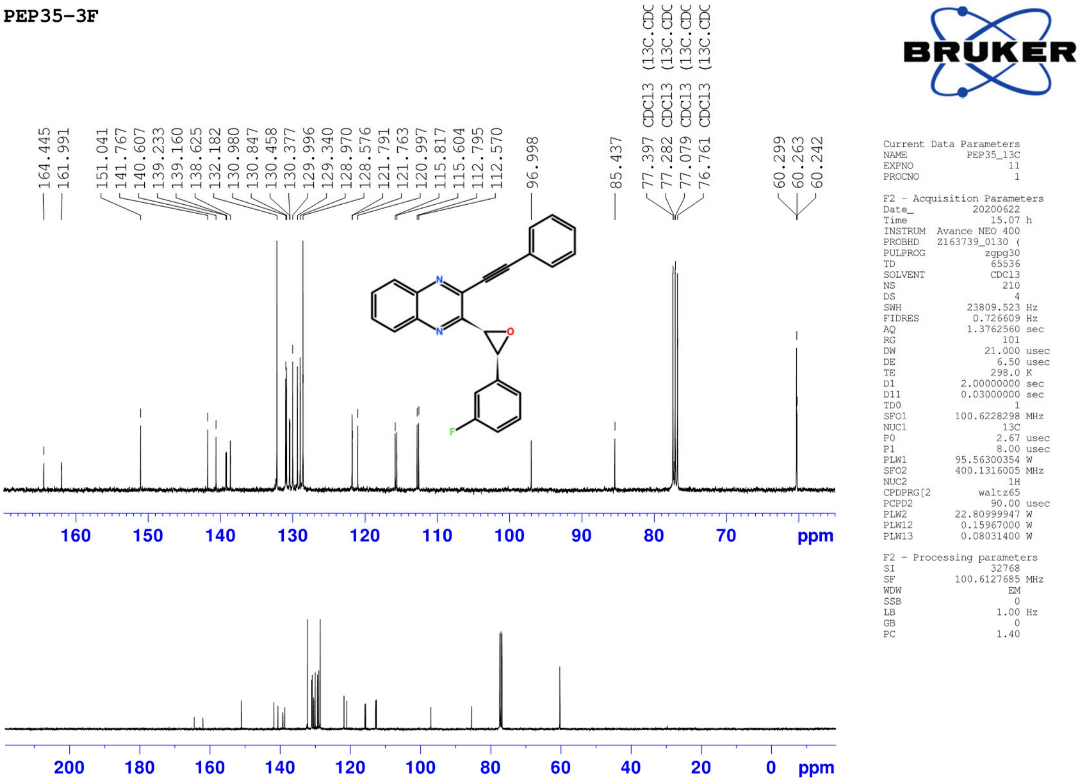

Compound 20b

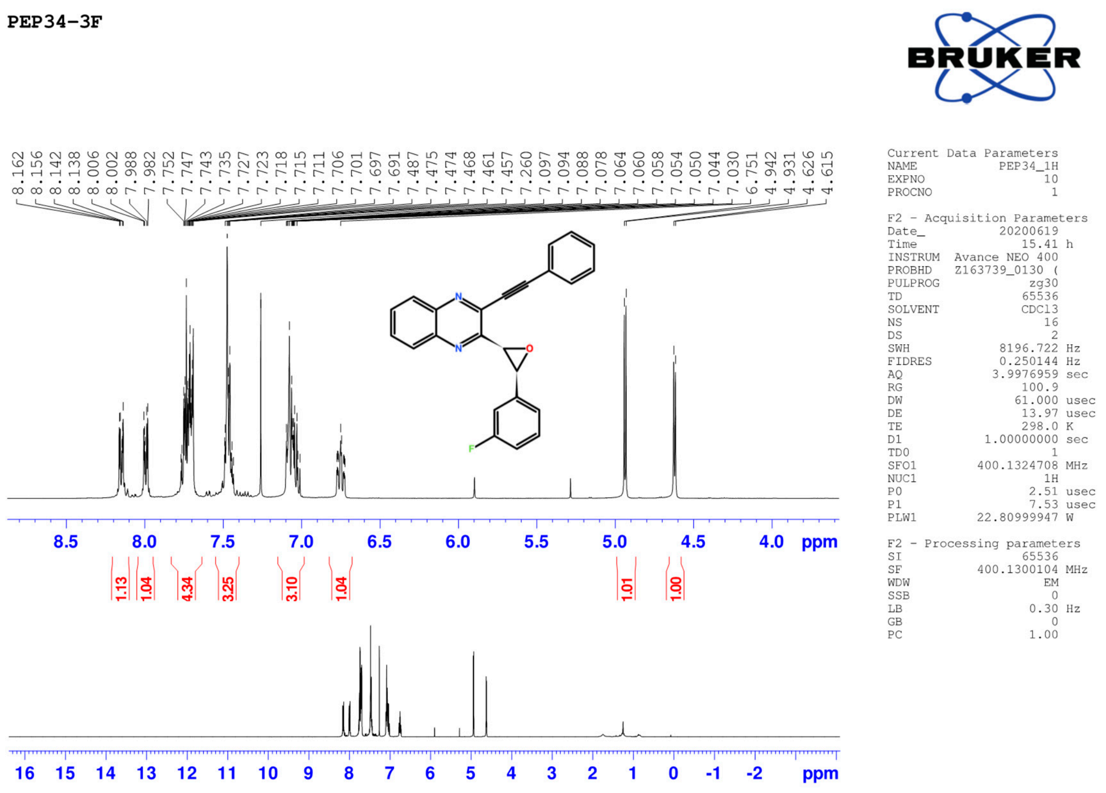

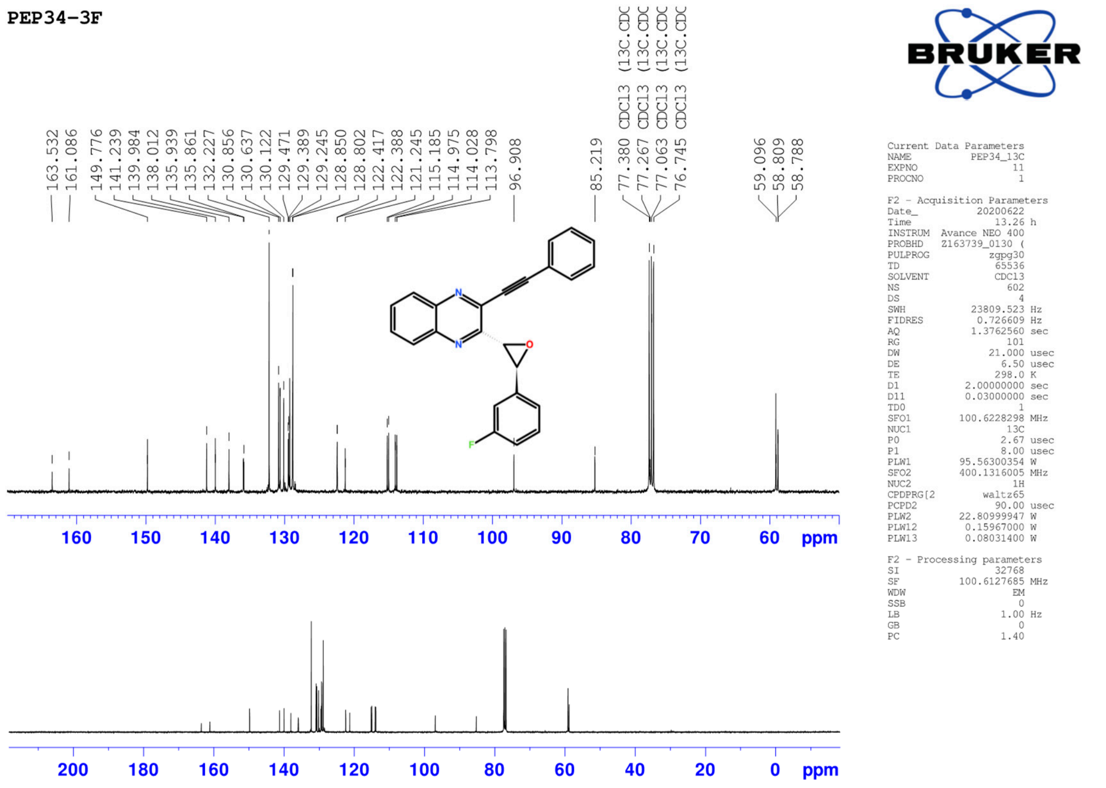

```
Data File C:\Chem32\...rganique\prestation_chimie_organique 2022-05-31\OnlineEdited--031.D
Sample Name: PEP34

    =====================================================================
    Acq. Operator   : SYSTEM                          Seq. Line :   31
    Acq. Instrument : LC1290                           Location :   P1-D3
    Injection Date  : 31/5/2022 9:06:01 PM                  Inj :   1
                                                    Inj Volume : 1.000 µl
    Method          : C:\Chem32\1\Data\prestation_chimie_organique\prestation_chimie_organique
                      2022-05-31\prestation_chimie_org.M (Sequence Method)
    Last changed    : 31/5/2022 1:07:56 PM by SYSTEM
    Additional Info : Peak(s) manually integrated
```

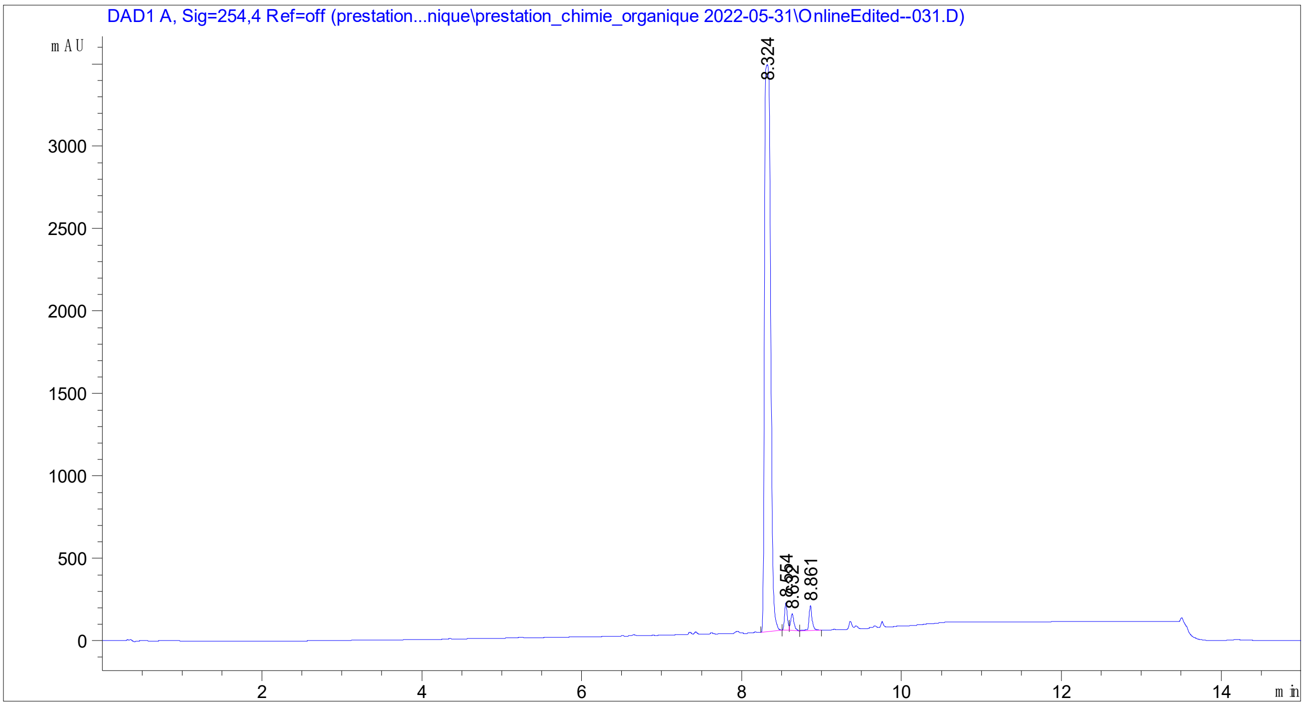


```
    =====================================================================
                         Area Percent Report
    =====================================================================

    Sorted By             :      Signal
    Multiplier            :      1.0000
    Dilution              :      1.0000
    Use Multiplier & Dilution Factor with ISTDs


    Signal 1: DAD1 A, Sig=254,4 Ref=off
```

Peak #	RetTime [min]	Type	Width [min]	Area [mAU*s]	Height [mAU]	Area %
1	8.324	BB	0.0827	1.78862e4	3439.96216	94.2043
2	8.554	BV	0.0363	394.31558	168.49573	2.0768
3	8.632	VB	0.0444	300.44666	101.93674	1.5824
4	8.861	VB R	0.0397	405.65085	151.90590	2.1365

Totals : 1.89866e4 3862.30053

```
    =====================================================================
```

Compound 21a

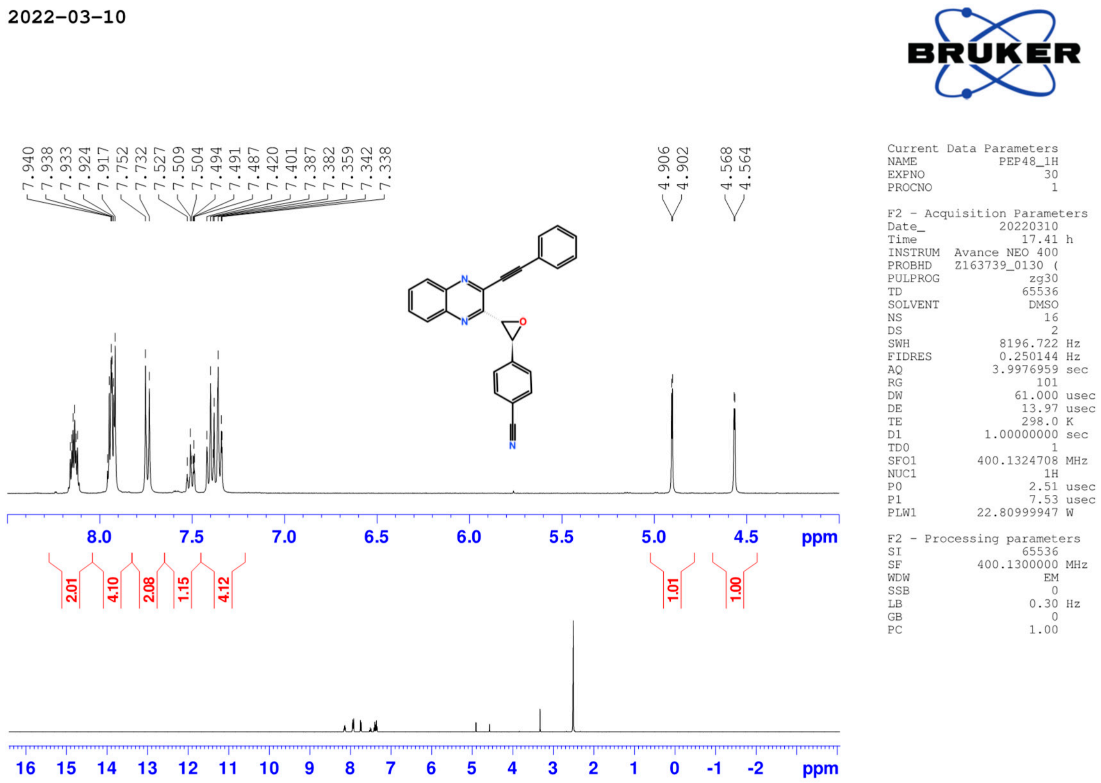

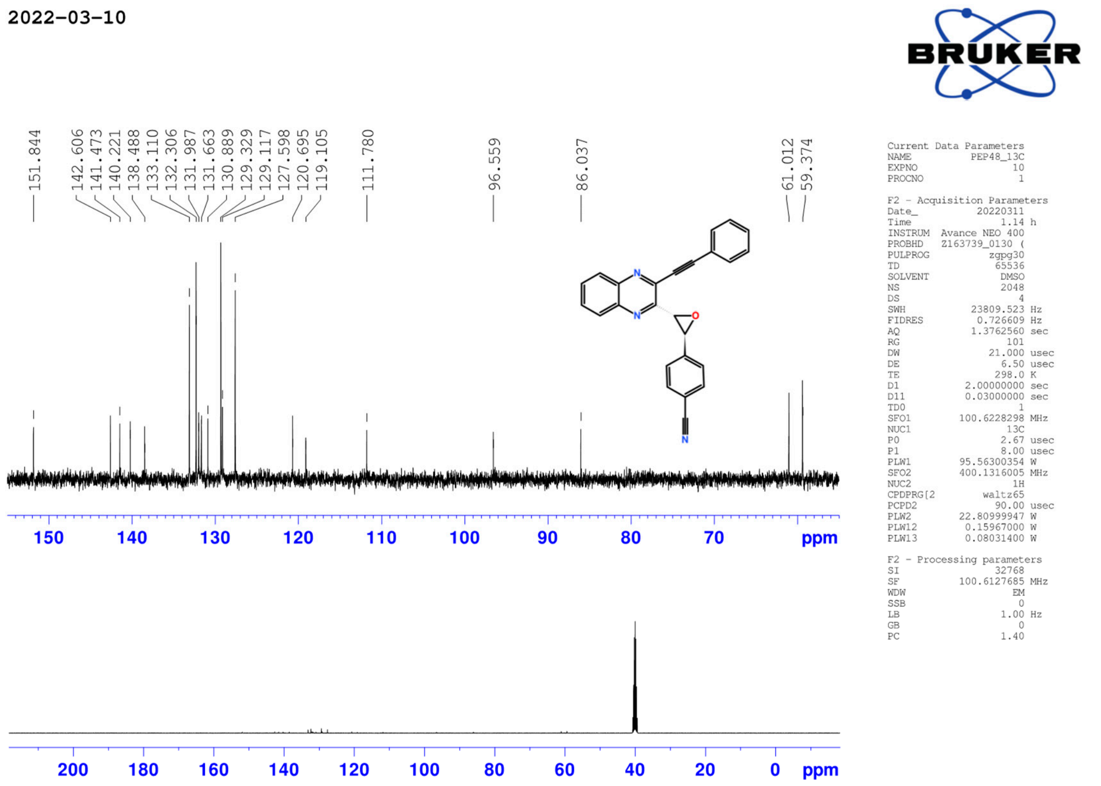

Data File C:\Chem32\...rganique\prestation_chimie_organique 2022-05-31\OnlineEdited--039.D
Sample Name: PEP48

===
Acq. Operator : SYSTEM Seq. Line : 39
Acq. Instrument : LC1290 Location : P1-E2
Injection Date : 31/5/2022 11:12:11 PM Inj : 1
Inj Volume : 1.000 µl
Method : C:\Chem32\1\Data\prestation_chimie_organique\prestation_chimie_organique 2022-05-31\prestation_chimie_org.M (Sequence Method)
Last changed : 31/5/2022 1:07:56 PM by SYSTEM
Additional Info : Peak(s) manually integrated

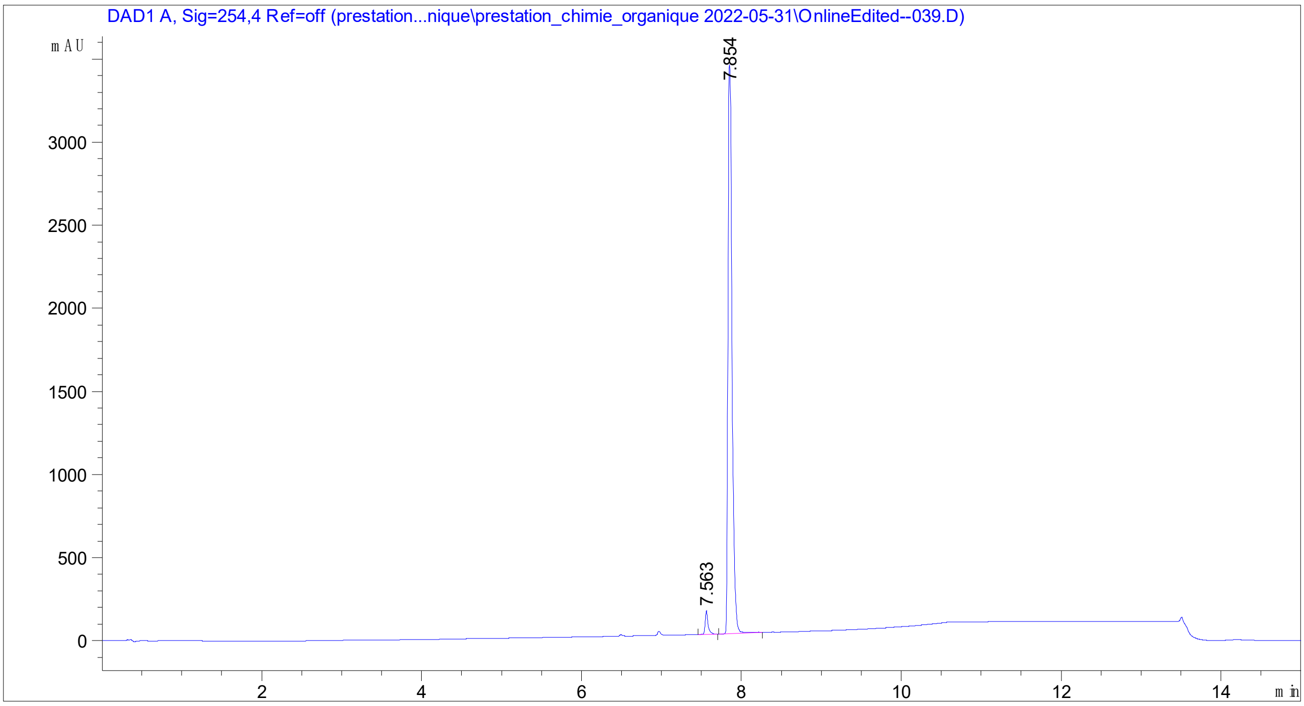

===
Area Percent Report
===

Sorted By : Signal
Multiplier : 1.0000
Dilution : 1.0000
Use Multiplier & Dilution Factor with ISTDs

Signal 1: DAD1 A, Sig=254,4 Ref=off

Peak #	RetTime [min]	Type	Width [min]	Area [mAU*s]	Height [mAU]	Area %
1	7.563	BB	0.0378	365.28397	143.02545	2.8536
2	7.854	BV R	0.0572	1.24355e4	3423.59668	97.1464

Totals : 1.28007e4 3566.62213

===
*** End of Report ***

Compound 21b

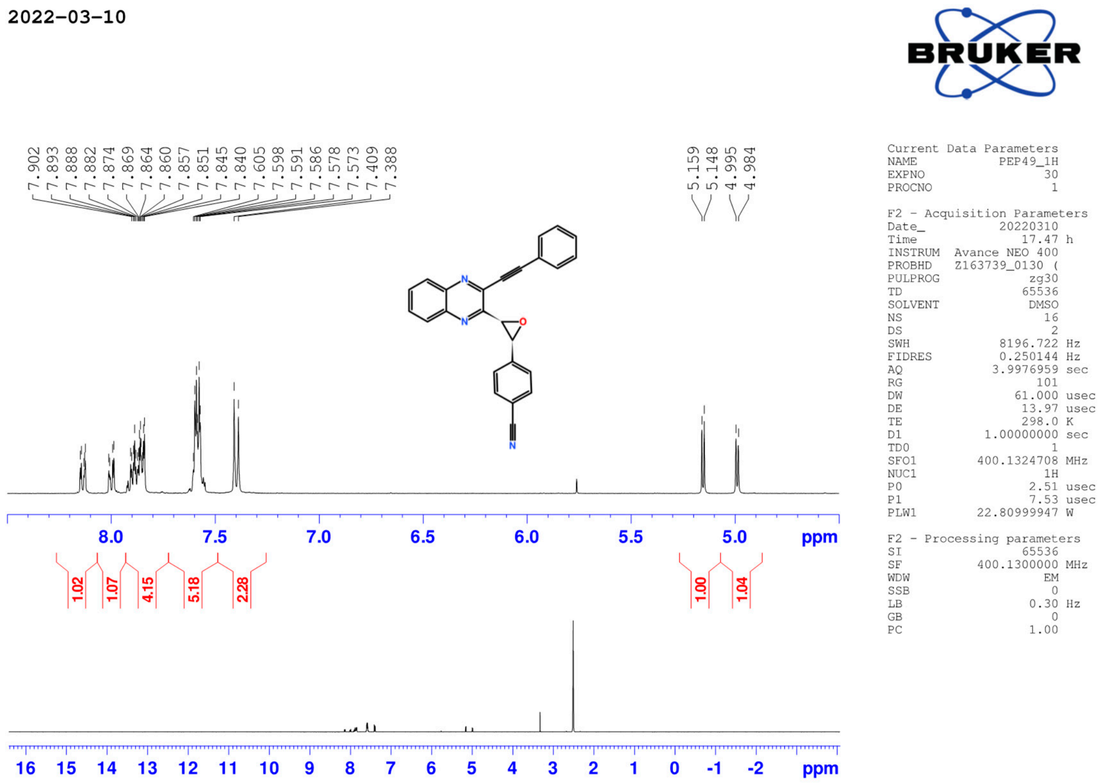

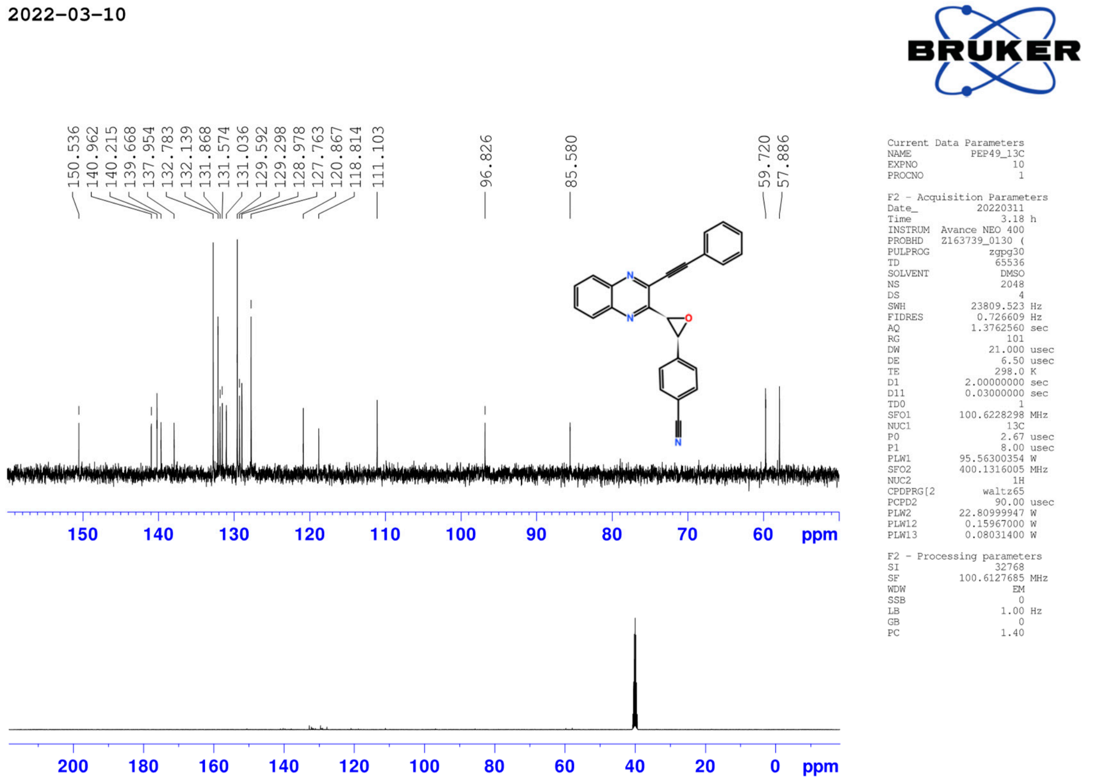

Compound 22a

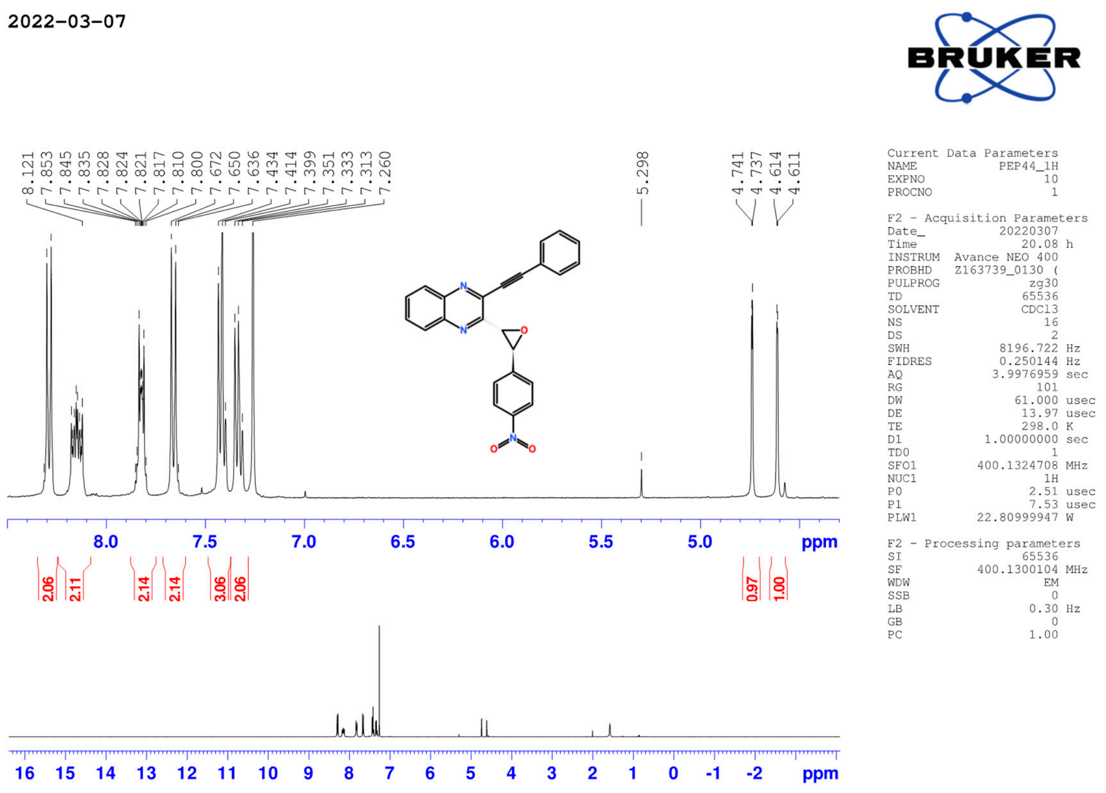

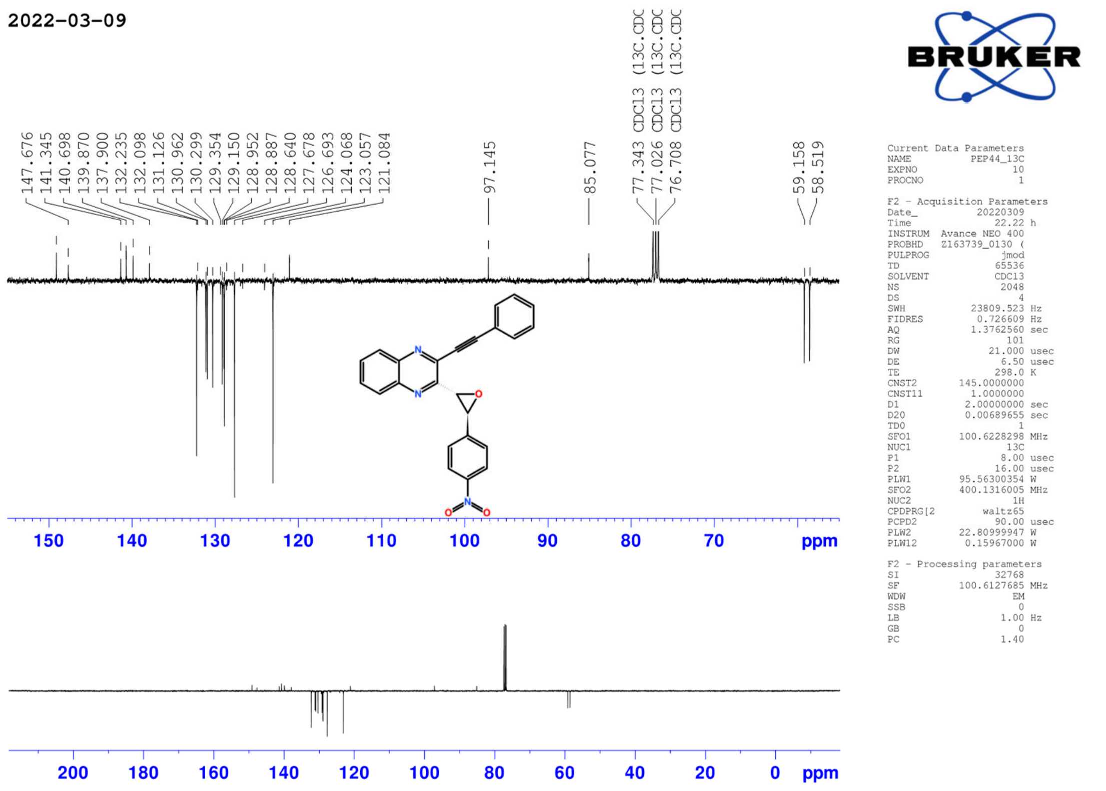

Data File C:\Chem32\...rganique\prestation_chimie_organique 2022-05-31\OnlineEdited--035.D
Sample Name: PEP44

===
Acq. Operator : SYSTEM Seq. Line : 35
Acq. Instrument : LC1290 Location : P1-D7
Injection Date : 31/5/2022 10:09:07 PM Inj : 1
Inj Volume : 1.000 µl
Method : C:\Chem32\1\Data\prestation_chimie_organique\prestation_chimie_organique 2022-05-31\prestation_chimie_org.M (Sequence Method)
Last changed : 31/5/2022 1:07:56 PM by SYSTEM
Additional Info : Peak(s) manually integrated

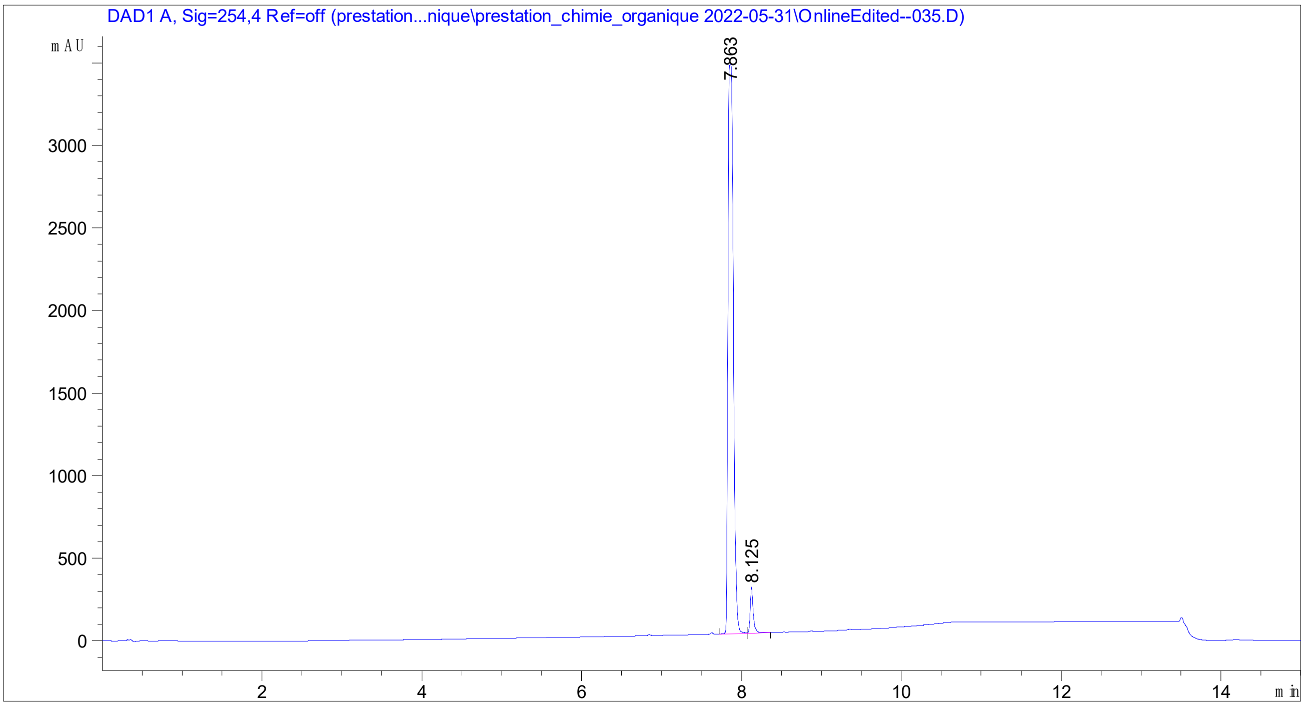

===
Area Percent Report
===

Sorted By : Signal
Multiplier : 1.0000
Dilution : 1.0000
Use Multiplier & Dilution Factor with ISTDs

Signal 1: DAD1 A, Sig=254,4 Ref=off

Peak #	RetTime [min]	Type	Width [min]	Area [mAU*s]	Height [mAU]	Area %
1	7.863	BV	0.0745	1.61083e4	3445.96948	95.7239
2	8.125	VV R	0.0385	719.57733	275.56274	4.2761

Totals : 1.68279e4 3721.53223

===
*** End of Report ***

Compound 22b

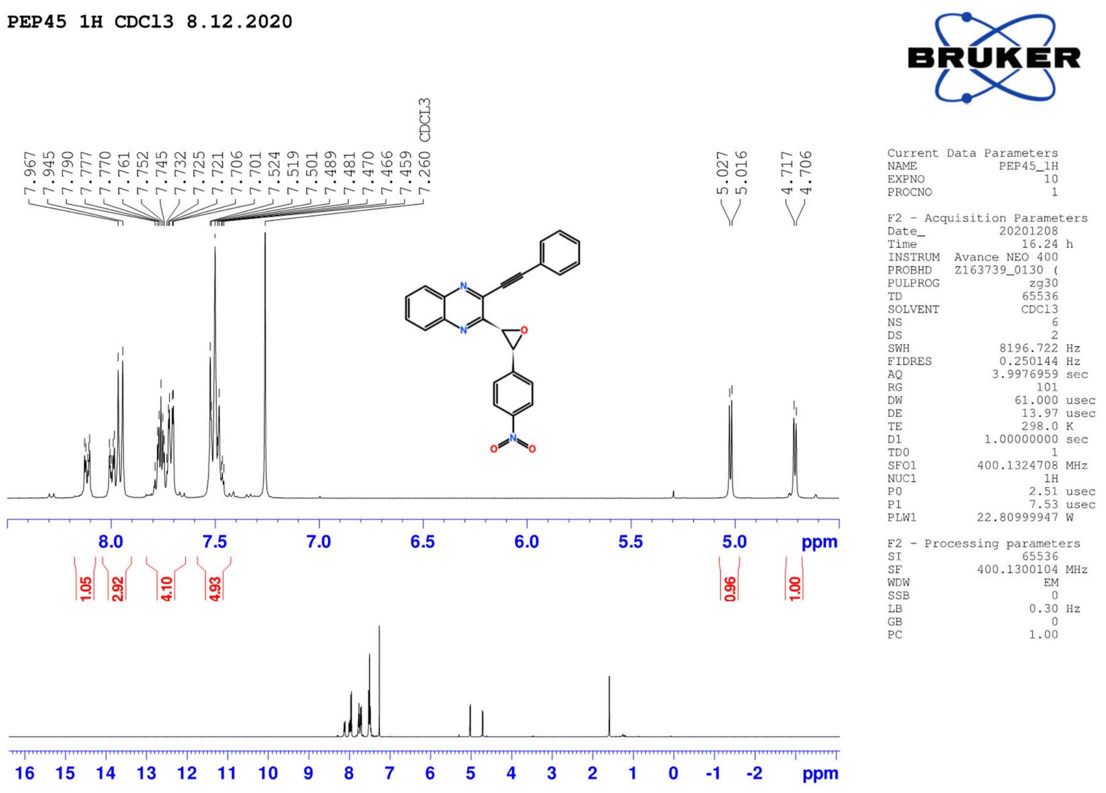

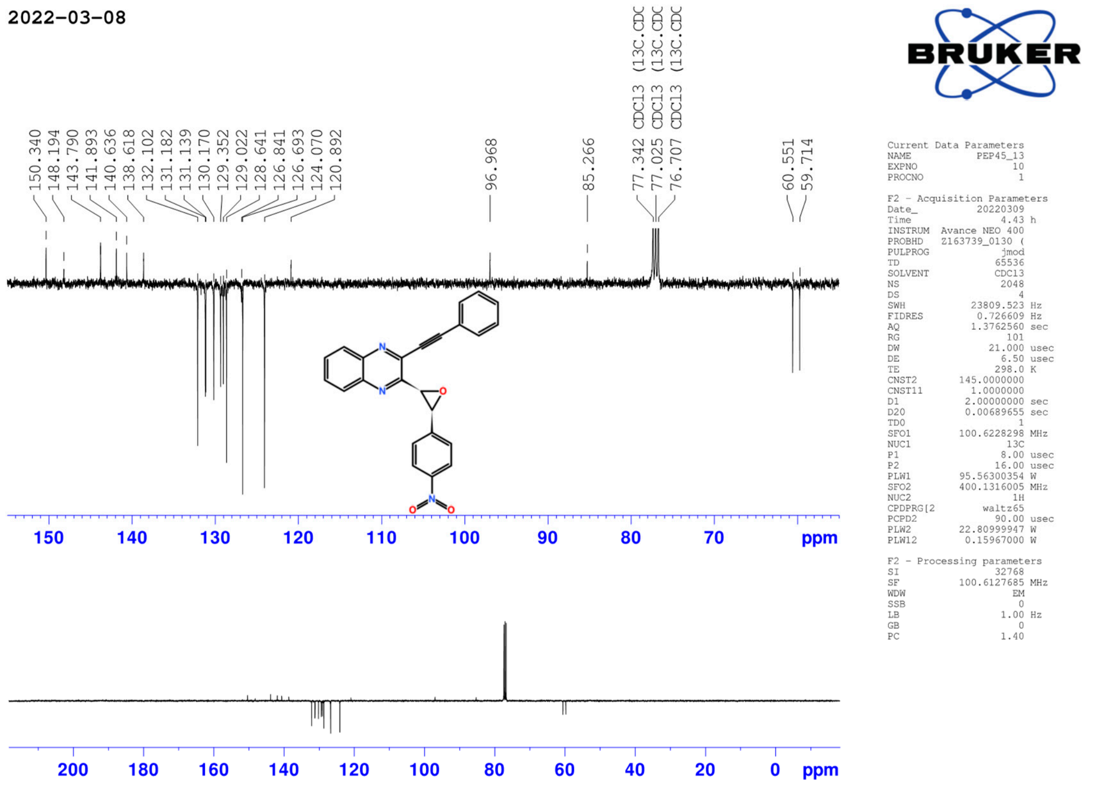

Data File C:\Chem32\...rganique\prestation_chimie_organique 2022-05-31\OnlineEdited--036.D
Sample Name: PEP45

===

Acq. Operator : SYSTEM Seq. Line : 36
Acq. Instrument : LC1290 Location : P1-D8
Injection Date : 31/5/2022 10:24:53 PM Inj : 1
Inj Volume : 1.000 µl
Method : C:\Chem32\1\Data\prestation_chimie_organique\prestation_chimie_organique 2022-05-31\prestation_chimie_org.M (Sequence Method)
Last changed : 31/5/2022 1:07:56 PM by SYSTEM
Additional Info : Peak(s) manually integrated

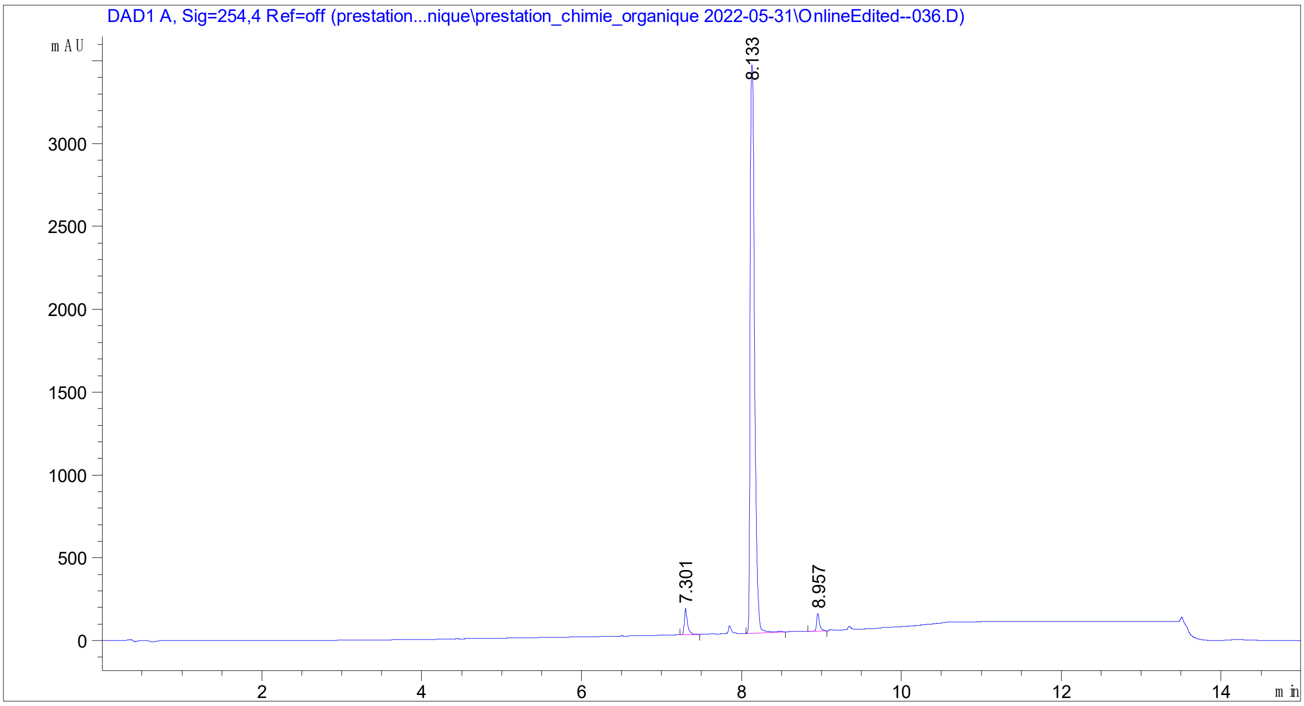

===

Area Percent Report

===

Sorted By : Signal
Multiplier : 1.0000
Dilution : 1.0000
Use Multiplier & Dilution Factor with ISTDs

Signal 1: DAD1 A, Sig=254,4 Ref=off

Peak #	RetTime [min]	Type	Width [min]	Area [mAU*s]	Height [mAU]	Area %
1	7.301	BV R	0.0410	451.15408	159.28886	3.2742
2	8.133	BV R	0.0594	1.30718e4	3431.33911	94.8666
3	8.957	BB	0.0364	256.19012	105.13464	1.8593

Totals : 1.37791e4 3695.76262

===

*** End of Report ***

Compound 23a

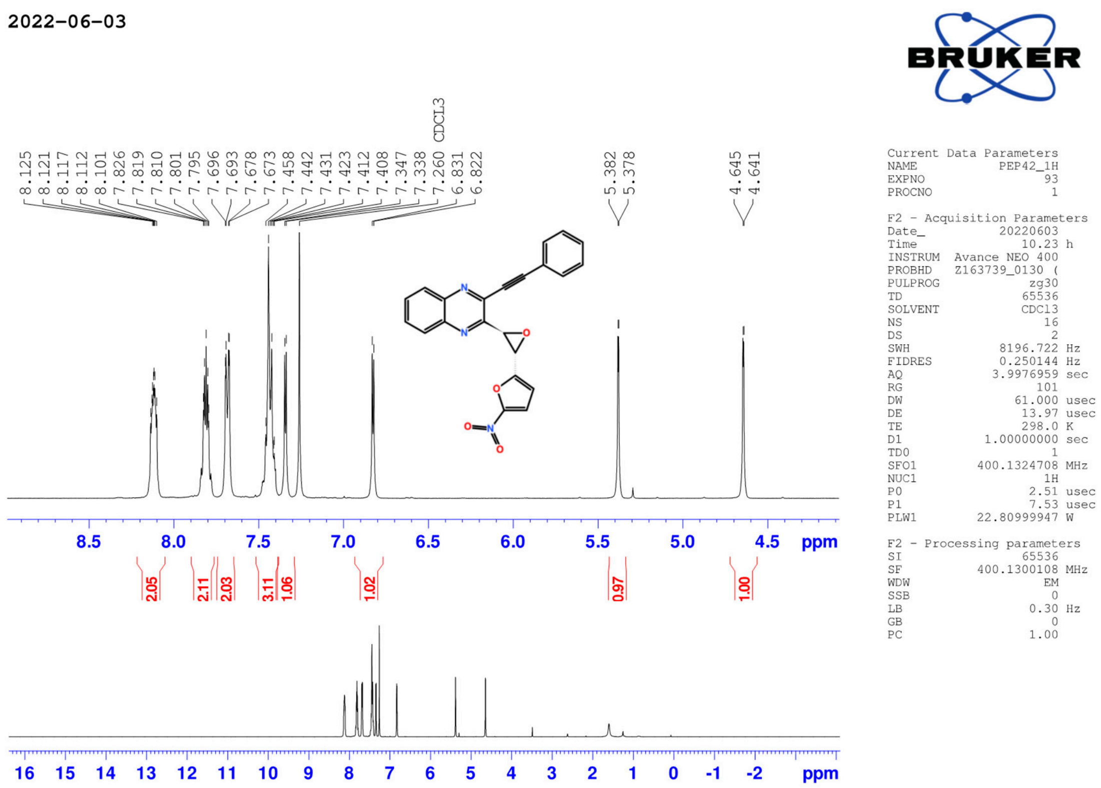

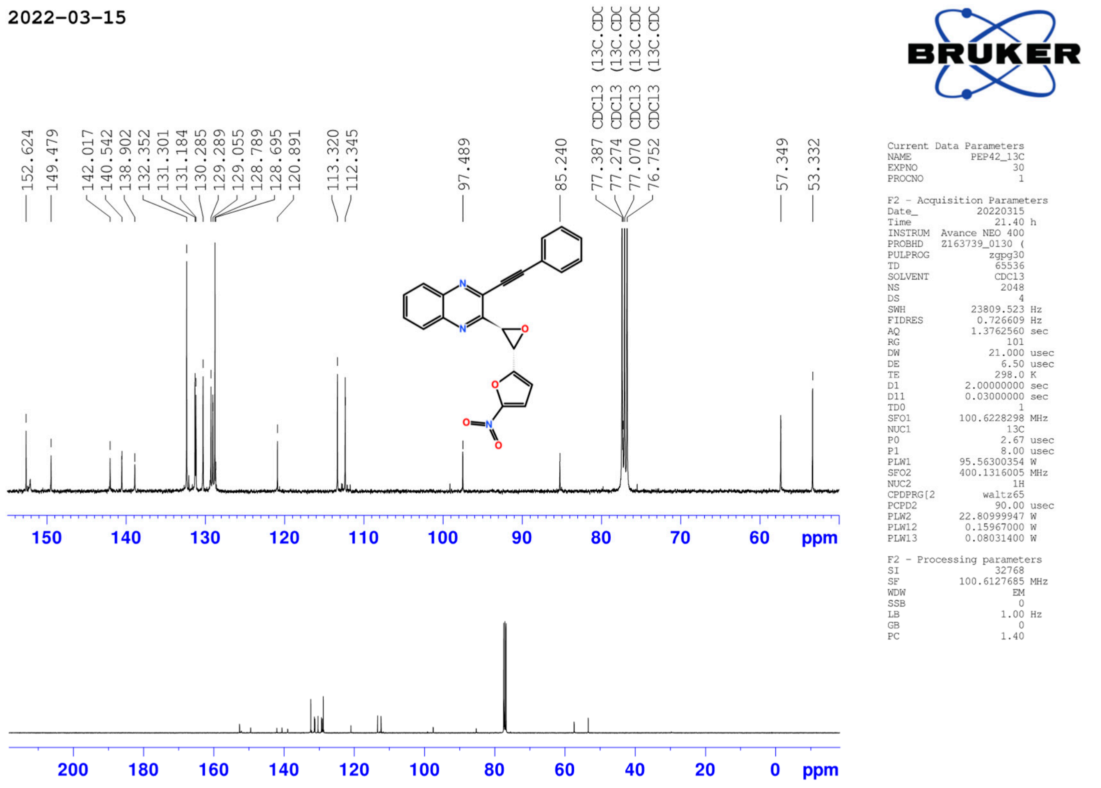

Compound 23

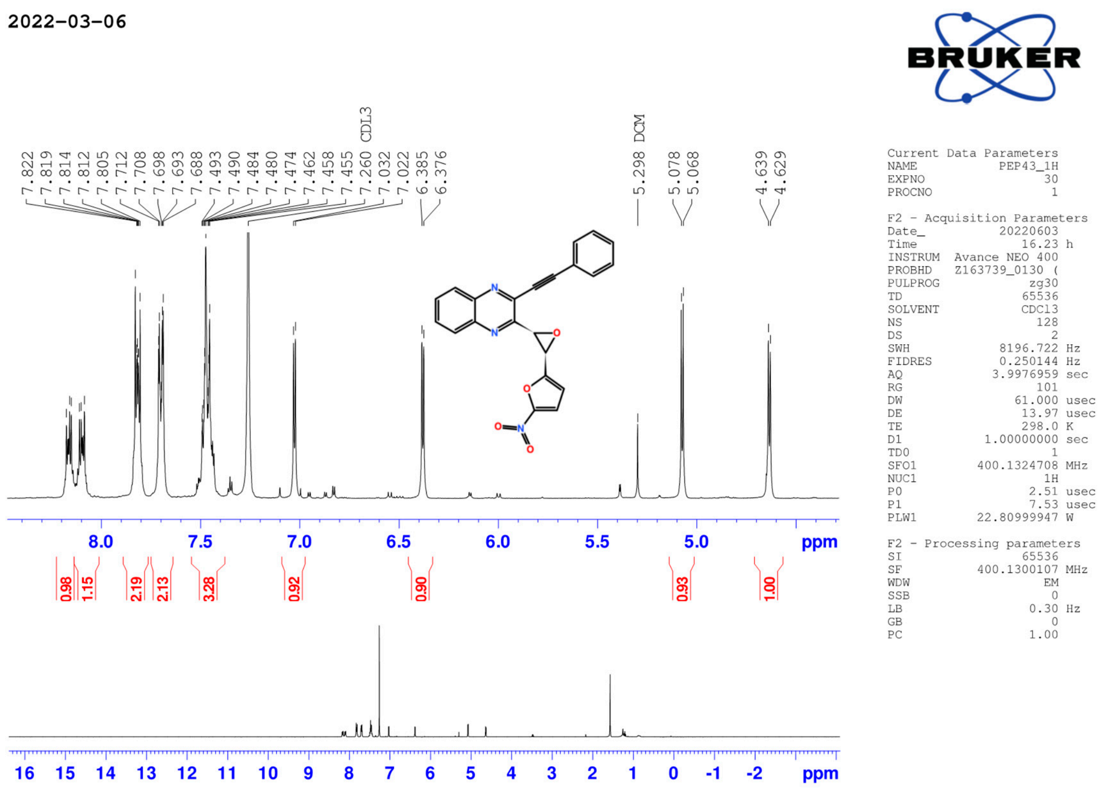
2022-03-06
BRUKER
7.822
7.819
7.814
7.812
7.805
7.712
7.708
7.698
7.693
7.688
7.493
7.490
7.484
7.480
7.474
7.462
7.458
7.455
7.260 CDL3
7.032
7.022
6.385
6.376
5.298 DCM
5.078
5.068
4.639
4.629
0.98
1.15
2.19
2.13
3.28
0.92
0.90
0.93
1.00
8.0 7.5 7.0 6.5 6.0 5.5 5.0 ppm
16 15 14 13 12 11 10 9 8 7 6 5 4 3 2 1 0 -1 -2 ppm
Current Data Parameters
NAME PEP43_1H
EXPNO 30
PROCNO 1
F2 - Acquisition Parameters
Date_ 20220603
Time 16.23 h
INSTRUM Avance NEO 400
PROBHD Z163739_0130 (
PULPROG zg30
TD 65536
SOLVENT CDCl3
NS 128
DS 2
SWH 8196.722 Hz
FIDRES 0.250144 Hz
AQ 3.9976959 sec
RG 101
DW 61.000 usec
DE 13.97 usec
TE 298.0 K
D1 1.00000000 sec
TD0 1
SFO1 400.1324708 MHz
NUC1 1H
P0 2.51 usec
P1 7.53 usec
PLW1 22.80999947 W
F2 - Processing parameters
SI 65536
SF 400.1300107 MHz
WDW EM
SSB 0
LB 0.30 Hz
GB 0
PC 1.00

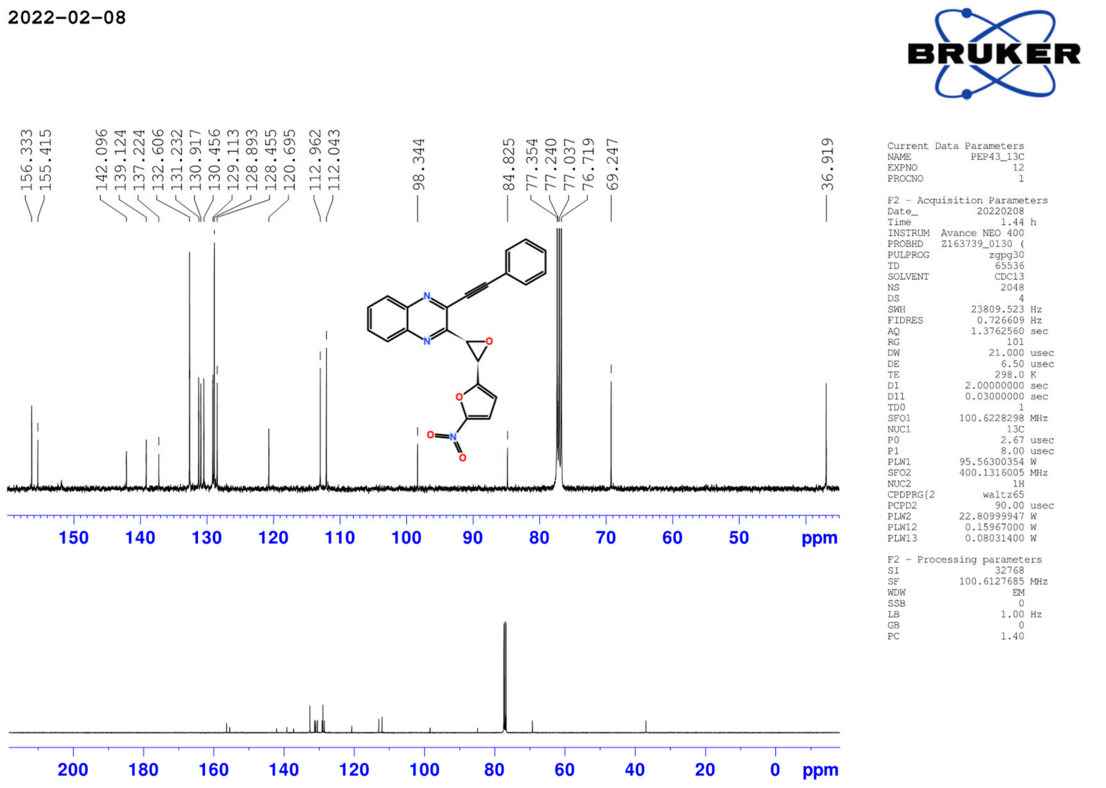
2022-02-08
BRUKER
156.333
155.415
142.096
139.124
137.224
132.606
131.232
130.917
130.456
129.113
128.893
128.455
120.695
112.962
112.043
98.344
84.825
77.354
77.240
77.037
76.719
69.247
36.919
150 140 130 120 110 100 90 80 70 60 50 ppm
200 180 160 140 120 100 80 60 40 20 0 ppm
Current Data Parameters
NAME PEP43_13C
EXPNO 12
PROCNO 1
F2 - Acquisition Parameters
Date_ 20220208
Time 1.44 h
INSTRUM Avance NEO 400
PROBHD Z163739_0130 (
PULPROG zgpg30
TD 65536
SOLVENT CDCl3
NS 2048
DS 4
SWH 23809.523 Hz
FIDRES 0.726609 Hz
AQ 1.3762560 sec
RG 101
DW 21.000 usec
DE 6.50 usec
TE 298.0 K
D1 2.00000000 sec
D11 0.03000000 sec
TD0 1
SFO1 100.6228298 MHz
NUC1 13C
P0 2.67 usec
P1 8.00 usec
PLW1 95.56300354 W
SFO2 400.1316005 MHz
NUC2 1H
CPDPRG[2 waltz65
PCPD2 90.00 usec
PLW2 22.80999947 W
PLW12 0.15967000 W
PLW13 0.08031400 W
F2 - Processing parameters
SI 32768
SF 100.6127685 MHz
WDW EM
SSB 0
LB 1.00 Hz
GB 0
PC 1.40

Compound 24a

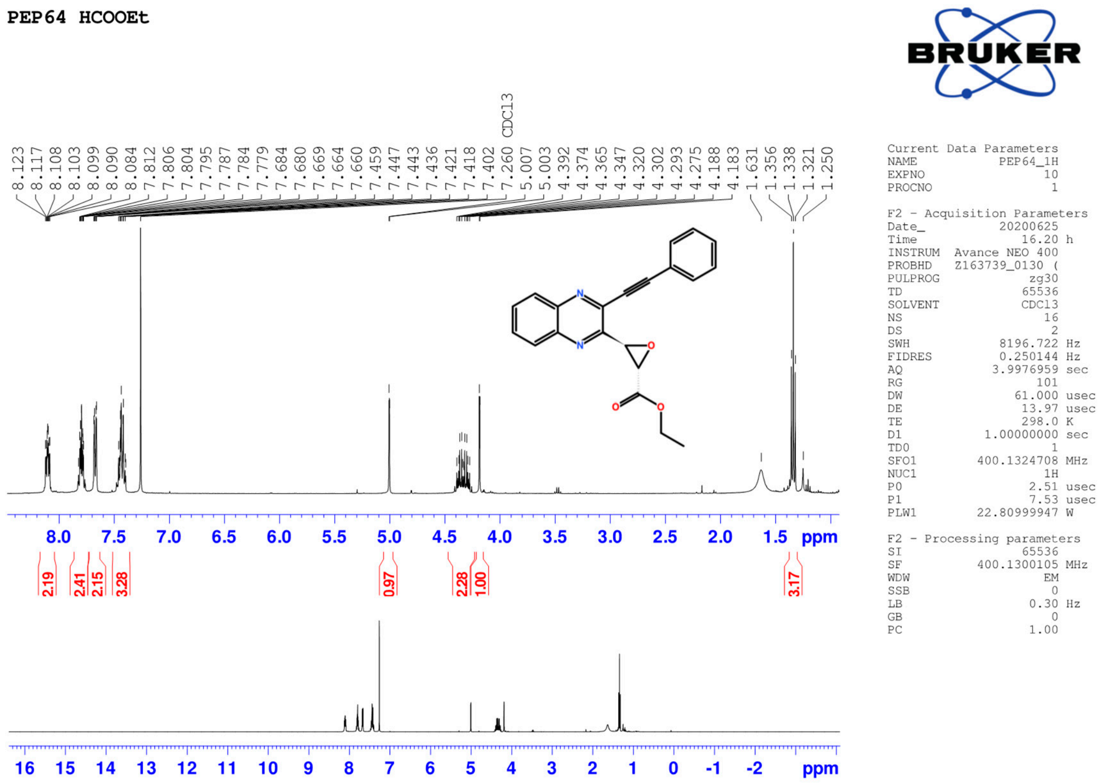

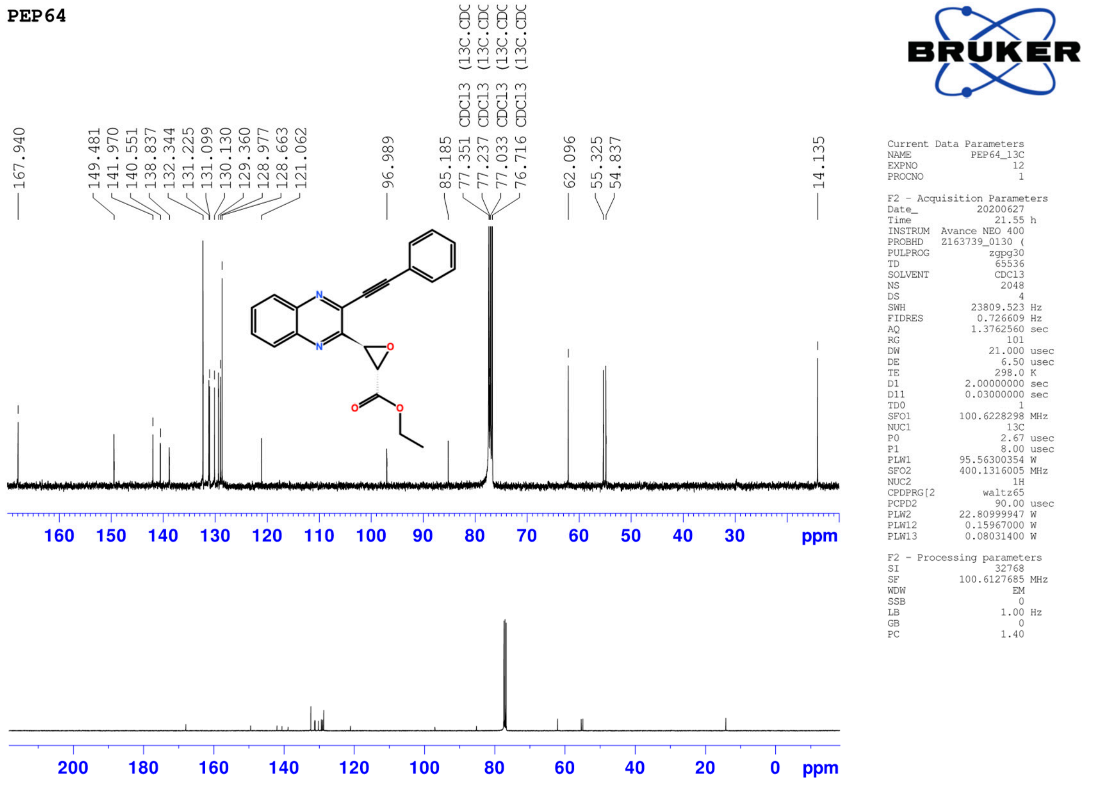

Data File C:\Chem32\...rganique\prestation_chimie_organique 2022-05-31\OnlineEdited--040.D
Sample Name: PEP64

===

Acq. Operator : SYSTEM Seq. Line : 40
Acq. Instrument : LC1290 Location : P1-E3
Injection Date : 31/5/2022 11:27:59 PM Inj : 1
Inj Volume : 1.000 µl
Method : C:\Chem32\1\Data\prestation_chimie_organique\prestation_chimie_organique 2022-05-31\prestation_chimie_org.M (Sequence Method)
Last changed : 31/5/2022 1:07:56 PM by SYSTEM
Additional Info : Peak(s) manually integrated

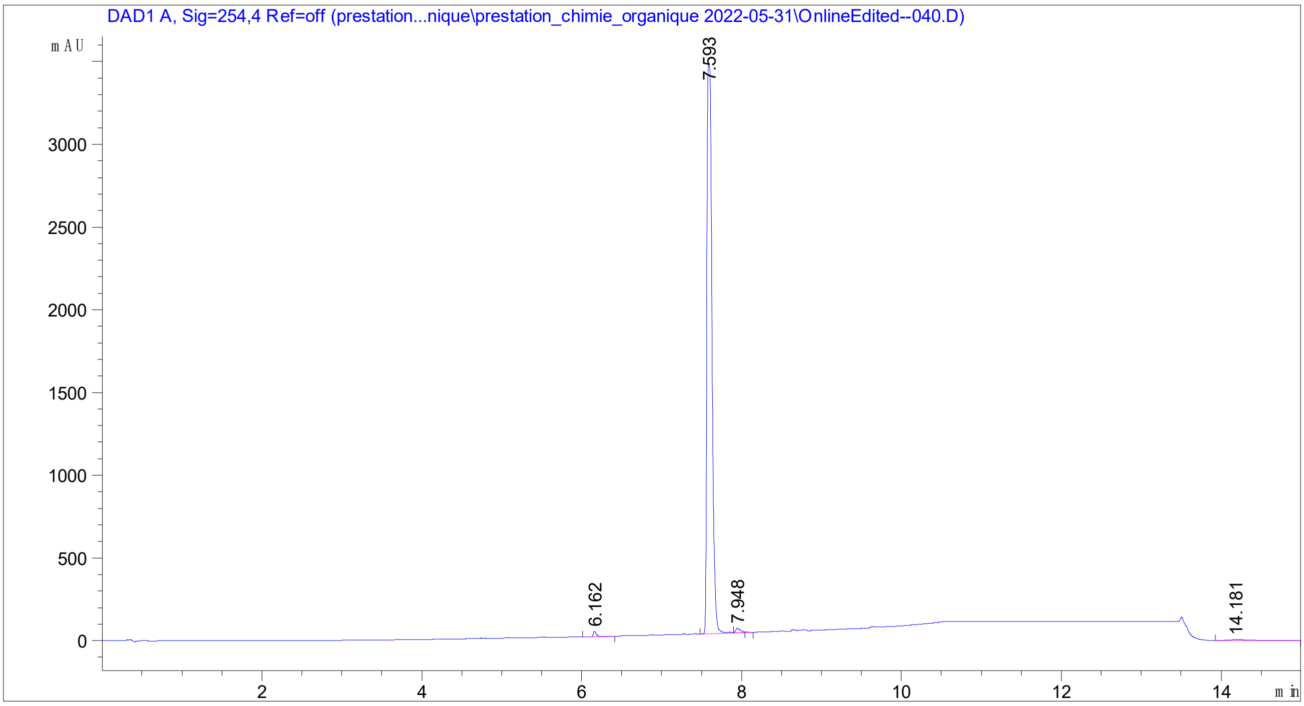

===

Area Percent Report

===

Sorted By : Signal
Multiplier : 1.0000
Dilution : 1.0000
Use Multiplier & Dilution Factor with ISTDs

Signal 1: DAD1 A, Sig=254,4 Ref=off

Peak #	RetTime [min]	Type	Width [min]	Area [mAU*s]	Height [mAU]	Area %
1	6.162	BV R	0.0395	91.22353	31.71634	0.5877
2	7.593	BV R	0.0704	1.51796e4	3439.07544	97.7983
3	7.948	VV E	0.0582	120.64300	29.18264	0.7773
4	14.181	BBA	0.3004	129.86763	6.10058	0.8367

Totals : 1.55214e4 3506.07499

===

Compound 25

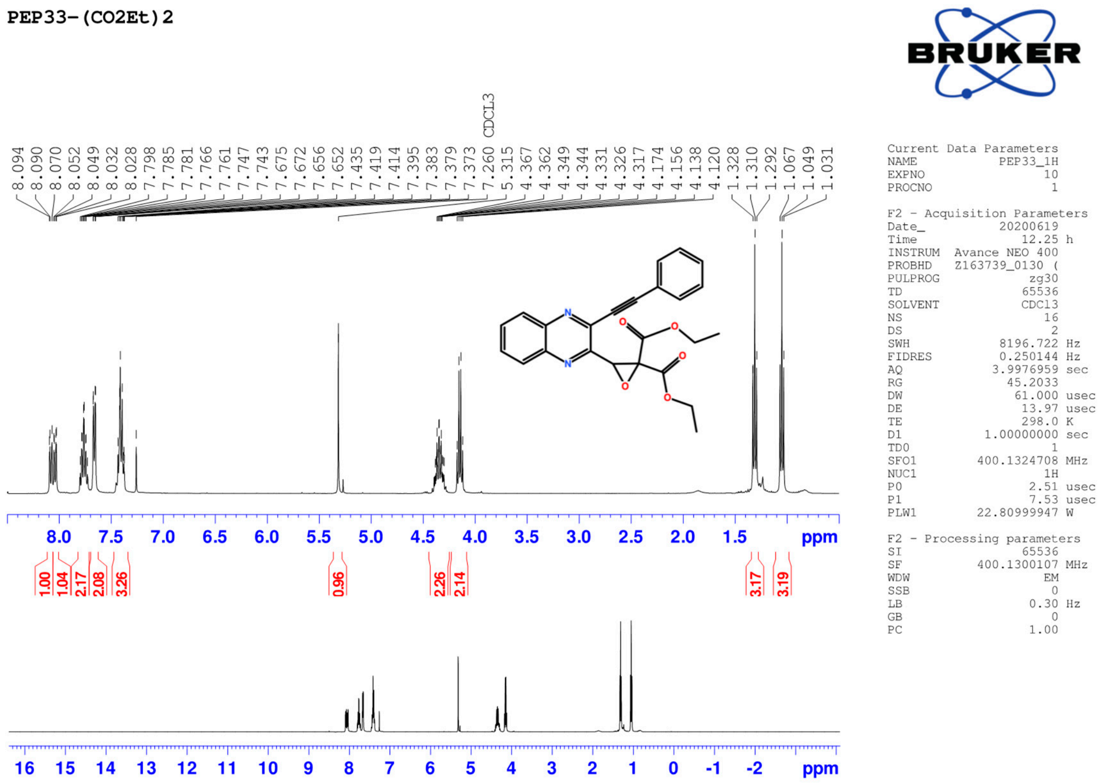
PEP33-(CO2Et)2
8.094
8.090
8.070
8.052
8.049
8.032
8.028
7.798
7.785
7.781
7.766
7.761
7.747
7.743
7.675
7.672
7.656
7.652
7.435
7.419
7.414
7.395
7.383
7.379
7.373
7.260 CDCL3
5.315
4.367
4.362
4.349
4.344
4.331
4.326
4.317
4.174
4.156
4.138
4.120
1.328
1.310
1.292
1.067
1.049
1.031
1.00
1.04
2.17
2.08
3.26
0.96
2.26
2.14
3.17
3.19
8.0 7.5 7.0 6.5 6.0 5.5 5.0 4.5 4.0 3.5 3.0 2.5 2.0 1.5 ppm
16 15 14 13 12 11 10 9 8 7 6 5 4 3 2 1 0 -1 -2 ppm
BRUKER
Current Data Parameters
NAME PEP33_1H
EXPNO 10
PROCNO 1
F2 - Acquisition Parameters
Date_ 20200619
Time 12.25 h
INSTRUM Avance NEO 400
PROBHD Z163739_0130 (
PULPROG zg30
TD 65536
SOLVENT CDCl3
NS 16
DS 2
SWH 8196.722 Hz
FIDRES 0.250144 Hz
AQ 3.9976959 sec
RG 45.2033
DW 61.000 usec
DE 13.97 usec
TE 298.0 K
D1 1.00000000 sec
TD0 1
SFO1 400.1324708 MHz
NUC1 1H
P0 2.51 usec
P1 7.53 usec
PLW1 22.80999947 W
F2 - Processing parameters
SI 65536
SF 400.1300107 MHz
WDW EM
SSB 0
LB 0.30 Hz
GB 0
PC 1.00

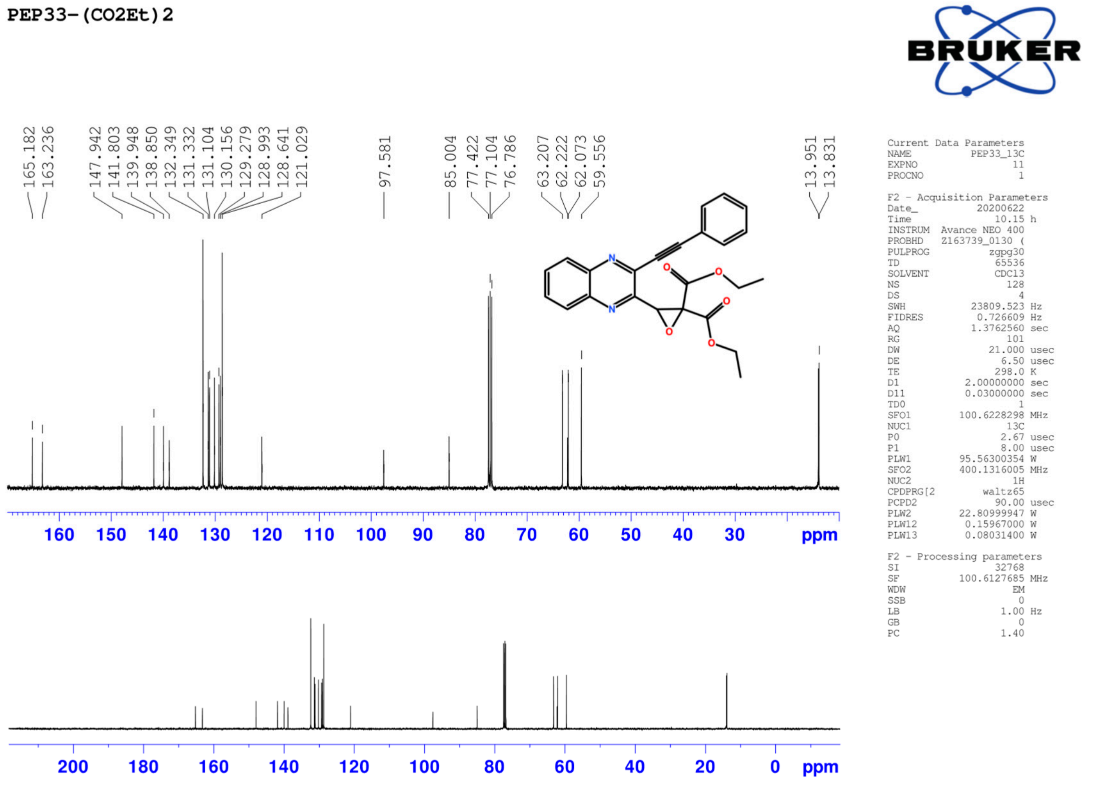
PEP33-(CO2Et)2
165.182
163.236
147.942
141.803
139.948
138.850
132.349
131.332
131.104
130.156
129.279
128.993
128.641
121.029
97.581
85.004
77.422
77.104
76.786
63.207
62.222
62.073
59.556
13.951
13.831
160 150 140 130 120 110 100 90 80 70 60 50 40 30 ppm
200 180 160 140 120 100 80 60 40 20 0 ppm
BRUKER
Current Data Parameters
NAME PEP33_13C
EXPNO 11
PROCNO 1
F2 - Acquisition Parameters
Date_ 20200622
Time 10.15 h
INSTRUM Avance NEO 400
PROBHD Z163739_0130 (
PULPROG zgpg30
TD 65536
SOLVENT CDCl3
NS 128
DS 4
SWH 23809.523 Hz
FIDRES 0.726609 Hz
AQ 1.3762560 sec
RG 101
DW 21.000 usec
DE 6.50 usec
TE 298.0 K
D1 2.00000000 sec
D11 0.03000000 sec
TD0 1
SFO1 100.6228298 MHz
NUC1 13C
P0 2.67 usec
P1 8.00 usec
PLW1 95.56300354 W
SFO2 400.1316005 MHz
NUC2 1H
CPDPRG[2 waltz65
PCPD2 90.00 usec
PLW2 22.80999947 W
PLW12 0.15967000 W
PLW13 0.08031400 W
F2 - Processing parameters
SI 32768
SF 100.6127685 MHz
WDW EM
SSB 0
LB 1.00 Hz
GB 0
PC 1.40

Data File C:\Chem32\...rganique\prestation_chimie_organique 2022-05-31\OnlineEdited--030.D
Sample Name: PEP33

===
Acq. Operator : SYSTEM Seq. Line : 30
Acq. Instrument : LC1290 Location : P1-D2
Injection Date : 31/5/2022 8:50:15 PM Inj : 1
Inj Volume : 1.000 µl
Method : C:\Chem32\1\Data\prestation_chimie_organique\prestation_chimie_organique 2022-05-31\prestation_chimie_org.M (Sequence Method)
Last changed : 31/5/2022 1:07:56 PM by SYSTEM
Additional Info : Peak(s) manually integrated

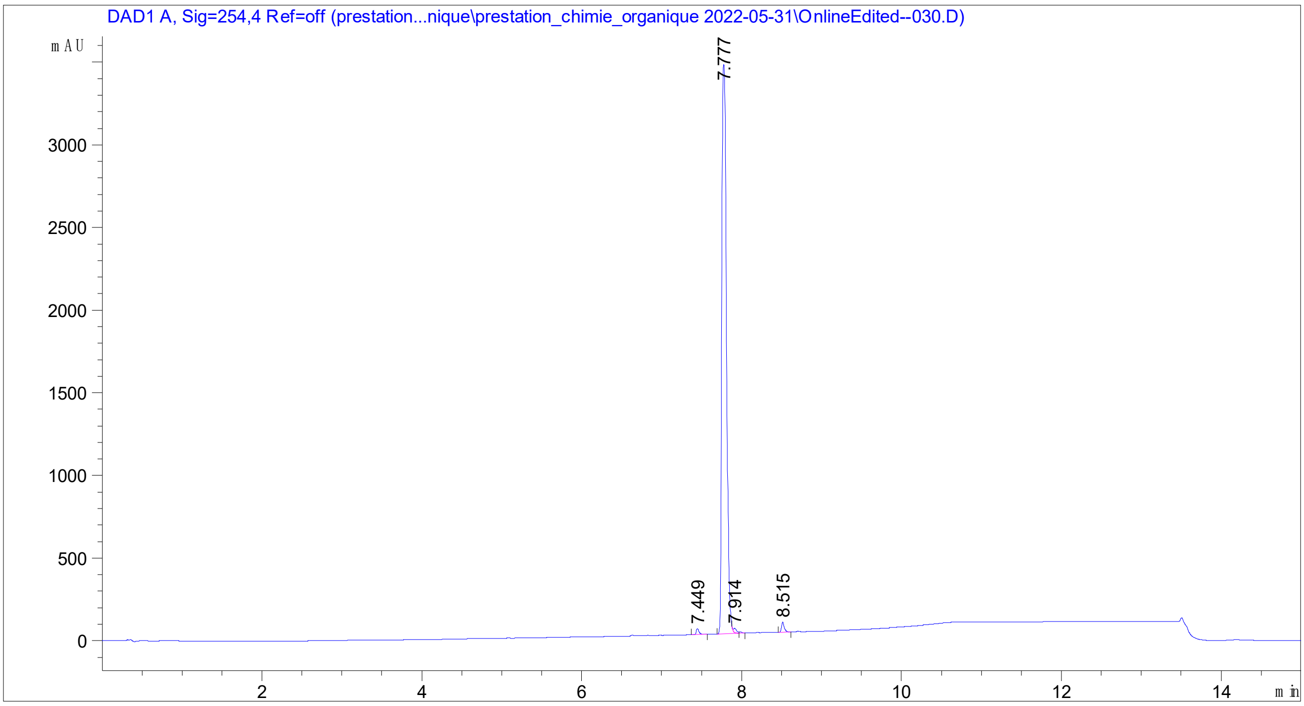

===
Area Percent Report
===

Sorted By : Signal
Multiplier : 1.0000
Dilution : 1.0000
Use Multiplier & Dilution Factor with ISTDs

Signal 1: DAD1 A, Sig=254,4 Ref=off

Peak #	RetTime [min]	Type	Width [min]	Area [mAU*s]	Height [mAU]	Area %
1	7.449	BV R	0.0375	91.06236	36.00343	0.6481
2	7.777	BV R	0.0625	1.37244e4	3442.15552	97.6824
3	7.914	VV E	0.0399	75.30146	27.51309	0.5360
4	8.515	BB	0.0384	159.26624	63.13708	1.1336

Totals : 1.40500e4 3568.80912

===
